# Direct Synthesis of Various Thioesters from Acyl Fluorides and Thiosilanes through the Assistance of a Si–F Bond Formation

**DOI:** 10.1021/acs.joc.5c01677

**Published:** 2025-10-02

**Authors:** Ryuki Takeuchi, Kento Ishida, Norio Sakai

**Affiliations:** Department of Pure and Applied Chemistry, Faculty of Science and Technology, Tokyo University of Science (RIKADAI), Noda, Chiba 278-8519, Japan

## Abstract

Herein it is described that the coupling reactions of aromatic and aliphatic acyl fluorides with phenyl trimethylsilyl sulfide proceeded smoothly under neat conditions to produce thioester derivatives. The addition of a catalytic amount of typical bases, such as triethylamine (Et_3_N) and potassium butoxide (KO^*t*^Bu), effectively activates thiosilanes, which could facilitate the coupling reactions of aroyl fluorides with a variety of aryl/alkyl thiosilanes containing an alkyl group, a halogen, an ester, or a heterocyclic ring to produce a variety of thioesters in practical yields.

## Graphical abstract

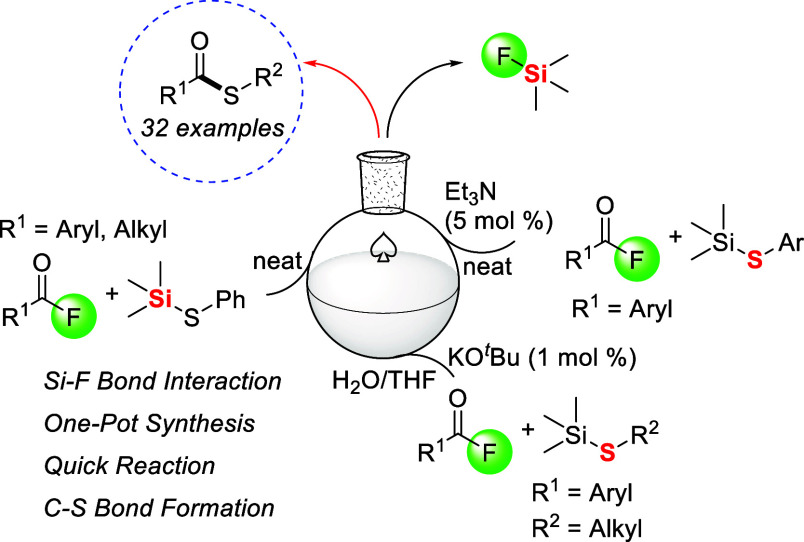

## Introduction

Thioesters constitute one of the fundamental functional groups in organic chemistry and have also been utilized in polymer science and biochemistry.^1^ For example, acyl CoA, which is a type of thioester derivative, is an important component of tricarboxylic acid (TCA) cycle.^2^ Moreover, thioesters have been employed as synthetic intermediates. Thioesters react with organometallic reagents in the presence of transition-metal catalysts to afford ketone derivatives,^3^ and the reduction of thioesters with hydrosilanes in the presence of palladium on carbon (Pd/C) produces various aldehydes (the Fukuyama reduction).^4^ Moreover, thioesters can be easily transformed into esters,^5^ sulfides,^6^ or sulfinate esters^7^ in the presence of various transition metal catalysts.

Thioesters are generally synthesized from carboxylic acids and thiol derivatives. The classical synthetic method involves the coupling of acyl chlorides with thiols in the presence of a base.^8^ To promote these transformations, various additives, such as CsF-Celite,^9a,9b^ a phase-transfer catalyst,^9c^ or 1,1,3,3,3-hexafluoroisopropan-2-ol (HFIP),^9d^ have been added (Scheme 1a). Condensation between carboxylic acids and thiols with a condensation reagent, such as *N,N’*-dicyclohexylcarbodiimide (DCC), also induces a reliable preparation of thioesters (Scheme 1b).^10^ In addition to these methods, synthetic methodologies with acyl fluorides, which are generally less reactive and easier to handle than acyl chlorides and have recently attracted a lot of interest in our community,^11^ have also been developed (Scheme 1c). For example, Arisawa and Yamaguchi et al. prepared thioesters from acyl fluorides and disulfides in the presence of a rhodium­(I)-catalyst and triphenylphosphine.^12^ Manabe et al. reported the stepwise synthesis of thioesters from acyl fluorides generated in situ from *N*-formylsaccharin, bromobenzene, and potassium fluoride in the presence of a palladium catalyst and thiols in the presence of triethylamine as a base.^13^

**Scheme 1:**
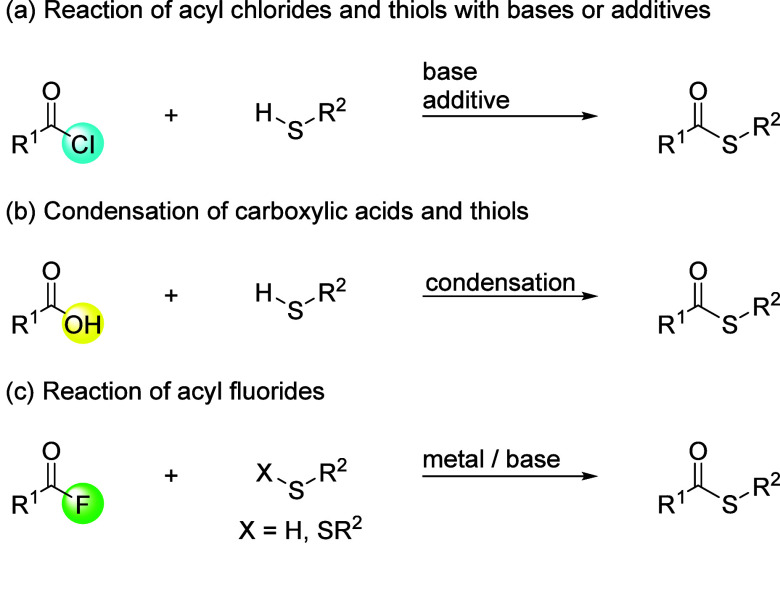
Approaches to Thioesters through Carboxylic Acid Derivatives and Thiol Derivatives

Other facile approaches involving the reactions of acyl chlorides with metal thiolates, such as thallium thiolates,^14a^ ^b^ tin thiolates,^14c^ copper thiolates,^14d^ germanium thiolates,^14e^ mercurial thiolates,^14f^ and zinc thiolates,^14g^ ^h^ have also been developed (Scheme 2a).^14^ As a more practical preparation, the coupling reaction of acyl halides with easy-to-handle thiosilanes could enable us to effectively produce a variety of thioester derivatives (Scheme 2b).^15^ Talley reported the synthesis of thioesters through the reaction of acyl chlorides and thiosilanes without any additives.^16^ This reaction proceeds without an additive as a reaction promotor. However, the procedure in this reaction requires the heating conditions under the solution to undertake the desired coupling reaction. Ando et al. found that the addition of potassium fluoride (KF)/18-crown-6 to the same reaction mixture promotes the preparation of thioesters.^15^ In contrast, the effective and useful synthesis of thioesters through the coupling of acyl fluorides and thiosilanes has not been studied extensively except for one trial, in which Ando et al. examined the coupling reaction of benzoyl fluoride with ethyl trimethylsilyl sulfide, but the expected thioester was not obtained (Scheme 2c).^15^ On the other hand, these methods still need stoichiometric amounts of base and a high temperature to start the desired reaction. In some cases, the employment of metals, such as thallium, tin, and mercury, has an environmental impact. Therefore, the development of an effective and simple approach to produce various thioesters from acyl fluorides and thiosilanes has been in high demand. Recently, as examples utilizing the interaction of a Si–F bond, the photoredox-catalyzed couplings of acyl fluorides with organosilanes in the presence of N-heterocyclic carbene and the reaction of sulfonimidoyl fluorides with silyl-substituted alkynes have been reported.^17^ In addition, the synthesis of thioesters through the palladium-catalyzed coupling of in situ generated acyl fluoride intermediates with triisopropylsilyl thioesters was disclosed by Nakada et.al.^18^ Until now, as unique synthesis of thioesters, the practical methods involving three-component couplings of a carboxylic acid or an aldehyde, elemental sulfur, and a hydrocarbon,^19a,19b^ metal-catalyzed oxidative coupling of a carboxylic acid or an aldehyde with a disulfide,^19c–19e^ and an oxidative coupling of a methyl arene with a disulfide, have been achieved.^19f^

**Scheme 2:**
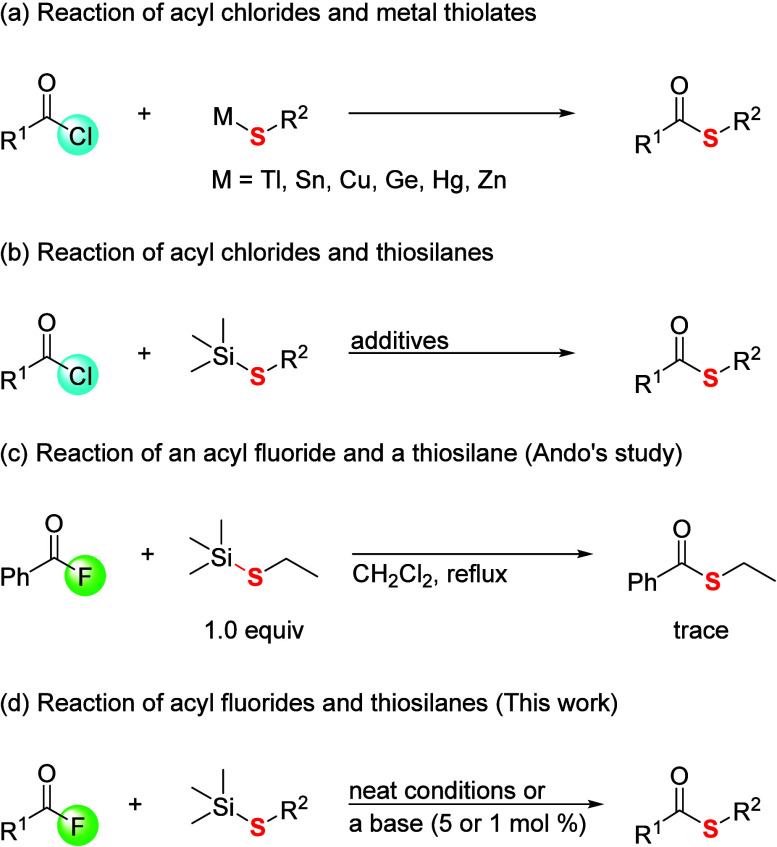
Approaches to Thioesters through Acyl Chlorides and Metal Thiolates or Acyl Fluorides and Thiosilane

In this context, we demonstrate various molecular transformations using these unique acyl fluorides. During our ongoing study on coupling reactions using acyl fluorides,^20^ we found that coupling of various acyl fluorides with trimethyl­(phenylthio)­silane proceeds under neat conditions at room temperature to effectively produce a variety of thioester derivatives (Scheme 2d). We also found that the catalytic addition of a typical base to reaction systems activated a Si–S bond of thiosilanes to skillfully improve the nucleophilicity of a sulfur atom, which led to facilitating subsequent C–S bond formation of thioesterification. Herein, we report the scope and limitations of this study.

## Results and Discussion

The reaction conditions were investigated using 3,5-dimethylbenzoyl fluoride (**1a**: 0.5 mmol) and trimethyl­(phenylthio)­silane (**2a**: 0.75 mmol) as the model substrate (Table 1 and Table S1 and S2). Initially, when acyl fluoride **1a** and thiosilane **2a**, which was prepared from benzoyl chloride and KF, were reacted at 80 °C for 1 h in toluene, the corresponding reaction did not proceed smoothly, forming the desired *S*-phenyl 3,5-dimethylbenzenecarbothioate **3aa** in only 2% yield (entry 1). Therefore, to activate thiosilane **2a**, the addition of several bases was examined. When the reaction was performed in the presence of a typical carbonate, such as Li_2_CO_3_ and K_2_CO_3_, the desired thioester **3aa** was obtained in moderate yields (entries 2 and 3). In contrast, CaCO_3_ was ineffective for the coupling reaction (entry 4). These results may depend on the low solubility of inorganic salts. Therefore, when a catalyst (5 mol %) of KF^18^ or potassium ethylxanthate (KEX) was added as the base, the yield of **3aa** remarkably improved to 89% and 97%, respectively (entries 5 and 6). The addition of triethylamine (Et_3_N) slightly decreased the yield of the thioester **3aa** (entry 7). In the case of KEX, thioester **3aa** was obtained in an acceptable yield, although thiosilane **2a** decreased to 1.1 equiv for acyl fluoride **1a** (entry 8). Moreover, conducting the coupling reaction at 60 °C, the yield of **3aa** did not decrease (entry 9). Surprisingly, the reaction proceeded effectively in the absence of solvents at room temperature to give the desired thioester **3aa** in nearly quantitative yield (entries 10 and 11). Moreover, under neat conditions, the reaction did not require the addition of a base to the reaction mixture, and the coupling reaction between **1a** and **2a** in equimolar amounts was completed within 1 min at room temperature (entries 12 and 13). After the evaporation of the reaction mixture, impurities such as Me_3_SiF were nearly absent, eliminating the need for standard purification.

**Table 1:**
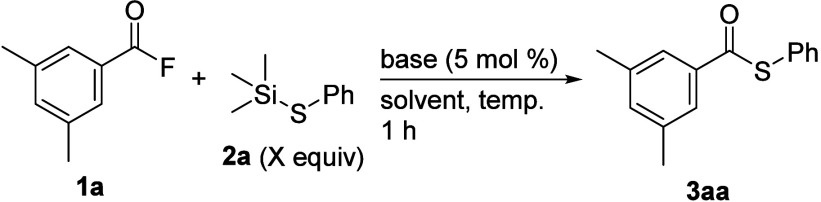
Optimization of the Reaction Conditions^a^

entry	base	X	solvent	temp	GC yield of **3aa**
		(equiv)		(°C)	(%)
1		1.5	toluene	80	2
2	Li_2_CO_3_	1.5	toluene	80	38
3	K_2_CO_3_	1.5	toluene	80	67
4	CaCO_3_	1.5	toluene	80	4
5	KF	1.5	toluene	80	89
6	KEX	1.5	toluene	80	97
7	Et_3_N	1.5	toluene	80	86
8	KEX	1.1	toluene	80	91
9	KEX	1.1	toluene	60	90
10	KEX	1.1		60	93
11	KEX	1.1		r.t.	92
12		1.1		r.t.	91
13^b^,^c^		1.0		r.t.	(95)

a **1a** (0.5 mmol), a base (5 mol %), a solvent (0.5 mL), 80 °C, 1 h.

b 1 min.

c Isolated yield.

The generality of the aromatic acyl fluorides was then examined under the optimal conditions (Scheme 3). First, the chemical properties and electronic effects of each substituent on aromatic acyl fluorides were examined. In the case of **1b**, which has an unsubstituted group, the desired thioester **3ba** was obtained in good yield. When using substrate **1** with either electron-donating or withdrawing groups, such as methyl, phenyl, or fluoro groups, the desired coupling reaction proceeded smoothly to give the corresponding thioesters **3ca**, **3da**, or **3ea** in good yields. Moreover, acyl fluorides **1f** having a 2,4,6-trimethyl group did not give the desired thioester **3fa**. These results show that the steric hindrance around the benzene ring of acyl fluorides has a significant effect on the approach of thiosilane **2**. In contrast, when acyl fluorides **1g** and **1h** with 2-iodo and 2,6-difluoro groups were used, the expected thioesters **3ga** and **3ha** were obtained in relatively good yields. Pentafluorobenzoyl fluoride **1i** also exhibited robust reactivity in this transformation. The coupling reaction of acyl fluoride **1j** with a 1-naphthyl group also proceeded to afford **3ja** in 70% yield.

**Scheme 3:**
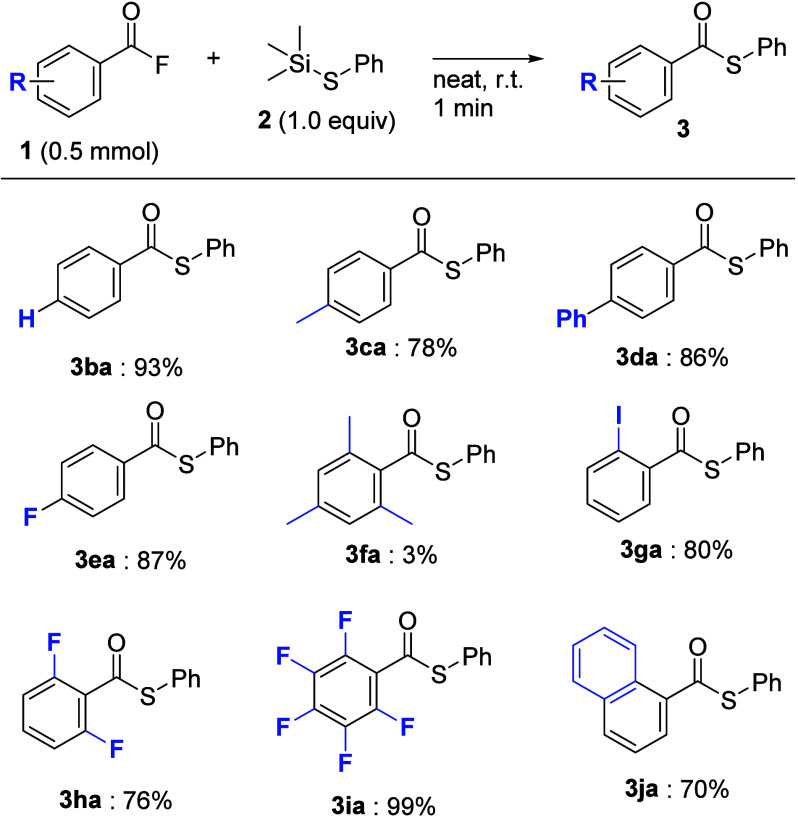
Substrate Scope of Aromatic Acyl Fluorides (without Et_3_N)[Fn sch3-fn1]

a
**1** (0.5 mmol), **2a** (0.5 mmol), rt, 1 min.

Next, thioesterification was applied to various aliphatic acyl fluorides **1k**–**p** (Scheme 4). For example, when heptanoyl fluoride (**1k**) and acyl fluoride **1l** derived from a fatty acid with a 15-carbon chain were used, the desired thioesters **3ka** and **3la** were obtained in 67% and 42% yields, respectively. For **3la**, it seems that there is a steric hindrance between the long carbon chain moiety of **1l** and thiosilane **2a**. Moreover, when substrates **1m** and **1n** with secondary or tertiary alkyl groups, such as cyclohexyl and 1-adamantyl groups, were used under optimal conditions, the desired products thioesters **3ma** and **3na**, respectively, were obtained in good yields. Interestingly, although acyl fluoride **1o** with a 1-phenyl-1-cyclopentyl ring significantly decreased the yield of thioester **3oa**, acyl fluoride **1p** with a 1-phenyl-1-cyclopropyl ring afforded the desired thioester **3pa** in high yield. To clarify the difference in the results between **1o** and **1p**, structural optimization was performed using DFT calculations. Detailed calculation results are provided in the Supporting Information involving the optimal structure of **1o** (Figure S1) and **1p** (Figure S2) by DFT calculations and Cartesian Coordinates of acyl fluoride **1o** (Table S6) and **1p** (Table S7). The results strongly implied that the steric congestion between the cyclopropyl ring and the next carbonyl group facilitates a thiosilane approach. This coupling reaction could be applied to acyl fluoride **1q** with a conjugate alkene moiety to give thioester **3qa** in good yields.

**Scheme 4:**
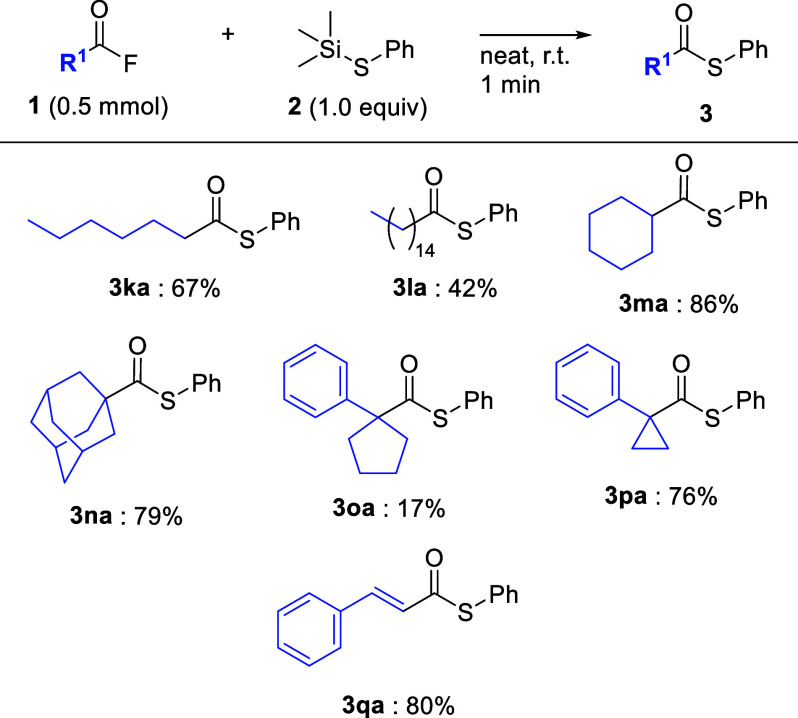
Substrate Scope of Aliphatic Acyl Fluorides (without Et_3_N)[Fn sch4-fn1]

a
**1** (0.5 mmol), **2a** (0.5 mmol), rt, 1 min.

In the above studies, we found that acyl fluorides, having an electron-donating methoxy group or having an alkyl substituent at the 2- or 6-position led to low yields of the corresponding aromatic thioesters **3ea**, **3ga**, and **3ia**. Thus, we re-examined the reaction conditions and found that the addition of a catalytic amount of Et_3_N (5 mol %) drastically improved the product yield (Table S3). Thus, we added a base to the reaction mixture containing acyl fluorides (Scheme 5). For instance, when substrates **1r** and **1s** with 4-methoxy and 3,5-dimethoxy groups, respectively, were reacted in the presence of Et_3_N (5 mol %), the desired reactions proceeded cleanly to give thioesters **3ra** and **3sa**, respectively, in quantitative yields. Similarly, the coupling of acyl fluoride **1f** with a mesityl group with thiosilane **2a** improved the yield of thioester **3fa**. Although when using aliphatic acyl fluoride **1l**, thioester **3la** was obtained in low yield. For **3la**, when the reaction time was extended to 10 min, the yield of **3la** was not improved. In contrast, the coupling reaction of **1o** with a cyclopentyl ring with **2a** produced thioester **3oa** in 98% yield.

**Scheme 5:**
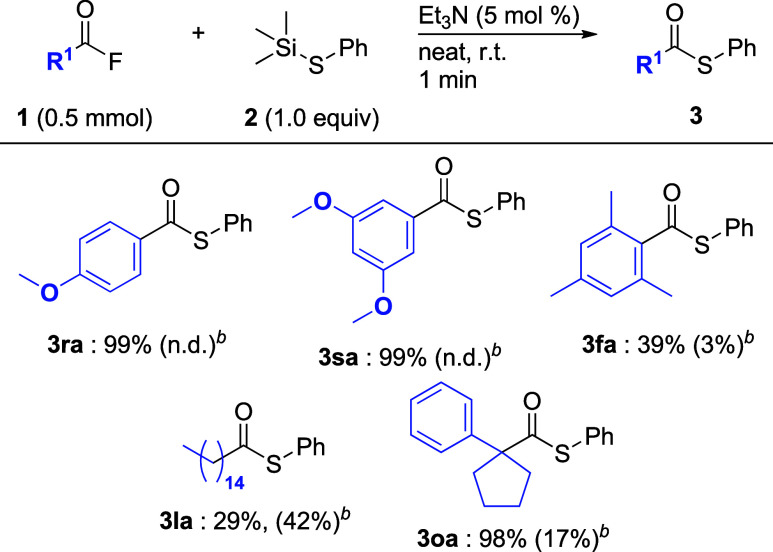
Substrate Scope of Acyl Fluorides and Phenylthiosilanes (with Et_3_N)[Fn sch5-fn1]

a
**1** (0.5 mmol), **2a** (0.5 mmol), and Et_3_N (5 mol %), rt, 1 min.
b
**1** (0.5 mmol), **2a** (0.5 mmol), rt, 1 min.

Next, the substrate scope of the aryl group on the thiosilanes was examined (Scheme 6). When the coupling reactions of **1a** with various aryl thiosilanes **2a–j** that involve substituents on the benzene ring were carried out without a base, the desired aryl thioesters were not obtained in all cases. These results imply that aryl thiosilanes having an electron-donating group, such as a methyl group, involve a stable S–Si bond, the characteristic of which led to a decrease in the reactivity of the coupling between these thiosilanes and acyl fluorides. Therefore, in addition to the results shown in Scheme 5, when a catalytic amount (5 mol %) of Et_3_N was added to each reaction system, as expected, the reactivity of all coupling reactions drastically improved to afford the corresponding thioesters **3ab–3aj** in high yields. Regardless of the electronic effect on the group of benzene rings, when aryl thiosilanes with methyl, methoxy, fluoro, and chloro groups at the *para*-position were used, the desired thioesters **3ab–3ae** were obtained in good to high yields. Similarly, the coupling reaction with thiosilanes at the *meta*-position proceeded smoothly to afford the expected thioesters **3af–3ah** in practical yield. Disubstituted aryl thiosilanes with a 3,5-dimethyl group **2i** or a 2,4-dimethyl group **2j** also gave the desired thioesters **3ai** and **3aj** in 92% and 79% yields, respectively. Consequently, the position of the group on the benzene ring did not influence the nucleophilic attack on acyl fluoride.

**Scheme 6:**
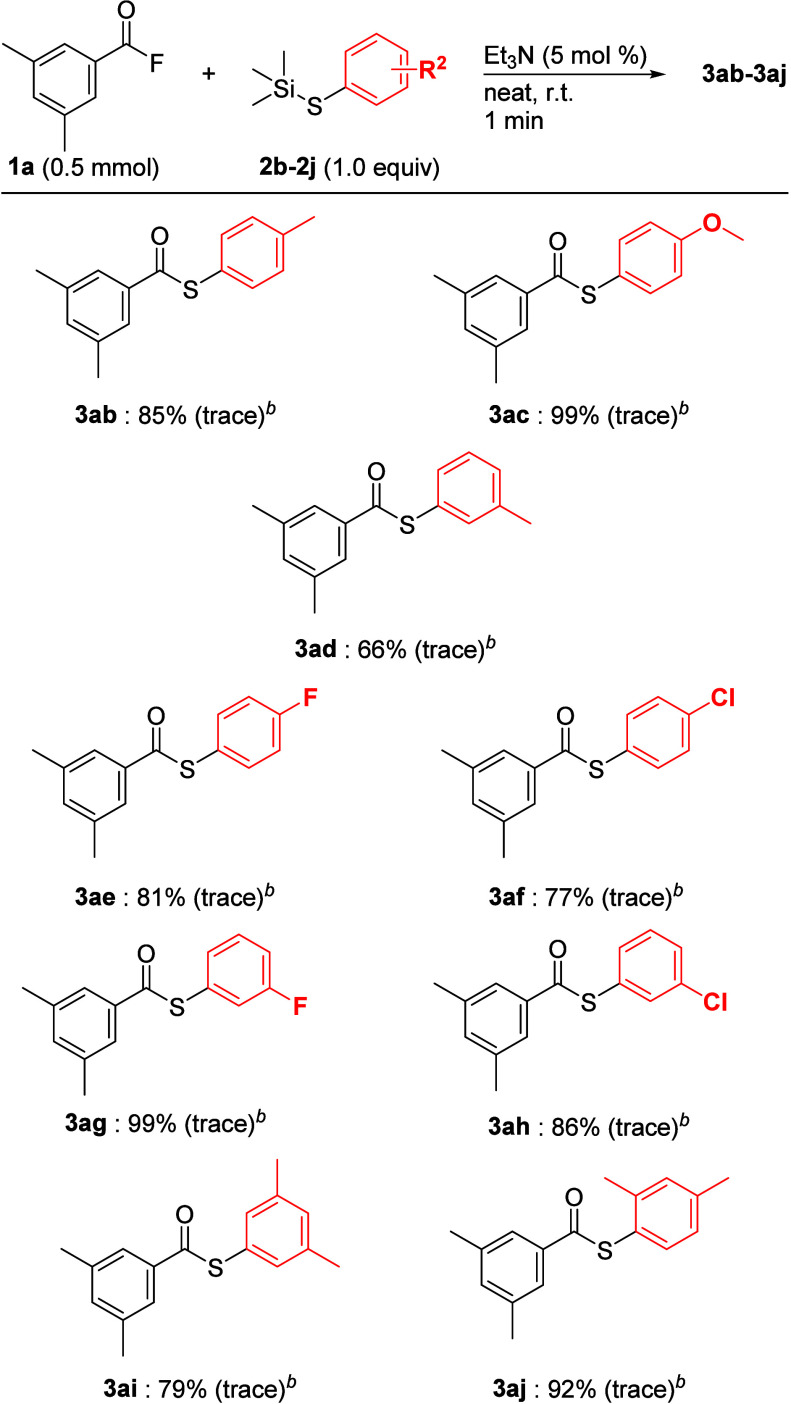
Substrate Scope **1a** and Aryl Thiosilanes (with Et_3_N)[Fn sch6-fn1]

a
**1a** (0.5 mmol), **2** (0.5 mmol), Et_3_N (5 mol %), rt, 1 min.
b
**1a** (0.5 mmol), **2** (0.5 mmol), rt, 1 min.

Moreover, when the coupling of acyl fluoride **1a** with alkyl thiosilane, decyl trimethylsilyl sulfide (**2k**), was examined in the presence and absence of Et_3_N, the desired coupling reactions did not proceed (Table 2, entries 1 and 2). Therefore, we re-examined the optimal conditions for the coupling reaction using alkyl thiosilanes and found that the addition of a stronger base to the reaction mixture in a THF-aqueous solution led to the desired coupling reaction, producing the corresponding thioester derivative (Tables S4 and S5). For instance, when 5 mol % of the strong base KO^*t*^Bu was used under neat conditions, the desired thioester **3ak** was obtained in only 8% GC yield (entry 3). This result strongly implies that an alkoxide anion contributes to the cleavage of the Si–S bond of thiosilane. To further improve the solubility of KO^*t*^Bu, water (0.5 mL) was added to the reaction mixture. Although the addition of water alone was ineffective, the coupling in THF increased the GC yield to 67% (entries 4 and 5). Thus, conducting the reaction in a THF-aqueous solution (H_2_O/THF = 1/49) significantly increased the yield, giving the desired thioester **3ak** in high yield (entry 6). Furthermore, the yield of **3ak** did not decrease, although the amount of the base was reduced to 0.01 equiv per thiosilane **2k** (entry 7). Conditions without a base did not result in the desired coupling reaction (entry 8).

**Table 2:**
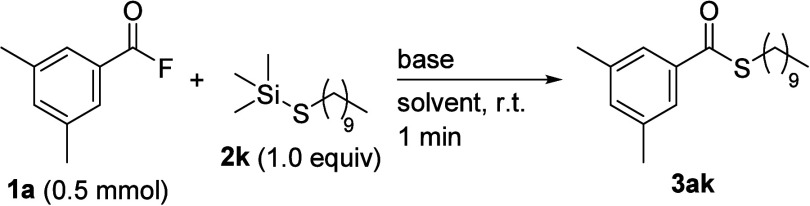
Optimization of the Reaction Conditions for Alkylthiosilane^a^

entry	base	solvent	GC yield of **3ak**
	(mol %)	(mL)	(%)
1			n.d.
2	Et_3_N (5)		n.d.
3	KO*^t^* Bu (5)		8 (trace)
4	KO*^t^* Bu (5)	H_2_O (0.5)	19
5	KO*^t^* Bu (5)	THF (0.5)	67
6	KO*^t^* Bu (5)	H_2_O/THF (1:49) (0.05)	97
7	KO*^t^* Bu (1)	H_2_O/THF (1:49) (0.05)	99 (98)^a^
8		H_2_O/THF (1:49) (0.05)	n.d.

a **1a** (0.5 mmol), **2k** (0.5 mmol), 1 min.

The generality of alkyl thiosilanes was examined under the optimal conditions listed in Table 2 (Scheme 7). When thiosilane **2l** bearing an *n*-butyl group was used, the desired reaction proceeded to give the desired thioester **3al** in 37% yield; however, the reason for the decrease in the yield of **3al** was unclear at this stage. To investigate the steric effect of the alkyl group on the alkyl thiosilanes, thiosilane **2m** with a *t*-butyl group was treated under the optimal conditions. As expected, the yield of **3am** decreased. Interestingly, the coupling reactions of acyl fluoride **1a** with thiosilanes **2n** and **2o** with an ester group and a furfuryl group afforded the corresponding thioesters **3an** and **3ao** in quantitative yields. On the other hand, when the reaction of palmitoyl fluoride (**1l**) with (1-decylthio)­trimethylsilane (**2k**) was examined under the conditions shown in Scheme 7, the desired thioester was not obtained.

**Scheme 7:**
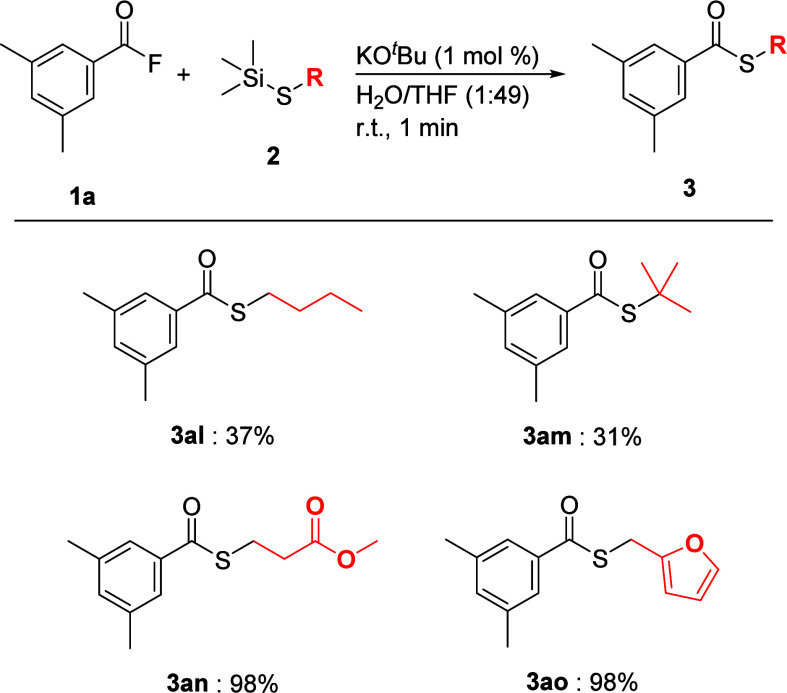
Substrate Scope of Alkyl thiosilanes[Fn sch7-fn1]

a
**1a** (0.5 mmol), **2** (0.5 mmol), KO*^t^* Bu (1 mol %), H_2_O/THF (1:49; 0.05 mL), rt, 1 min.

This reaction could be applied to the gram-scale synthesis of thioester **3aa** (Scheme 8). When acyl fluoride **1a** (5 mmol) was reacted with phenyl thiosilane **2a** (5 mmol) under neat conditions, the corresponding coupling reaction was completed within 5 min to afford thioester **3aa** in 98% yield (1.19 g) after column chromatography.

**Scheme 8:**
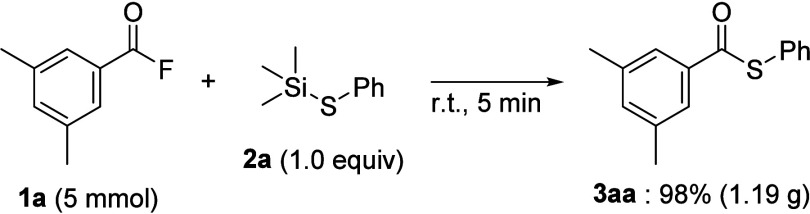
Gram-Scale Synthesis of Thioester **3aa**

Several control experiments were conducted to determine the reaction mechanisms of these couplings (Scheme 9). Initially, the radical scavengers, 2,2,6,6-tetramethylpiperidine 1-oxyl radical (TEMPO) and dibutylhydroxytoluene (BHT), were added to each reaction mixture (Scheme 9A). In all the coupling reactions, since the yields of thioesters **3aa**, **3ad**, and **3ak** did not decrease significantly, it was implied that the radical process was not involved in the coupling reactions. In addition, to demonstrate the steric effect of the silyl group on thiosilane **1a**, coupling reactions of **1a** with phenyl­(triisopropyl)­silane (**2p**) were examined under neat conditions (Scheme 9B). Regardless of the presence or absence of the base, the coupling reaction did not occur. These results showed that the steric hindrance of the silyl group strongly influenced the approach between thiosilane and acyl fluoride. Moreover, when the coupling of acyl chloride **1a**’ with aryl thiosilane **2a** was performed under neat conditions, the expected thioester **3aa** was obtained in low yield (Scheme 9C). Similarly, when running acyl fluoride **1a** or acyl chloride **1a’** with thiol **2a’**, neither coupling produced the desired thioester **3aa** (Scheme 9D). Based on these results, the strong interaction between silicon and fluorine atoms functions as a driving force to promote thioesterification.

**Scheme 9:**
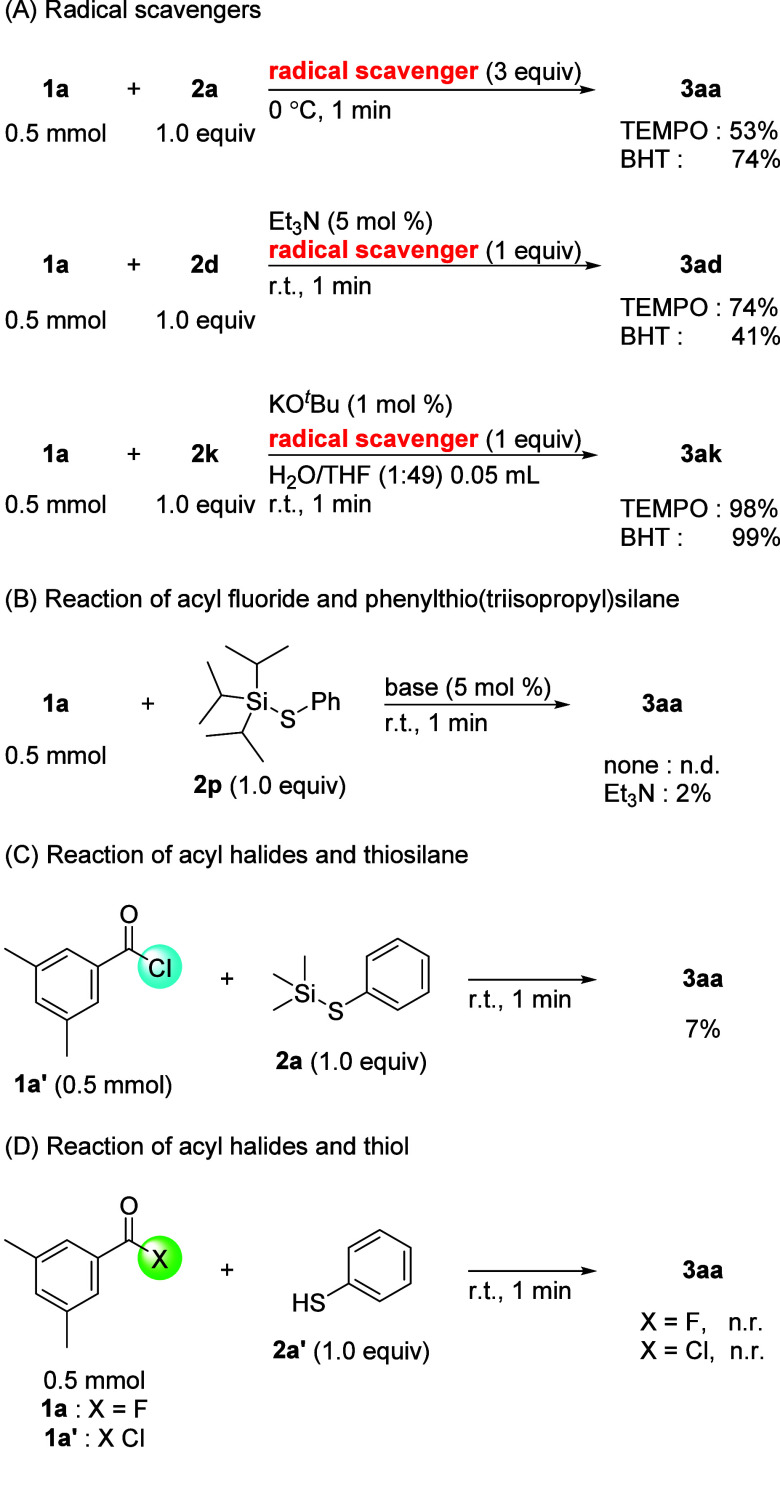
Control Experiments

Based on the results of the control experiments and the related mechanism reported in precedent literature,^18^ a plausible mechanism for the coupling reaction is illustrated in Scheme 10. In the case of direct coupling without a base (Scheme 10A), it is assumed that (i) the nucleophilic sulfur atom of thiosilane **2** adds to the carbonyl carbon of acyl fluoride **1** to generate intermediate **A**, (ii) the silyl group in intermediate **A** shifts to form intermediate **B**, and (iii) the emission of Me_3_SiF occurs from intermediate **B**, facilitated by a strong interaction between the fluorine and a silicon atoms to give thioester **3**. The formation of Me_3_SiF was confirmed by ^1^H-, ^13^C­{^1^H}-, and ^19^F-NMR spectroscopy (Figures S3 and S4 and S5). In the case of the coupling with a base (Scheme 10B), it is assumed that (i) a base (Et_3_N or KO^*t*^Bu) functions as an activator of thiosilane **2** to produce thiolate anion **C**; (ii) in situ generated **C** nucleophilically adds to acyl fluoride **1** to give intermediate **D**; (iii) intermediate **D** liberates a fluoride anion to produce thioester **3**; and (vi) the eliminated fluoride anion is incorporated into this catalytic cycle to activate starting thiosilane **2** again.

**Scheme 10:**
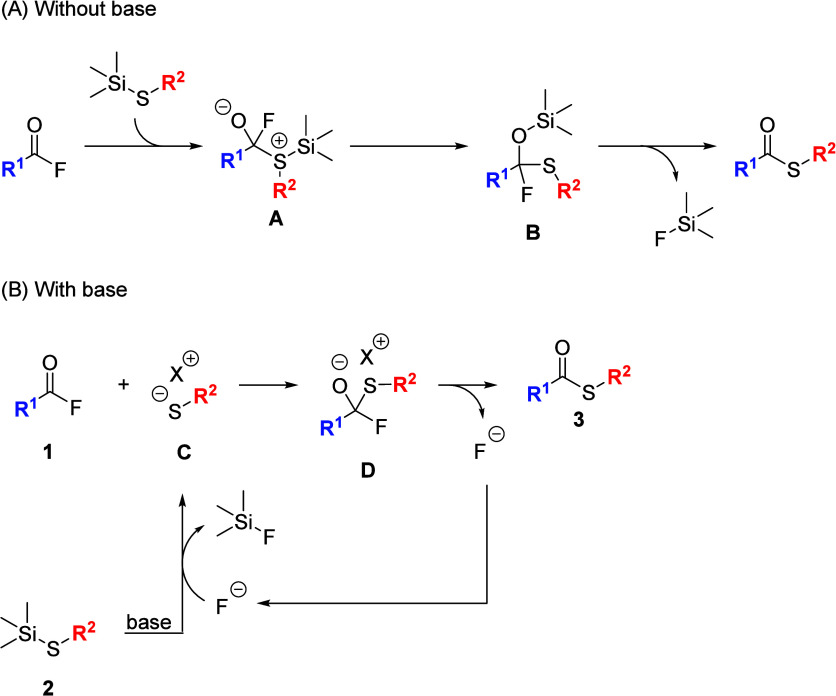
Plausible Reaction Mechanism

## Conclusion

In conclusion, we developed a method for the synthesis of thioesters from aromatic/aliphatic acyl fluorides and a variety of thiosilanes. Remarkably, when phenyl thiosilane was employed, the desired coupling reactions proceeded effectively under neat conditions at room temperature to produce the corresponding thioesters in good to excellent yields, high product purity was obtained by the removal of the sole byproduct, Me_3_SiF. Moreover, the coupling reactions of acyl fluorides with various aryl/alkyl thiosilanes proceeded smoothly with the assistance of a catalytic amount of KO^*t*^Bu in a THF-aqueous solution.

## Experimental Section

### General Information

All reactions were carried out in air, unless otherwise noted. All the heating reactions were performed in an oil bath. Unless otherwise noted, all reagents were obtained from commercial suppliers and used without further purification. Toluene, THF, Et_2_O, and hexane were distilled over sodium benzophenone. CH_3_CN and CH_2_Cl_2_ were dried over CaH_2_ and distilled water. CHCl_3_ was dried over CaCl_2_ and distilled water. Column chromatography was performed using silica and alumina gels. The ^1^H NMR spectra were recorded at 500 and 400 MHz using tetramethylsilane as an internal standard (0.00 ppm). ^13^C­{^1^H} NMR spectra were recorded at 126 and 100 MHz using the central peak of CDCl_3_ (77.0 ppm). Chemical shifts in the ^19^F NMR spectra were reported in ppm relative to the external reference, CF_3_C_6_H_5_ (−62.6 ppm). High-resolution mass spectra (HRMS) were obtained in FAB-positive mode using 3-nitrobenzyl alcohol (NBA) as the matrix. The GC analyses were performed using a DB-5 capillary column (30 m × 0.25 mm with a film thickness 0.25 μm). Acyl fluorides **1** and thiosilanes **2** were prepared by the literature method.^21–24^

### Representative Procedure A for the Synthesis of Acyl Fluoride Derivatives

KF (2.91 g, 50.0 mmol) and CH_3_CN (25 mL) were added to a 100 mL round-bottom flask. Then, 3,5-dimethylbenzoyl chloride (4.22 g, 25.0 mmol) was added to the mixture. The mixture was stirred at 50 °C for 115 h under N_2_. The resulting mixture was filtered, and the solvent was removed under reduced pressure. The crude material was purified by distillation under reduced pressure to yield 3,5-dimethylbenzoyl fluoride **1a** (2.73 g, 72%).

### 3,5-Dimethylbenzoyl Fluoride (**1a**)^25^

Transparent solid (2.73 g, 72%); ^1^H NMR (400 MHz, CDCl_3_) δ 7.64 (s, 2H), 7.31 (s, 1H), 2.37 (s, 6H); ^13^C­{^1^H} NMR (100 MHz, CDCl_3_) δ 157.7 (d, *J*_C–F_ = 343 Hz), 138.8 (d, *J*_C–F_ = 10 Hz), 137.0, 129.0 (d, *J*_C–F_ = 3.0 Hz), 124.6 (d, *J*_C–F_ = 59 Hz); ^19^F NMR (376 MHz, CDCl_3_) δ = 18.4; MS (EI): *m*/*z* 152 (M^+^).

### Representative Procedure B for the Synthesis of Acyl Fluoride Derivatives

A 100 mL round-bottom flask was charged with KHF_2_ (1.56 g, 20.0 mmol) and H_2_O (17 mL). The mixture was then stirred at room temperature for 1 h. Subsequently, tetrabutylammonium chloride (0.2 mmol), 2,6-difluorobenzoyl chloride (1.77 g, 10.0 mmol), and CH_2_Cl_2_ (25 mL) were added to the mixture. The reaction mixture was stirred at room temperature for 2 h. The resulting mixture was washed with CH_2_Cl_2_. The combined organic layers were dried over Na_2_SO_4_ and filtered, and the filtrate was evaporated under reduced pressure. The crude material was purified via distillation under reduced conditions to give 2,6-difluorobenzoyl fluoride **1l** (0.79 g, 25%).

### 2,6-Difluorobenzoyl Fluoride (**1l**)^26^

White solid (0.79 g, 25%); ^1^H NMR (400 MHz, CDCl_3_) δ 7.70–7.63 (m, 1H), 7.10–7.06 (m, 2H); ^13^C­{^1^H} NMR (126 MHz, CDCl_3_) δ 162.4 (dd, *J*_C–F_ = 264.2, 3.8 Hz), 150.7 (d, *J*_C–F_ = 343.4 Hz), 136.5 (dd, *J*_C–F_ = 11.3 Hz), 112.8 (dd, *J*_C–F_ = 21.4, 2.5 Hz); ^19^F NMR (470 MHz, CDCl_3_) δ 47.8 (t, *J*_F–F_ = 23.5 Hz), −104.8 (d, *J*_F–F_ = 47.1 Hz); MS (EI): *m*/*z* 160 (M^+^).

### Representative Procedure C for the Synthesis of Acyl Fluoride Derivatives

To a 100 mL round-bottom flask, SOCl_2_ (2.38 g, 20.0 mmol), CH_2_Cl_2_ (10 mL), 3,5-dimethoxybenzoic acid (1.82 g, 10.0 mmol), and DMF (a few drops) were added. The mixture was stirred overnight at room temperature under N_2_. The resulting mixture was evaporated under reduced pressure to produce acyl chlorides.

KF (1.16 g, 20.0 mmol) and CH_3_CN (10 mL) were added to a 100 mL round-bottom flask. Subsequently, 3,5-dimethoxybenzoyl chloride was added to the crude product. The mixture was stirred for 24 h at 50 °C under N_2_. The resulting mixture was filtered, and the solvent was removed under reduced pressure. The crude material was purified via silica gel column chromatography or distilled under reduced pressure to give 3,5-dimethoxybenzoyl fluoride **1f** (0.96 g, 52%).

### 3,5-Dimethoxybenzoyl Fluoride (**1f**)^25^

White solid (0.96 g, 52%); ^1^H NMR (500 MHz, CDCl_3_) δ 7.17 (d, *J* = 2.5 Hz, 2H), 6.76 (t, *J* = 2.5 Hz, 1H), 3.84 (s, 6H); ^13^C­{^1^H} NMR (126 MHz, CDCl_3_) δ 161.0, 157.3 (d, *J*_C–F_ = 334.7 Hz), 126.5 (d, *J*_C–F_ = 60.4 Hz), 108.8 (d, *J*_C–F_ = 3.8 Hz), 108.0, 55.7; ^19^F NMR (376 MHz, CDCl_3_) δ 18.9; MS (EI): *m*/*z* 184 (M^+^).

### Representative Procedure for the Synthesis of Thiosilane Derivatives

Benzenethiol (11.0 g, 100 mmol), THF (100 mL), and Et_3_N (12.1 g, 120 mmol) were added to a 100 mL round-bottom flask. Trimethylsilyl chloride (12.0 g, 110 mmol) was added to the mixture. Subsequently, the mixture was stirred at room temperature for 24 h under N_2_. The mixture was evaporated under reduced pressure and filtered, and the solvent was removed under reduced pressure. The crude material was purified via distillation under reduced pressure to yield trimethyl­(phenylthio)­silane **2a** (15 g, 82%).

### Trimethyl­(phenylthio)­silane (**2a**)^27^

Colorless oil (15 g, 82%); ^1^H NMR (400 MHz, CDCl_3_) δ 7.42–7.40 (m, 2H), 7.25–7.23 (m, 3H), 0.27 (s,9 H); ^13^C­{^1^H} NMR (100 MHz, CDCl_3_) δ 135.1, 131.4, 128.7, 126.8, 0.81; MS (EI): *m*/*z* 182 (M^+^).

### Representative Procedure for the Synthesis of Thioester Derivatives

3,5-Dimethylbenzoyl fluoride (76.1 mg, 0.500 mmol), and trimethyl­(phenylthio)­silane (91.2 mg, 0.500 mmol) were added to a 10 mL round-bottom flask. The mixture was then stirred at room temperature for 1 min. After the reaction, the crude material was purified by silica gel column chromatography (hexane to hexane/EtOAc = 99:1) to yield *S*-phenyl 3,5-dimethylbenzothioate **3aa** (116.1 mg, 99%).

### *S*-Phenyl 3,5-Dimethylbenzothioate (**3aa**)^28^

White solid (116.1 mg, 99%); ^1^H NMR (400 MHz, CDCl_3_) δ 7.63 (s, 2H), 7.52–7.49 (m, 2H), 7.46–7.43 (m, 3H), 7.23 (s, 1H), 2.38 (s, 6H); ^13^C­{^1^H} NMR (100 MHz, CDCl_3_) δ 190.3, 138.4, 136.6, 135.3, 135.0, 129.4, 129.2, 127.6, 125.1, 21.2; MS (FAB-Magnetic sector): *m*/*z* calcd for C_15_H_15_OS (M^+^ + H): 243.08; found: 243.08.

### Representative Procedure for the Synthesis of Thioester Derivatives

To a 10 mL round-bottom flask was charged with 3,5-dimethylbenzoyl fluoride (76.1 mg, 0.500 mmol), Et_3_N (2.5 mg, 0.025 mmol), and trimethyl­(4-methylphenylthio)­silane (98.2 mg, 0.500 mmol) were added. The mixture was then stirred at room temperature for 1 min. After the reaction, the crude material was purified by silica gel column chromatography (hexane to hexane/EtOAc = 99:1) to yield *S*-(4-methylphenyl) 3,5-dimethylbenzothioate **3ab** (112.5 mg, 85%).

### *S*-(4-Methylphenyl) 3,5-Dimethylbenzothioate (**3ab**)^29^

White solid (112.5 mg, 85%); ^1^H NMR (400 MHz, CDCl_3_) δ 7.62 (s, 2H), 7.39–7.36 (m, 2H), 7.26–7.24 (m, 2H), 7.21 (s, 1H), 2.39 (s, 3H), 2.37 (s, 6H); ^13^C­{^1^H} NMR (100 MHz, CDCl_3_) δ 190.7, 139.6, 138.4, 136.7, 135.2, 135.0, 130.0, 125.1, 124.0, 21.3, 21.2; MS (FAB-Magnetic Sector): *m*/*z* calcd for C_16_H_17_OS (M^+^ + H): 257.10; found: 257.10.

### Representative Procedure for the Synthesis of Thioester Derivatives

To a 10 mL round-bottom flask, 3,5-dimethylbenzoyl fluoride (76.1 mg, 0.500 mmol), KO^t^Bu (0.56 mg, 0.0050 mmol), THF (49 μL), H_2_O (1 μL), and (1-decylthio)­trimethylsilane (123.3 mg, 0.5000 mmol) were added. The mixture was then stirred at room temperature for 1 min. The reaction mixture was then evaporated under reduced pressure. After the reaction, the crude material was purified using silica gel column chromatography (hexane/EtOAc = 99:1) to give *S*-(1-decyl) 3,5-dimethylbenzothioate **3ak** (145.6 mg, 98%).

### *S*-(1-Decyl) 3,5-Dimethylbenzothioate (**3ak**)

Colorless oil (145.6 mg, 98%); ^1^H NMR (400 MHz, CDCl_3_) δ 7.57 (s, 2 H), 7.17 (s, 1H), 3.04 (t, *J* = 7.2 Hz, 2H), 2.35 (s, 6H), 1.69–1.61 (m, 2H), 1.45–1.23 (m, 14H), 0.88 (t, *J* = 7.2 Hz, 3 H); ^13^C­{^1^H} NMR (100 MHz, CDCl_3_) δ 192.4, 138.2, 137.3, 134.8, 124.9, 31.9, 29.6, 29.53, 29.49, 29.3, 29.2, 29.0, 28.9, 22.7, 21.2, 14.1; HRMS (FAB): *m*/*z* calcd for C_19_H_31_OS (M^+^ + H): 307.2096; found: 307.2095.

## Supplementary Material



## Data Availability

The data underlying this study are available in the published article and its online Supporting Information.

## Notes

General experimental information, synthesis methods of starting materials, and NMR spectra of the prepared products (PDF)
