# Supplementary material for: Direct Synthesis of Various Thioesters from Acyl Fluorides and Thiosilanes through the Assistance of a Si–F Bond Formation
Source: J Org Chem. 2025 Oct 2;90(41):14664–72. doi: 10.1021/acs.joc.5c01677 (PMC12538585; doi:10.1021/acs.joc.5c01677)
Supplement: Supplementary file 1 [file jo5c01677_si_001.pdf]

Supporting Information

Direct Synthesis of Various Thioesters from Acyl Fluorides and Thiosilanes through the Assistance of a Si-F Bond Formation

Ryuki Takeuchi, Kento Ishida, and Norio Sakai*

Department of Pure and Applied Chemistry, Faculty of Science and Technology, Tokyo University of Science, Noda, Chiba 278-8510, Japan

Table of Contents

1. General Information ··· S2
2. Preparation of Acyl Fluorides ··· S2
 Method A ··· S2
 Method B ··· S2
 Method C ··· S3
3. Preparation of Thiosilanes ··· S3
4. Optimization of Reaction Conditions ··· S3
 4.1. Screening of Base-Catalysts ··· S3
 4.2. Screening of Other Reaction Conditions ··· S4
 4.3. Optimization of Reaction Conditions with Aryl Thiosilanes ··· S5
 4.4. Screening of Base-Catalysts with an Alkyl Thiosilane ··· S6
 4.5. Screening of Other Reaction Conditions ··· S7
5. Substrate Scope of Acyl Fluoride Derivatives ··· S8
6. Substrate Scope of Aryl Thiosilane Derivatives ··· S8
7. Substrate Scope of Alkyl Thiosilane Derivatives ··· S9
8. Gram-Scale Synthesis of **3aa** ··· S9
9. Control Experiment ··· S9
 9.1. Radical Scavengers Experiment using **1a** and **2a** ··· S9
 9.2. Radical Scavengers Experiment using **1a** and **2d** ··· S10
 9.3. Radical Scavengers Experiment using **1a** and **2k** ··· S10
 9.4. Substrate Scope of Triisopropyl(phenylthio)silane (**2p**) ··· S10
 9.5. Reaction of Acyl Chloride and Thiosilane **2a** ··· S11
 9.6. Reaction of Acyl Halides and Thiol **2a’** ··· S11
 9.7. Detection of Trimethylsilyl Fluoride by NMR ··· S12
10. Spectra Data ··· S13
11. Optimal Structure of **1o** and **1p** by DFT calculations ··· S36
12. Cartesian Coordinates of Acyl Fluoride **1o** and **1p** ··· S37
13. References ··· S40
14. NMR Charts ··· S43-S125

1. General Information

All reactions were carried out under an ambient atmosphere, unless otherwise noted. Toluene, THF, Et_2O, and hexane were distilled over Na-benzophenone prior to the use. CH_3CN and CH_2Cl_2 were dried over CaH_2 and then distilled. $CHCl_3$ was dried over $CaCl_2$ and was distilled. Other regents were used without further purification, unless otherwise noted. Reactions were monitored by TLC analysis of reaction aliquots. Column chromatography was performed using silica and alumina gel. The ^1H NMR spectra were recorded at 500 MHz or 400 MHz using tetramethylsilane as an internal standard (0.00 ppm). The $^{13}C\{^1H\}$ NMR spectra were recorded at 126 MHz or 100 MHz using the center peak of $CDCl_3$ (77.0 ppm). Unless otherwise stated, spectra were recorded with the proton-decoupled mode. Chemical shifts in the ^{19}F NMR spectra are reported in ppm relative to the external reference, $CF_3C_6H_5$ (δ -62.6) High-resolution mass spectra (HRMS) were obtained under FAB-positive mode using NBA (3-nitrobenzyl alcohol) as a matrix. GC analyses were performed using a DB-5 capillary column (30 m × 0.25 mm; film thickness 0.25 μm).

2. Preparation of Acyl Fluorides

Method A[1]

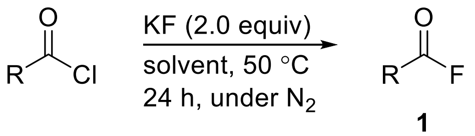

To a 100-mL round-bottom flask, KF (2 equiv) and CH_3CN or THF (1 M) were added. An acyl chloride (1 equiv) was added to the mixture solution. The mixture was stirred at 50 ˚C for 24 h under N_2. The resulting mixture was filtered, and the solvent removed under reduced pressure. The crude material was purified via silica gel column chromatography or distillation under reduced pressure to give the corresponding acyl fluorides **1**.

Method B[2]

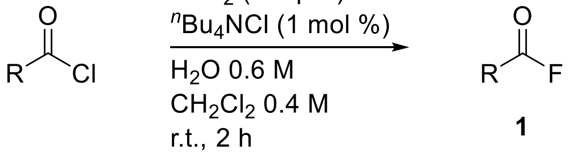

To a 100-mL round-bottom flask, KHF_2 (2 equiv) and H_2O (0.6 M) were added. The mixture solution was stirred at room temperature for 1 h. Tetrabutylammonium chloride (1 mol %), acyl chloride (1 equiv), and CH_2Cl_2 (0.4 M) were added to the mixture solution. Following the addition, the reaction was stirred at room temperature for 2 h. The resulting mixture was washed with CH_2Cl_2. The combined organic layer was dried over Na_2SO_4 and filtered, and then the filtrate was evaporated under the reduced pressure. The crude material was purified via silica gel column chromatography or

distillation under the reduced pressure to give the corresponding acyl fluorides **1**.

Method C[3]

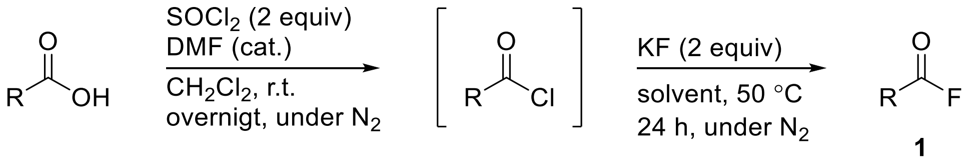

To a 100-mL round-bottom flask, $SOCl_2$ (2 equiv), CH_2Cl_2 (1 M), a carboxylic acid (1 equiv), and DMF (few drops) were successively added. The mixture was stirred at room temperature overnight under N_2. The resulting mixture was evaporated under reduced pressure to give the acyl chlorides.

A 100 mL round-bottom flask was charged with KF (2 equiv) and CH_3CN or THF (1 M). After an acyl chloride (1 equiv) was added. The mixture was stirred for 24 h at 50 ˚C under N_2. The resulting mixture was filtered, and the solvent was removed under the reduced pressure. The crude material was purified via silica gel column chromatography or distillation under reduced pressure to give the corresponding acyl fluorides **1**.

3. Preparation of Thiosilanes[4]

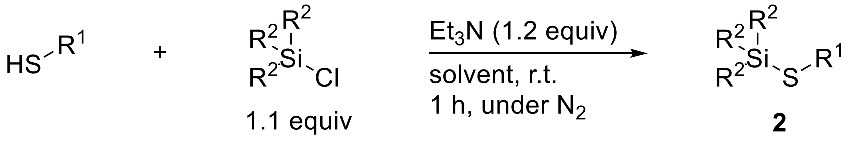

To a 100-mL round-bottom flask, a thiol (1 equiv), Et_2O or THF (1 M), and Et_3N (1.2 equiv) were added. The mixture solution was added trimethylsilyl chloride or triisopropylsilyl chloride (1.1 equiv). Following the addition, the mixture was stirred at room temperature for 1 h under N_2. The mixture was evaporated under reduced pressure, filtered, and the solvent was removed again under reduced pressure. The crude material was purified via distillation under reduced pressure to give the corresponding thiosilanes **2**.

4. Optimization of Reaction Conditions

4.1. Screening of Base-Catalysts

To a 10-mL round-bottom flask, 3,5-dimethylbenzoyl fluoride (76.1 mg, 0.500 mmol), a base (0.0250 mmol), toluene (0.5 mL), and trimethyl(phenylthio)silane (91.2 mg, 0.500 mmol) were added. The mixture was stirred at 80 °C for 1 h. After the reaction, H_2O (1 mL) and dodecane (20 mg, 0.140 mmol) were added to the reaction mixture, which was then extracted with EtOAc (10 mL). The combined organic layer was analyzed by GC and LRMS.

Table S1. Screening of base-catalysts

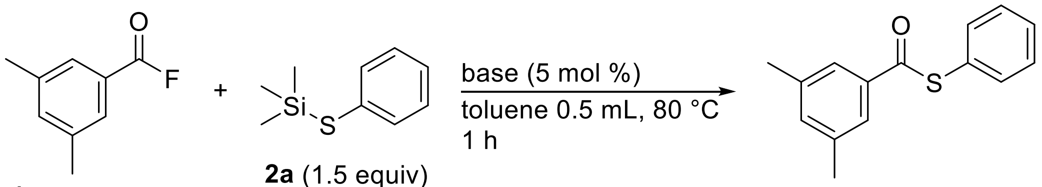

entry	base	conv. of **1a**	yield of **3aa**
		(%)	(%)
1	----	42	2
2	Li_2CO_3	72	38
3	Na_2CO_3	45	9
4	K_2CO_3	100	67
5	Cs_2CO_3	87	59
6[a]	Cs_2CO_3	100	84
7	Ag_2CO_3	53	16
8	$CaCO_3$	44	4
9	Ba_2CO_3	76	41
10	$(NH_4)_2CO_3$	60	20
11	KF	100	89
12[a]	CsF	100	77
13	KF/18-crown-6	100	84
14	KSAc	100	90
15	KEX	100	97
16	Et_3N	100	86

[a] Reaction time was 2 h.

4.2. Screening of other reaction conditions

To a 10-mL round-bottom flask, 3,5-dimethylbenzoyl fluoride (**1a**: 76.1 mg, 0.500 mmol), potassium ethylxanthate (0.0250 mmol), a solvent (0.5 mL), and trimethyl(phenylthio)silane (**2a**) were added. The mixture was stirred. After the reaction, H_2O (1 mL) and dodecane (20 mg, 0.140 mmol) were added to the reaction mixture, which was then extracted with EtOAc (10 mL). The combined organic layer was analyzed by GC and LRMS.

Table S2. Screening of reaction conditions

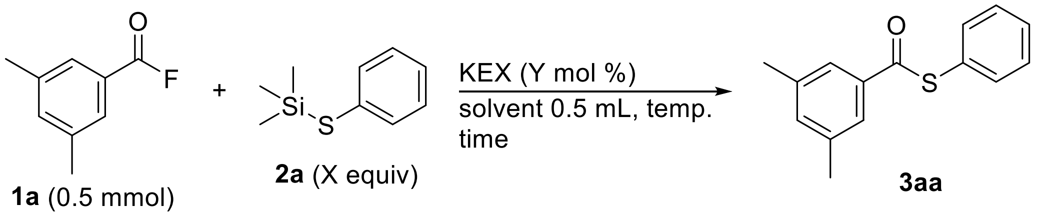

entry	X	Y	solvent	temp.	time	conv. of **1a**	yield of **3aa**
	(equiv)	(mol %)		(˚C)	(min)	(%)	(%)
1	1.5	5	toluene	80	60	100	97
2	1.5	5	toluene	80	60	100	91
3	1.3	5	toluene	80	60	100	88
4	1.1	5	toluene	80	60	100	91
5	1.0	5	toluene	80	60	100	83
6	1.1	5	toluene	60	60	100	90
7	1.1	5	toluene	40	60	95	70
8	1.1	5	toluene	r.t.	60	90	64
9	1.1	5	hexane	60	60	97	89
10	1.1	5	THF	60	60	100	90
11	1.1	5	CH_3CN	60	60	100	89
12	1.1	5	----	60	60	100	93
13	1.1	5	----	r.t.	60	100	92
14	1.1	3	----	r.t.	60	100	92
15	1.1	1	----	r.t.	60	100	90
16	1.1	----	----	r.t.	60	100	91
17	1.1	----	----	r.t.	1	100	98 (98)[a]
18	1.0	----	----	r.t.	1	100	95 (95)[a]

[a] Isolated yield.

4.3. Optimization of reaction conditions with aryl thiosilanes

To a 10-mL round-bottom flask, 3,5-dimethylbenzoyl fluoride (**1a**: 76.1 mg, 0.500 mmol), a base (0.0250 mmol), and trimethyl(arylthio)silane (**2**: 0.500 mmol) were successively added. The mixture was at room temperature for 1 min. After the reaction, H_2O (1 mL) and dodecane (20 mg, 0.140 mmol) were added to the reaction mixture, which was then extracted with EtOAc (10 mL). The combined organic layer was isolated by silica gel column chromatography.

Table S3. Screening of reaction conditions

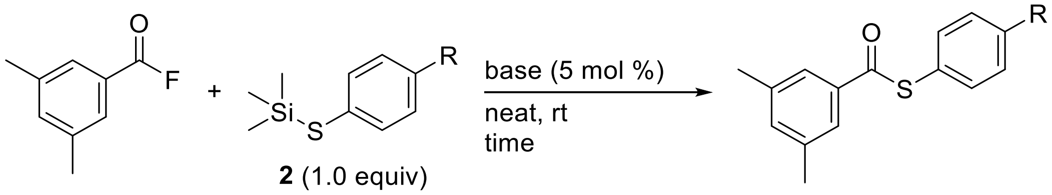

1a (0.5 mmol) **2** (1.0 equiv) **3**

entry	R	base	time	Isolated yield of **3**
			(min)	(%)
1	CH_3	----	1	0
2	Cl	----	1	0
3	CH_3	----	10	0
4	Cl	----	10	0
5	CH_3	KF	1	87
6	Cl	KF	1	69
7	F	KF	1	82
8	CH_3	Et_3N	1	85
9	Cl	Et_3N	1	77
10	F	Et_3N	1	81

4.4. Screening of Base-Catalysts with Alkyl Thiosilane

A 10-mL round-bottom flask was charged with 3,5-dimethylbenzoyl fluoride (**1a**: 76.1 mg, 0.500 mmol), a base (0.0250 mmol), and (1-decylthio)trimethylsilane (**2k**: 0.500 mmol). The mixture was stirred at room temperature to 70 ˚C for 1 min. After the reaction, H_2O (1 mL) and dodecane (20 mg, 0.140 mmol) were added to the reaction mixture, which was then extracted with EtOAc (10 mL). The combined organic layer was analyzed by GC and LRMS.

Table S4. Screening of base-catalysts

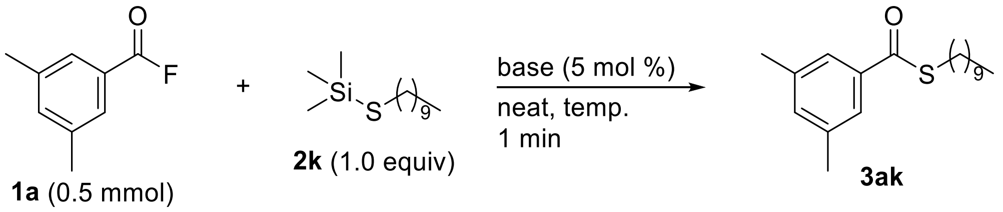

1a (0.5 mmol) **2k** (1.0 equiv) **3ak**

entry	base	temp.	conv. of **1a**	yield of **3ak**
		(˚C)	(%)	(%)
1	----	r.t.	31	n.d.
2	----	50	37	n.d.
3	----	60	35	n.d.

4	----	70	34	n.d.
5	Et_3N	r.t.	49	n.d.
6	Et_3N	50	43	n.d.
7	Et_3N	60	37	n.d.
8	Et_3N	70	32	n.d.
9	KO^{t}Bu	r.t.	49	8 (trace)[a]
10	KO^{t}Bu	50	75	60
11	KO^{t}Bu	60	62	36
12	KO^{t}Bu	70	76	40
13	NaOH	r.t.	48	3
14	NaOH	60	62	13
15	NaOH	70	68	24

[a] Isolated yield.

4.5. Screening of Other Reaction Conditions

To a 10-mL round-bottom flask, 3,5-dimethylbenzoyl fluoride (**1a**: 76.1 mg, 0.500 mmol), a base, solvent, and (1-decylthio)trimethylsilane (**2k**: 0.500 mmol) were successively added. The mixture was stirred at room temperature for 1 min. After the reaction, H_2O (1 mL) and dodecane (20 mg, 0.140 mmol) were added to the reaction mixture, which was then extracted with EtOAc (10 mL). The combined organic layer was analyzed by GC and LRMS.

Table S5. Screening of reaction conditions

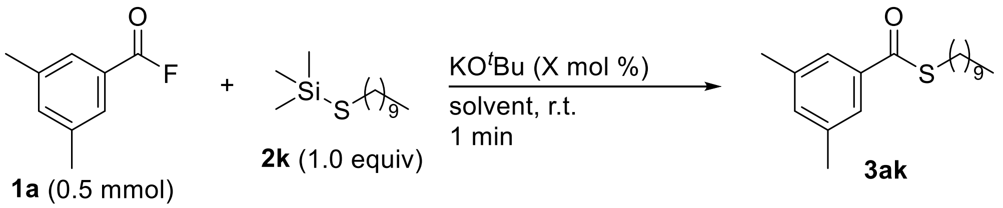

entry	X	solvent	conv. of **1a**	yield of **3ak**
	(mol %)	(mL)	(%)	(%)
1	5	THF (0.5)	89	67
2	5	Et_2O (0.5)	52	n.d.
3	5	CH_2Cl_2 (0.5)	67	19
4	5	$CHCl_3$ (0.5)	51	9
5	5	hexane (0.5)	56	12
6	5	H_2O (0.5)	69	19
7	5	H_2O/THF (1:4) (0.5)	86	59
8	5	H_2O/THF (1:9) (0.5)	87	59
9	5	H_2O/THF (1:19) (0.5)	97	93

10	5	H_2O/THF (1:49) (0.5)	100	99
11	5	H_2O/THF (1:99) (0.5)	73	31
12	5	H_2O/THF (1:199) (0.5)	72	27
13	5	H_2O/T100HF (1:499) (0.5)	88	56
14	5	H_2O/THF (1:49) (0.4)	100	96
15	5	H_2O/THF (1:49) (0.3)	100	96
16	5	H_2O/THF (1:49) (0.2)	100	98
17	5	H_2O/THF (1:49) (0.1)	100	98
18	5	H_2O/THF (1:49) (0.05)	100	97
19	3	H_2O/THF (1:49) (0.05)	100	98
20	1	H_2O/THF (1:49) (0.05)	100	99 (98)[a]
21	----	H_2O/THF (1:49) (0.05)	38	n.d.

[a] Isolated yield.

5. Substrate Scope of Acyl Fluoride Derivatives

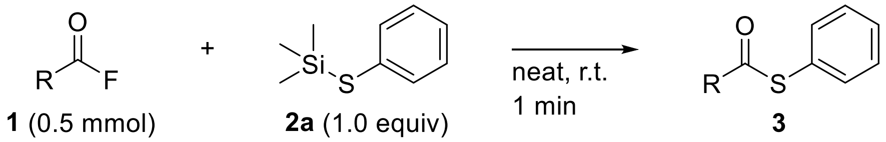

To a 10 mL round-bottom flask, an acyl fluoride (**1**: 0.500 mmol) and trimethyl(phenylthio)silane (**2a**: 91.2 mg, 0.500 mmol) were added. The mixture was stirred at room temperature for 1 min. After the reaction, the crude material was purified via silica gel column chromatography (hexane to hexane/EtOAc = 99:1) to give the corresponding thioesters **3**.

6. Substrate Scope of Aryl Thiosilane Derivatives

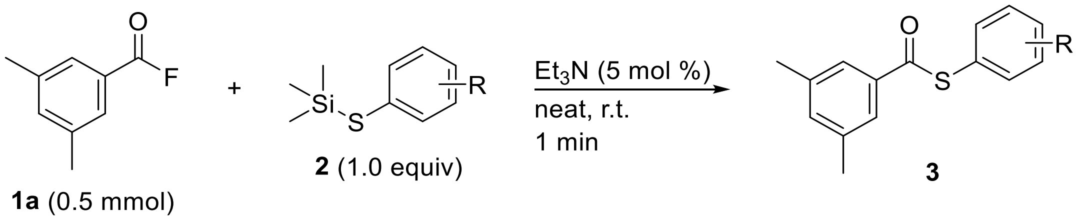

To a 10-mL round-bottom flask, 3,5-dimethylbenzoyl fluoride (**1a**: 76.1 mg, 0.500 mmol), Et_3N (2.5 mg, 5 mol %), and trimethyl(arylthio)silane (**2**: 0.5 mmol) were added. The mixture was stirred at room temperature for 1 min. After the reaction, the crude material was purified via silica gel column chromatography (hexane to hexane/EtOAc = 99:1) to give the corresponding thioester derivative **3**.

7. Substrate Scope of Alkyl Thiosilane Derivatives

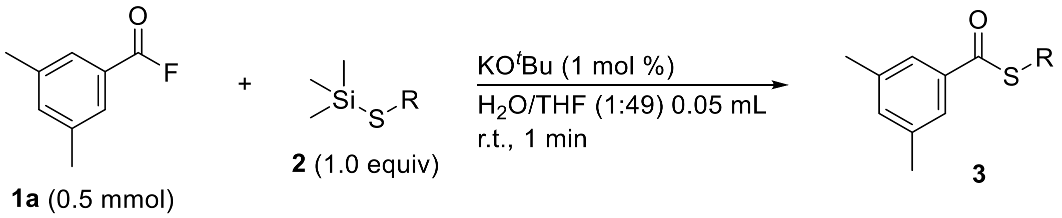

To a 10-mL round-bottom flask, 3,5-dimethylbenzoyl fluoride (**1a**: 76.1 mg, 0.500 mmol), KO^{t}Bu (0.56 mg, 1 mol %), THF (49 μL), H_2O (1 μL), and trimethyl(alkylthio)silane (**2**: 0.500 mmol) were added. The mixture was stirred at room temperature for 1 min. The reaction mixture was evaporated under reduced pressure. After the reaction, the crude material was purified via silica gel column chromatography (hexane/EtOAc = 99:1) to give the corresponding thioester derivative **3**.

8. Gram-Scale Synthesis of 3aa

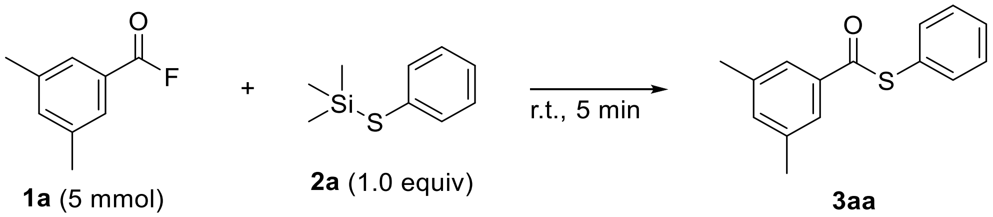

A 10-mL round-bottom flask was charged with 3,5-dimethylbenzoyl fluoride (**1a**: 760.1 mg, 5.000 mmol) and trimethyl(phenylthio)silane (**2a**: 911.8 mg, 5.000 mmol). The mixture was stirred at room temperature for 1 min. The reaction mixture was evaporated under reduced pressure. After the reaction, the crude material was purified via silica gel column chromatography (hexane/EtOAc = 99:1) to afford **3aa** as a white solid (1.19 g, 98%).

9. Control Experiment

9.1. Radical Scavengers Experiment using 1a and 2a

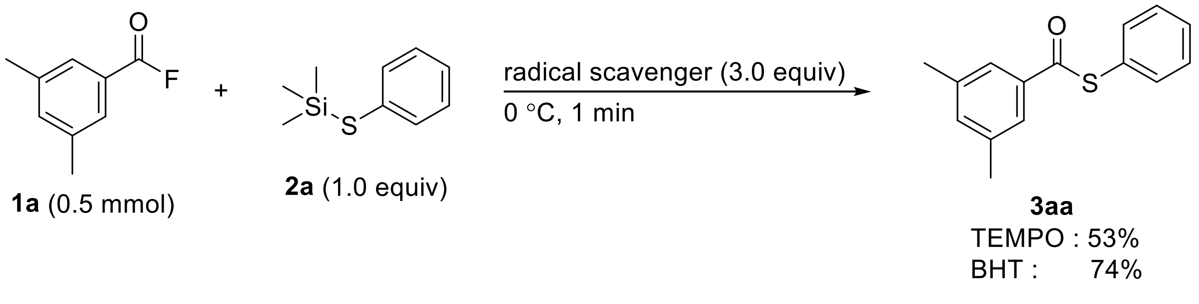

To a 10-mL round-bottom flask, 3,5-dimethylbenzoyl fluoride (**1a**: 76.1 mg, 0.500 mmol), TEMPO or BHT (1.5 mmol), and trimethyl(phenylthio)silane (**2a**: 91.2 mg, 0.500 mmol) were added. The mixture was stirred at 0 °C for 1 min. After the reaction, H_2O (1 mL) and dodecane (20 mg, 0.14 mmol) were added to the reaction mixture, which was then extracted with EtOAc (10 mL). The combined organic layer was analyzed by GC and LRMS.

9.2. Radical Scavengers Experiment using 1a and 2d

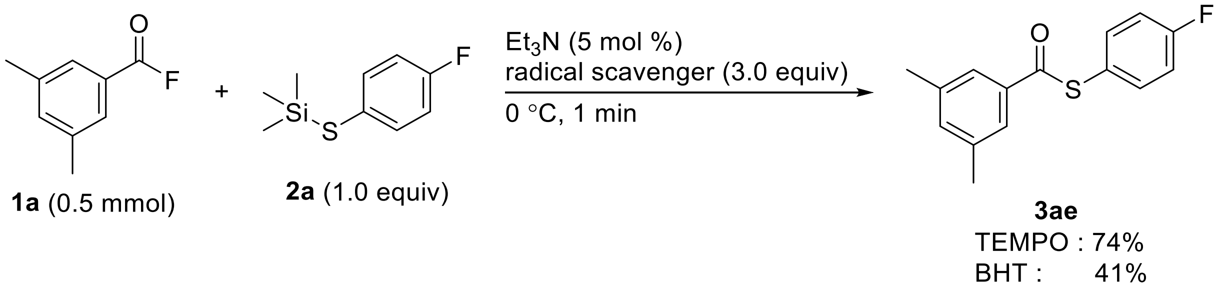

To a 10-mL round-bottom flask, 3,5-dimethylbenzoyl fluoride (**1a**: 76.1 mg, 0.500 mmol), Et_3N (2.5 mg, 5 mol %), TEMPO or BHT (1.5 mmol), and trimethyl(4-fluorophenylthio)silane (**2e**: 100.2 mg, 0.500 mmol) were added. The mixture was stirred at room temperature for 1 min. After the reaction, H_2O (1 mL) and dodecane (20 mg, 0.14 mmol) were added to the reaction mixture, which was then extracted with EtOAc (10 mL). The combined organic layer was analyzed by GC and LRMS.

9.3. Radical Scavengers Experiment using 1a and 2k

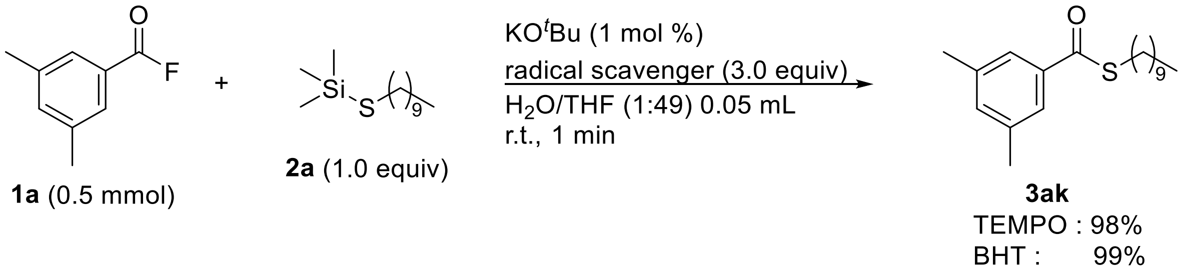

To a 10-mL round-bottom flask, 3,5-dimethylbenzoyl fluoride (**1a**: 76.1 mg, 0.500mmol), KO^{t}Bu (0.56 mg, 1 mol %), TEMPO or BHT (1.5 mmol), H_2O (1 μL), THF (49 μL), and (1-decylthio)trimethylsilane (**2k**: 123.3 mg, 0.5000 mmol) were added. The mixture was stirred at room temperature for 1 min. After the reaction, H_2O (1 mL) and dodecane (20 mg, 0.14 mmol) were added to the reaction mixture, which was then extracted with EtOAc (10 mL). The combined organic layer was analyzed by GC and LRMS.

9.4. Substrate Scope of Triisoprpyl(phenylthio)silane (2p)

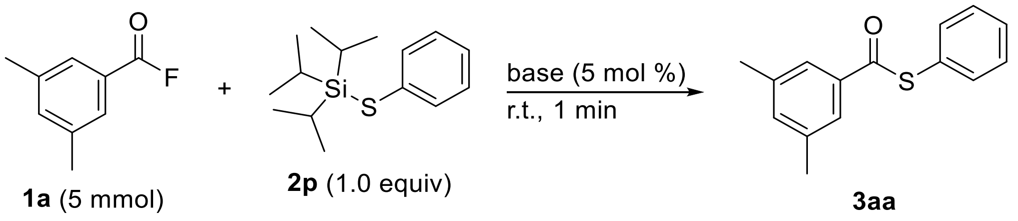

To a 10-mL round-bottom flask, 3,5-dimethylbenzoyl fluoride (**1a**: 76.1 mg, 0.500 mmol) and triisopropyl(phenylthio)silane (**2p**: 133.3 mg, 0.5000 mmol) were added. The mixture was stirred at room temperature for 1 min. After the reaction, the crude material was purified via silica gel column chromatography (hexane to hexane/EtOAc = 99:1).

To a 10-mL round-bottom flask, 3,5-dimethylbenzoyl fluoride (**1a**: 76.1 mg, 0.500 mmol), Et_3N (2.5 mg, 5 mol %), and triisopropyl(phenylthio)silane (**2p**: 133.3 mg, 0.5000 mmol) were added. The mixture was stirred at room temperature for 1 min. After the reaction, the crude material was purified via silica gel column chromatography (hexane to hexane/EtOAc =99:1) to afford **3aa** (2%).

9.5. Reaction of Acyl Chloride and Thiosilane 2a

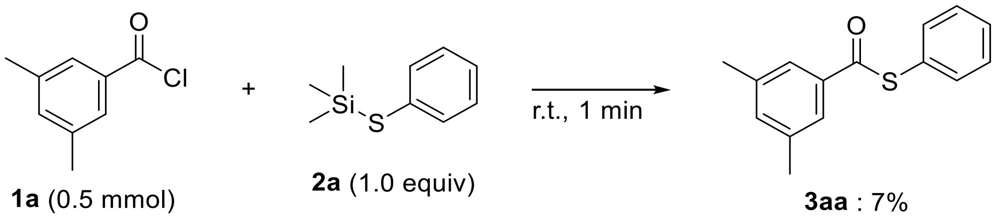

1a (0.5 mmol) **2a** (1.0 equiv) **3aa** : 7%

To a 10-mL round-bottom flask, 3,5-dimethylbenzoyl chloride (**1a**: 84.3 mg, 0.500 mmol) and trimethyl(phenylthio)silane (**2a**: 91.2 mg, 0.500 mmol) were added. The mixture was stirred at room temperature for 1 min. After the reaction, H_2O (1 mL) and dodecane (20 mg, 0.14 mmol) were added to the reaction mixture, which was then extracted with EtOAc (10 mL). The combined organic layer was analyzed by GC and LRMS.

9.6. Reaction of Acyl Halides and Thiol 2a'

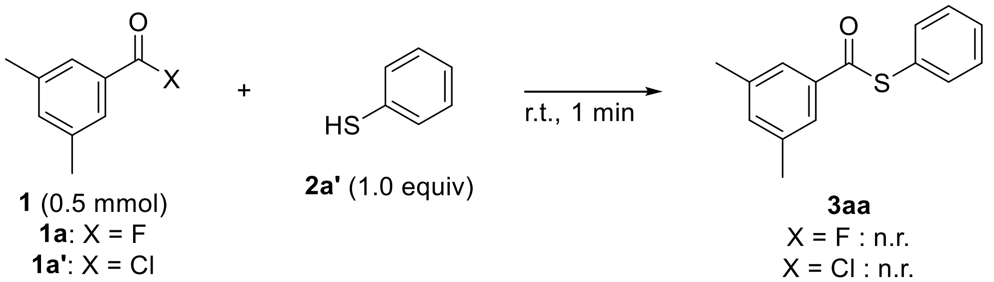

1 (0.5 mmol)
1a: X = F
1a': X = Cl

2a' (1.0 equiv)

3aa
X = F : n.r.
X = Cl : n.r.

To a 10-mL round-bottom flask, 3,5-dimethylbenzoyl fluoride (**1a**: 76.1 mg, 0.500 mmol) and benzenethiol (**2a'**: 55.1 mg, 0.500 mmol) were added. The mixture was stirred at room temperature for 1 min. After the reaction, H_2O (1 mL), and dodecane (20 mg, 0.14 mmol) were added to the reaction mixture, which was then extracted with EtOAc (10 mL). The combined organic layer was analyzed by GC and LRMS.

To a 10-mL round-bottom flask, 3,5-dimethylbenzoyl chloride (**1a'**: 84.3 mg, 0.500 mmol) and benzenethiol (**2a'**: 55.1 mg, 0.500 mmol) were added. The mixture was stirred at room temperature for 1 min. After the reaction, H_2O (1 mL), and dodecane (20 mg, 0.14 mmol) were added to the reaction mixture, which was then extracted with EtOAc (10 mL). The combined organic layer was analyzed by GC and LRMS.

9.7. Detection of Trimethylsilyl Fluoride by NMR

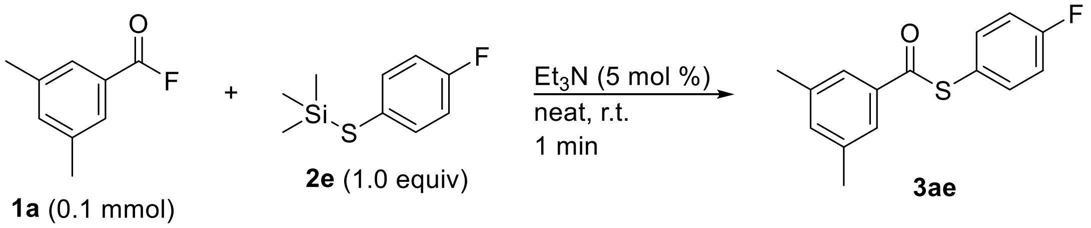

A 10-mL round-bottom flask was charged with 3,5-dimethylbenzoyl fluoride (**1a**: 15.2 mg, 0.100 mmol), triethylamine (0.51 mg, 0.0050 mmol), and (4-fluorophenylthio)trimethylsilane (**2e**: 20.0 mg, 0.100 mmol). The mixture was stirred at room temperature for 1 min. After the reaction, the crude material was measured. Trimethylsilyl fluoride was detected (Figure S1 to S3). ^{1}H NMR (400 MHz, $CDCl_3$) δ 0.26 (s); ^{13}C NMR (100 MHz, $CDCl_3$) δ 0.65; ^{19}F NMR (376 MHz, $CDCl_3$) δ -157.6.

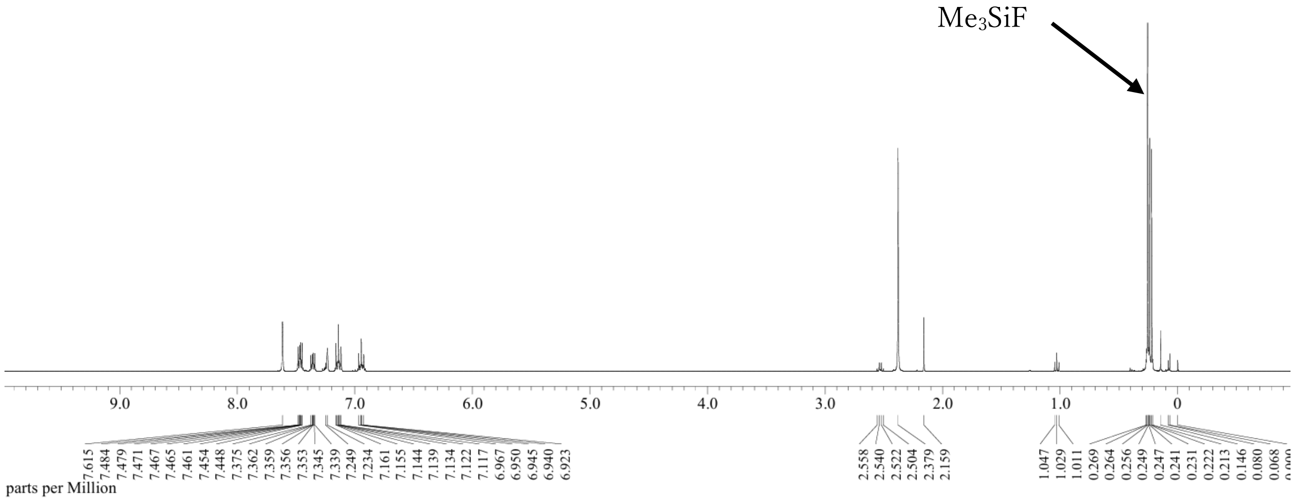

Figure S3. ^{1}H NMR of the crude product after the reaction of **1a** with **2e**

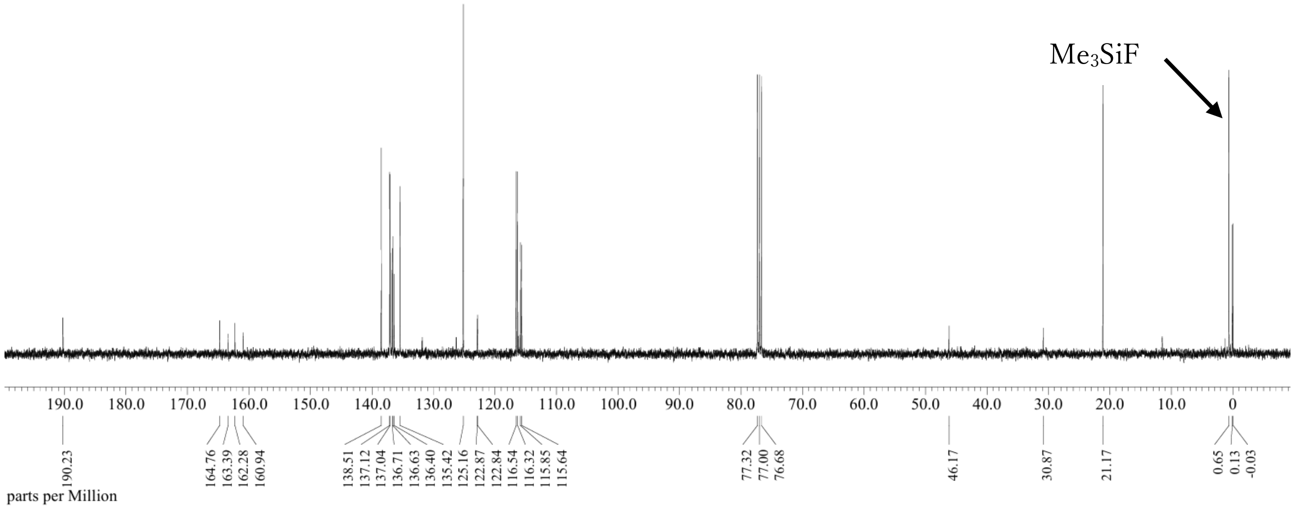

Figure S4. $^{13}C\{^{1}H\}$ NMR of the crude product after the reaction of **1a** with **2e**

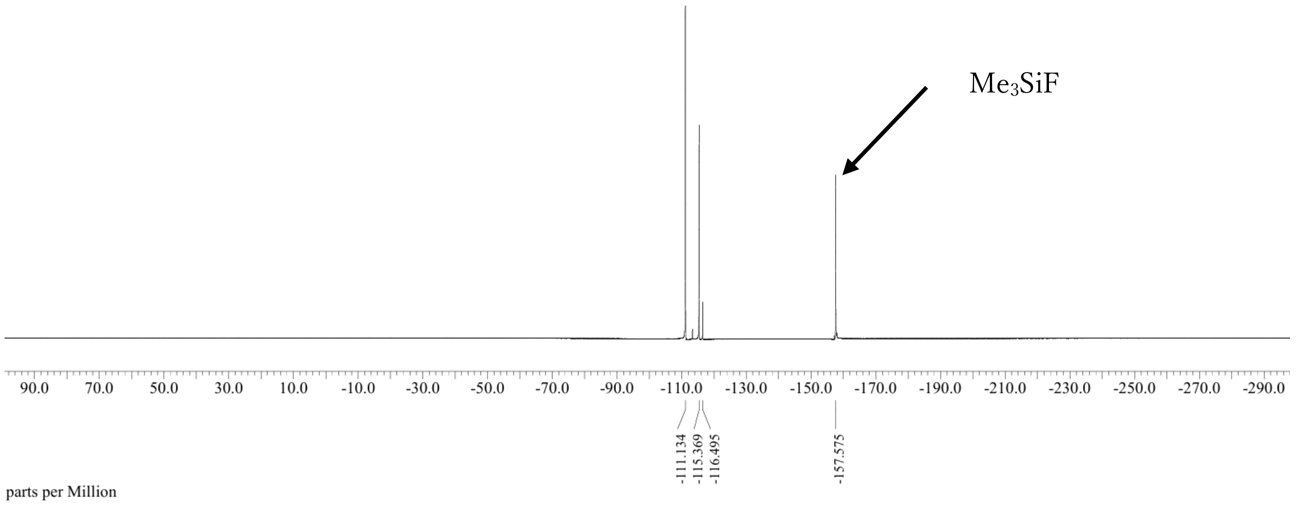

Figure S5. ^{19}F NMR of the crude product after the reaction of **1a** with **2e**

10. Spectra Data

3,5-Dimethybenzoyl fluoride (1a)[5]

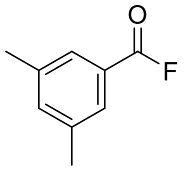

Method A was followed by 3,5-dimethylbenzoyl chloride (4.22 g, 25.0 mmol) for 115 h. Distillation under the reduced pressure afforded 3,5-dimethylbenzoyl fluoride as a transparent solid (2.73 g, 72%): ^{1}H NMR (400 MHz, $CDCl_3$) δ 7.64 (s, 2H), 7.31 (s, 1H), 2.37 (s, 6H); $^{13}C\{^{1}H\}$ NMR (100 MHz, $CDCl_3$) δ 157.7 (d, $J_{C\text{-}F}$ = 343 Hz), 138.8 (d, $J_{C\text{-}F}$ = 10 Hz), 137.0, 129.0 (d, $J_{C\text{-}F}$ = 3.0 Hz), 124.6 (d, $J_{C\text{-}F}$ = 59 Hz); ^{19}F NMR (376 MHz, $CDCl_3$) δ 18.4; MS (EI): *m/z* 152 (M^+).

Benzoyl fluoride (1b)[6]

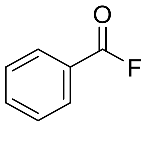

Method A was followed by benzoyl chloride (4.22 g, 30.0 mmol) for 72 h. Distillation under the reduced pressure afforded benzoyl fluoride as a colorless oil (1.97 g, 53%): ^{1}H NMR (400 MHz, $CDCl_3$) δ 8.05 (dd, J = 8.0, 1.2 Hz, 2H), 7.73–7.69 (m, 1H), 7.55–7.51 (m, 2H); $^{13}C\{^{1}H\}$ NMR (100 MHz, $CDCl_3$) δ 157.4 (d, $J_{C\text{-}F}$ = 343 Hz), 135.3, 131.4 (d, $J_{C\text{-}F}$ = 4.0 Hz), 129.0 (d, $J_{C\text{-}F}$ = 1.0 Hz), 124.9 (d, $J_{C\text{-}F}$ = 61 Hz); ^{19}F NMR (376 MHz, $CDCl_3$) δ 18.2; MS (EI): *m/z* 124 (M^+).

4-Methylbenzoyl fluoride (1c)[6]

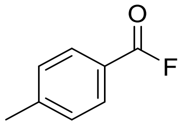

Method A was followed by 4-methylbenzoyl chloride (1.55 g, 11.0 mmol). Distillation under the reduced pressure afforded 4-methylbenzoyl fluoride as a colorless oil (1.10 g, 72%): ^{1}H NMR (400 MHz, $CDCl_3$) δ 7.92 (d, *J* = 8.0 Hz, 2H), 7.32 (d, *J* = 8.0 Hz, 2H), 2.45 (s, 3H); ^{13}C{^{1}H} NMR (100 MHz, $CDCl_3$) *δ* 157.5 (d, $J_{C\text{-}F}$ = 342 Hz), 146.6, 131.4 (d, $J_{C\text{-}F}$ = 4.0 Hz), 129.7, 122.0 (d, $J_{C\text{-}F}$ = 60 Hz), 21.9; ^{19}F NMR (376 MHz, $CDCl_3$) *δ* 17.5; MS (EI): *m/z* 138 (M^+).

4-Phenylbenzoyl fluoride (1d)[7]

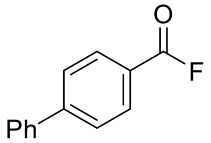

Method A was followed by 4-phenylbenzoyl chloride (1.98 g, 10.0 mmol). Column chromatography (hexane) afforded 4-phenylbenzoyl fluoride as a white solid (1.20 g, 60%): ^{1}H NMR (400 MHz, $CDCl_3$) *δ* 8.11 (d, *J* = 8.0 Hz, 2H), 7.74 (dd, *J* = 8.8, 1.6 Hz, 2H), 7.65–7.62 (m, 2H), 7.52–7.42 (m, 3H); ^{13}C{^{1}H} NMR (100 MHz, $CDCl_3$) *δ* 157.4 (d, $J_{C\text{-}F}$ = 343 Hz), 148.1, 139.2, 132.0 (d, $J_{C\text{-}F}$ = 4.0 Hz), 129.1, 128.8, 127.6, 127.4, 123.4 (d, $J_{C\text{-}F}$ = 61 Hz); ^{19}F NMR (376 MHz, $CDCl_3$) *δ* 18.2; MS (EI): *m/z* 200 (M^+).

4-Fluorobenzoyl fluoride (1e)[8]

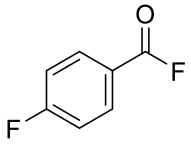

Method A was followed by 4-fluorobenzoyl chloride (4.76 g, 30.0 mmol) for 72 h. Distillation under the reduced pressure afforded 4-fluorobenzoyl fluoride as a colorless oil (2.09 g, 49%): ^{1}H NMR (500 MHz, $CDCl_3$) *δ* 8.11–8.07 (m, 2H), 7.24–7.20 (m, 2H); ^{13}C{^{1}H} NMR (126 MHz, $CDCl_3$) *δ* 167.1 (d, $J_{C\text{-}F}$ = 258 Hz), 156.4 (d, $J_{C\text{-}F}$ = 343 Hz), 134.2 (q, $J_{C\text{-}F}$ = 5.0 Hz), 121.2 (dd, $J_{C\text{-}F}$ = 63, 2.5 Hz), 116.5 (d, $J_{C\text{-}F}$ = 21.4 Hz); ^{19}F NMR (471 MHz, $CDCl_3$) *δ* 18.1, -100.5; MS (EI): *m/z* 142 (M^+).

4-Methoxybenzoyl fluoride (1r)[6]

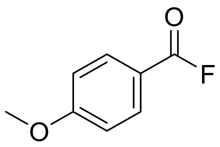

Method A was followed by 4-methoxybenzoyl chloride (3.41 g, 20.0 mmol). Distillation under the reduced pressure afforded 4-methoxybenzoyl fluoride as a colorless oil (1.82 g, 59%): ^{1}H NMR (400 MHz, $CDCl_3$) δ 7.98 (d, J = 9.2 H, 2H), 6.98 (dd, J = 9.2, 1.2 Hz, 2H), 3.90 (s, 3H); ^{13}C{^{1}H} NMR (100 MHz, $CDCl_3$) δ 165.2, 157.2 (d, $J_{C\text{-}F}$ = 338 Hz), 133.7 (d, $J_{C\text{-}F}$ = 4.0 Hz), 116.7 (d, $J_{C\text{-}F}$ = 62 Hz), 114.4, 55.6; ^{19}F NMR (376 MHz, $CDCl_3$) δ 16.0; MS (EI): *m/z* 154 (M^+).

3,5-Dimethoxybenzoyl fluoride (1s)[9]

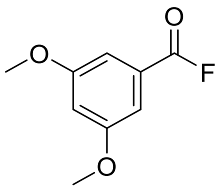

Method C was followed by 3,5-dimethoxybenzoyl chloride (1.82 g, 10.0 mmol). Column chromatography (hexane/EtOAc) afforded 3,5-dimethoxybenzoyl fluoride as a white solid (0.96 g, 52%); ^{1}H NMR (500 MHz, $CDCl_3$) δ 7.17 (d, J = 2.5 Hz, 2H), 6.76 (t, J = 2.5 Hz, 1H), 3.84 (s, 6H); ^{13}C{^{1}H} NMR (126 MHz, $CDCl_3$) δ 161.0, 157.3 (d, $J_{C\text{-}F}$ = 334.7 Hz), 126.5 (d, $J_{C\text{-}F}$ = 60.4 Hz), 108.8 (d, $J_{C\text{-}F}$ = 3.8 Hz), 108.0, 55.7; ^{19}F NMR (376 MHz, $CDCl_3$) δ 18.9; MS (EI): *m/z* 184 (M^+).

2,4,6-Trimethylbenzoyl fluoride (1f)[9]

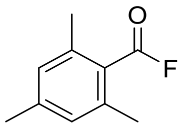

Method A was followed by 2,4,6-trimethylbenzoyl chloride. Column chromatography (hexane) afforded 2,4,6-trimethylbenzoyl fluoride as a white solid (2.63 g, 76%): ^{1}H NMR (400 MHz, $CDCl_3$) δ 6.92 (s, 2H), 2.44 (d, J = 4.0 Hz, 6H), 2.31 (s, 3H); ^{13}C{^{1}H} NMR (100 MHz, $CDCl_3$) δ 158.5 (d, $J_{C\text{-}F}$ = 351 Hz), 142.7, 139.5, 129.6 (d, $J_{C\text{-}F}$ = 2.0 Hz), 123.4 (d, $J_{C\text{-}F}$ = 54 Hz); ^{19}F NMR (376 MHz, $CDCl_3$) δ 52.6; MS (EI): *m/z* 166 (M^+).

2-Iodobenzoyl fluoride (1g)[11]

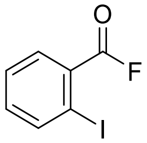

Method C was followed by 2-iodobenzoic acid (2.48g, 10.0 mmol). Column chromatography (hexane : EtOAc = 9 : 1) afforded 2-iodobenzoyl fluoride as a white solid (1.76 g, 70%): ^{1}H NMR (400 MHz, $CDCl_3$) δ 8.14–8.11 (m, 1H), 8.02 (dd, J = 8.0, 2.0 Hz, 1H), 7.51 (td, J = 7.6, 1.2 Hz, 1H), 7.33–7.29 (m, 1H); ^{13}C{^{1}H} NMR (100 MHz, $CDCl_3$) δ 155.1 (d, $J_{C\text{-}F}$ = 344 Hz), 142.6 (d, $J_{C\text{-}F}$ = 4.0 Hz), 135.2, 133.4 (d, $J_{C\text{-}F}$ = 2.0 Hz), 128.4 (d, $J_{C\text{-}F}$ = 15 Hz), 127.9, 97.1 (d, $J_{C\text{-}F}$ = 6.0 Hz); ^{19}F NMR (376 MHz, $CDCl_3$) δ 28.8; MS (EI): *m/z* 250 (M^+).

2,6-Difluorobenzoyl fluoride (1h)[6]

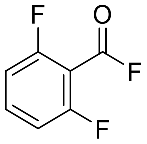

Method B was followed by 2,6-difluorobenzoyl chloride (1.16 g, 10.0 mmol) and iodoethane (3.12 g, 20.0 mmol) for 2 h. Distillation under the reduced pressure afforded 2,6-difluorobenzoyl fluoride as a white solid (0.79 g, 25%): ^{1}H NMR (400 MHz, $CDCl_3$) δ 7.70–7.63 (m, 1H), 7.10–7.06 (m, 2H); ^{13}C{^{1}H} NMR (126 MHz, $CDCl_3$) δ 162.4 (dd, $J_{C\text{-}F}$ = 264.2, 3.8 Hz), 150.7 (d, $J_{C\text{-}F}$ = 343.4 Hz), 136.5 (dd, $J_{C\text{-}F}$ = 11.3 Hz), 112.8 (ddd, $J_{C\text{-}F}$ = 23.0, 4.0, 1.0 Hz), 104.7 (dt, J = 104, 14 Hz); ^{19}F NMR (471 MHz, $CDCl_3$) δ 47.8 (t, $J_{F\text{-}F}$ = 23.5 Hz), -104.8 (d, $J_{F\text{-}F}$ = 47.1); MS (EI): *m/z* 160 (M^+).

2,3,4,5,6-Pnetafluorobenzoyl fluoride (1i)[6]

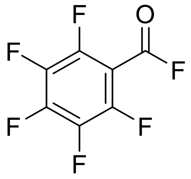

Method A was followed by 2,3,4,5,6-pentafluorobenzoyl chloride (2.31 g, 10.0 mmol), for 24 h. Distillation under the reduced pressure afforded 2,3,4,5,6-pentafluorobenzoyl fluoride as a colorless oil (0.50 g, 23%); ^{13}C{^{1}H} NMR (100 MHz, $CDCl_3$) δ 148.6 (dd, $J_{C\text{-}F}$ = 343, 1.0 Hz). 148.9–146.0 (m), 147.3–144.3 (m), 139.6–136.7 (m), 102.7–101.8 (m); ^{19}F NMR (376 MHz, $CDCl_3$) δ 47.7–47.1 (m, 1 F), -133.8 (s, 2 F), -141.7–-142.0 (m, 1 F), -159.0–-159.4 (m, 2 F); MS (EI): *m/z* 214 (M^+).

1-Naphthoyl fluoride (1j)[12]

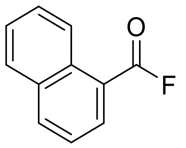

Method A was followed by 1-naphthoyl chloride (1.72 g, 10.0 mmol). Column chromatography (hexane) afforded 1-naphthoyl fluoride as a white solid (1.28 g, 73%): ^{1}H NMR (400 MHz, $CDCl_3$) δ 9.00 (d, J = 8.8 Hz, 1H), 8.33 (dd, J = 7.2, 1.2 Hz, 1H), 8.16 (d, J = 8.4 Hz, 1H), 7.92 (d, J = 8.4 Hz, 1H), 7.72–7.62 (m, 1H), 76.0–7.52 (m, 2H); ^{13}C{^{1}H} NMR (100 MHz, $CDCl_3$) δ 156.4 (d, $J_{C\text{-}F}$ = 343 Hz), 136.6, 133.8, 133.8, 133.7, 133.6, 132.1 (d, $J_{C\text{-}F}$ = 7.0 Hz), 129.1, 129.0, 125.1, 124.5, 120.2 (d, $J_{C\text{-}F}$ = 56 Hz); ^{19}F NMR (376 MHz, $CDCl_3$) δ 30.0; MS (EI): *m/z* 174 (M^+).

Heptanoyl fluoride (1k)[13]

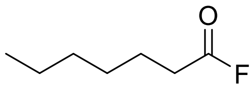

Method B was followed by heptanoyl chloride (1.49 g, 10.0 mmol) for 5 h. Column chromatography (hexane) afforded heptanoyl fluoride as a colorless oil (0.28 g, 21%): ^{1}H NMR (400 MHz, $CDCl_3$) δ 2.52–2.48 (m, 2H), 1.68 (quin, J = 7.6 Hz, 2H), 1.41–1.26 (m, 6H), 0.90 (t, J = 6.8 Hz, 3H); ^{13}C{^{1}H} NMR (100 MHz, $CDCl_3$) δ 163.6 (d, $J_{C\text{-}F}$ = 359 Hz), 32.1 (d, $J_{C\text{-}F}$ = 50 Hz), 31.2, 28.3, 23.9 (d, $J_{C\text{-}F}$ = 2 Hz), 22.4, 13.9; ^{19}F NMR (376 MHz, $CDCl_3$) δ 45.4; MS (EI): *m/z* 132 (M^+).

Palmitoyl fluoride(1l)[7]

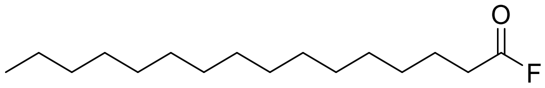

Method B was followed by palmitoyl chloride (8.25 g, 30.0 mmol). Column chromatography (hexane) afforded palmitoyl fluoride as a white solid (7.04 g, 91%): ^{1}H NMR (400 MHz, $CDCl_3$) δ 2.49 (td, J = 7.2, 0.8 Hz, 2H), 1.67 (quin, J = 7.6 Hz, 2H), 1.63–1.26 (brs, 24H), 0.88 (t, J = 6.8 Hz, 3H); ^{13}C{^{1}H} NMR (100 MHz, $CDCl_3$) δ 163.6 (d, $J_{C\text{-}F}$ = 359 Hz), 32.4, 31.9, 31.9, 29.7, 29.6, 29.5, 29.4, 29.3, 29.1, 28.7, 23.9, 22.7, 14.1; ^{19}F NMR (376 MHz, $CDCl_3$) δ 45.5; MS (EI): *m/z* 258 (M^+).

Cyclohexanoyl fluoride (1m)[14]

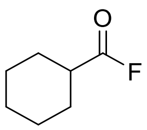

Method A was followed by cyclohexanoyl chloride (4.25 g, 29.0 mmol). Distillation under the reduced pressure afforded cyclohexanoyl fluoride as a colorless oil (1.33 g, 35%): ^{1}H NMR (500 MHz, $CDCl_3$) δ 2.44–2.38 (m, 1H), 1.98–1.95 (m, 2H), 1.81–1.77 (m, 2H), 1.67–1.63 (m, 1H), 1.53–

1.45 (m, 2H), 1.34–1.21 (m, 3H); $^{13}C\{^1H\}$ NMR (100 MHz, $CDCl_3$) δ 165.6 (d, $J_{C\text{-}F}$ = 459.9 Hz), 41.3 (d, $J_{C\text{-}F}$ = 58 Hz), 28.0, 25.3, 24.9; ^{19}F NMR (471 MHz, $CDCl_3$) δ 36.8; MS (EI): *m/z* 130 (M^+).

1-Adamantanoyl fluoride (1n)[10]

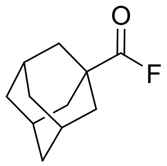

Method A was followed by 1-adamantanoyl chloride (3.97 g, 20.0 mmol). Column chromatography (hexane) afforded 1-adamantanoyl fluoride as a white solid (3.00 g, 82%): ^1H NMR (400 MHz, $CDCl_3$) δ 2.07 (m, 3H), 1.97–1.96 (m, 6H), 1.76–1.70 (m, 6H); $^{13}C\{^1H\}$ NMR (100 MHz, $CDCl_3$) δ 167.0 (d, $J_{C\text{-}F}$ = 370 Hz), 40.4 (d, $J_{C\text{-}F}$ = 44 Hz), 37.8, 36.0, 27.3; ^{19}F NMR (376 MHz, $CDCl_3$) δ 23.9; MS (EI) *m/z* 182 (M^+).

1-Phenyl-1-cyclopentanecarbonyl fluoride (1o)

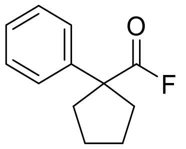

Method C was followed by 1-phenyl-1-cyclopentanecarboxylic acid (5.71 g, 30.0 mmol) for 30 h. Column chromatography (hexane) afforded 1-phenyl-1-cyclopentanecarbonyl fluoride as a colorless oil (3.80 g, 66%): ^1H NMR (400 MHz, $CDCl_3$) δ 7.40–7.25 (m, 5H), 2.67–2.61 (m, 2H), 2.05–1.98 (m, 2H), 1.86–1.76 (m, 4H); $^{13}C\{^1H\}$ NMR (100 MHz, $CDCl_3$) δ 165.8 (d, $J_{C\text{-}F}$ = 369 Hz), 140.1 (d, $J_{C\text{-}F}$ = 3.0 Hz), 128.7, 127.7, 126.9, 58.3 (d, $J_{C\text{-}F}$ = 42 Hz), 36.0, 23.6; ^{19}F NMR (376 MHz, $CDCl_3$) δ 29.4; HRMS (FAB-Magnetic Sector): *m/z* calcd for $C_{12}H_{13}FO$ (M^+): 192.0950; found: 192.0949.

1-Phenyl-1-cyclopropanecarbonyl fluoride (1p)

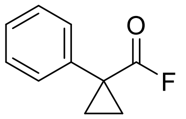

Method C was followed by 1-phenyl-1-cyclopropanecarboxylic acid (1.62 g, 10.0 mmol) for 23 h. Column chromatography (hexane) afforded 1-phenyl-1-cyclopropanecarbonyl fluoride as a colorless oil (1.50 g, 91%): ^1H NMR (400 MHz, $CDCl_3$) δ 7.38–7.30 (m, 5H), 1.81–1.78 (m, 2H), 1.45–1.42 (m, 2H); $^{13}C\{^1H\}$ NMR (100 MHz, $CDCl_3$) δ 164.9 (d, $J_{C\text{-}F}$ = 349 Hz), 136.6 (d, $J_{C\text{-}F}$ = 1.0 Hz), 130.2, 128.6, 128.1, 27.1 (d, $J_{C\text{-}F}$ = 62 Hz), 17.1; ^{19}F NMR (376 MHz, $CDCl_3$) δ 24.6; LRMS (EI): *m/z* 164 (M^+). Anal. calcd for $C_{10}H_9FO$: C, 73.16; H, 5.53; found: C, 73.28; H, 5.71.

Cinnamoyl fluoride (1q)[14]

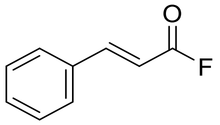

Method B was followed by cinnamoyl chloride (5.00 g, 30.0 mmol) for 24 h. C Distillation under the reduced pressure afforded cinnamoyl fluoride as a transparent solid (1.70 g, 38%): ^{1}H NMR (400 MHz, $CDCl_3$) δ 7.83 (d, J = 16 Hz, 1H), 7.57–7.54 (m, 2H), 7.49–7.41 (m, 3H), 6.36 (dd, J = 16, 7.6 Hz, 1H); $^{13}C\{^{1}H\}$ NMR (100 MHz, $CDCl_3$) δ 157.1 (d, $J_{C\text{-}F}$ = 338 Hz), 151.4 (d, $J_{C\text{-}F}$ = 5.0 Hz), 133.1, 131.8, 129.1, 128.7, 112.0 (d, $J_{C\text{-}F}$ = 67 Hz); ^{19}F NMR (376 MHz, $CDCl_3$) δ 25.7; MS (EI): *m/z* 150 (M^+).

(Phenylthio)trimethylsilane (2a)[15]

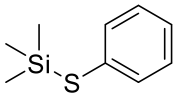

Preparation of the thiosilane was followed by benzenethiol (11.0 g, 100 mmol) and THF (100 mL) for 24 h. Distillation under the reduced pressure afforded (phenylthio)trimethylsilane as a colorless oil (15.0 g, 82%): ^{1}H NMR (400 MHz, $CDCl_3$) δ 7.42–7.40 (m, 2H), 7.25–7.23 (m, 3H), 0.27 (s, 9H); $^{13}C\{^{1}H\}$ NMR (100 MHz, $CDCl_3$) δ 135.1, 131.4, 128.7, 126.8, 0.81; MS (EI): *m/z* 182 (M^+).

Trimethyl(4-methylphenylthio)silane (2b)[16]

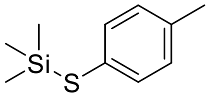

Preparation of the thiosilane was followed by 4-methylbenzenethiol (1.24 g, 10.0 mmol) and THF (10 mL) for 2 h. Distillation under the reduced pressure afforded trimethyl(4-methylphenylthio)silane as a colorless oil (1.82 g, 93%): ^{1}H NMR (400 MHz, $CDCl_3$) δ 7.30–7.27 (m, 2H), 7.06–7.04 (m, 2H), 2.31 (s, 3H), 0.26 (s, 9H); $^{13}C\{^{1}H\}$ NMR (100 MHz, $CDCl_3$) δ 136.6, 135.0, 129.5, 127.5, 21.0, 0.76; MS (EI): *m/z* 196 (M^+).

Trimethyl(4-methoxyphenylthio)silane (2c)[17]

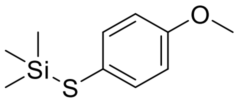

Preparation of the thiosilane was followed by 4-methoxybenzenethiol (1.40 g, 10.0 mmol) and THF (10 mL) for 1 h. Distillation under the reduced pressure afforded trimethyl(4-methoxyphenylthio)silane as a colorless oil (1.40 g, 66%). ^{1}H NMR (400 MHz, $CDCl_3$) δ 7.32–7.30

(m,2H), 6.80–6.79 (dd, J = 6.4, 2.0 Hz, 2H), 3.78 (s, 3H), 0.25 (s, 9H); $^{13}C\{^{1}H\}$ NMR (100 MHz, $CDCl_3$) δ 158.9, 136.3, 132.4, 114.3, 55.2, 0.70; MS (EI): *m/z* 212 (M^+).

Trimethyl(3-methylphenylthio)silane (2d)[16]

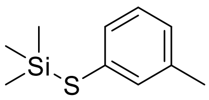

Preparation of the thiosilane was followed by 3-methylbenzenethiol (1.40 g, 10.0 mmol) and THF (10 mL) for 1 h. Distillation under the reduced pressure afforded trimethyl(3-methylphenylthio)silane as a colorless oil (1.40 g, 66%): ^1H NMR (400 MHz, $CDCl_3$) δ 7.23–7.20 (m, 2H), 7.13 (t, J = 7.6 Hz, 1 H), 7.04 (d, J = 7.6 Hz, 1 H), 2.31 (s, 3H), 0.27 (s, 9H); $^{13}C\{^{1}H\}$ NMR (100 MHz, $CDCl_3$) δ 138.4, 135.7, 132.0, 131.0, 128.5, 127.6, 21.3, 0.84; MS (EI): *m/z* 196 (M^+).

(4-Fluorophenylthio)trimethylsilane (2e)[16]

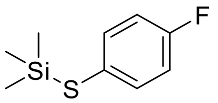

Preparation of the thiosilane was followed by 4-fluorobenzenethiol (1.28 g, 10.0 mmol) and THF (10 mL) for 1 h. Distillation under the reduced pressure afforded (4-fluorophenylthio)trimethylsilane as a colorless oil (1.59 g, 80%): ^1H NMR (400 MHz, $CDCl_3$) δ 7.38–7.35 (m, 2H), 6.95 (t, J = 8.8Hz, 2 H), 0.26 (s, 9H); $^{13}C\{^{1}H\}$ NMR (100 MHz, $CDCl_3$) δ 162.2 (d, $J_{C\text{-}F}$ = 246 Hz), 136.7 (d, $J_{C\text{-}F}$ = 8.0 Hz), 126.3 (d, $J_{C\text{-}F}$ = 4.0 Hz), 115.8 (d, $J_{C\text{-}F}$ = 21 Hz); ^{19}F NMR (376 MHz, $CDCl_3$) δ -115.4, 0.68; HRMS (FAB-Magnetic Sector): *m/z* calcd for $C_9H_{13}FSSi$ (M^+): 200.0491; found: 200.0488.

(4-Chlorophenylthio)trimethylsilane (2f)[16]

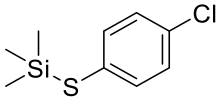

Preparation of the thiosilane was followed by 4-chlorobenzenethiol (1.45 g, 10.0 mmol) and THF (10 mL) for 2 h. Distillation under the reduced pressure afforded (4-chlorophenylthio)trimethylsilane as a colorless oil (1.95 g, 90%): ^1H NMR (400 MHz, $CDCl_3$) δ 7.34–7.32 (m, 2H), 7.23–7.20 (m, 2H), 0.27 (s, 9H); $^{13}C\{^{1}H\}$ NMR (100 MHz, $CDCl_3$) δ 136.3, 133.1, 130.7, 128.8, 0.75; MS (EI): *m/z* 216 (M^+).

(4-Fluorophenylthio)trimethylsilane (2g)[16]

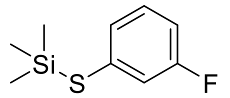

Preparation of the thiosilane was followed by 3-fluorobenzenethiol (1.28 g, 10.0 mmol) and THF (10 mL) for 1 h. Distillation under the reduced pressure afforded (3-fluorophenylthio)trimethylsilane as a colorless oil (1.44 g, 72%): ^{1}H NMR (400 MHz, $CDCl_3$) δ 7.26–7.17 (m, 2H), 7.15–7.12 (m, 1H), 6.97–6.91 (m, 1H) 0.29 (s, 9H); ^{13}C{^{1}H} NMR (100 MHz, $CDCl_3$) δ 162.3 (d, J_{C-F} = 248 Hz), 133.7 (d, J_{C-F} = 8.0 Hz), 130.8 (d, J_{C-F} = 3.0 Hz), 129.8 (d, J_{C-F} = 9.0 Hz), 121.8 (d, J_{C-F} = 21 Hz), 114.0 (d, J_{C-F} = 21 Hz), 0.79; ^{19}F NMR (376 MHz, $CDCl_3$) δ = -112.6; HRMS (FAB-Magnetic Sector): *m/z* calcd for $C_9H_{14}FSSi$ (M^++H): 201.0564; found: 201.0571.

(3-Chlorophenylthio)trimethylsilane (2h)[16]

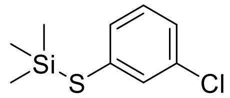

Preparation of the thiosilane was followed by 3-chlorobenzenethiol (1.45 g, 10.0 mmol) and THF (10 mL) for 1 h. Distillation under the reduced pressure afforded (3-chlorophenylthio)trimethylsilane as a colorless oil (1.64 g, 76%): ^{1}H NMR (400 MHz, $CDCl_3$) δ 7.42–7.41 (m, 1H), 7.28–7.25 (m, 2H), 7.13 (s, 1H), 0.29 (s, 9H); ^{13}C{^{1}H} NMR (100 MHz, $CDCl_3$) δ 134.6, 133.1, 130.0, 129.6, 128.9, 127.1, 0.78; MS (EI): *m/z* 216 (M^+).

Trimethyl(3,5-dimethylphenylthio)silane (2i)[16]

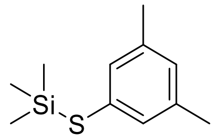

Preparation of the thiosilane was followed by 3,5-dimethylbenzenethiol (1.38 g, 10.0 mmol) and THF (10 mL) for 1 h. Distillation under the reduced pressure afforded trimethyl(3,5-dimethylphenylthio)silane as a colorless oil (0.51 g, 24%): ^{1}H NMR (400 MHz, $CDCl_3$) δ 7.03 (s, 2H), 6.86 (s, 1H), 2.27 (d, J = 0.4 Hz, 6H), 0.27 (s, 9H); ^{13}C{^{1}H} NMR (100 MHz, $CDCl_3$) δ 138.2, 132.7, 130.6, 128.6, 21.1, 0.86; HRMS (FAB-Magnetic Sector): *m/z* calcd for $C_{11}H_{19}SSi$ (M^++H): 211.0977; found: 211.0951.

Trimethyl(2,4-dimethylphenylthio)silane (2j)

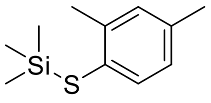

Preparation of the thiosilane was followed by 2,4-dimethylbenzenethiol (1.38 g, 20.0 mmol) and THF (30 mL) for 2 h at 60 C. Distillation under the reduced pressure afforded trimethyl(2,4-dimethylphenylthio)silane as a colorless oil (3.64 g, 94%): ^{1}H NMR (400 MHz, $CDCl_3$) δ 7.27 (d, J = 7.6 Hz, 1H), 7.02 (s, 1H), 6.88 (d, J = 8.0 Hz, 1H), 2.38 (s, 3H), 2.27 (s, 3H), 0.25 (s, 9H); ^{13}C{^{1}H} NMR (100 MHz, $CDCl_3$) δ 141.5, 136.8, 136.1, 131.2, 127.1, 126.8, 21.9, 20.9, 0.91; HRMS (FAB-Magnetic Sector): m/z calcd for $C_{11}H_{18}SSi$ (M^+): 210.0898; found: 210.0899.

(1-Decylthio)trimethylsilane (2k)[17]

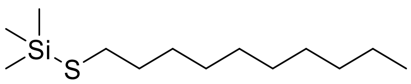

Preparation of the thiosilane was followed by 1-decanethiol (1.74 g, 10.0 mmol) and THF (10 mL) overnight. Distillation under the reduced pressure afforded trimethyl(1-decylthio)silane as a colorless oil (2.32 g, 94%): ^{1}H NMR (500 MHz, $CDCl_3$) δ 2.49 (t, J = 7.2 H, 2H), 1.63–1.56 (m, 2H), 1.38–1.26 (m, 14H), 0.88 (t, J = 6.8 Hz, 3H), 0.31 (s, 9H); ^{13}C{^{1}H} NMR (126 MHz, $CDCl_3$) δ 33.1, 31.9, 29.5, 29.3, 29.2, 28.8, 26.4, 22.7, 14.1, 0.9; MS (EI) m/z 246 (M^+).

(*n*-Butylthio)trimethylsilane (2l)

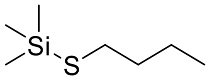

Preparation of the thiosilane was followed by 1-butanethiol (1.80 g, 20.0 mmol) and Et_2O (40 mL) for 4 h. Distillation under the reduced pressure afforded (butylthio)trimethylsilane as a colorless oil (1.44 g, 44%): ^{1}H NMR (400 MHz, $CDCl_3$) δ 2.50 (t, J = 7.2 Hz, 2H), 1.62–1.55 (m, 2H), 1.46–1.37 (m, 2H), 0.91 (t, J = 7.2 Hz, 3H), 0.31 (s, 9H); ^{13}C{^{1}H} NMR (100 MHz, $CDCl_3$) δ 35.1, 26.0, 21.8, 13.6; HRMS (ESI) m/z calcd for $C_7H_{19}SSi$ (M^++H): 163.0971; found: 163.0977.

(*t*-Butylthio)triemthylsilane (2m)[18]

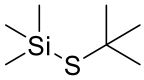

Preparation of the thiosilane was followed by 2-methyl-2-propanethiol (1.80 g, 20.0 mmol) and Et_2O (40 mL). The mixture was stirred 5 h under reflux. Distillation under the reduced pressure afforded (*t*-butylthio)trimethylsilane as a colorless oil (1.38 g, 42%): ^{1}H NMR (400 MHz, $CDCl_3$) δ 1.46 (s, 9H), 0.36 (s, 9H); ^{13}C{^{1}H} NMR (100 MHz, $CDCl_3$) δ 44.7, 35.3, 2.8; MS (EI) m/z 162 (M^+).

Trimethyl(methyl propionylthio)silane (2n)

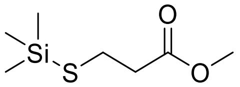

Preparation of the thiosilane was followed by methyl 3-mercaptopropionate (2.4 g, 20 mmol) and THF (20 mL) for 2 h. Distillation under the reduced pressure afforded trimethyl(methyl propionylthio)silane as a colorless oil (2.94 g, 99%): ^{1}H NMR (400 MHz, $CDCl_3$) δ 3.70 (s, 3H), 2.78–2.74 (m, 2H), 2.63–2.59 (m, 2H), 0.33 (s, 9H); ^{13}C{^{1}H} NMR (100 MHz, $CDCl_3$) δ 172.2, 51.7, 37.5, 21.2, 0.77; HRMS (FAB-Magnetic Sector): *m/z* calcd for $C_7H_{17}O_2SSi$ (M^{+}+H): 193.0713; found: 193.0720.

(Furfurylthio)trimethylsilane (2o)

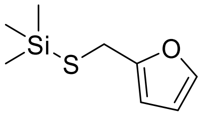

Preparation of the thiosilane was followed by furfuryl mercaptan (1.14 g, 10.0 mmol) and THF (10 mL) for 1 h. Distillation under the reduced pressure afforded (furfurylthio)trimethylsilane as a colorless oil (1.80 g, 97%): ^{1}H NMR (400 MHz, $CDCl_3$) δ 7.34 (dd, *J* = 2.0, 0.8 Hz, 1H), 6.29 (dd, *J* = 3.2, 2.0 Hz 1H), 6.15 (dd, *J* = 3.6, 0.8 Hz, 1H), 3.73 (s, 2H), 0.29 (s, 9H); ^{13}C{^{1}H} NMR (100 MHz, $CDCl_3$) δ 153.3, 141.7, 110.4, 100.7, 22.6, 0.63; HRMS (FAB-Magnetic Sector): *m/z* calcd for $C_8H_{14}OSSi$ (M^{+}): 186.0534; found: 186.0537.

Triisopropyl(phenylthio)silane (2p)[19]

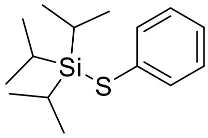

Preparation of the thiosilane was followed by benzenethiol (0.55 g, 5.0 mmol), triisopropylsilyl chloride (0.55 g, 5.0 mmol), and THF (5 mL) for 23 h. Alumina gel column chromatography (hexane) to afford triisopropyl(phenylthio)silane as a colorless oil (1.10 mg, 83%): ^{1}H NMR (400 MHz, $CDCl_3$) δ 7.50–7.48 (m, 2H), 7.22–7.19 (m, 3H), 1.29–1.19 (m, 3H), 1.07 (d, *J* = 6.8 Hz, 18H); ^{13}C{^{1}H} NMR (100 MHz, $CDCl_3$) δ 135.4, 131.5, 128.6, 126.7, 18.4, 13.0; MS (EI): *m/z* 266 (M^{+}).

***S*-Phenyl 3,5-diemthylbenzothioate (3aa)**[20]

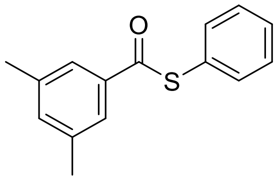

General procedure for the substrate of acyl fluoride derivatives was followed by 3,5-

dimethylbenzoyl fluoride (76.1 mg, 0.500 mmol). Silica gel column chromatography (hexane to hexane : EtOAc = 99:1) afforded *S*-phenyl 3,5-dimethylbenzothioate as a white solid (116.1 mg, 95%): m.p. 45.6–46.2 ˚C; ^{1}H NMR (400 MHz, $CDCl_3$) *δ* 7.63 (s, 2H), 7.52–7.49 (m, 2H), 7.46–7.43 (m, 3H), 7.23 (s, 1H), 2.38 (s, 6H); ^{13}C{^{1}H} NMR (100 MHz, $CDCl_3$) *δ* 190.3, 138.4, 136.6, 135.3, 135.0, 129.4, 129.2, 127.6, 125.1, 21.2; MS (FAB-Magnetic sector): *m/z* calcd for $C_{15}H_{15}OS$ (M^{+}+H): 243.08; found: 243.08.

_S_-Phenyl benzothioate (3ba)[21]

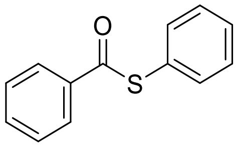

General procedure for the substrate scope of acyl fluorides was followed by benzoyl fluoride (62.1 mg, 0.500 mmol). Silica gel column chromatography (hexane to hexane : EtOAc = 99:1) afforded *S*-phenyl benzothioate as a white solid (117.2 mg, 93%): m.p. 55.7–56.1 ˚C; ^{1}H NMR (500 MHz, $CDCl_3$) *δ* 8.04–8.02 (m, 2H), 7.63–7.59 (m, 1H), 7.54–7.45 (m, 7H); ^{13}C{^{1}H} NMR (126 MHz, $CDCl_3$) *δ* 190.2, 136.6, 135.1, 133.7, 129.5, 129.2, 128.7, 127.5, 127.3; MS (FAB-Magnetic Sector): *m/z* calcd for $C_{13}H_{11}OS$ (M^{+}+H): 215.05; found: 215.11.

_S_-Phenyl 4-methylbenzothioate (3ca)[21]

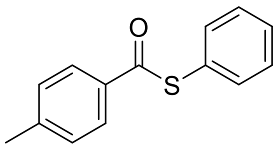

General procedure for the substrate scope of acyl fluorides was followed by 4-methylbenzoyl fluoride (69.1 mg, 0.500 mmol). Silica gel column chromatography (hexane to hexane : EtOAc = 99:1) afforded *S*-phenyl 4-methylbenzothioate as a white solid (115.2 mg, 78%): m.p. 92.0–92.8 ˚C; ^{1}H NMR (400 MHz, $CDCl_3$) *δ* 7.92 (dd, *J* = 8.4, 2.4 Hz, 2H), 7.53–7.44 (m, 5H), 7.29–7.26 (m, 2H), 2.42 (d, *J* = 2.8 Hz, 3H); ^{13}C{^{1}H} NMR (100 MHz, $CDCl_3$) *δ* 189.7, 144.6, 135.1, 134.0, 129.4, 129.2, 129.0, 127.5, 127.4, 21.7; MS (FAB-Magnetic Sector): *m/z* calcd for $C_{14}H_{13}OS$ (M^{+}+H): 229.07; found: 229.12.

S-Phenyl 4-phenylbenzothioate (3da)[22]

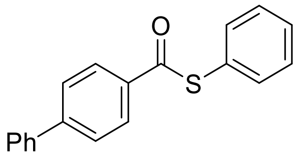

General procedure for the substrate of acyl fluoride derivatives was followed by 4-phenylbenzoyl fluoride (100.1 mg, 0.5000 mmol). Silica gel column chromatography (hexane to hexane : EtOAc = 99:1) afforded *S*-phenyl 4-phenylbenzothioate as a white solid (124.8 mg, 86%): ^{1}H NMR (400 MHz, $CDCl_3$) δ 8.10–8.07 (m, 2H), 7.68 (dd, J = 10.4, 2.0 Hz, 2H), 7.62–7.60 (m, 2H), 7.54–7.50 (m, 2H), 7.48–7.37 (m, 6H); ^{13}C{^{1}H} NMR (100 MHz, $CDCl_3$) δ 189.6, 146.3, 139.6, 135.2, 135.0, 129.5, 129.2, 128.9, 128.3, 128.0, 127.3, 127.2; MS (FAB-Magnetic Sector): *m/z* calcd for $C_{19}H_{15}OS$ (M^++H): 291.08; found: 291.11.

S-Phenyl 4-fluorobenzothioate (3ea)[21]

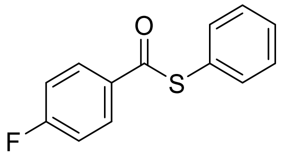

General procedure for the substrate of acyl fluoride derivatives was followed by 4-fluorobenzoyl fluoride (71.1 mg, 0.500 mmol). Silica gel column chromatography (hexane to hexane : EtOAc = 99:1) afforded *S*-phenyl 4-fluorobenzothioate as a white solid (104.1 mg, 87%): ^{1}H NMR (400 MHz, $CDCl_3$) δ 8.08–8.03 (m, 2H), 7.52–7.44 (m, 5H), 7.15 (t, J = 8.0 Hz, 2H); ^{13}C{^{1}H} NMR (100 MHz, $CDCl_3$) δ 188.6, 166.0 (d, $J_{C\text{-}F}$ = 254 Hz), 135.1, 132.9, 130.0 (d, $J_{C\text{-}F}$ = 9.0 Hz), 129.6, 129.3, 127.0, 115.9 (d, $J_{C\text{-}F}$ = 22 Hz); ^{19}F NMR (376 MHz, $CDCl_3$) δ -104.0; MS (FAB-Magnetic Sector): *m/z* calcd for $C_{13}H_{10}FOS$ (M^++H): 233.04; found: 233.05.

S-Phenyl 4-methoxybenzothioate (3ra)[21]

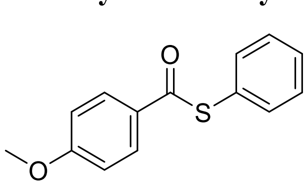

General procedure for the substrate of acyl fluoride derivatives was followed by 4-methoxybenzoyl fluoride (77.1 mg, 0.500 mmol) and Et_3N (2.5 mg, 0.025 mmol). Silica gel column chromatography (hexane to hexane : EtOAc = 99:1) afforded *S*-phenyl 4-methoxybenzothioate as a white solid (119.3 mg, 99%): ^{1}H NMR (400 MHz, $CDCl_3$) δ 8.01 (d, J = 8.8 Hz, 2H), 7.53–7.49 (m, 2H), 7.46–7.42 (m, 3H), 6.95 (d, J = 8.8 Hz, 2H), 3.87 (s, 3H); ^{13}C{^{1}H} NMR (100 MHz, $CDCl_3$) δ 188.6, 163.9, 135.2,

129.7, 129.3, 129.1, 127.6, 113.9, 55.5; MS (FAB-Magnetic Sector): *m/z* calcd for $C_{14}H_{13}O_2S$ (M^++H): 245.06; found: 245.08.

***S*-Phenyl 3,5-dimethoxybenzothioate (3sa)**[23]

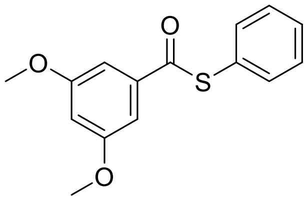

General procedure for the substrate of acyl fluoride derivatives was followed by 3,5-dimethylbenzoyl fluoride (96.1 mg, 0.500 mmol) and Et_3N (2.5 mg, 0.025 mmol). Silica gel column chromatography (hexane to hexane : EtOAc = 99:1) afforded *S*-phenyl 3,5-dimethylbenzothioate as a colorless oil (138.3 mg, 99%): ^{1}H NMR (400 MHz, $CDCl_3$) δ 7.51–7.48 (m, 2H), 7.45–7.43 (m, 3H), 7.15 (d, J = 2.0 Hz, 2.0H), 6.67 (t, J = 2.4 Hz, 1H), 3.81 (s, 6H); $^{13}C\{^1H\}$ NMR (100 MHz, $CDCl_3$) δ 189.9, 160.8, 138.4, 134.9, 129.5, 129.2, 127.3, 105.9, 105.0, 55.5; MS (FAB-Magnetic Sector): *m/z* calcd for $C_{15}H_{15}O_3S$ (M^++H): 275.07; found: 275.07.

***S*-Phenyl 2,4,6-trimethylbenzothioate (3fa)**[21]

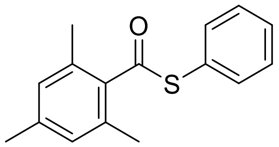

General procedure for the substrate of acyl fluoride derivatives was followed by 2,4,6-trimethylbenzoyl fluoride (83.1 mg, 0.500 mmol). Silica gel column chromatography (hexane to hexane : EtOAc = 99:1) afforded *S*-Phenyl 2,4,6-trimethylbenzothioate as a white solid (4.5 mg, 3%).

A 10-mL round-bottom flask was charged with 2,4,6-trimethylbenzoyl fluoride (83.1 mg, 0.5 mmol), Et_3N (2.5 mg, 0.025 mmol), and trimethyl(phenylthio)silane (91.1 mg, 0.500 mmol). The mixture was stirred at room temperature for 1 min under air. Column chromatography (hexane to hexane : EtOAc = 99:1) afforded *S*-phenyl 2,4,6-trimethylbenzenecarbothioate as a white solid (49.9 mg, 39%): ^{1}H NMR (400 MHz, $CDCl_3$) δ 7.54–7.51 (m, 2H), 7.48–7.42 (m, 3H), 6.86 (d, J = 0.4 Hz, 2H), 2.38 (s, 6H), 2.29 (s, 3H); $^{13}C\{^1H\}$ NMR (100 MHz, $CDCl_3$) δ 196.0, 139.5, 137.2, 134.3, 133.7, 129.5, 129.3, 128.4, 128.0, 21.1, 19.0; MS (EI): *m/z* 147 (M^+-SC_6H_5).

S-Phenyl 2-iodobenzothioate (3ga)[24]

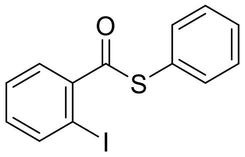

General procedure for the substrate scope of acyl fluorides was followed by 2-iodobenzoyl fluoride (125 mg, 0.500 mmol). Silica gel column chromatography (hexane to hexane : EtOAc = 99:1) afforded *S*-phenyl 2-iodobenzothioate as a white solid (170.4 mg, 80%): ^{1}H NMR (400 MHz, $CDCl_3$) δ 7.96 (dd, J = 8.0, 0.8 Hz, 1H), 7.71 (dd, J = 6.4, 1.6 Hz, 1H), 7.57–7.54 (m, 2H), 7.48–7.42 (m, 4H), 7.18 (td, J = 7.6, 1.6 Hz, 1H); ^{13}C{^{1}H} NMR (125 MHz, $CDCl_3$) δ 192.3, 142.3, 140.8, 134.5, 132.4, 129.7, 129.3, 128.6, 128.0, 127.4, 91.6; MS (FAB-Magnetic Sector): *m/z* calcd for $C_{13}H_{10}IOS$ (M^++H): 340.95; found: 340.98.

S-Phenyl 2,6-diflorobenzothioate (3ha)

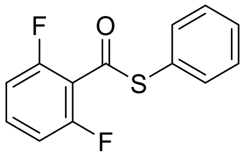

General procedure for the substrate of acyl fluoride derivatives was followed by 2,6-difluorobenzoyl fluoride (80.1 mg, 0.500 mmol). Silica gel column chromatography (hexane to hexane : EtOAc = 99:1) afforded *S*-phenyl 2,6-difluorobenzothioate as a white solid (126.5 mg, 76%): m.p. 69.1–70.0 ˚C; ^{1}H NMR (400 MHz, $CDCl_3$) δ 7.56–7.38 (m, 6H), 7.00–6.93 (m, 2H); ^{13}C{^{1}H} NMR (100 MHz, $CDCl_3$) δ 185.2, 159.0 (dd, $J_{C\text{-}F}$ = 253, 6.0 Hz), 134.7, 132.7 (t, $J_{C\text{-}F}$ = 10 Hz), 130.0, 129.4, 129.0, 127.4, 112.0 (d, $J_{C\text{-}F}$ = 25 Hz); ^{19}F NMR (376 MHz, $CDCl_3$) δ 108.3; HRMS (FAB-Magnetic Sector): *m/z* calcd for $C_{13}H_9F_2OS$ (M^++H): 251.0342; found: 251.0356.

S-Phenyl 2,3,4,5,6-pentafluorobenzothioate (3ia)

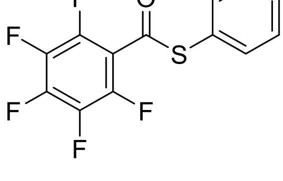

General procedure for the substrate of acyl fluoride derivatives was followed by 2,3,4,5,6-pentafluorobenzoyl fluoride (107 mg, 0.500 mmol). Silica gel column chromatography (hexane to hexane : EtOAc = 99:1) afforded *S*-phenyl 2,3,4,5,6-pentafluorobenzothioate as a white solid (152.5 mg, 99%): m.p. 44.9–45.5 ˚C; ^{1}H NMR (400 MHz, $CDCl_3$) δ 7.55–7.46 (m, 5H); ^{13}C{^{1}H} NMR (100 MHz, $CDCl_3$) δ 182,2, 145.0–144.0 (m), 142.4–141.4 (m), 139.0–136.1 (m), 134.6, 130.5, 129.6,

125.6, 114.3–113.9 (m); ^{19}F NMR (376 MHz, $CDCl_3$) δ -139.6 (d, $J_{F\text{-}F}$ = 17.7 Hz), -148.7 (t, $J_{F\text{-}F}$ = 18.0 Hz), -159.3—159.4 (m); HRMS (FAB-Magnetic Sector): *m/z* calcd for $C_{13}H_6F_5OS$ (M^++H): 305.0054; found: 305.0053.

S-Phenyl 1-naphthalencarbothioate (3ja)[21]

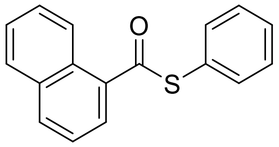

General procedure for the substrate of acyl fluoride derivatives was followed by 1-naphthoyl fluoride (87.1 mg, 0.500 mmol). Silica gel column chromatography (hexane to hexane : EtOAc = 99:1) afforded *S*-phenyl 1-naphthalenecarbothioate as a white solid (93.1 mg, 70%): ^{1}H NMR (400 MHz, $CDCl_3$) δ 8.54 (dd, J = 8.4, 0.8 Hz, 1H), 8.21 (dd, J = 7.6, 1.2 Hz, 1H), 8.01 (d, J = 8.0 Hz, 1H), 7.88–7.85 (m, 1H), 7.61–7.46 (m, 8H); ^{13}C{^{1}H} NMR (100 MHz, $CDCl_3$) δ 192.2, 134.9, 134.6, 133.8, 133.2, 129.5, 129.3, 128.3, 128.2, 128.1, 128.0, 126.7, 125.2, 124.4; MS (FAB-Magnetic Sector): *m/z* calcd for $C_{17}H_{13}OS$ (M^++H): 265.07; found: 265.07.

S-Phenyl heptanethioate (3ka)[25]

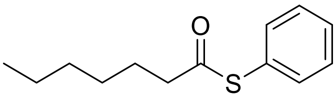

General procedure for the substrate of acyl fluoride derivatives was followed by 1-heptanoyl fluoride (66.1 mg, 0.500 mmol). Silica gel column chromatography (hexane to hexane : EtOAc = 99:1) afforded *S*-phenyl heptanethioate as a colorless oil (78.4 mg, 67%): ^{1}H NMR (400 MHz, $CDCl_3$) δ 7.42–7.37 (m, 5H), 2.64 (t, J = 7.6 Hz, 2H), 1.70 (quin, J = 7.2 Hz, 2H), 1.39–1.25 (m, 6H), 0.89 (t, J = 6.8 Hz, 3H); ^{13}C{^{1}H} NMR (100 MHz, $CDCl_3$) δ 197.4, 134.4, 129.2, 129.1, 127.9, 43.6, 31.4, 28.6, 25.5, 22.4, 14.0; MS (FAB-Magnetic Sector): *m/z* calcd for $C_{13}H_{19}OS$ (M^++H): 223.12; found: 223.12.

S-Phenyl hexadecanethioate (3la)[26]

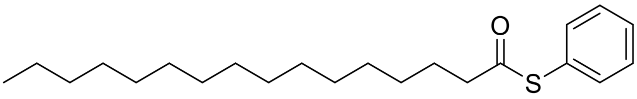

General procedure for the substrate of acyl fluoride derivatives was followed by palmitoyl fluoride (129.2 mg, 0.5000 mmol). Silica gel column chromatography (hexane to hexane : EtOAc = 99:1) afforded *S*-phenyl hexadecanethioate as a white solid (74.6 mg, 42%): ^{1}H NMR (400 MHz, $CDCl_3$) δ 7.40 (s, 5H), 2.65 (t, J = 7.6 Hz, 2H), 1.71 (quin, J = 7.6 Hz, 2H), 1.53 (s, 24H), 0.88 (t, J = 6.8 Hz,

3H); $^{13}C\{^1H\}$ NMR (100 MHz, $CDCl_3$) δ 197.6, 134.5, 129.3, 129.1, 127.9, 43.7, 31.9, 29.7, 29.7, 29.6, 29.6, 29.6, 29.4, 29.4, 29.2, 28.9, 25.6, 22.7, 14.1; MS (FAB-Magnetic Sector): *m/z* calcd for $C_{22}H_{37}OS$ (M^++H): 349.26; found: 349.22.

_S_-Phenyl cyclohexanethioate (3ma)[21]

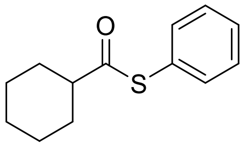

General procedure for the substrate of acyl fluoride derivatives was followed by cyclohexanoyl fluoride (65.1 mg, 0.500 mmol). Silica gel column chromatography (hexane to hexane : EtOAc = 99:1) afforded *S*-phenyl cyclohexanethioate as a white solid (100.3 mg, 86%): ^1H NMR (400 MHz, $CDCl_3$) δ 7.41–7.38 (m, 5H), 2.63–2.57 (m, 1H), 2.02–1.97 (m, 2H), 1.84–1.80 (m, 2H), 1.69–1.66 (m, 1H), 1.65–1.47 (m, 2H), 1.36–1.21 (m, 3H); $^{13}C\{^1H\}$ NMR (100 MHz, $CDCl_3$) δ 200.7, 134.5, 129.1, 129.0, 127.9, 52.5, 29.5, 28.7, 25.5, 25.4, 25.3; MS (FAB-Magnetic Sector): *m/z* calcd for $C_{13}H_{17}OS$ (M^++H): 221.10; found: 221.10.

_S_-Phenyl 1-adamantanethioate (3na)[21]

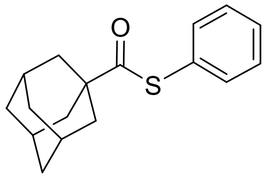

General procedure for the substrate of acyl fluoride derivatives was followed by 1-adamantanoyl fluoride (91.1 mg, 0.500 mmol). Silica gel column chromatography (hexane to hexane : EtOAc = 99:1) afforded *S*-phenyl 1-adamantanethioate as a white solid (108.7 mg, 79%): ^1H NMR (400 MHz, $CDCl_3$) δ 7.39–7.37 (m, 5H), 2.09 (s, 3H), 2.00 (m, 6H), 1.78–1.71 (m, 6H); $^{13}C\{^1H\}$ NMR (100 MHz, $CDCl_3$) δ 204.2, 135.0, 129.0, 129.0, 128.0, 49.0, 39.2, 36.4, 28.2; MS (FAB-Magnetic Sector): *m/z* calcd for $C_{17}H_{21}OS$ (M^++H): 273.13; found: 273.13.

_S_-Phenyl 1-phenyl-1-cyclopentanethioate (3oa)

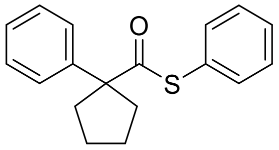

General procedure for the substrate of acyl fluoride derivatives was followed by 1-phenyl-1-cyclopentanoyl fluoride (96.1 mg, 0.500 mmol). Silica gel column chromatography (hexane to

hexane : EtOAc = 99:1) afforded *S*-phenyl 1-phenyl-1-cyclopentanethioate as a transparent solid (24.1 mg, 17%).

A 10-mL round-bottom flask was charged with 1-phenyl-1-cyclopentanecarbonyl fluoride (96.1 mg, 0.500 mmol) and Et_3N (2.5 mg, 0.025 mmol). The mixture was stirred at room temperature for 1 min. Column chromatography (hexane to hexane : EtOAc = 99:1) afforded *S*-phenyl 1-phenyl-1-cyclopentanethioate as a transparent solid (139.1 mg, 98%): m.p. 35.4–36.0 ˚C; ^{1}H NMR (400 MHz, $CDCl_3$) δ 7.45–7.43 (m, 2H), 7.38–7.26 (m, 8H), 2.74–2.68 (m, 2H), 2.09–2.02 (m, 2H), 1.80–1.75 (m, 4H); ^{13}C{^{1}H} NMR (100 MHz, $CDCl_3$) δ 201.9, 142.2, 134.7, 128.9, 128.4, 127.6, 127.3, 66.9, 36.5, 23.6; HRMS (FAB-Magnetic Sector): *m/z* calcd for $C_{18}H_{19}OS$ (M^++H): 283.1157; found: 283.1162.

S-Phenyl 1-phenyl-1-cyclopropanethioate (3pa)

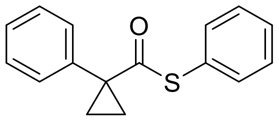

General procedure for the substrate of acyl fluoride derivatives was followed by 1-phenyl-1-cyclopropanoyl fluoride (82.1 mg, 0.500 mmol). Silica gel column chromatography (hexane to hexane : EtOAc = 99:1) afforded *S*-phenyl 1-phenyl-1-cyclopropanethioate as a white solid (96.4 mg, 76%): m.p. 91.7–92.7˚C; ^{1}H NMR (400 MHz, $CDCl_3$) δ 7.56–7.53 (m, 2H), 7.42–7.38 (m, 3H), 7.36–7.30 (m, 5H), 1.75 (q, J = 3.6 Hz, 2H), 1.30 (q, J = 3.6 Hz, 2H); ^{13}C{^{1}H} NMR (100 MHz, $CDCl_3$) δ 199.6, 138.2, 134.5, 132.3, 129.1, 129.0, 128.44, 128.37, 38.0, 19.3; HRMS (FAB-Magnetic Sector): *m/z* calcd for $C_{16}H_{15}OS$ (M^++H): 255.0844; found: 255.0824.

***S*-Phenyl *trans*-thiocinnamate (3qa)**[22]

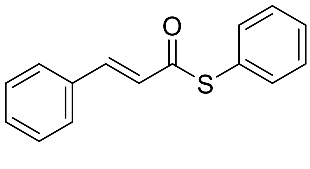

General procedure for the substrate of acyl fluoride derivatives was followed by cynnamoyl fluoride (75.1 mg, 0.500 mmol). Silica gel column chromatography (hexane to hexane : EtOAc = 99:1) afforded *S*-phenyl *trans*-thiocinnamate as a white solid (101.8 mg, 80%): ^{1}H NMR (400 MHz, $CDCl_3$) δ 7.68 (d, J = 15.6 Hz, 1H), 7.57–7.55 (m, 1H), 7.51–7.48 (m, 2H), 7.45–7.39 (m, 6H), 6.79 (d, J = 15.6 Hz, 1H); ^{13}C{^{1}H} NMR (100 MHz, $CDCl_3$) δ 188.0, 141.5, 134.6, 134.0, 130.7, 129.4, 129.2, 129.0, 128.5, 127.6, 124.1; MS (FAB-Magnetic Sector): *m/z* calcd for $C_{15}H_{13}OS$ (M^++H): 241.07; found: 241.07.

S-(4-Methylphenyl) 3,5-diemthylbenzothioate (3ab)[27]

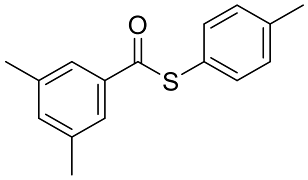

General procedure for the substrate of aryl thiosilane derivatives was followed by trimethyl(4-methylphenylthio)silane (101.8 mg, 0.5000 mmol). Silica gel column chromatography (hexane to hexane : EtOAc = 99:1) afforded *S*-(4-methylphenyl) 3,5-dimethylbenzothioate as a white solid (112.5 mg, 85%): ^{1}H NMR (400 MHz, $CDCl_3$) δ 7.62 (s, 2H), 7.39–7.36 (m, 2H), 7.26–7.24 (m, 2H), 7.21 (s, 1H), 2.39 (s, 3H), 2.37 (s, 6H); $^{13}C\{^1H\}$ NMR (100 MHz, $CDCl_3$) δ 190.7, 139.6, 138.4, 136.7, 135.2, 135.0, 130.0, 125.1, 124.0, 21.3, 21.2; MS (FAB-Magnetic Sector): *m/z* calcd for $C_{16}H_{17}OS$ (M^++H): 257.10; found: 257.10.

S-(4-Methoxyphenyl) 3,5-diemthylbenzothioate (3ac)[20]

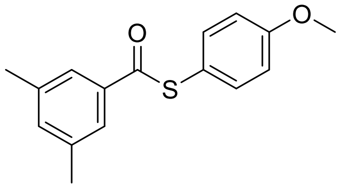

General procedure for the substrate of aryl thiosilane derivatives was followed by trimethyl(4-methoxyphenylthio)silane (108.4 mg, 0.5000 mmol). Silica gel column chromatography (hexane to hexane : EtOAc = 99:1) afforded *S*-(4-methoxyphenyl) 3,5--dimethylbenzothioate as a white solid (138.7 mg, 99%): ^{1}H NMR (400 MHz, $CDCl_3$) δ 7.62 (s, 2H), 7.42–7.39 (m, 2H), 7.22 (s, 1H), 7.00–6.97 (m, 2H), 3.84 (s, 3H), 2.38 (s, 6H); $^{13}C\{^1H\}$ NMR (100 MHz, $CDCl_3$) δ 191.2, 160.7, 138.4, 136.7, 136.6, 135.2, 125.1, 118.1, 114.9, 55.3, 21.2; MS (FAB-Magnetic Sector): *m/z* calcd for $C_{16}H_{17}O_2S$ (M^++H): 273.09; found: 273.08.

S-(3-Methylphenyl) 3,5-diemthylbenzothioate (3ad)

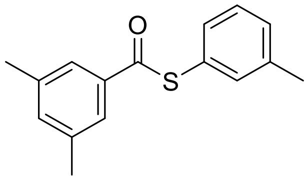

General procedure for the substrate of aryl thiosilane derivatives was followed by trimethyl(3-methylphenylthio)silane (100.1 mg, 0.5000 mmol). Silica gel column chromatography (hexane to hexane : EtOAc = 99:1) afforded *S*-(3-methylphenyl) 3,5-dimethylbenzothioate as a colorless oil (86.8 mg, 66%): ^{1}H NMR (400 MHz, $CDCl_3$) δ 7.62 (s, 2H), 7.34–7.29 (m, 3H), 7.23–7.20 (m, 2H), 2.37 (s, 3H), 2.36 (s, 6H); $^{13}C\{^1H\}$ NMR (100 MHz, $CDCl_3$) δ 190.4, 138.9, 138.4, 136.7, 135.5,

135.2, 132.0, 130.2, 129.0, 127.2, 125.1, 21.2, 21.1; HRMS (FAB-Magnetic Sector): *m/z* calcd for $C_{16}H_{17}OS$ (M^++H): 257.1000; found: 257.1007.

S-(4-Fluorophenyl) 3,5-diemthylbenzothioate (3ae)

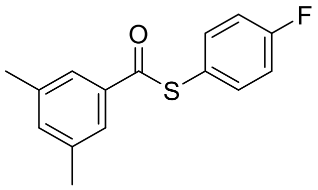

General procedure for the substrate of aryl thiosilane derivatives was followed by (4-fluorophenylthio)trimethylsilane (102.7 mg, 0.5000 mmol). Silica gel column chromatography (hexane to hexane : EtOAc = 99:1) afforded *S*-(4-fluorophenyl) 3,5-dimethylbenzothioate as a white solid (108.2 mg, 81%): m.p. 112.7–113.4 ˚C: ^{1}H NMR (400 MHz, $CDCl_3$) δ 7.61 (s, 2H), 7.49–7.44 (m, 2H), 7.23 (s, 1H), 7.17–7.11 (m, 2H), 2.38 (s, 6H); $^{13}C\{^1H\}$ NMR (100 MHz, $CDCl_3$) δ 190.3, 163.5 (d, $J_{C\text{-}F}$ = 249 Hz), 138.5, 137.1 (d, $J_{C\text{-}F}$ = 9.0 Hz), 136.4, 135.4, 125.2, 122.9 (d, $J_{C\text{-}F}$ = 4.0 Hz), 116.4 (d, $J_{C\text{-}F}$ = 22 Hz), 21.2; ^{19}F NMR (376 MHz, $CDCl_3$) δ -111.1; HRMS (FAB-Magnetic Sector): *m/z* calcd for $C_{15}H_{14}FOS$ (M^++H): 261.0749; found: 261.0748.

S-(4-Chlorophenyl) 3,5-diemthylbenzothioate (3af)

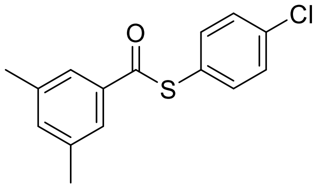

General procedure for the substrate of aryl thiosilane derivatives was followed by (4-chlorophenylthio)trimethylsilane (112.6 mg, 0.5000 mmol). Silica gel column chromatography (hexane to hexane : EtOAc = 99:1) afforded *S*-(4-chlorophenyl) 3,5-dimethylbenzothioate as a white solid (110.6 mg, 77%): m.p. 110.1–110.7 ˚C; ^{1}H NMR (400 MHz, $CDCl_3$) δ 7.61 (s, 2H), 7.42 (s, 4H), 7.24 (s, 1H), 2.38 (s, 6H); $^{13}C\{^1H\}$ NMR (100 MHz, $CDCl_3$) δ 189.8, 138.6, 136.4, 136.3, 135.8, 135.5, 129.4, 126.1, 125.2, 21.2; HRMS (FAB-Magnetic Sector): *m/z* calcd for $C_{15}H_{14}ClOS$ (M^++H): 277.0454; found: 277.0471.

S-(3-Fluorophenyl) 3,5-diemthylbenzothioate (3ag)

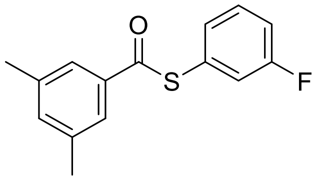

General procedure for the substrate of aryl thiosilane derivatives was followed by (3-fluorophenylthio)trimethylsilane (100.6 mg, 0.5000 mmol). Silica gel column chromatography (hexane to hexane : EtOAc = 99:1) to afford *S*-(3-fluorophenyl) 3,5-dimethylbenzothioate as a white solid (134.3 mg, 99%): m.p. 71.1–72.0 °C; ^{1}H NMR (400 MHz, $CDCl_3$) *δ* 7.61 (s, 2H), 7.47–7.44 (m, 2H), 7.22 (s, 1H), 7.12 (t, *J* = 8.8 Hz, 2H), 2.36 (d, *J* = 0.8 Hz, 6H); ^{13}C{^{1}H} NMR (100 MHz, $CDCl_3$) *δ* 190.2 (d, $J_{C\text{-}F}$ = 1.0 Hz), 163.5 (d, $J_{C\text{-}F}$ = 249 Hz), 138.5, 137.0 (d, $J_{C\text{-}F}$ = 9.0 Hz), 136.4, 135.4, 125.1, 122.8 (d, $J_{C\text{-}F}$ = 3.0 Hz), 116.4 (d, $J_{C\text{-}F}$ = 22 Hz), 21.1; ^{19}F NMR (376 MHz, $CDCl_3$) δ = -111.1; HRMS (FAB-Magnetic Sector): *m/z* calcd for $C_{15}H_{13}FOS$ (M^{+}+H): 261.0749; found: 261.0740.

_S_-(3-Chlorophenyl) 3,5-diemthylbenzothioate (3ah)

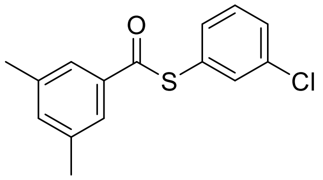

General procedure for the substrate of aryl thiosilane derivatives was followed by (3-chlorophenylthio)trimethylsilane (108.4 mg, 0.5000 mmol). Silica gel column chromatography (hexane to hexane : EtOAc = 99:1) to afford *S*-(3-chlorophenyl) 3,5-dimethylbenzothioate as a white solid (107.2 mg, 86%): m.p. 74.8–75.5 °C: ^{1}H NMR (400 MHz, $CDCl_3$) *δ* 7.61 (s, 2H), 7.52–7.51 (m, 1H), 7.43–7.37 (m, 3H), 7.24 (s, 1H), 2.38 (d, *J* = 0.8 Hz, 6H); ^{13}C{^{1}H} NMR (100 MHz, $CDCl_3$) *δ* 189.5, 138.6, 136.3, 135.6, 134.72, 134.66, 133.2, 130.1, 129.6, 129.4, 125.2, 21.2; HRMS (FAB-Magnetic Sector): *m/z* calcd for $C_{15}H_{14}ClOS$ (M^{+}+H): 277.0448; found: 277.0458.

_S_-(3,5-Dimethylphenyl) 3,5-diemthylbenzothioate (3ai)

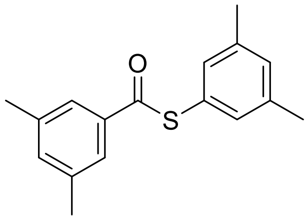

General procedure for the substrate of aryl thiosilane derivatives was followed by trimethyl(3,5-dimethylphenylthio)silane (106.2 mg, 0.5000 mmol). Silica gel column chromatography (hexane to hexane : EtOAc = 99:1) afforded *S*-(3,5-dimethylphenyl) 3,5-dimethylbenzothioate as a white solid (107.2 mg, 79%): m.p. 86.7–87.5 °C: ^{1}H NMR (400 MHz, $CDCl_3$) *δ* 7.62 (s, 2H), 7.20 (s,1H), 7.12 (s, 2H), 7.05 (s, 1H), 2.36 (s, 6H), 2.34 (s, 6H); ^{13}C{^{1}H} NMR (100 MHz, $CDCl_3$) *δ* 190.7, 138.8, 138.3, 136.8, 135.1, 132.6, 131.3, 126.8, 125.1, 21.1; HRMS (FAB-Magnetic Sector): *m/z* calcd for $C_{17}H_{19}OS$ (M^{+}+H): 271.1157; found: 271.1154.

_S_-(2,4-Dimethylphenyl) 3,5-diemthylbenzothioate (3aj)

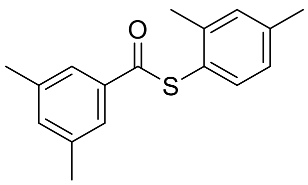

General procedure for the substrate of aryl thiosilane derivatives was followed by trimethyl(2,4-dimethylphenylthio)silane (106.7 mg, 0.5000 mmol). Silica gel column chromatography (hexane to hexane : EtOAc = 99:1) afforded *S*-(2,4-dimethylphenyl) 3,5-dimethylbenzothioate as a white solid (125.6 mg, 92%): m.p. 49.6–50.2 ˚C; ^{1}H NMR (400 MHz, $CDCl_3$) δ 7.65 (s, 2H), 7.34 (d, J = 7.6 Hz, 1H), 7.21–7.20 (m, 1H), 7.16 (s, 1H), 7.07–7.04 (m, 1H), 2.37 (d, *J* = 0.8 Hz, 6H), 2.35 (s, 3H), 2.34 (s, 3H); ^{13}C{^{1}H} NMR (100 MHz, $CDCl_3$) δ 190.1, 142.3, 140.2, 138.4, 136.8, 136.2, 135.1, 131.6, 127.4, 125.2, 123.5, 21.2, 21.1, 20.6; HRMS (FAB-Magnetic Sector): *m/z* calcd for $C_{17}H_{19}OS$ (M^{+}+H): 271.1151; found: 271.1158.

_S_-(1-Decyl) 3,5-diemthylbenzothioate (3ak)

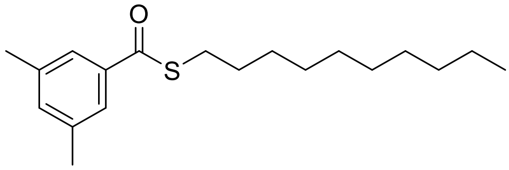

General procedure for the substrate of aliphatic thiosilane derivatives was followed by (1-decylthio)trimethylsilane (124.9 mg, 0.5000 mmol). Silica gel column chromatography (hexane to hexane : EtOAc = 99 : 1) afforded *S*-(1-decyl) 3,5-dimethylbenzothioate as a colorless oil (145.6 mg, 98%): ^{1}H NMR (400 MHz, $CDCl_3$) δ 7.57 (s, 2H), 7.17 (s, 1H), 3.04 (t, *J* = 7.2 Hz, 2H), 2.35 (s, 6H), 1.69–1.61 (m, 2H), 1.45–1.23 (m, 14H), 0.88 (t, *J* = 7.2 Hz, 3H); ^{13}C{^{1}H} NMR (100 MHz, $CDCl_3$) δ 192.4, 138.2, 137.3, 134.8, 124.9, 31.9, 29.6, 29.53, 29.49, 29.3, 29.2, 29.0, 28.9, 22.7, 21.2, 14.1; HRMS (FAB-Magnetic Sector): *m/z* calcd for $C_{19}H_{31}OS$ (M^{+}+H): 307.2096; found: 307.2095.

_S_-(_n_-Butyl) 3,5-diemthylbenzothioate (3al)[28]

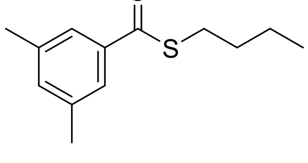

General procedure for the substrate of aliphatic thiosilane derivatives was followed by (1-butylthio)trimethylsilane (81.2 mg, 0.500 mmol). Silica gel column chromatography (hexane : EtOAc = 99:1) afforded *S*-(*n*-butyl) 3,5-dimethylbenzothioate as a colorless oil (42.7 mg, 37%): ^{1}H NMR (400 MHz, $CDCl_3$) δ 7.58 (s, 2H), 7.19 (s, 1H), 3.06 (t, *J* = 7.2 Hz, 2H), 2.36 (s, 6H), 1.65 (quin, *J* =

7.6 Hz, 2H), 1.45 (sex, J = 7.6 Hz, 2H), 0.95 (t, J = 7.2 Hz, 3H); $^{13}C\{^1H\}$ NMR (100 MHz, $CDCl_3$) δ 192.4, 138.2, 137.3, 134.8, 124.9, 31.6, 28.7, 22.0, 21.2, 13.6; MS (FAB-Magnetic Sector): *m/z* calcd for $C_{18}H_{19}OS$ (M^++H): 223.12; found: 223.12.

S-(t-Butyl) 3,5-diemthylbenzothioate (3am)

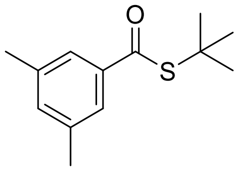

General procedure for the substrate of aliphatic thiosilane derivatives was followed by (*t*-butylthio)trimethylsilane (81.2 mg, 0.500 mmol). Silica gel column chromatography (hexane : EtOAc = 99:1) afforded *S*-(*t*-butyl) 3,5-dimethylbenzothioate as a colorless oil (34.2 mg, 31%): ^1H NMR (400 MHz, $CDCl_3$) δ 7.53 (s, 2H), 7.16 (s, 1H), 2.34 (s, 6H), 1.57 (s, 9H); $^{13}C\{^1H\}$ NMR (100 MHz, $CDCl_3$) δ 193.1, 138.3, 138.1, 134.5, 124.6, 47.9, 29.9, 21.2; HRMS (FAB-Magnetic Sector): *m/z* calcd for $C_{13}H_{19}OS$ (M^++H): 223.1157; found: 223.1152.

Methyl 3-(3,5-dimethylbenzothio)propanoate (3an)

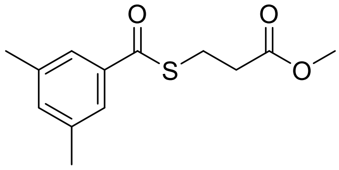

General procedure for the substrate of aliphatic thiosilane derivatives was followed by methyl(3-trimethylsilyl)propanoate (93.2 mg, 0.500 mmol). Silica gel column chromatography (hexane : EtOAc = 99:1) afforded methyl 3-(3,5-dimethylbenzothio)propanoate as a colorless oil (119.7 mg, 98%): ^1H NMR (400 MHz, $CDCl_3$) δ 7.55 (s, 2H), 7.18 (s, 1H), 3.70 (s, 3H), 3.29 (t, J = 6.8 Hz, 2H), 2.72 (t, J = 6.8 Hz, 2H), 2.34 (s, 6H); $^{13}C\{^1H\}$ NMR (100 MHz, $CDCl_3$) δ 191.4, 172.0, 138.1, 136.7, 135.0, 124.8, 51.6, 34.1, 23.8, 21.0; HRMS (FAB-Magnetic Sector): *m/z* calcd for $C_{13}H_{17}O_3S$ (M^++H): 253.0898; found: 253.0890.

S-Furfuryl 3,5-diemthylbenzothioate (3ao)

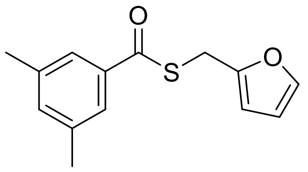

General procedure for the substrate of aliphatic thiosilane derivatives was followed by (furfurylthio)trimethylsilane (90.2 mg, 0.500 mmol). Silica gel column chromatography (hexane :

EtOAc = 99:1) afforded *S*-furfuryl 3,5-dimethylbenzothioate as a colorless oil (116.5 mg, 98%): ^{1}H NMR (400 MHz, $CDCl_3$) δ 7.56 (s, 2H), 7.33 (dd, J = 2.0, 0.8 Hz, 1H), 7.18 (s, 1H), 6.30–6.27 (m, 2H), 4.32 (s, 2H), 2.34 (d, J = 0.8 Hz, 6H); ^{13}C{^{1}H} NMR (100 MHz, $CDCl_3$) δ 190.8, 150.5, 142.1, 138.3, 136.6, 135.1, 125.0, 110.6, 108.0, 25.6, 21.1; HRMS (FAB-Magnetic Sector): *m/z* calcd for $C_{14}H_{15}O_2S$ (M^++H): 247.0793; found: 247.0800.

11. DFT Calculation

To clarify the difference in the results between **1o** and **1p**, a structural optimization was carried out by DFT calculations (Figure S1 and S2). The conditions are as follows: DFT/B3LYP/6-31G, Gibbs free energy, 293.15 K, kcal mol^{-1}.

1-Phenyl-1-cyclopentanecarbonyl fluoride (**1o**): −17410.98 eV

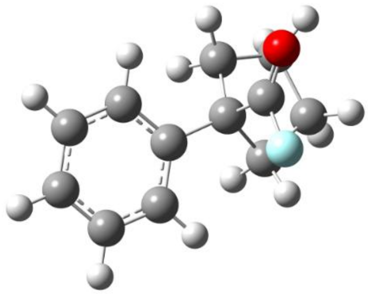

Figure S1. Optimal Structure of 1-Phenyl-1-cyclopentanecarbonyl Fluoride (**1o**)

1-Phenyl-1-cyclopropanecarbonyl fluoride (**1p**): −15272.65 eV

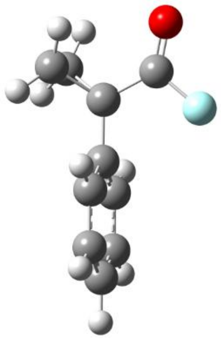

Figure S2. Optimal Structure of 1-Phenyl-1-cyclopropanecarbonyl Fluoride (**1p**)

12. Cartesian Coordinates of Acyl Fluoride 1o and 1p

Table S6. Atomic cartesian coordinates of acyl fluoride **1o**

	Coordinates		
	X	Y	Z
C	3.009530	-0.598327	-1.046118
C	1.611173	-0.560808	-1.080185
C	0.877675	-0.179232	0.054918
C	1.578877	0.188748	1.217825
C	2.976203	0.153322	1.251370
C	3.696854	-0.243905	0.119415
H	3.559361	-0.900119	-1.931977
H	1.095852	-0.825398	-1.996009
H	1.033299	0.504646	2.101017
H	3.499487	0.437613	2.158807
H	4.781471	-0.271843	0.144236
C	-1.063945	1.324708	-0.150826
O	-1.627240	2.085113	0.604914
F	-0.683191	1.791736	-1.430612
C	-0.658040	-0.128110	0.042923
C	-1.324233	-0.675554	1.338390
C	-1.339397	-0.994012	-1.062192
C	-2.788496	-1.018503	0.949668
H	-0.777132	-1.576494	1.636966
H	-1.273910	0.039004	2.163549
C	-2.812733	-1.121626	-0.608584
H	-1.235857	-0.562101	-2.060508
H	-0.848756	-1.974634	-1.063711

H	-3.106512	-1.953276	1.422885
H	-3.471255	-0.235481	1.294281
H	-3.413020	-0.309259	-1.035969
H	-3.257206	-2.059386	-0.956998

Table S7. Atomic cartesian coordinates of acyl fluoride **1p**

	Coordinates		
	X	Y	Z
C	2.562806	-0.091830	1.209725
C	1.176924	0.091830	1.210450
C	0.470855	0.211187	0.002116
C	1.178891	0.135335	-1.208141
C	2.564811	-0.052603	-1.211180
C	3.260237	-0.165923	-0.001688
H	3.095801	-0.180061	2.151080
H	0.637370	0.151437	2.151252
H	0.640973	0.221280	-2.147589
H	3.099381	-0.110353	-2.154009
H	4.335889	-0.310359	-0.003151
C	-1.882337	-0.801210	-0.009566
O	-3.097158	-0.858481	-0.012246
F	-1.141470	-1.996453	-0.019867
C	-1.020268	0.403435	0.004158
C	-1.634774	1.616599	-0.729604
C	-1.634967	1.599299	0.766233
H	-2.574617	1.449890	-1.243360
H	-0.931166	2.260383	-1.245086

H	-0.931279	2.230613	1.296840
H	-2.574916	1.420685	1.275763

13. Reference

[1] Ishikawa, N.; Kitazumi, T.; Yamazaki, T.; Mochida, Y.; Taruto, T. Enhanced Effect of Spray-Dried Potassium Fluoride on Fluorination. *Chem. Lett.* **1981**, 761–764.

[2] Tryniszewski, M.; Barbasiewicz, M. Gram-Scale Preparation of Acyl Fluorides and Their Reactions with Hindered Nucleophiles. *Synthesis* **2022**, *54*, 1446–1460.

[3] Chaudhari, S. S.; Akamanchi, K. G. Thionyl Chloride-Benzotriazole in Methylene Chloride: A Convenient Solution for Conversion of Alcohols and Carboxylic Acids Expeditiously into Alkyl Chlorides and Acid Chlorides by Simple Titration. *Synlett* **1999**, 1763–1765.

[4] Cappozzi, G.; Menichetti, S.; Rosi, A. Silicon in Organosulfur Chemistry, Part 3. Disulfide-Silyl Sulfide Interchange. A New Aspect of the Thiol-Disulfide Interchange. *J. Chem. Soc. Perkin Trans 2* **1992**, 2247–2251.

[5] Wu, F. -W.; Mao, Y. -J.; Pu, J.; Li, H. -L.; Ye, P.; Xu, Z. -Y.; Lou, S.-J.; Xu, D. -Q. Ni-Catalysed Deamidative Fluorination of Amides with Electrophilic Fluorinating Reagents. *Org. Biomol. Chem.* **2022**, *20*, 4091–4095.

[6] Morgan, P. J.; Saunders, G. C.; Macgregor, S. A.; Marr, A. C.; Lincence, P. Nucleophilic Fluorination Catalyzed by a Cyclometallated Rhodium Complex. *Organometallics* **2022**, *41*, 883–891.

[7] Song, H. -X.; Tian, Z. -Y.; Xiao, J. -C.; Zhang, C. -P. Tertiary-Amine-Initiated Synthesis of Acyl Fluorides from Carboxylic Acids and $CF_3SO_2OCF_3$. *Chem. Eur. J.* **2020**, *26*, 16261–16265.

[8] Meng, Q. -Y.; Döben, N.; Studer, A. Cooperative NHC and Photoredox Catalysis for the Synthesis of *β*-Trifluoromethylated Alkyl Aryl Ketones. *Angew. Chem. Int. Ed.* **2000**, *59*, 19956–19960.

[9] Arisawa, M.; Yamada, T.; Yamaguchi, M. Synthesis of Acylphosphine Sulfides by Rhodium-Catalyzed Reaction of Acid Fluorides and Diphosphine Disulfides. *Tetrahedron Lett.* **2010**, *51*, 4957–4958.

[10] Scattolin, T.; Deckers, K.; Schonebeck, F. Direst Synthesis of Acyl Fluorides from Carboxylic Acids with the Bench-Stable Solid Reagent $(Me_4N)SCF_3$. *Org. Lett.* **2017**, *19*, 5740–5743.

[12] Stanek, K.; Koller, R.; Togni, A. Reactivity of a 10-I-3 Hypervalent Iodine Trifluoromethylation Reagent With Phenols. *J. Org. Chem.* **2008**, *73*, 7678–7685.

[13] Zhao, S.; Guo, Y.; Su, Z.; Wu, C.; Chen, W.; Chen, Q. Y. Deoxyfluorination of Carboxylic, Sulfonic, Phosphinic Acids and Phosphine Oxides by Perfluoroalkyl Ether Carboxylic Acids Featuring CF_2O Units. *Chin. I. Chem.* **2021**, *39*, 1225–1232.

[14] Polterauer, D.; Hanselmann, P.; Littich, R.; Bersier, M.; Roberge, D. M.; Wagschal, S.; Hone, C. A.; Kappe, C. O. Sulfur Tetrafluoride (SF_4) as a Deoxyfluorination Reagent for Organic Synthesis in Continuous Flow Mode. *Org. Process Res. Dev.* **2023**, *27*, 2385–2392.

[15] Gonay, M.; Batisse, C.; Paquin, J. -F. Synthesis of Acyl Fluorides from Carboxylic Acids Using NaF-Assisted Deoxyfluorination with XtalFluor-E. *J. Org. Chem.* **2020**, *85*, 10253–10260.

[16] Mathieu-Pelta, I.; Evans, S. A. Highly Regioselective and Stereospecific Functionalization of 1,2-Propanediol with Trimethyl(X)silanes Employing the 1,3,2λ^5-Dioxaphospholane Methodology. *J. Org. Chem.* **1992**, *57*, 3409–3413.

[17] Zho, H.; Irran, E.; Klare, H. F. T.; Oestreich, M. Electrophilic Activation of S-Si Reagents Silylium Ions for Their Regio- and Diastereoselective Addition Across C-C Multiple Bonds. *Angew. Chem. Int. Ed.* **2024**, *63*, e202401599.

[18] Nishimoto, Y.; Okita, A.; Yasuda, M.; Baba, A. Synthesis of a Wide Range of Thioethers by Indium Triiodide-Catalyzed Direct Coupling between Alkyl Acetates and Thiosilanes. *Org. Lett.* **2012**, *14*, 1846–1849.

[19] Mesgar, M.; Nguyen-Le, J.; Daugulis, O. New Hindered Amide Base for Aryne Insertion into Si-P, Si-S, Si-N, and C-C Bonds. *J. Am. Chem. Soc.* **2018**, *140*, 13703–13710.

[20] Lechuga-Eduardo, H.; Zarza-Acuña, E.; Romero-Ortega, M. Synthesis of 3-Substituted 2-Cyclohexenones through umpoled Functionalization. *Tetrahedron Lett.* **2017**, *58*, 3234–3237.

[21] Badsara, S. S.; Liu, Y. -C.; Hsieh, P. -A.; Zeng, J. -W.; Lu, S. -Y.; Liu, Y. -W.; Lee, C. -F. Metal-Free sp^3 C-H Functionalization: A Novel Approach for the Synthesis of Selenide Ethers and Thioesters from Methyl Arenes. *Chem. Commun.* **2014**, *50*, 11374–11377.

[22] Xu, j.; Lu, F.; Sun, L.; Huang, M.; Jiang, J.; Wang, K.; Ouyang, D.; Lu, L.; Lei, A. Electrochemical Reductive Cross-Coupling of Acyl Chlorides and Sulfinic Acids towards the Synthesis of Thioesters. *Green Chem.* **2022**, *24*, 7350–7354.

[23] Zhu, F.; Rodriguez, J.; O'Neill, S.; Walczak, M. A. Acyl Glycosides through Stereospecific Glycosyl Cross-Coupling: Rapid Access to C(sp^3)-Linked Glycomimetic. *ACS Cent. Sci.* **2018**, *4*, 1652–1662.

[24] Xiao, Y. M.; Zhao, Y.; Li, J. -Q.; Yuan, J. -W.; Yang, L. -R.; Mao, P.; Mai, W. P. Palladium-Catalyzed Convenient Synthesis of Thioesters from Carboxylic Acids and Disulfides. *New J. Chem.* **2023**, *47*, 17092–17097.

[25] Lin, S. -M.; Zhang, J. -L.; Chen, J. -X.; Gao, W. -X.; Ding, J. -C.; Su, W. -K.; Wu, H. -Y. An Approach to the Synthesis of Thioesters and Selenoesters Promoted by Rongalite. *J. Braz. Chem. Soc.*

2010, *21*, 1616–1620.

[26] Ai, H. -J.; Rabeah, J.; Brückner. A.; Wu, X. -F. Rhodium-Catalyzed Carbonylative Coupling of Alkyl Halides with Thiols: A Radical Process faster than easier Nucleophilic Substitution. *Chem. Commun.* **2021**, *57*, 1466–1469.

[27] Movassagh, B.; Balalaie, S.; Shaygan, P. A New and Efficient Protocol for Preparation of Thiol Esters from Carboxylic Acids and Thiols in the Presence of 2-(1H-Benzotriazole-1-yl)-1,1,3,3-tetramethyluronium Tetrafluoroborate (TBTU). *Arkivoc* **2007**, *13*, 47–52.

[28] Zhao, B.; Fu, Y.; Shang, R. Oxalic Acid Monothioester for Palladium-Catalyzed Decarboxylative Thiocarbonylation and Hydrothiocarbonylation. *Org. Lett.* **2019**, *21*, 9521–9526.

[29] Wang, H.; Li, Y.; Sun, Y.; Li, Y. Palladium-Catalyzed Thiocarbonylation of Aryl Iodides Using CO_2. *J. Org. Chem.* **2023**, *88*, 8835–8842.

13. Spectra Chart

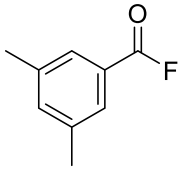

1a

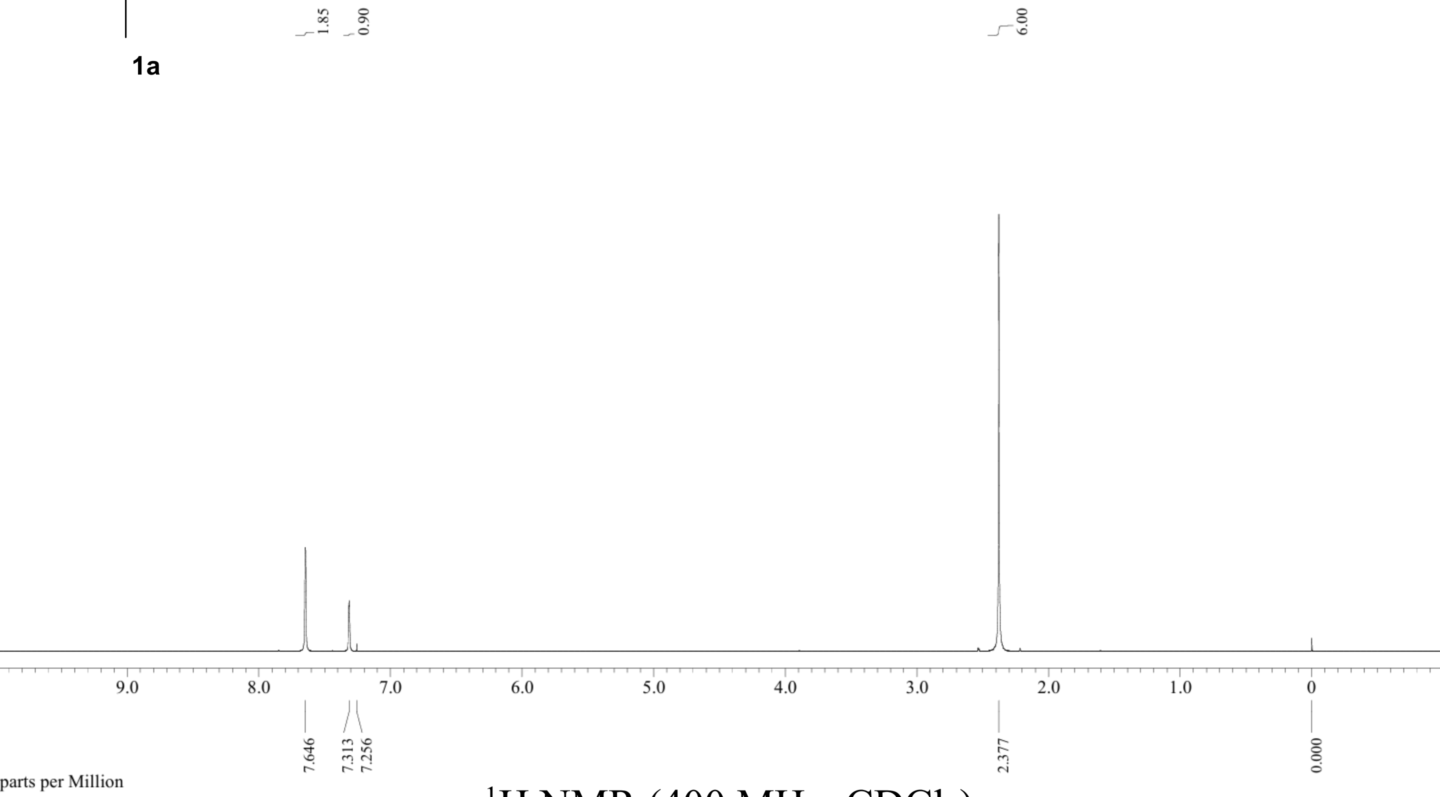

^{1}H NMR (400 MHz, $CDCl_3$)

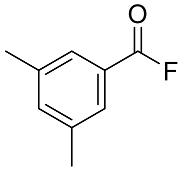

1a

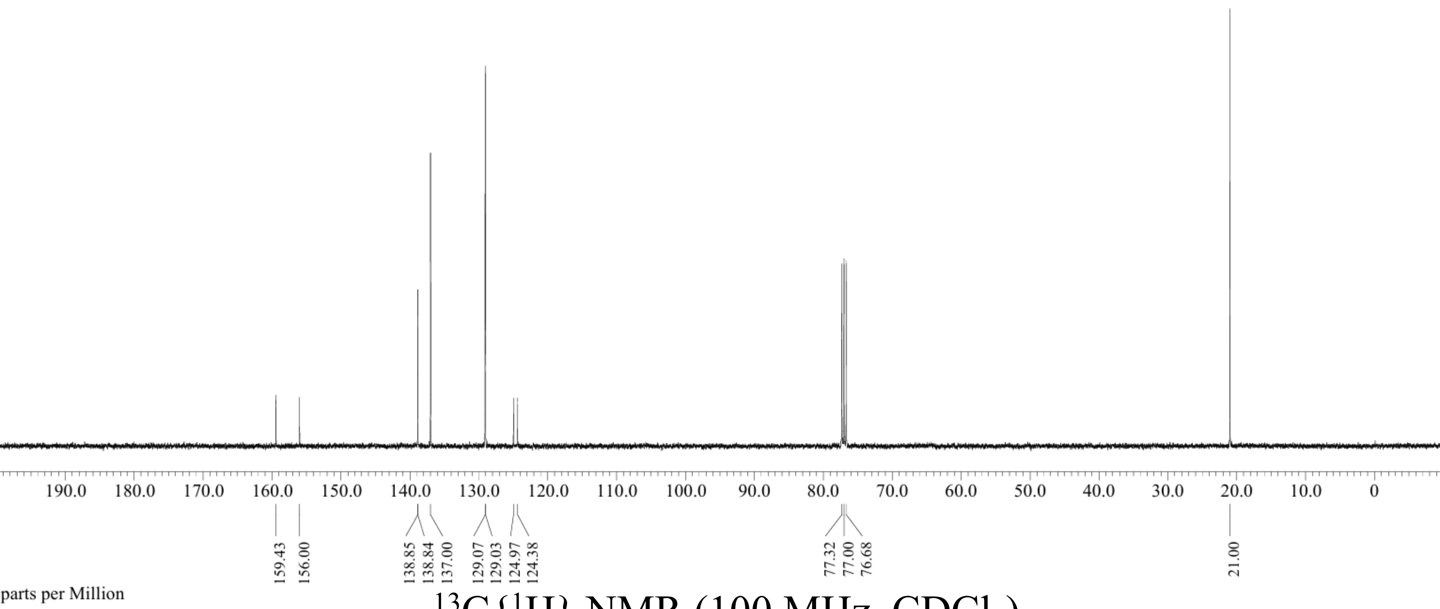

^{13}C{^{1}H} NMR (100 MHz, $CDCl_3$)

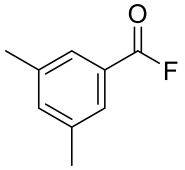

1a

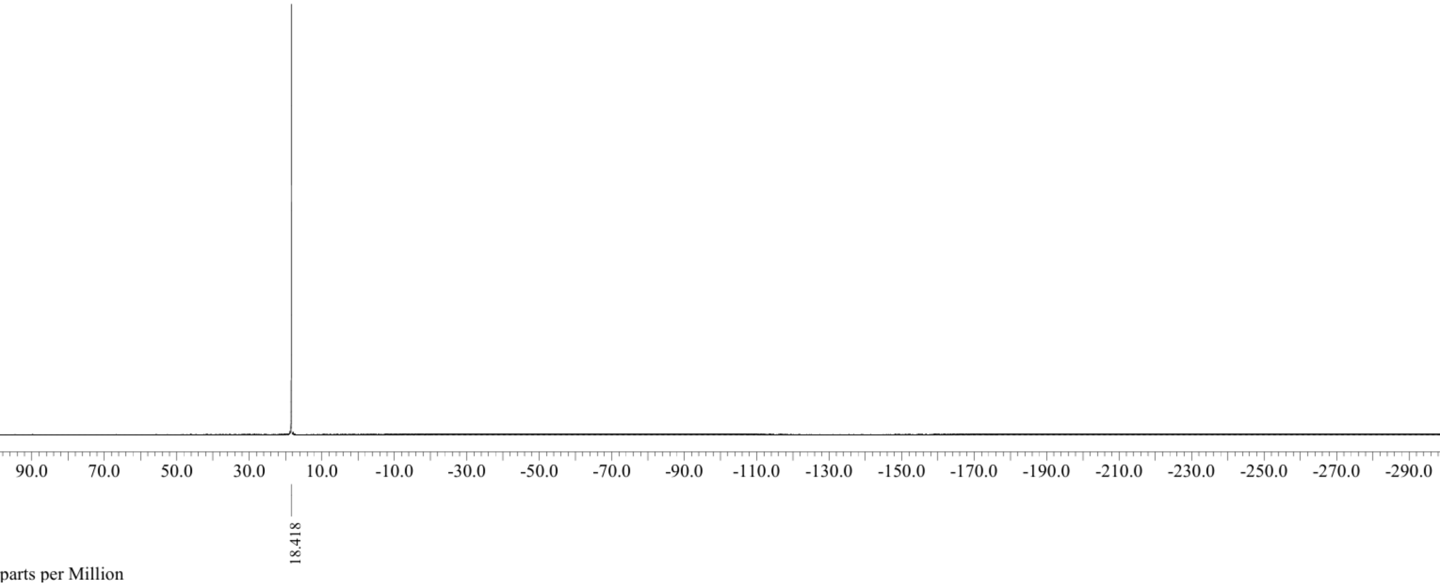

^{19}F NMR (376 MHz, $CDCl_3$)

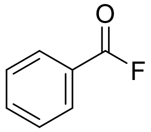

1b

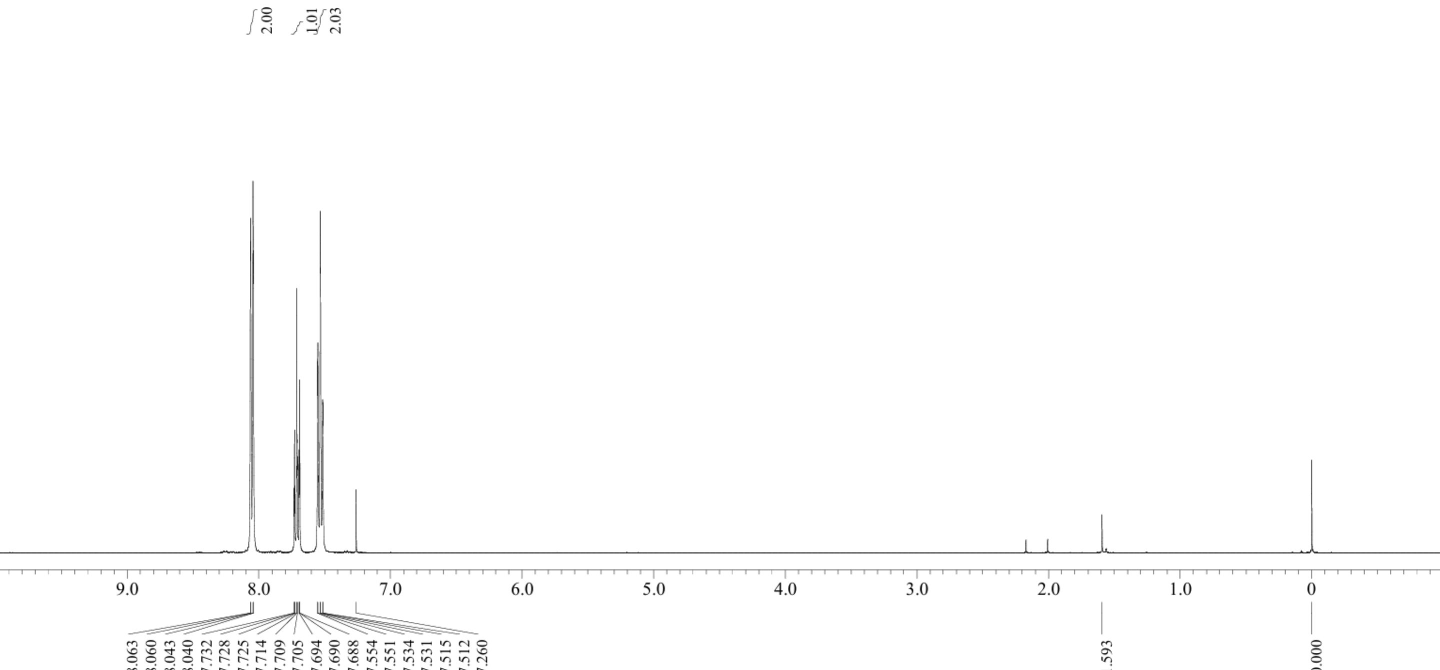

^{1}H NMR (400 MHz, $CDCl_3$)

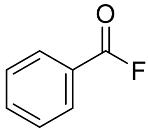

1b

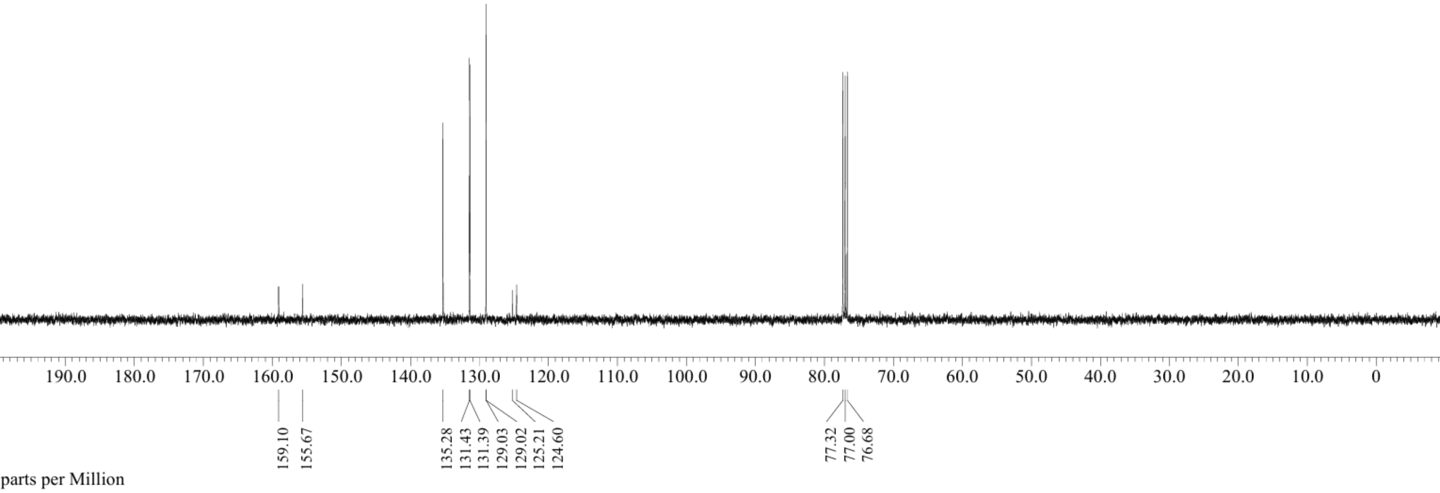

$^{13}C\{^{1}H\}$ NMR (100 MHz, $CDCl_3$)

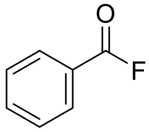

1b

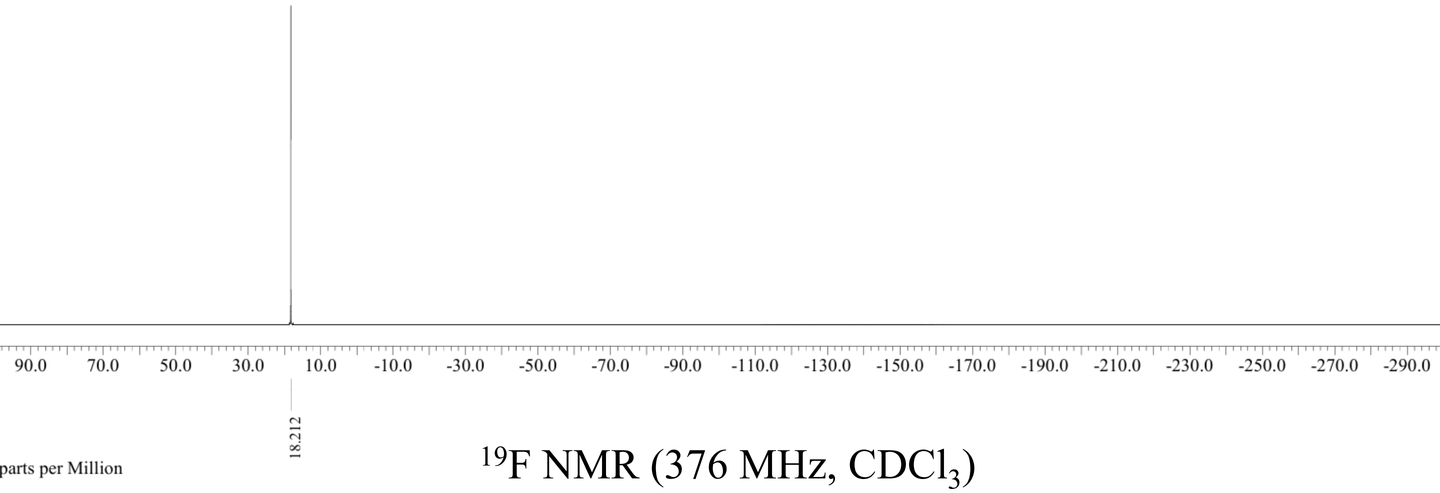

^{19}F NMR (376 MHz, $CDCl_3$)

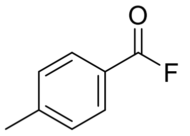

1c

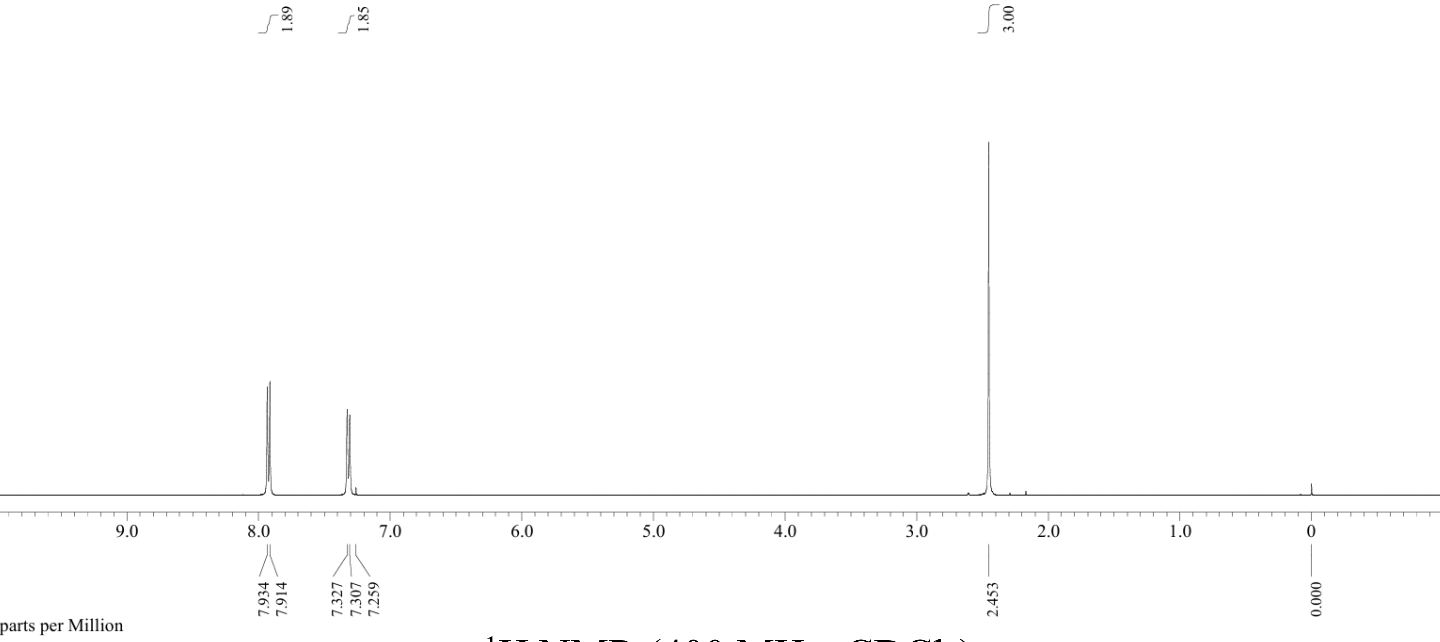

^{1}H NMR (400 MHz, $CDCl_3$)

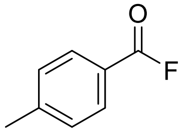

1c

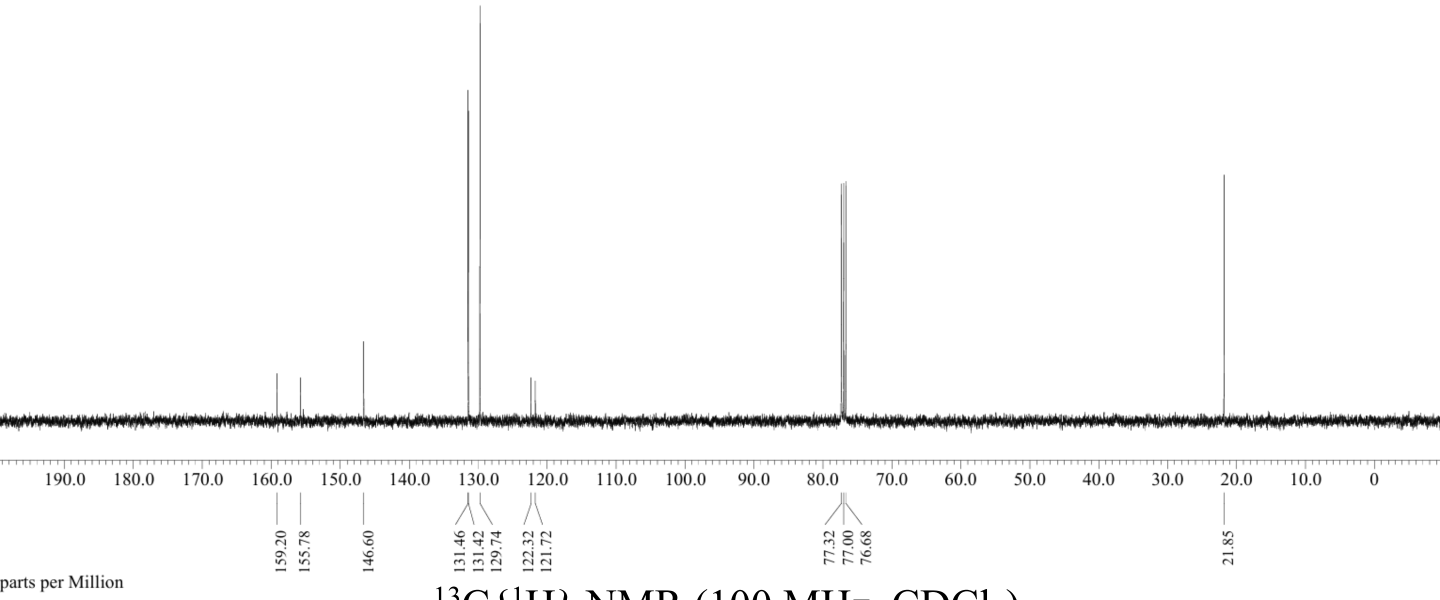

$^{13}C\{^{1}H\}$ NMR (100 MHz, $CDCl_3$)

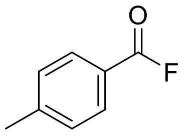

1c

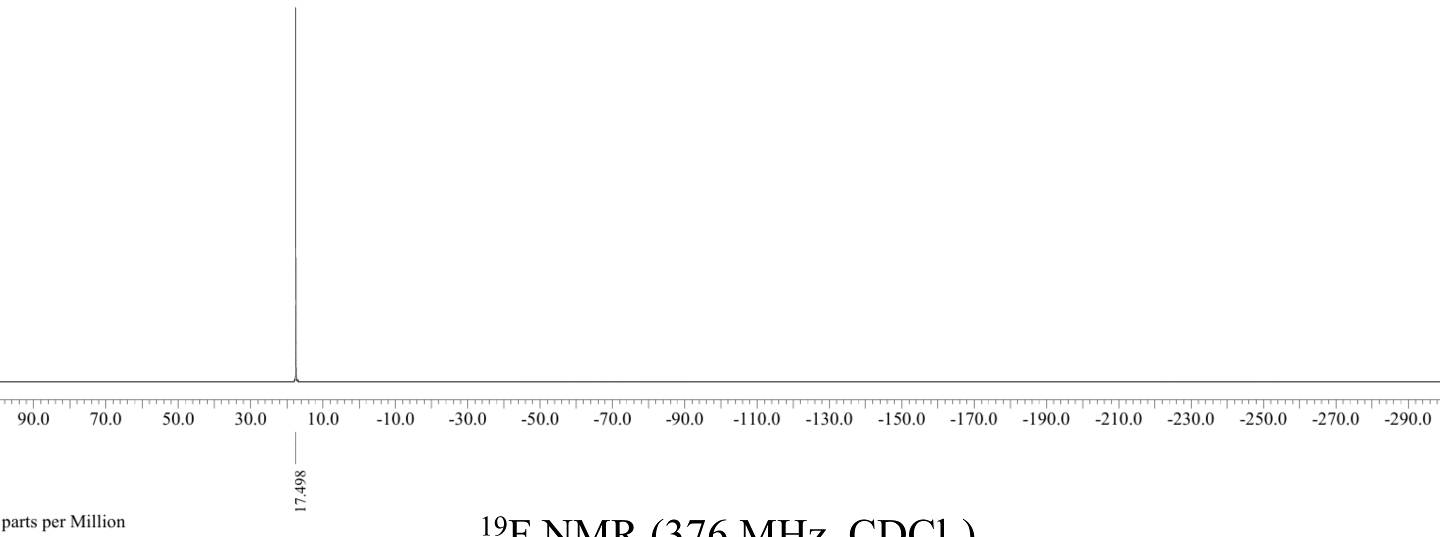

^{19}F NMR (376 MHz, $CDCl_3$)

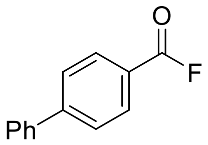

1d

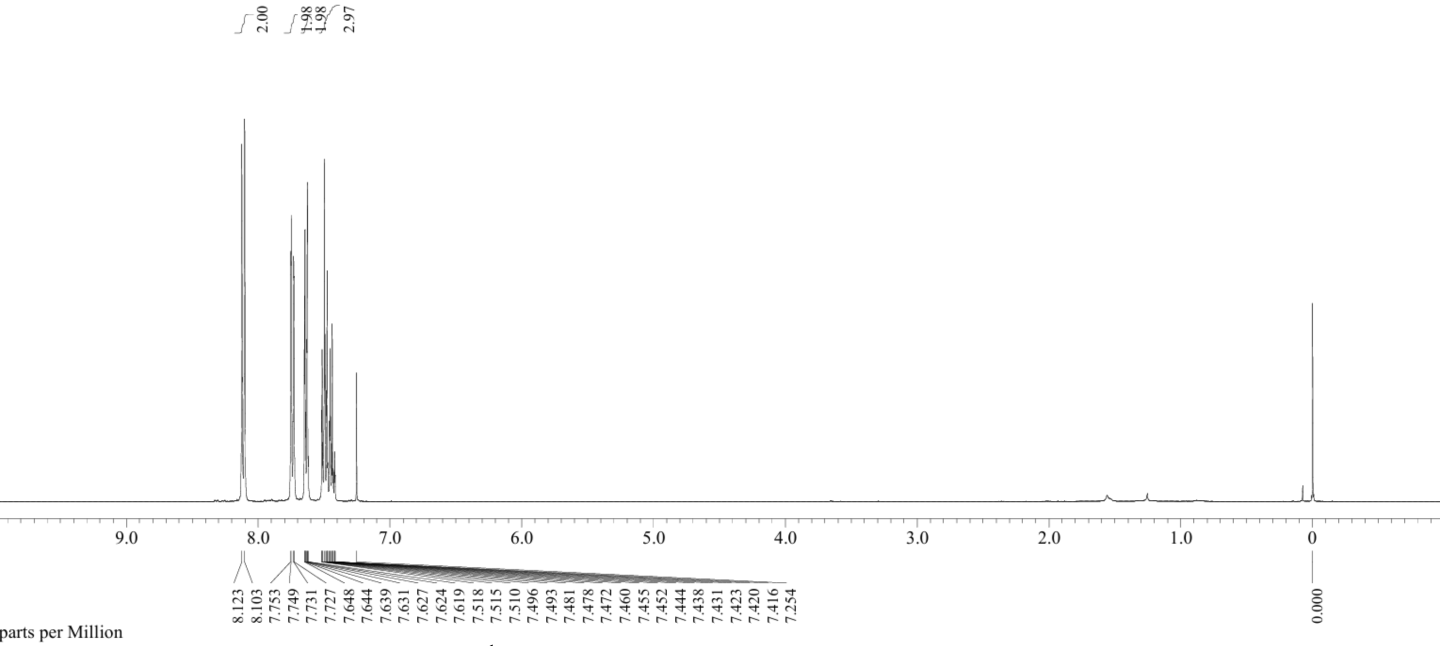

^{1}H NMR (400 MHz, $CDCl_3$)

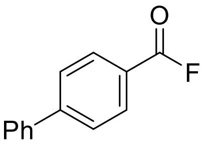

1d

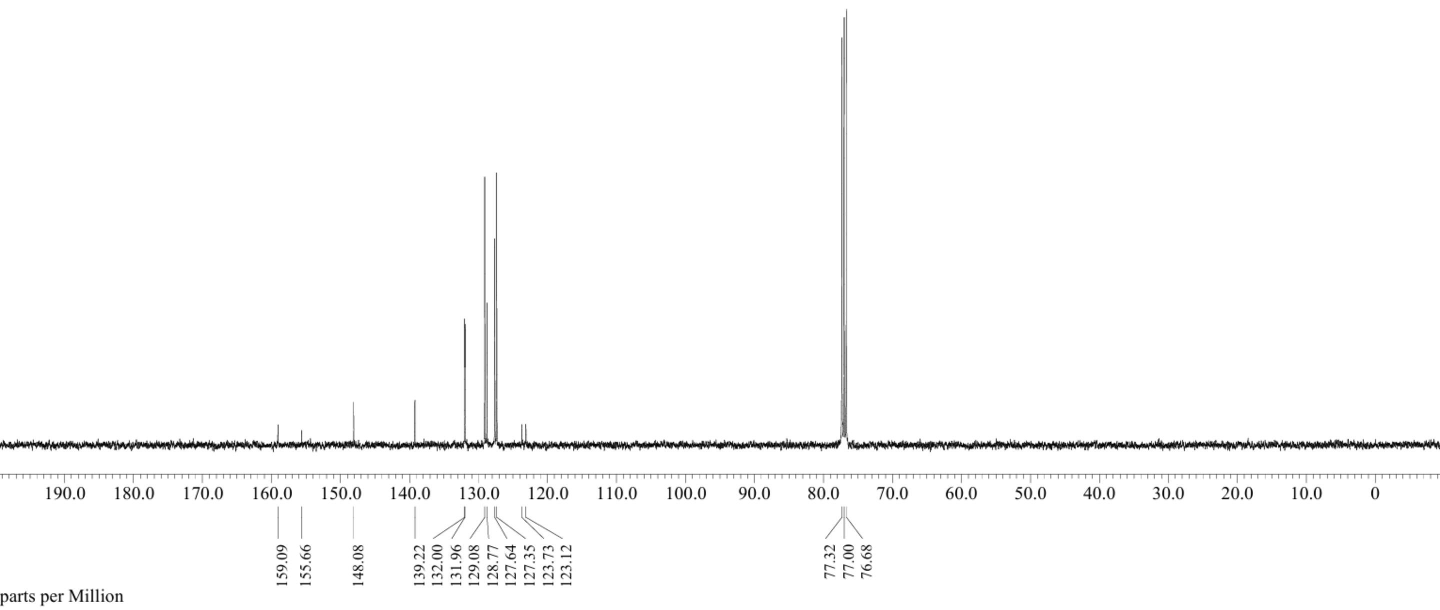

$^{13}C\{^{1}H\}$ NMR (100 MHz, $CDCl_3$)

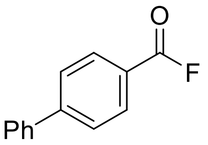

1d

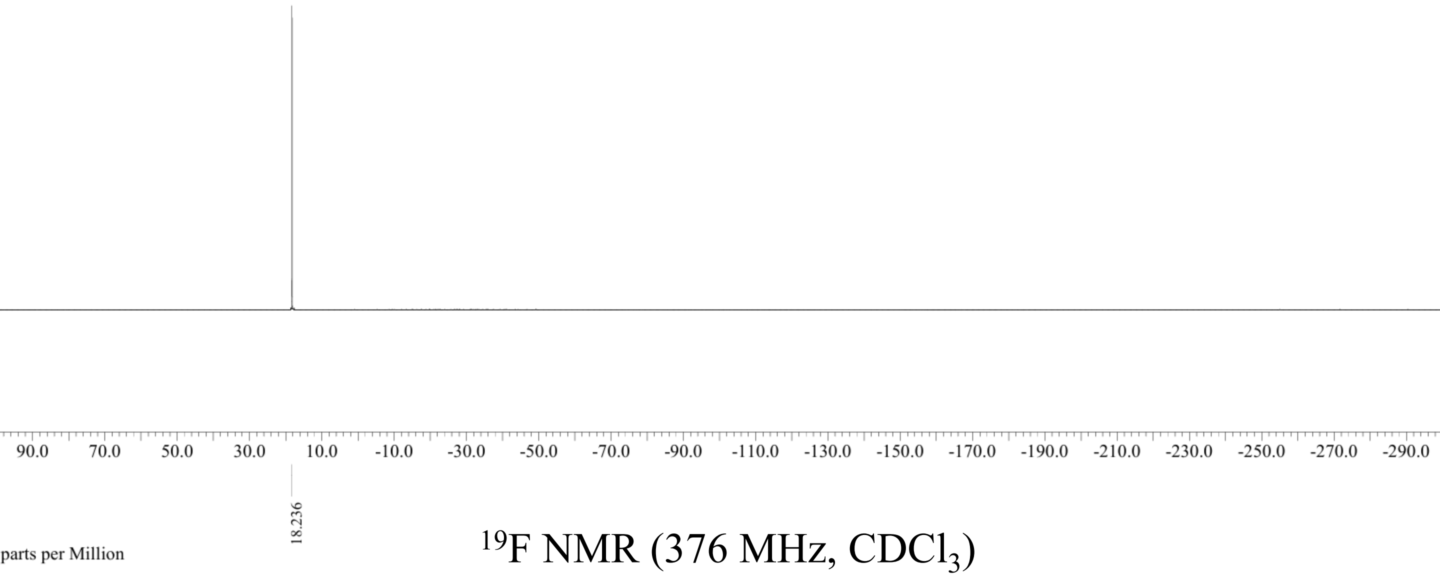

^{19}F NMR (376 MHz, $CDCl_3$)

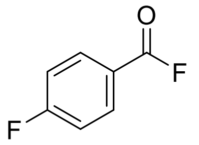

1e

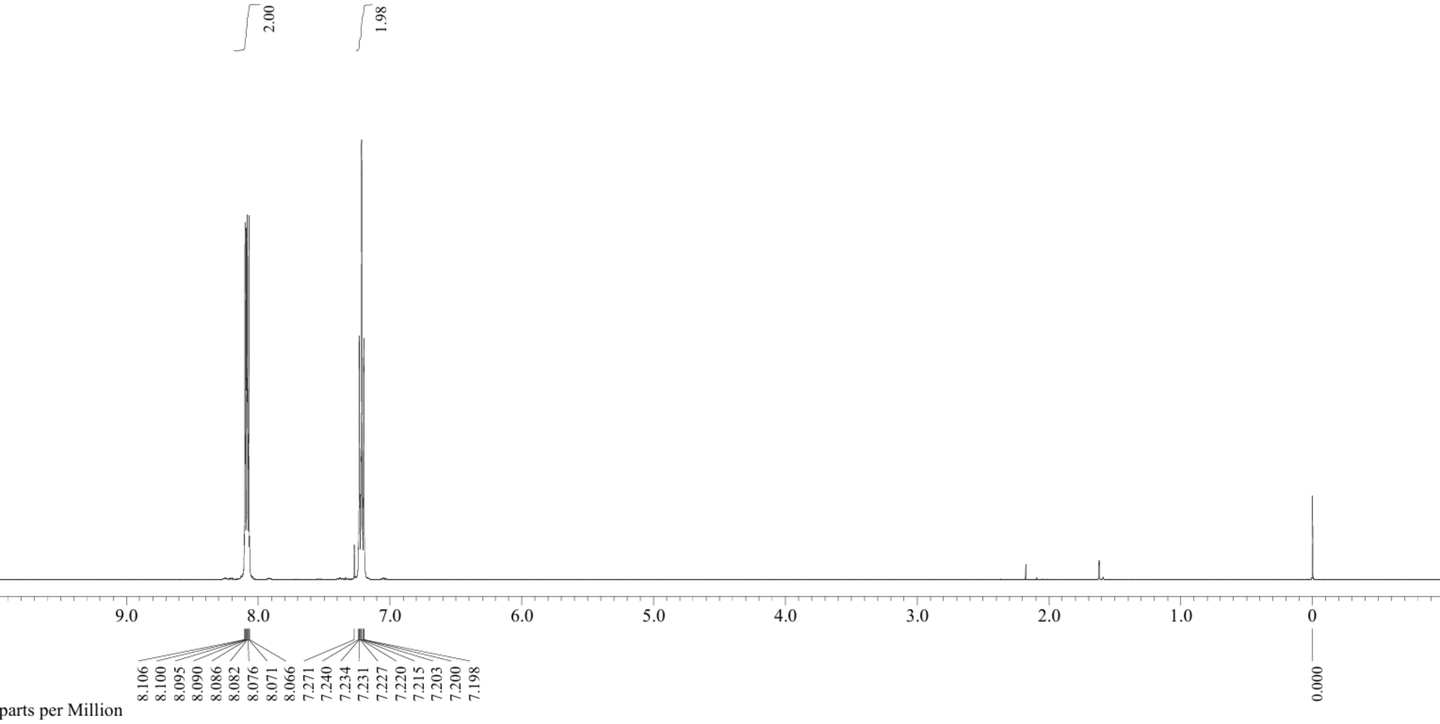

^{1}H NMR (500 MHz, $CDCl_3$)

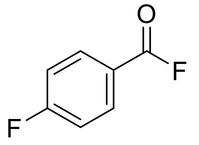

1e

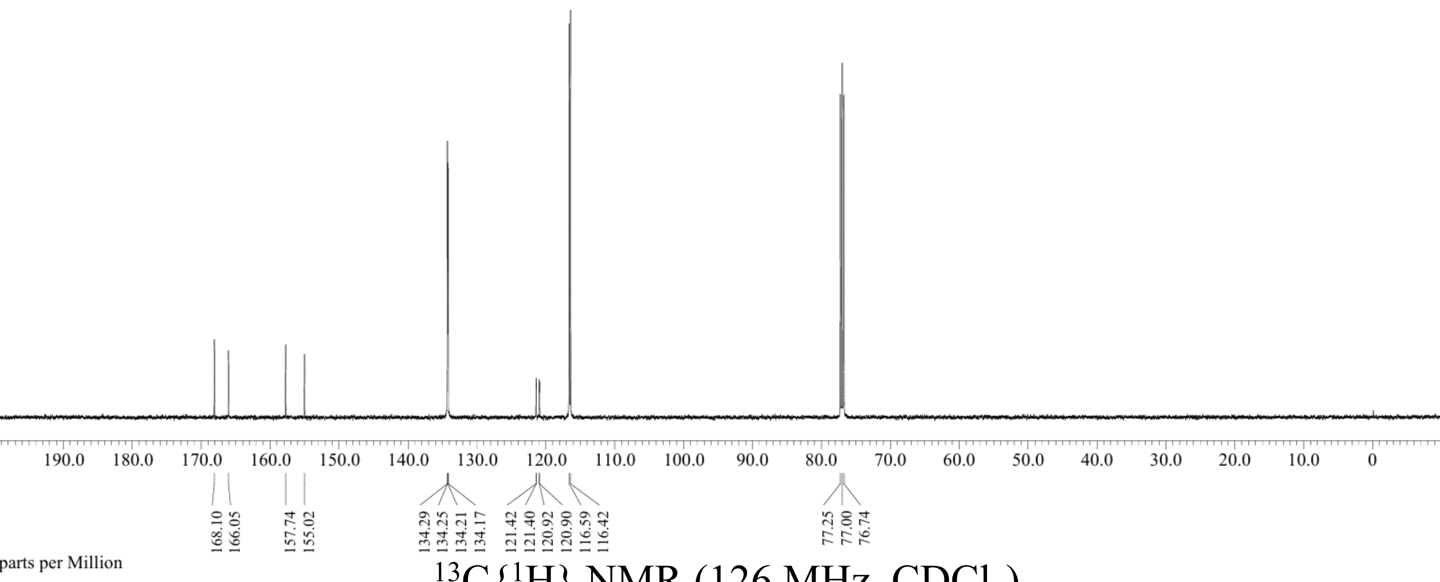

$^{13}C\{^{1}H\}$ NMR (126 MHz, $CDCl_3$)

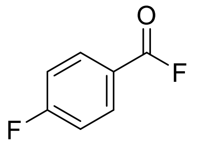

1e

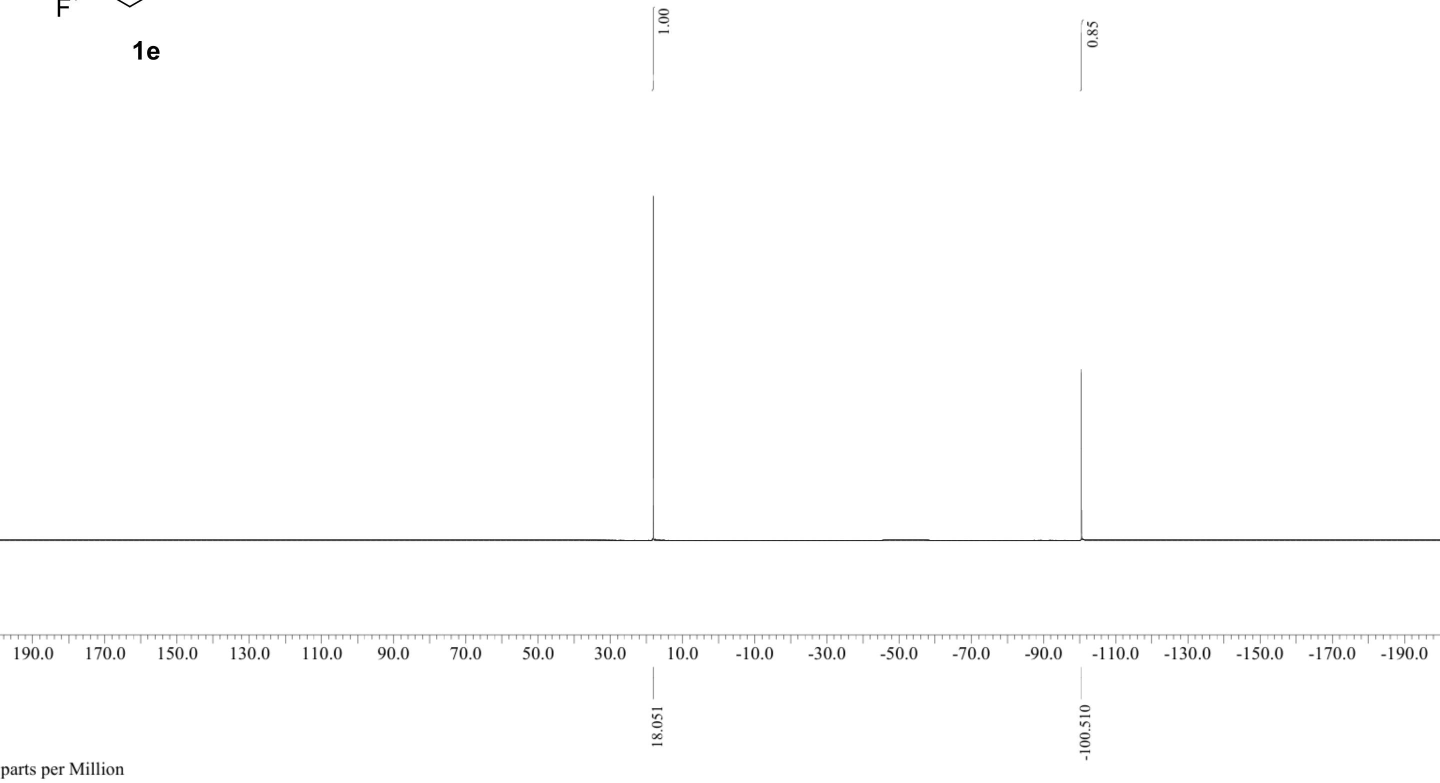

^{19}F NMR (471 MHz, $CDCl_3$)

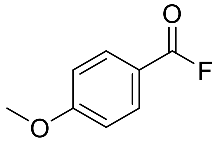

1f

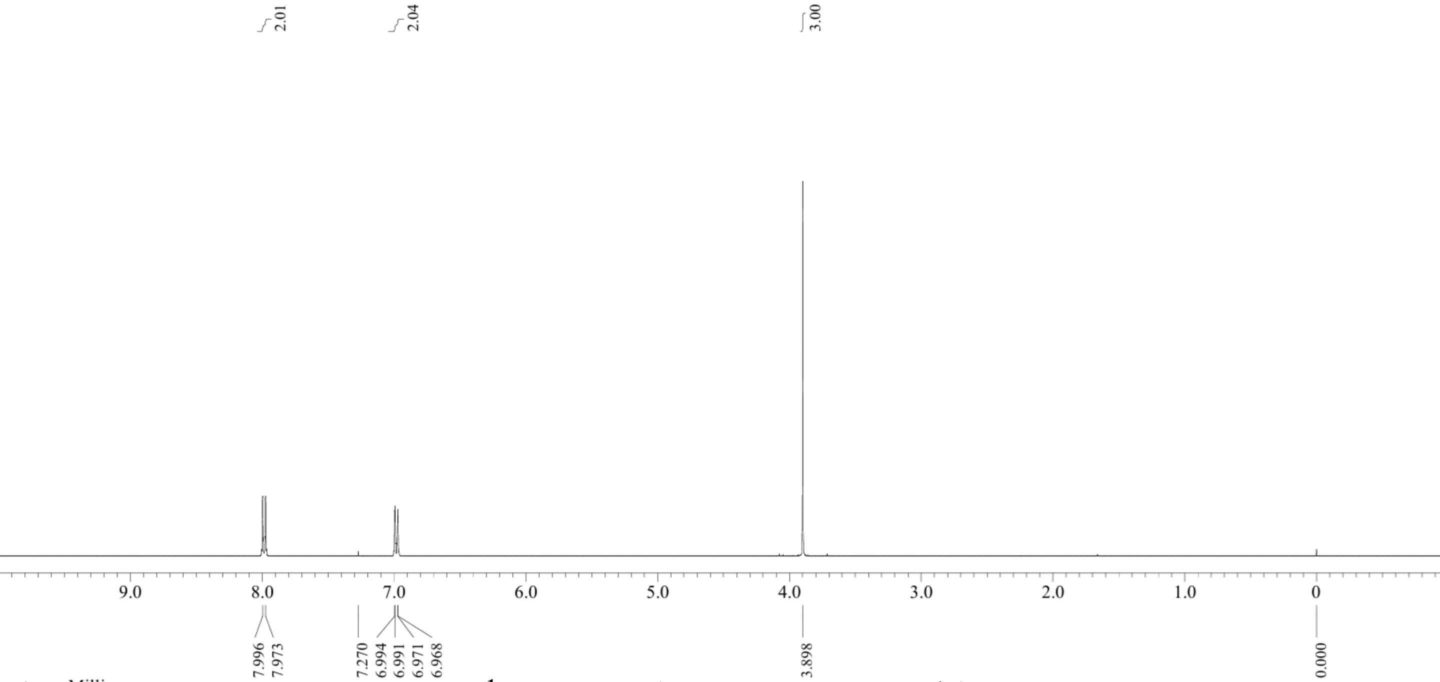

^{1}H NMR (400 MHz, $CDCl_3$)

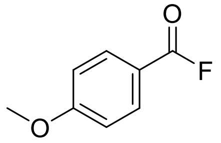

1f

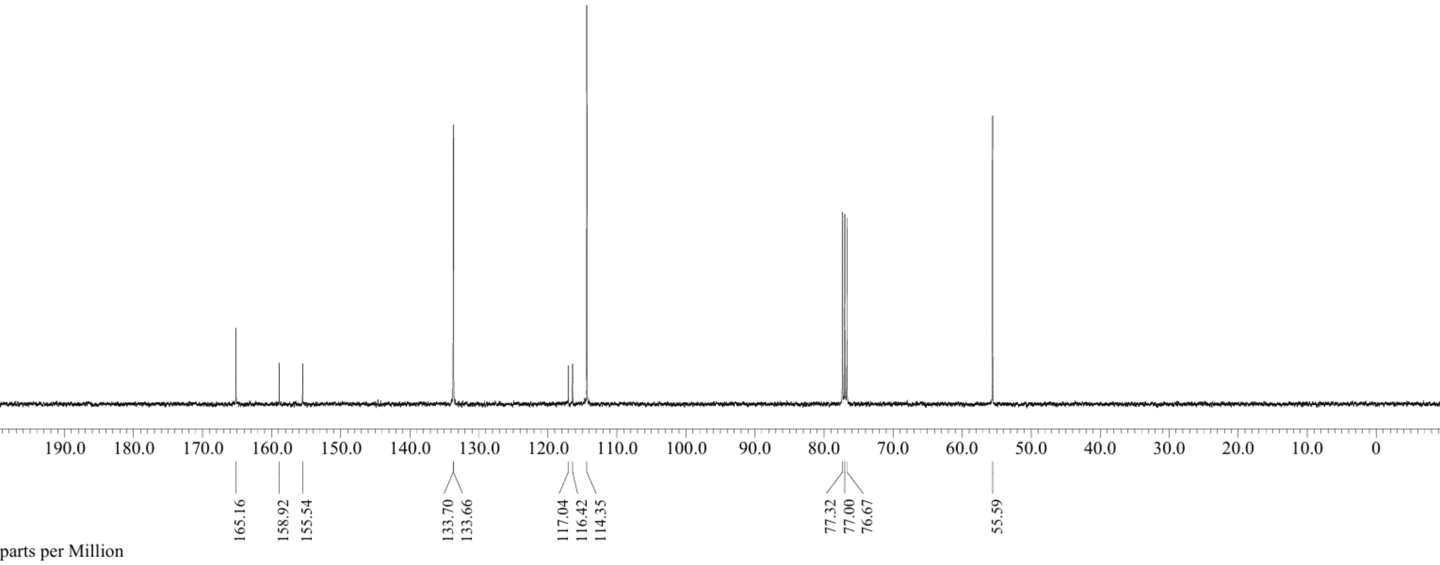

$^{13}C\{^{1}H\}$ NMR (100 MHz, $CDCl_3$)

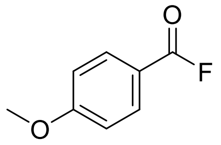

1f

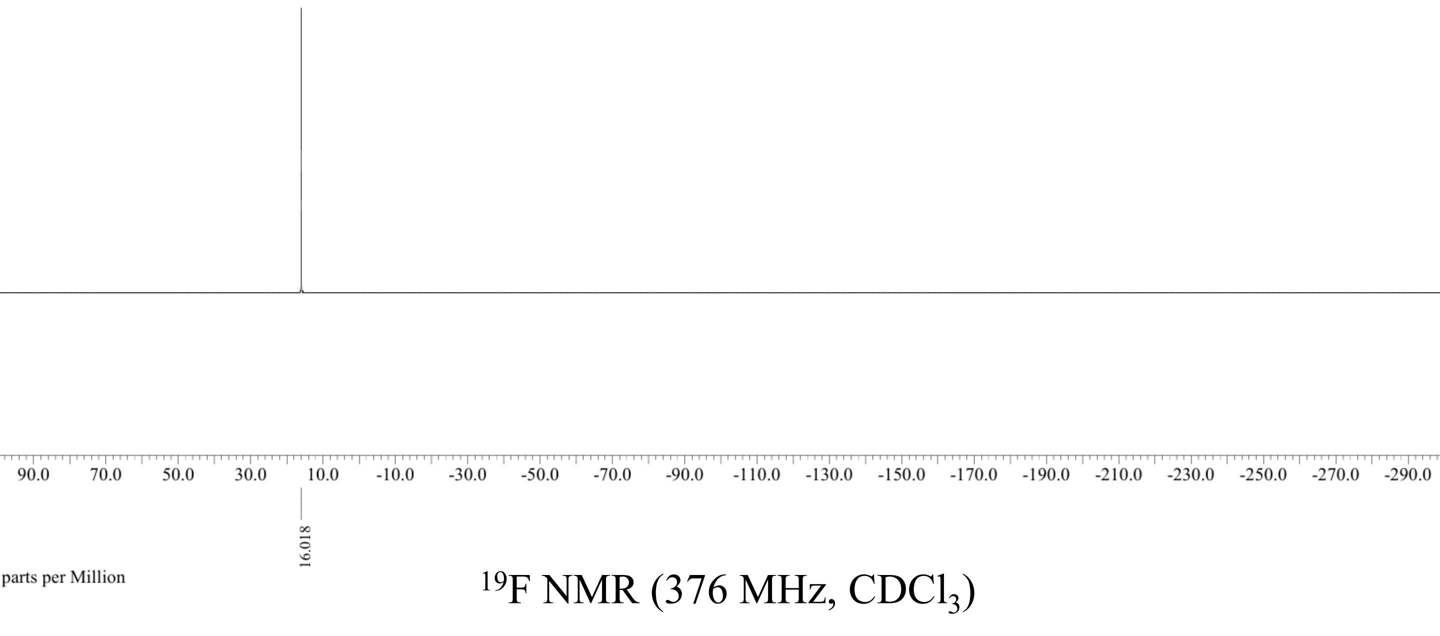

^{19}F NMR (376 MHz, $CDCl_3$)

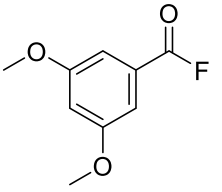

1g

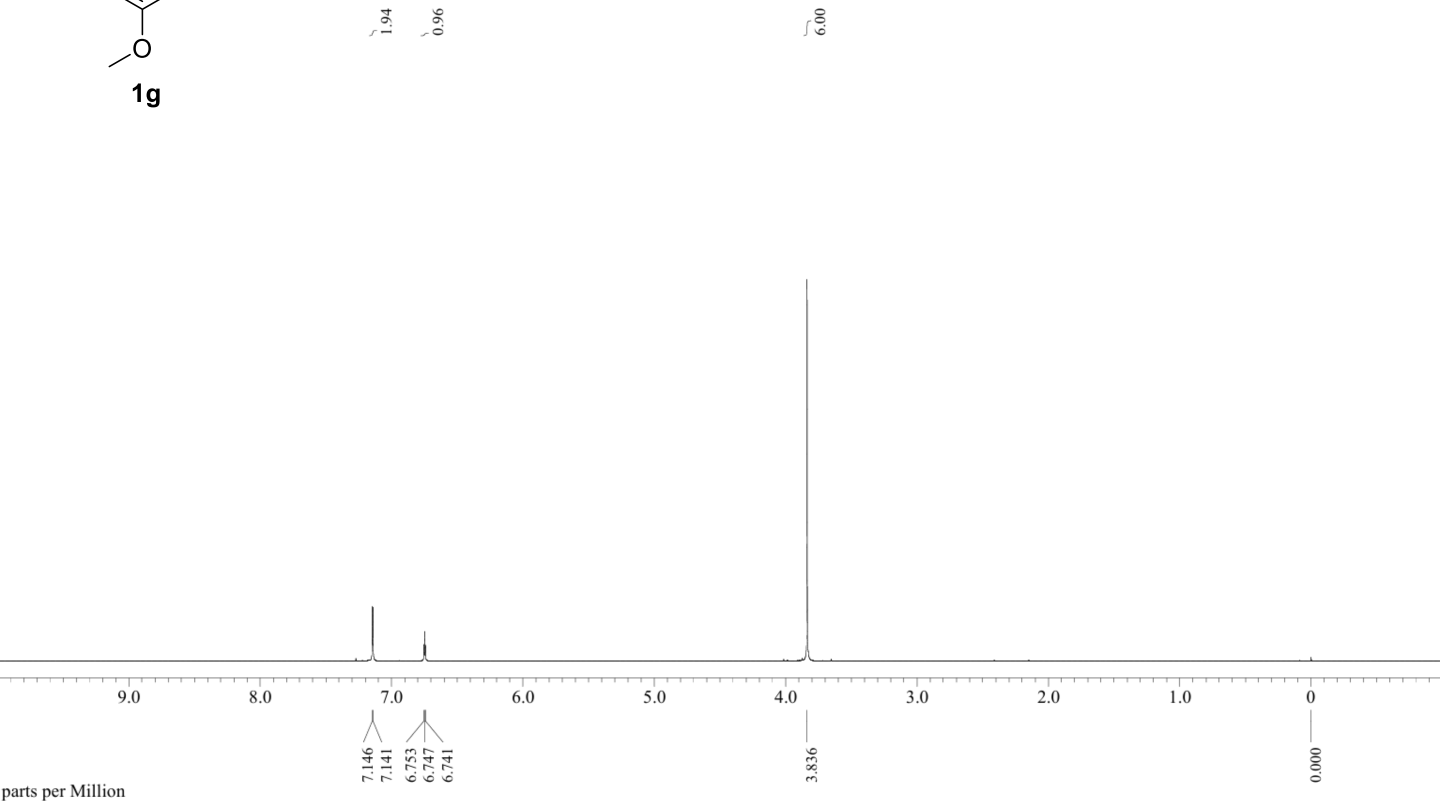

^{1}H NMR (500 MHz, $CDCl_3$)

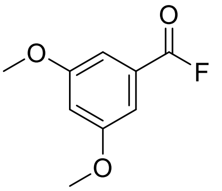

1g

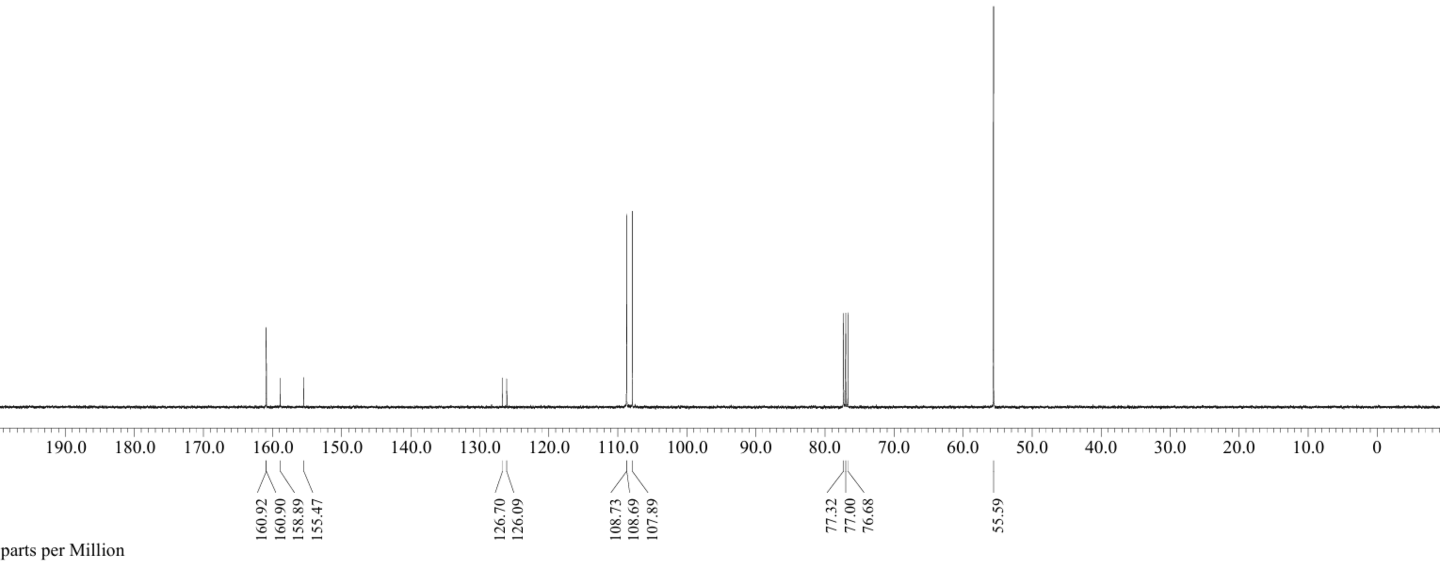

$^{13}C\{^{1}H\}$ NMR (126 MHz, $CDCl_3$)

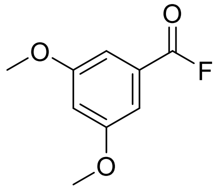

1g

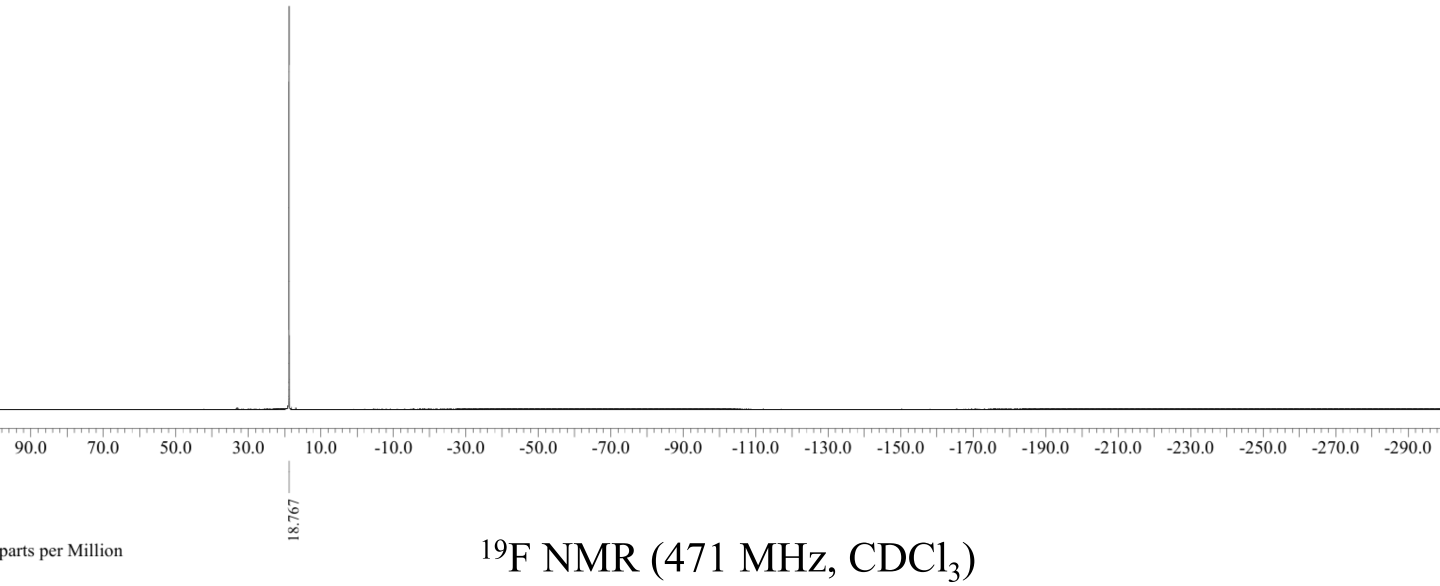

^{19}F NMR (471 MHz, $CDCl_3$)

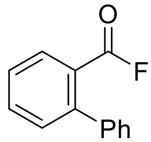

1h

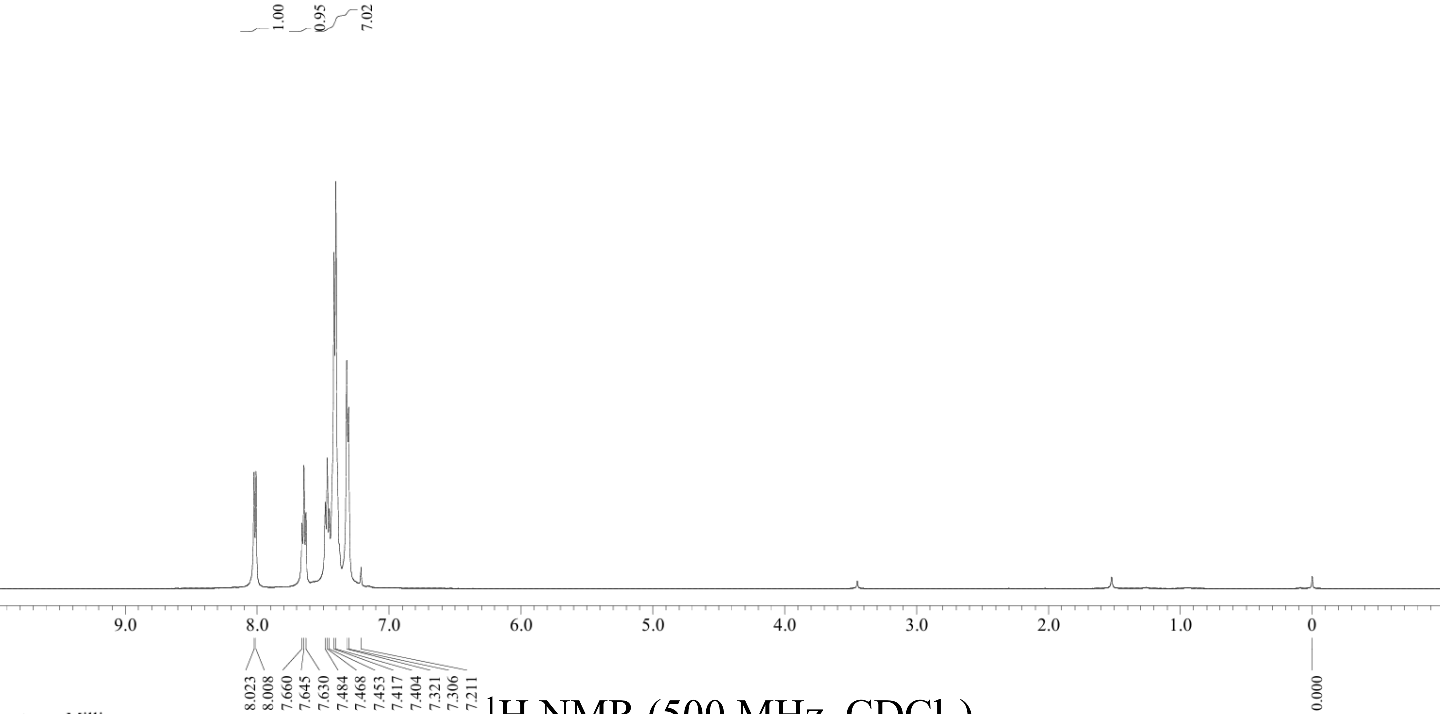

^{1}H NMR (500 MHz, $CDCl_3$)

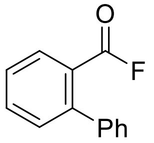

1h

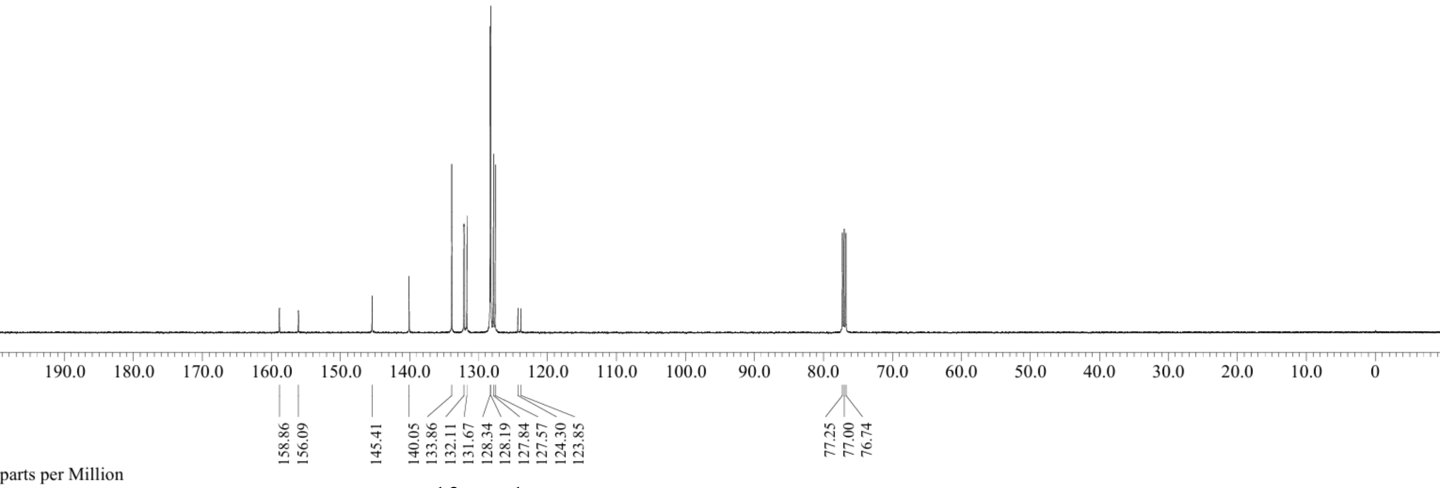

$^{13}C\{^{1}H\}$ NMR (126 MHz, $CDCl_3$)

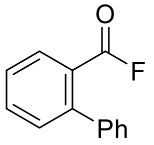

1h

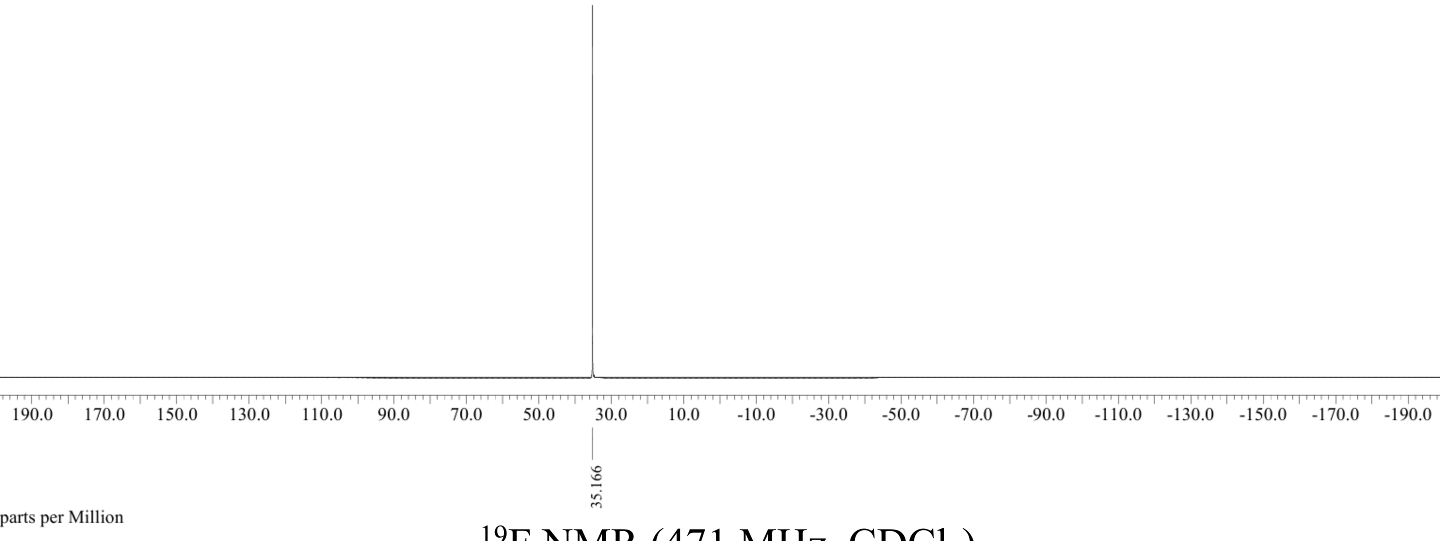

^{19}F NMR (471 MHz, $CDCl_3$)

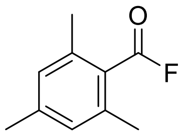

1j

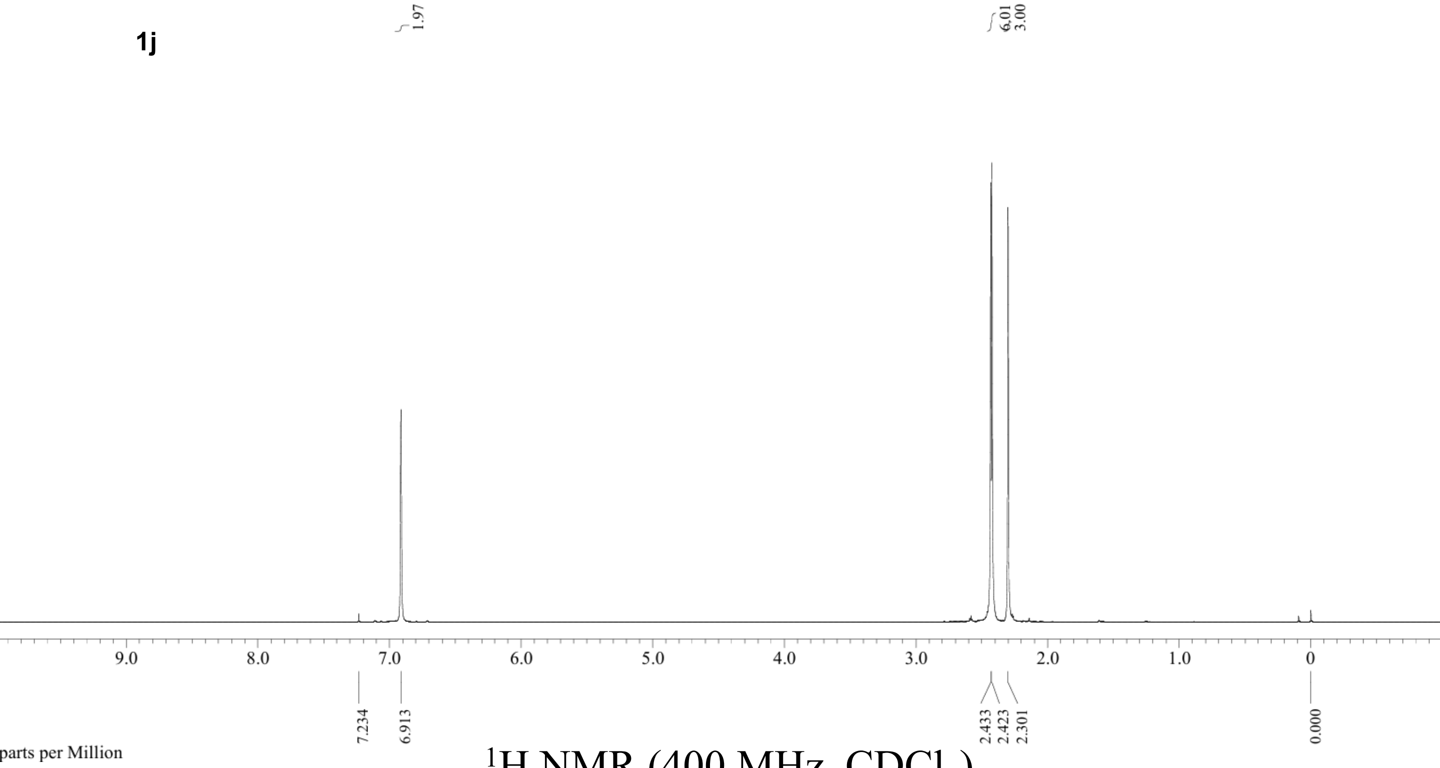

^{1}H NMR (400 MHz, $CDCl_3$)

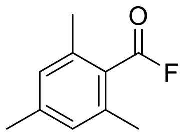

1i

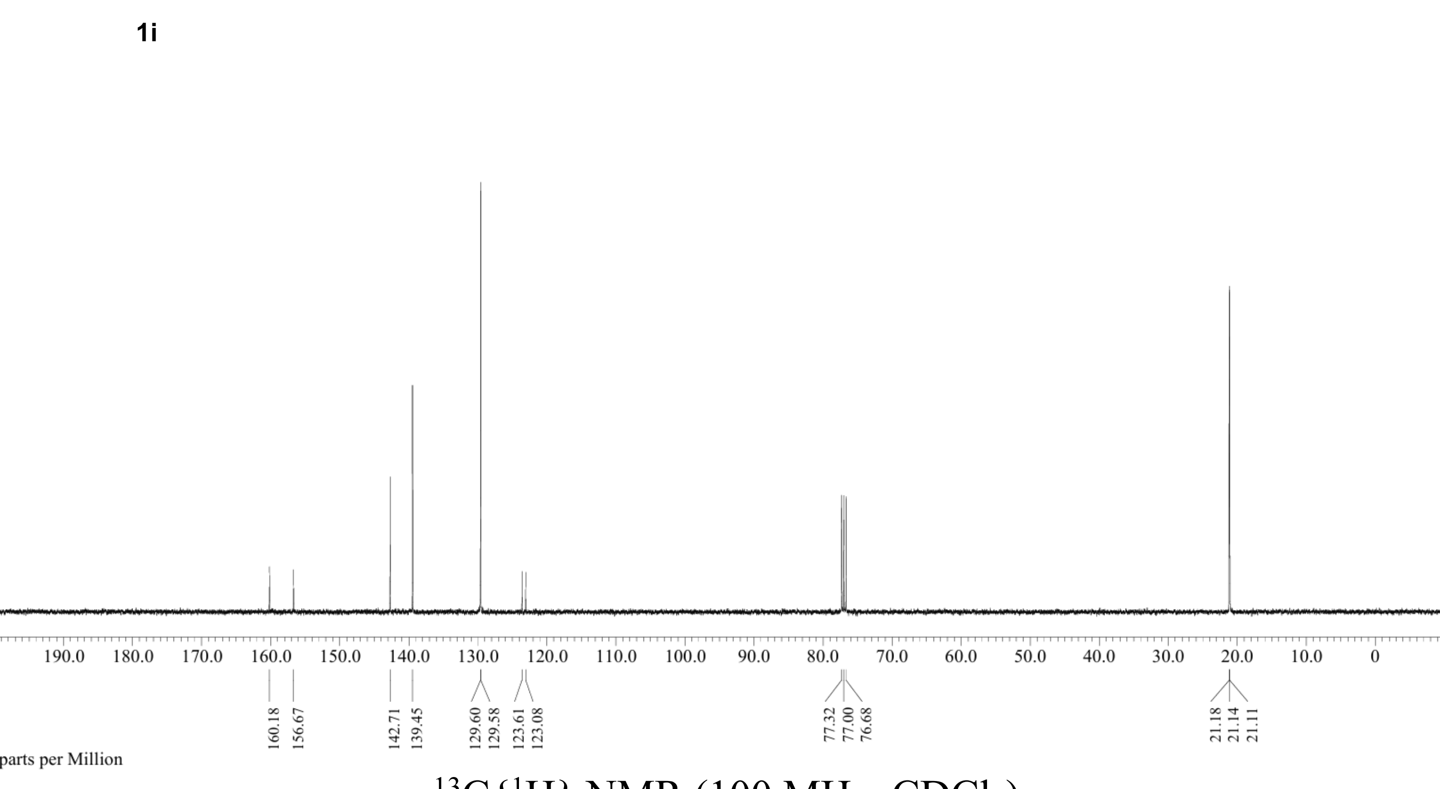

$^{13}C\{^{1}H\}$ NMR (100 MHz, $CDCl_3$)

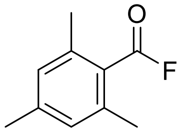

1i

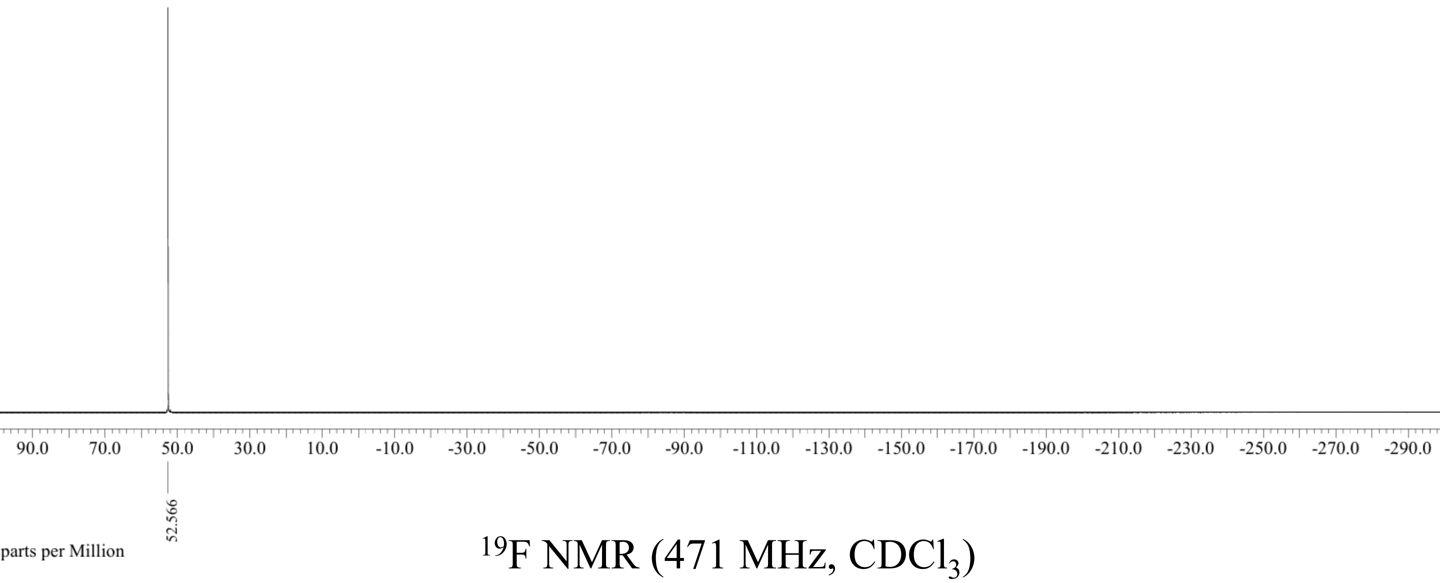

^{19}F NMR (471 MHz, $CDCl_3$)

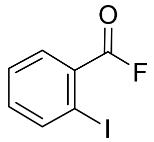

1j

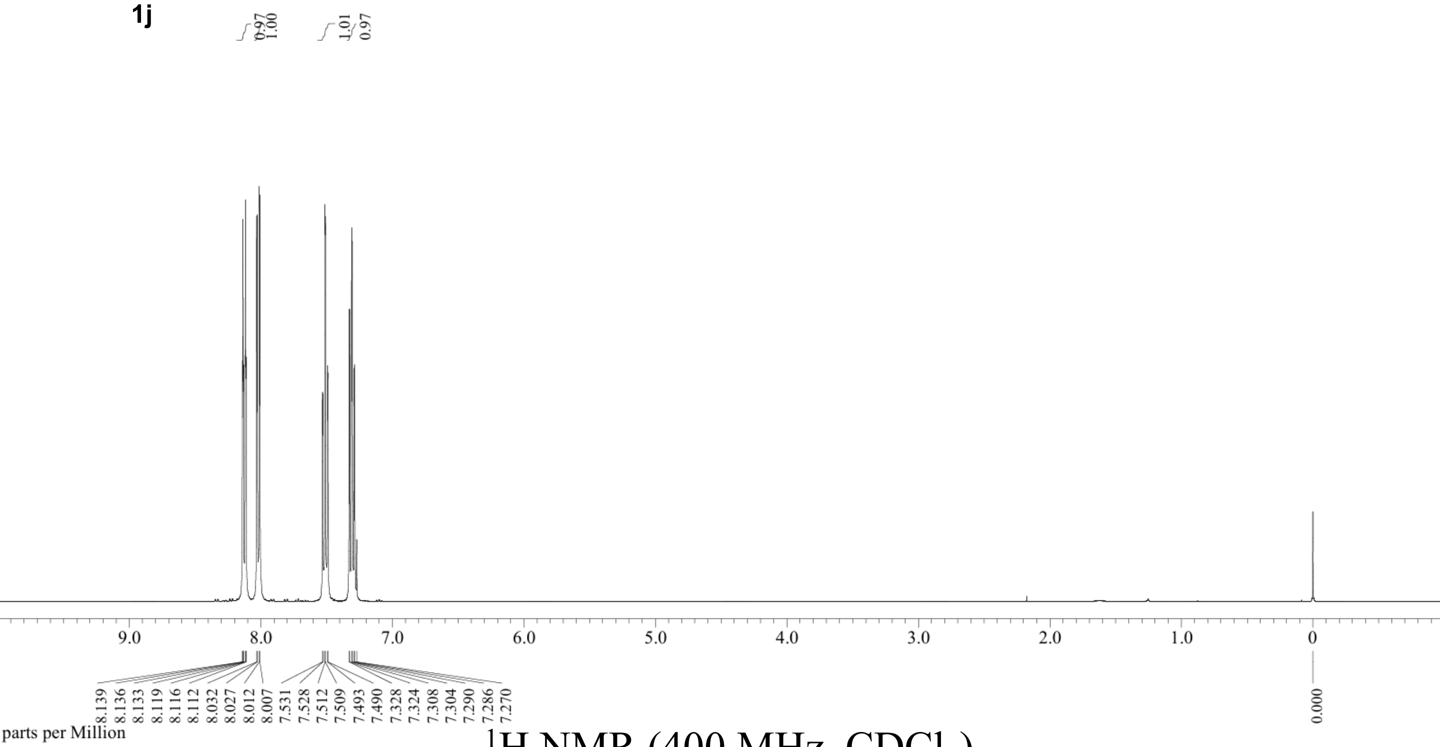

^{1}H NMR (400 MHz, $CDCl_3$)

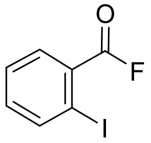

1j

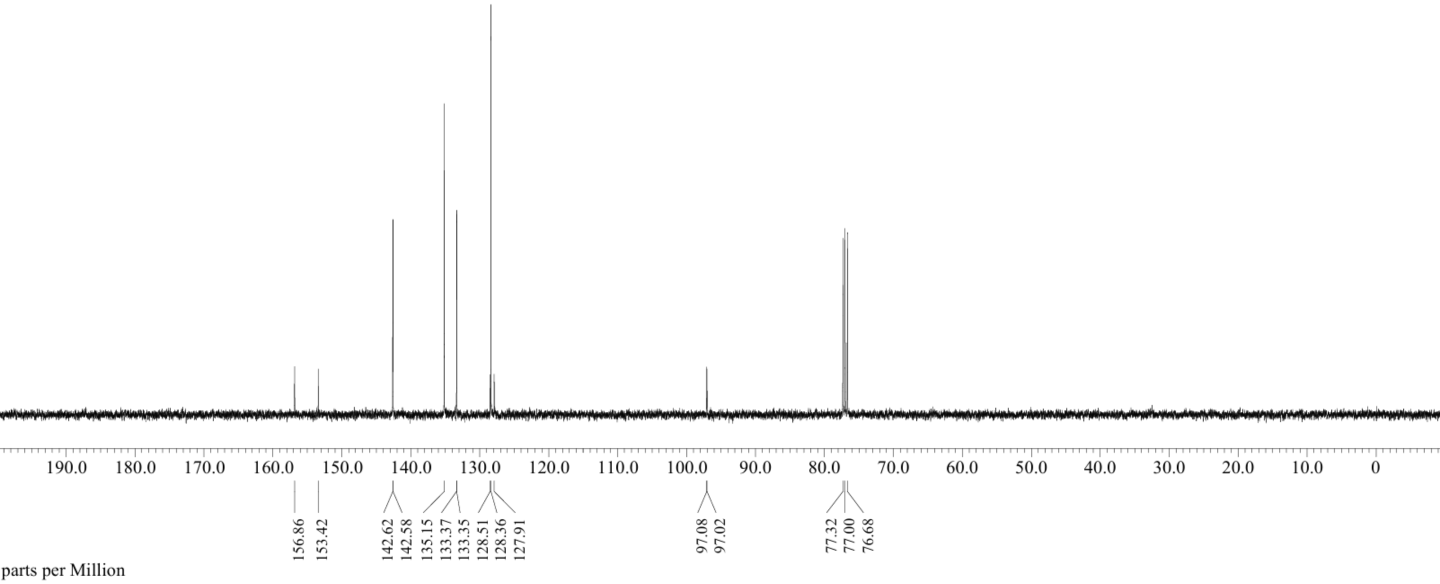

$^{13}C\{^{1}H\}$ NMR (126 MHz, $CDCl_3$)

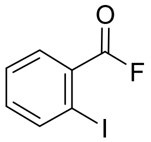

1j

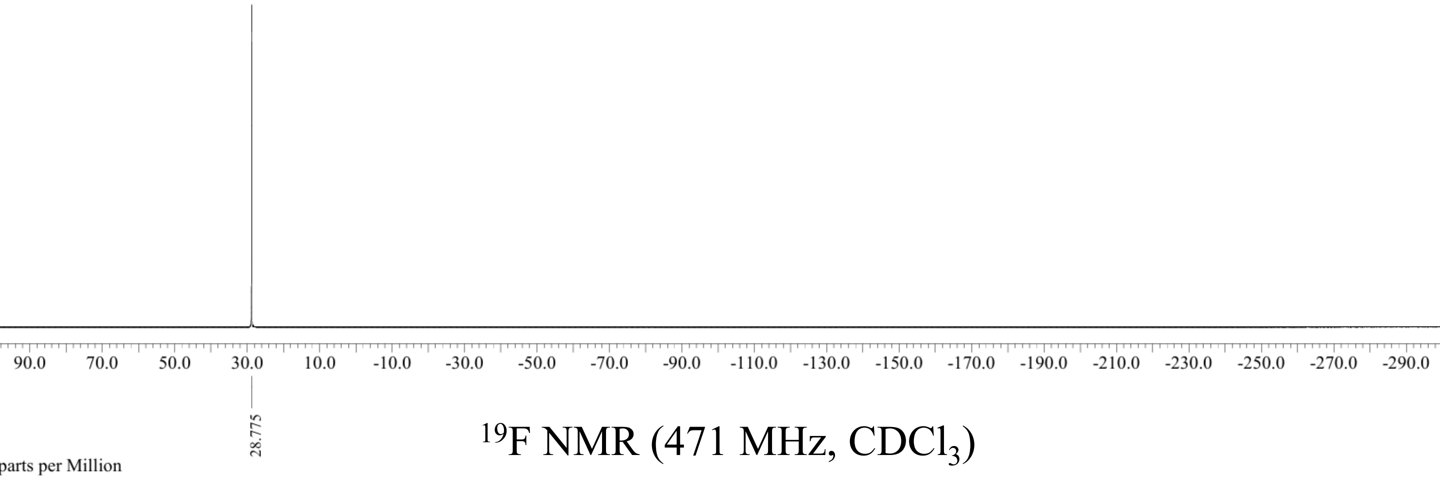

^{19}F NMR (471 MHz, $CDCl_3$)

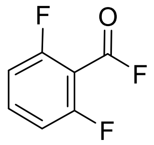

1k

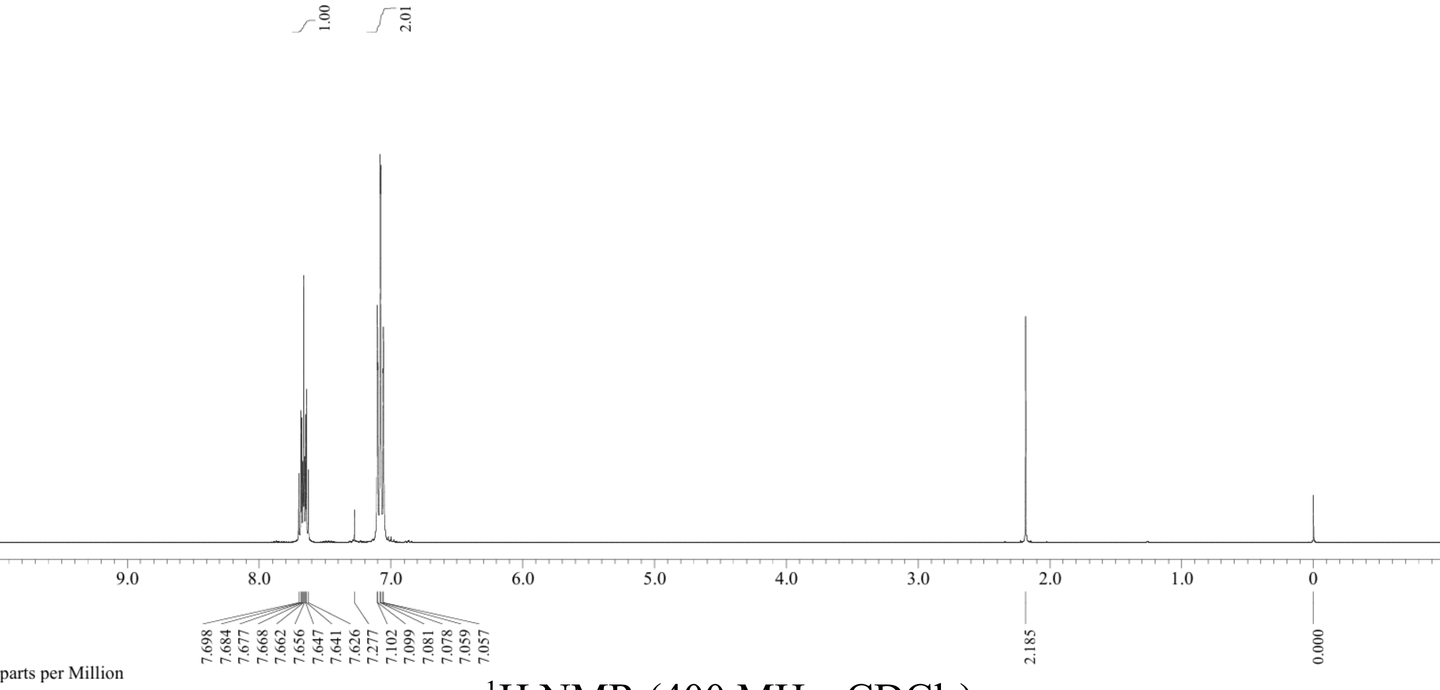

^{1}H NMR (400 MHz, $CDCl_3$)

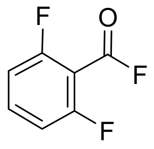

1k

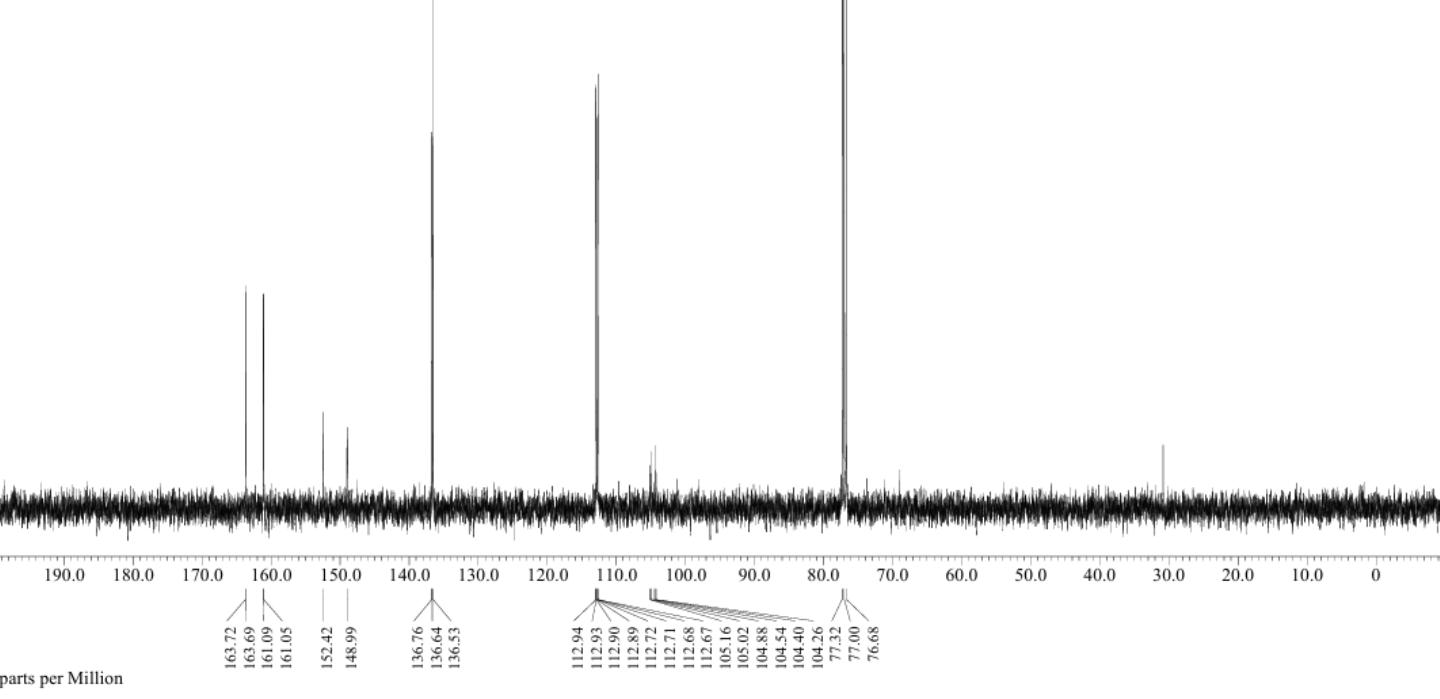

$^{13}C\{^{1}H\}$ NMR (100 MHz, $CDCl_3$)

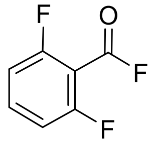

1k

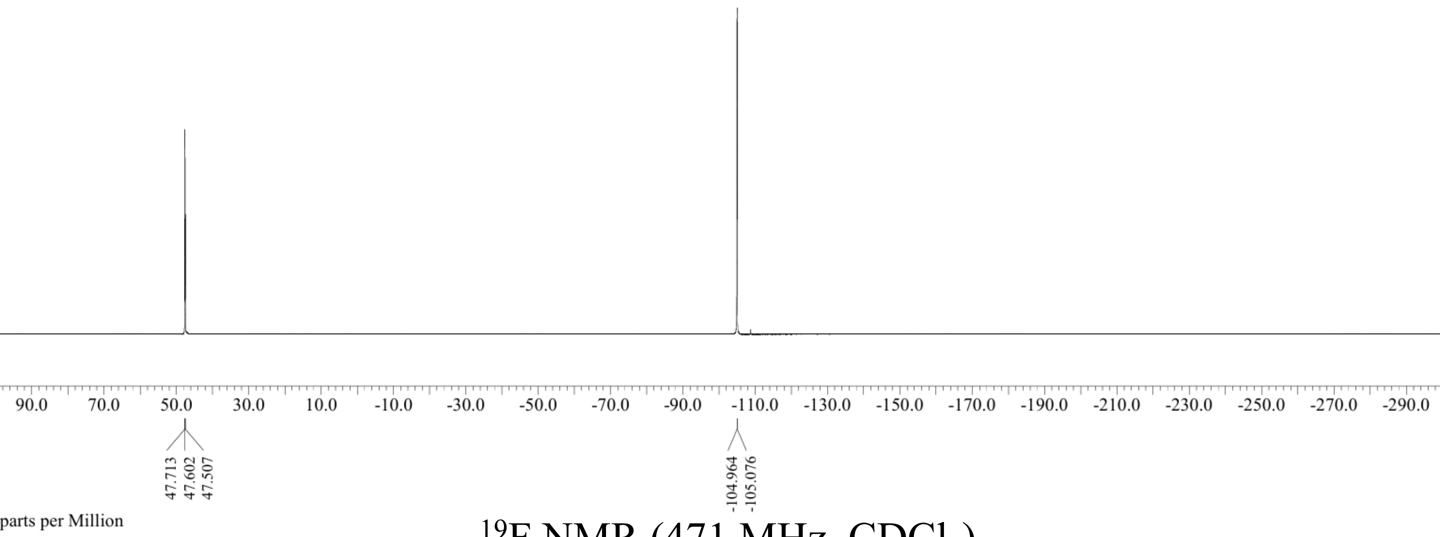

^{19}F NMR (471 MHz, $CDCl_3$)

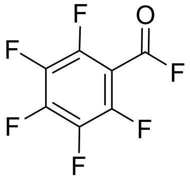

1l

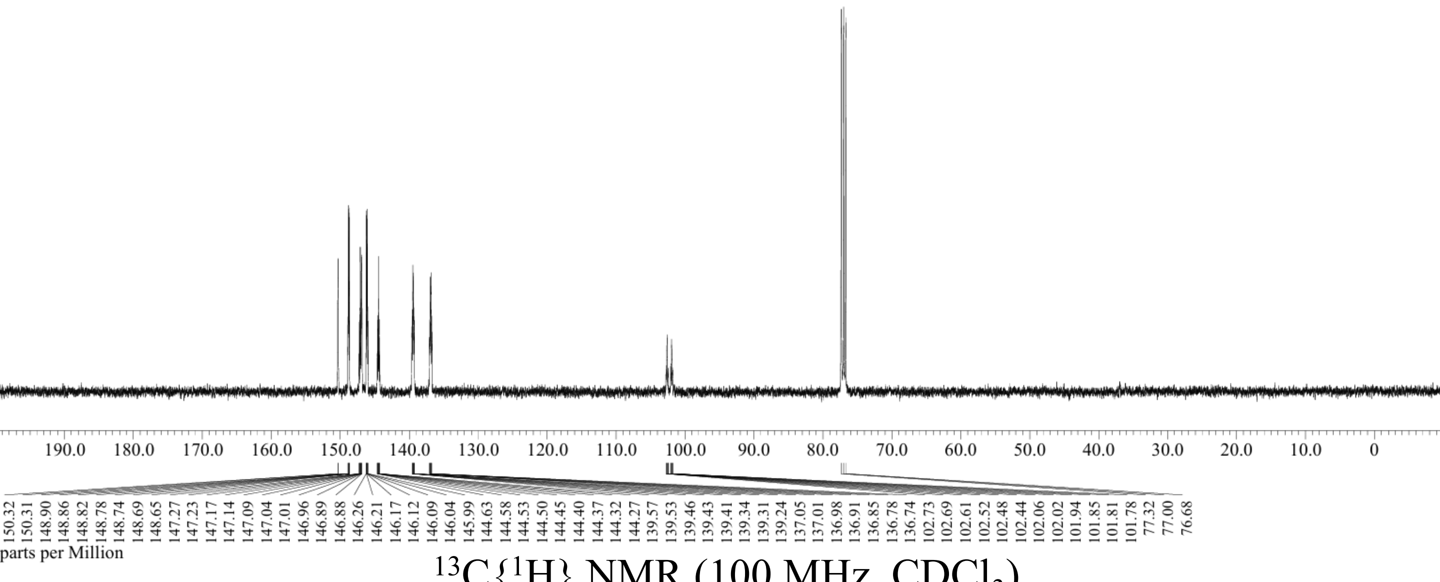

$^{13}C\{^1H\}$ NMR (100 MHz, $CDCl_3$)

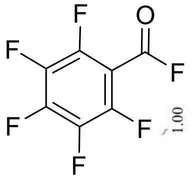

1l

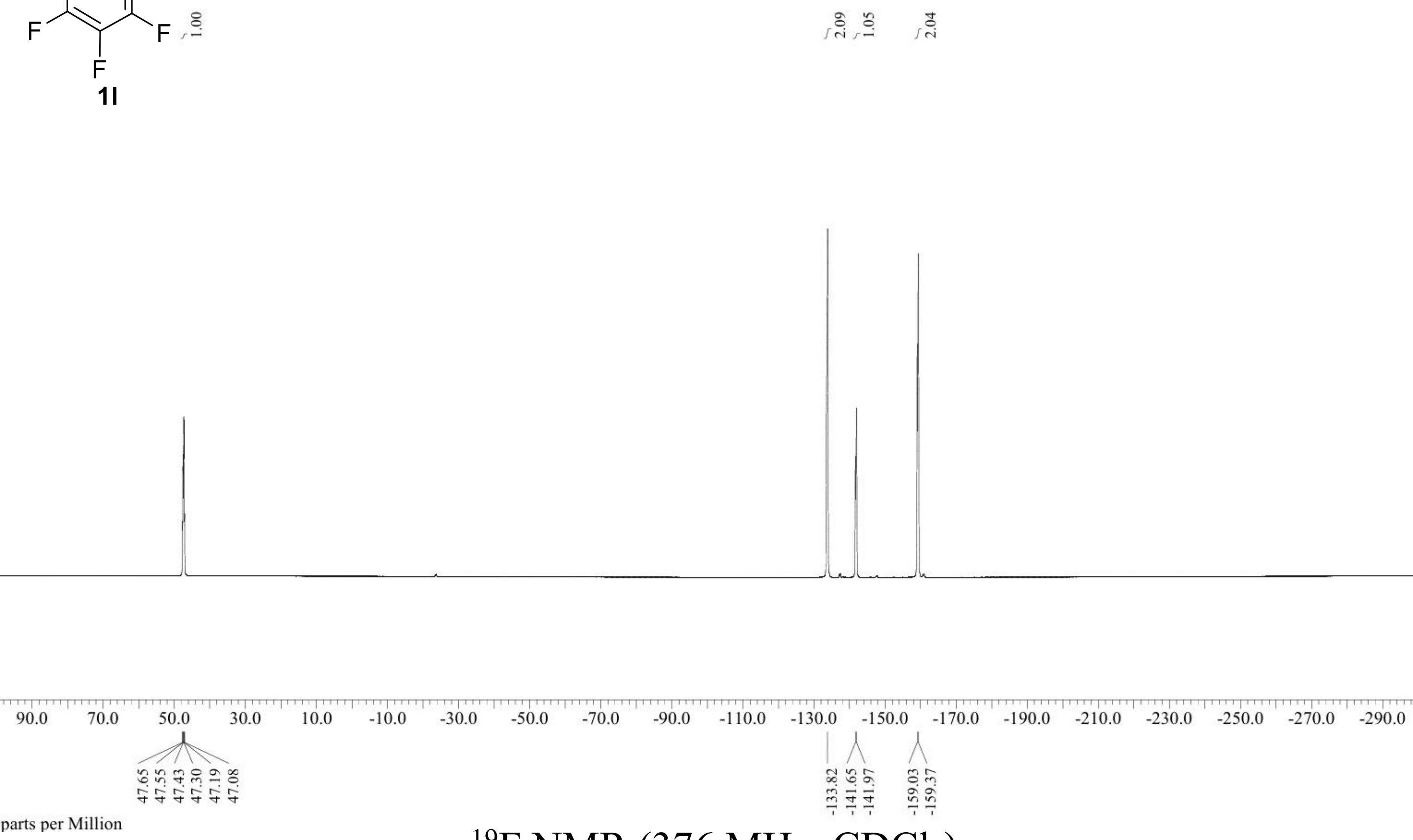

^{19}F NMR (376 MHz, $CDCl_3$)

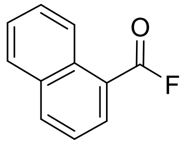

1m

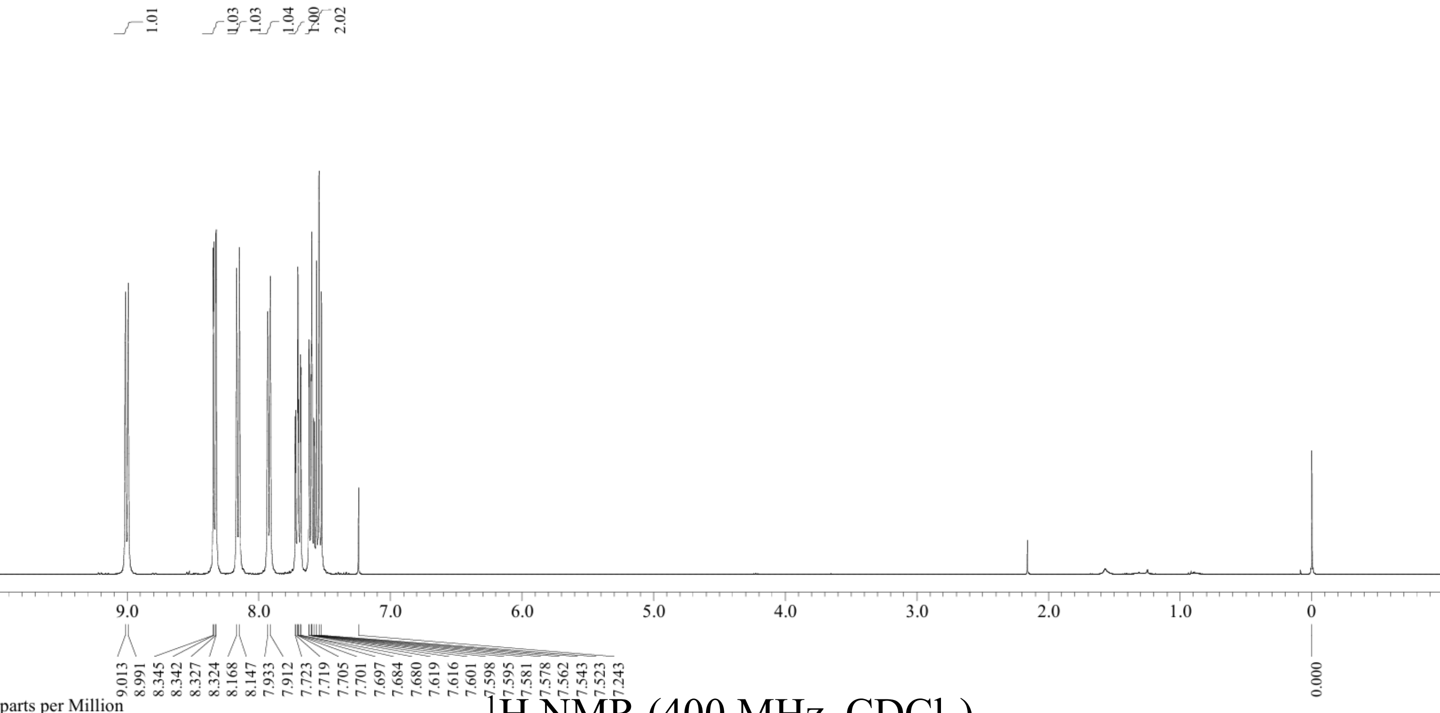

^{1}H NMR (400 MHz, $CDCl_3$)

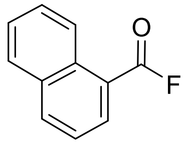

1m

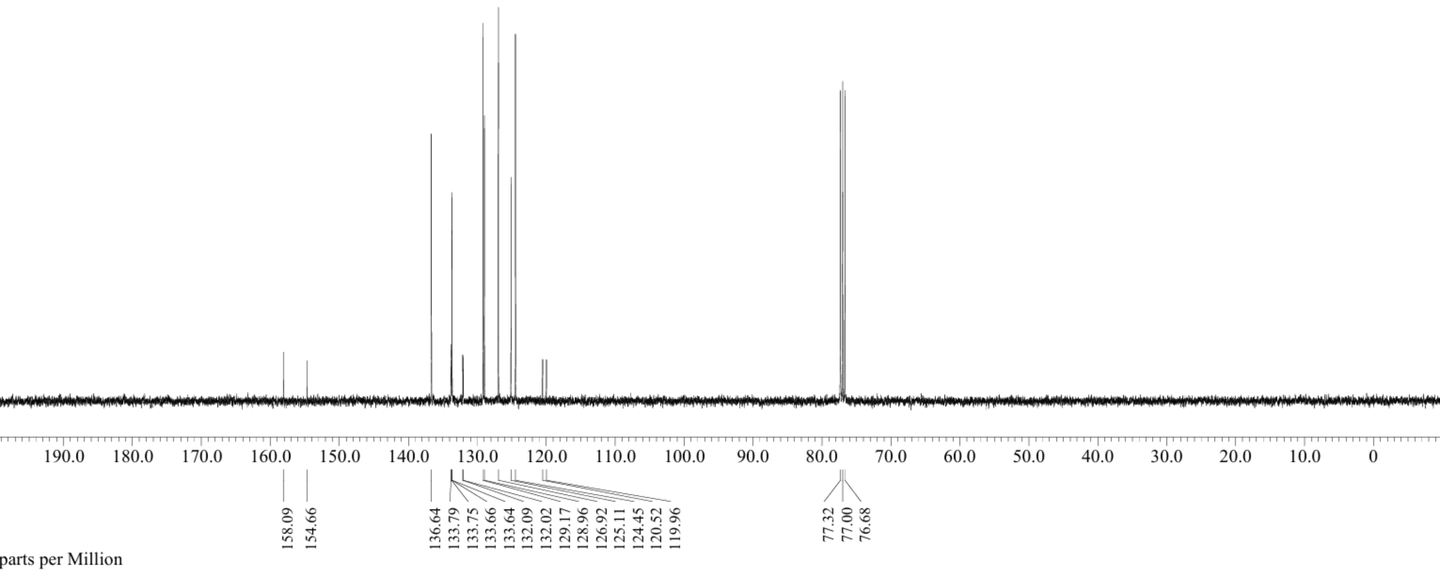

$^{13}C\{^{1}H\}$ NMR (100 MHz, $CDCl_3$)

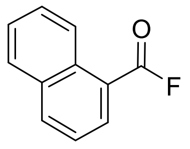

1m

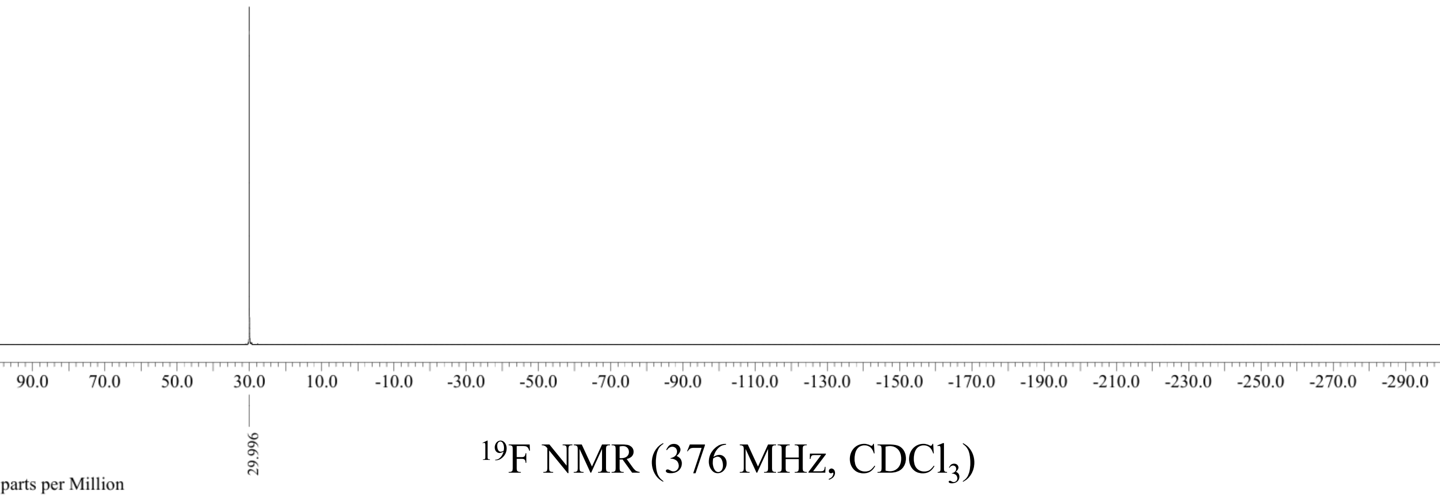

^{19}F NMR (376 MHz, $CDCl_3$)

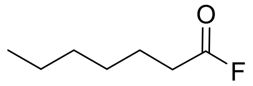

1n

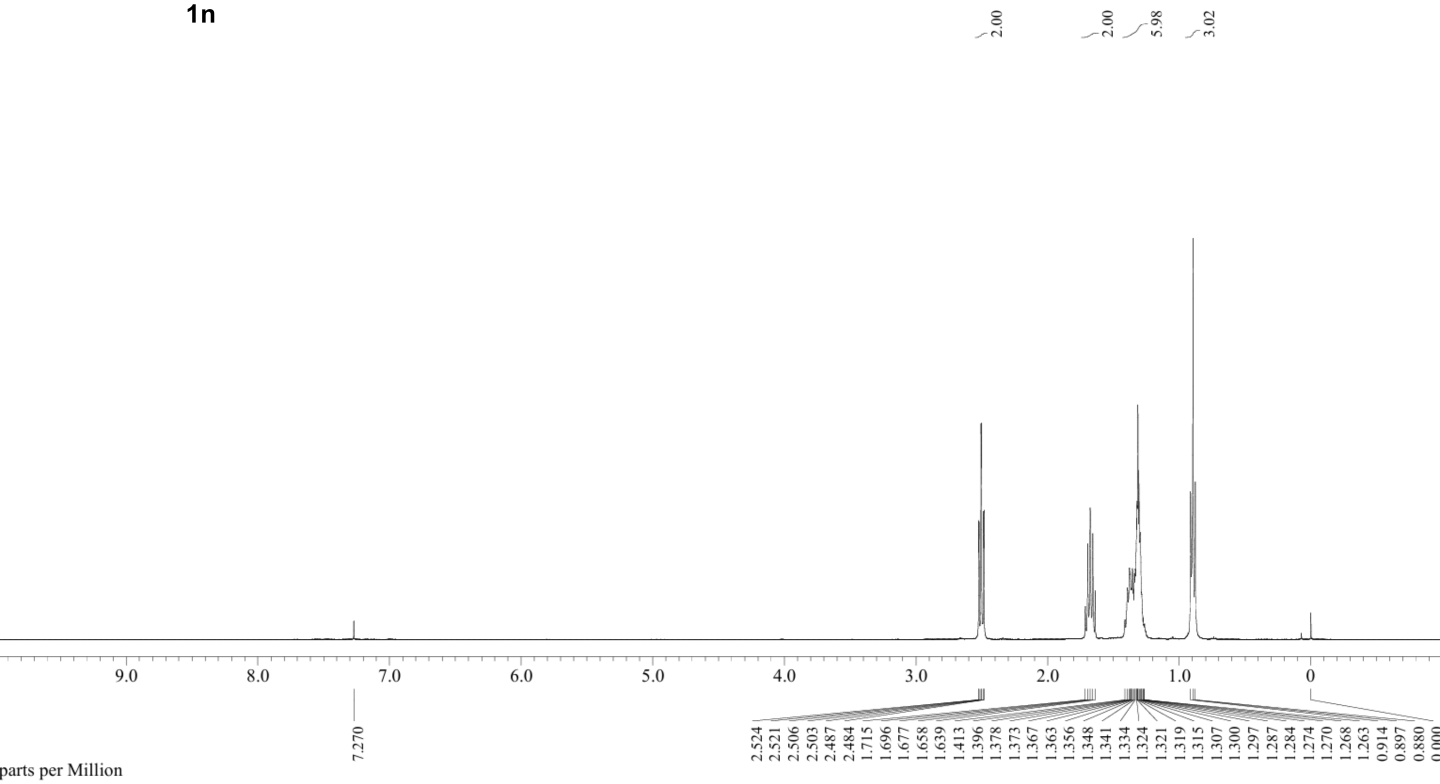

^{1}H NMR (400 MHz, $CDCl_3$)

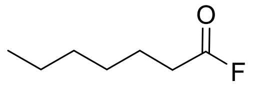

1n

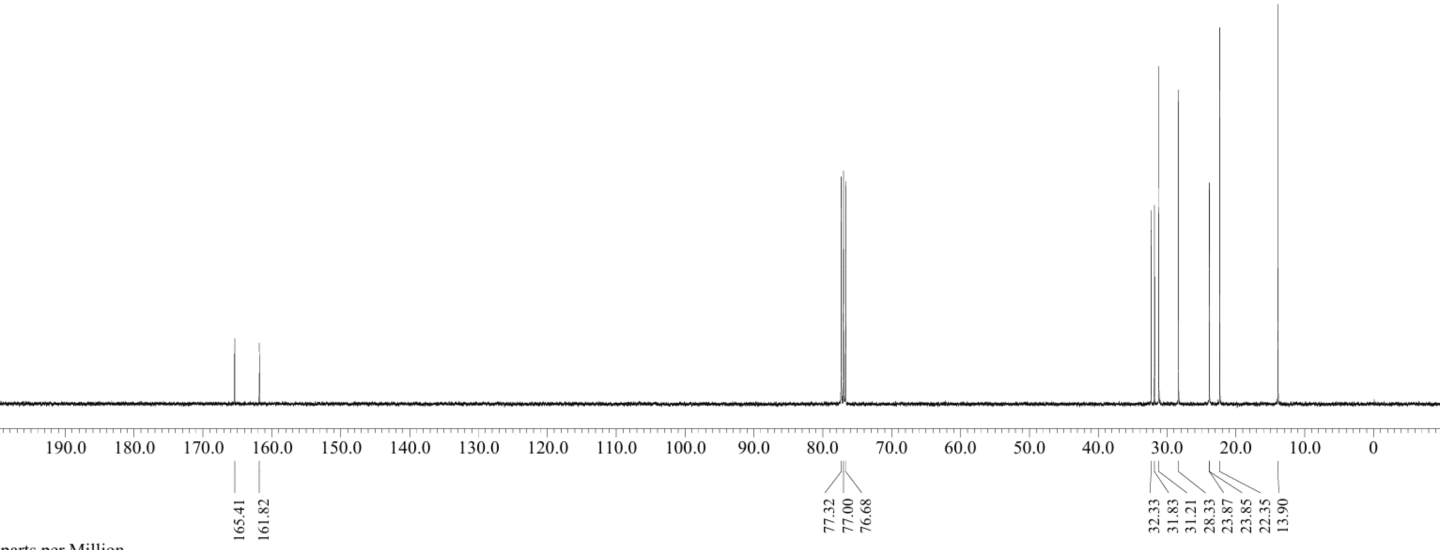

$^{13}C\{^{1}H\}$ NMR (100 MHz, $CDCl_3$)

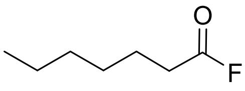

1n

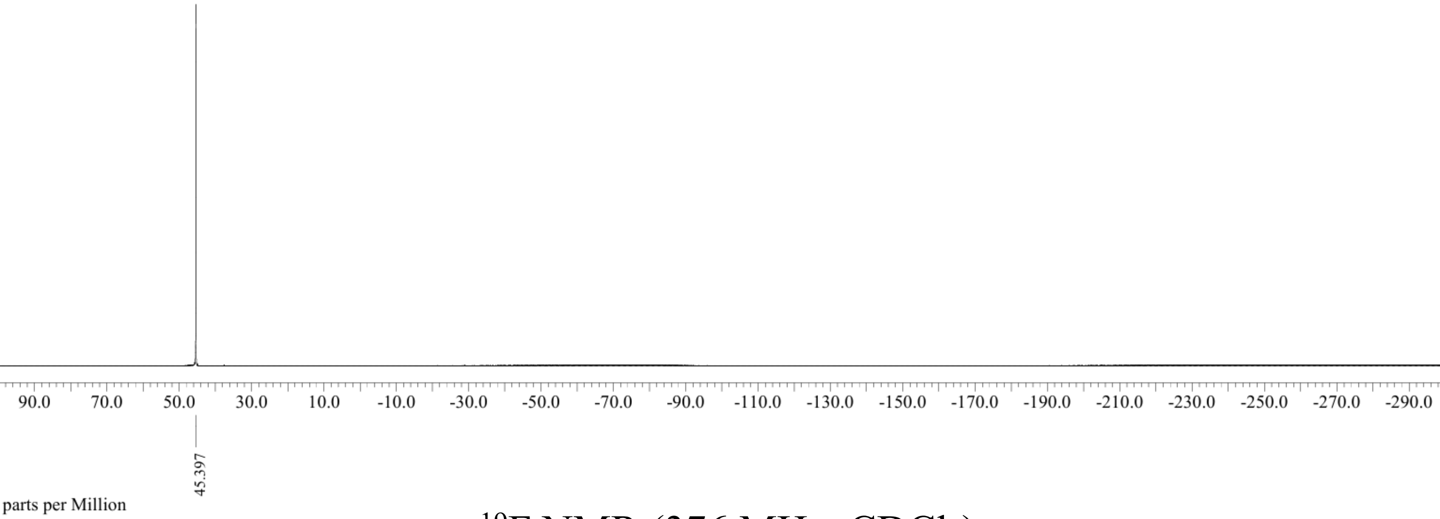

^{19}F NMR (376 MHz, $CDCl_3$)

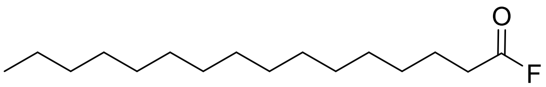

1o

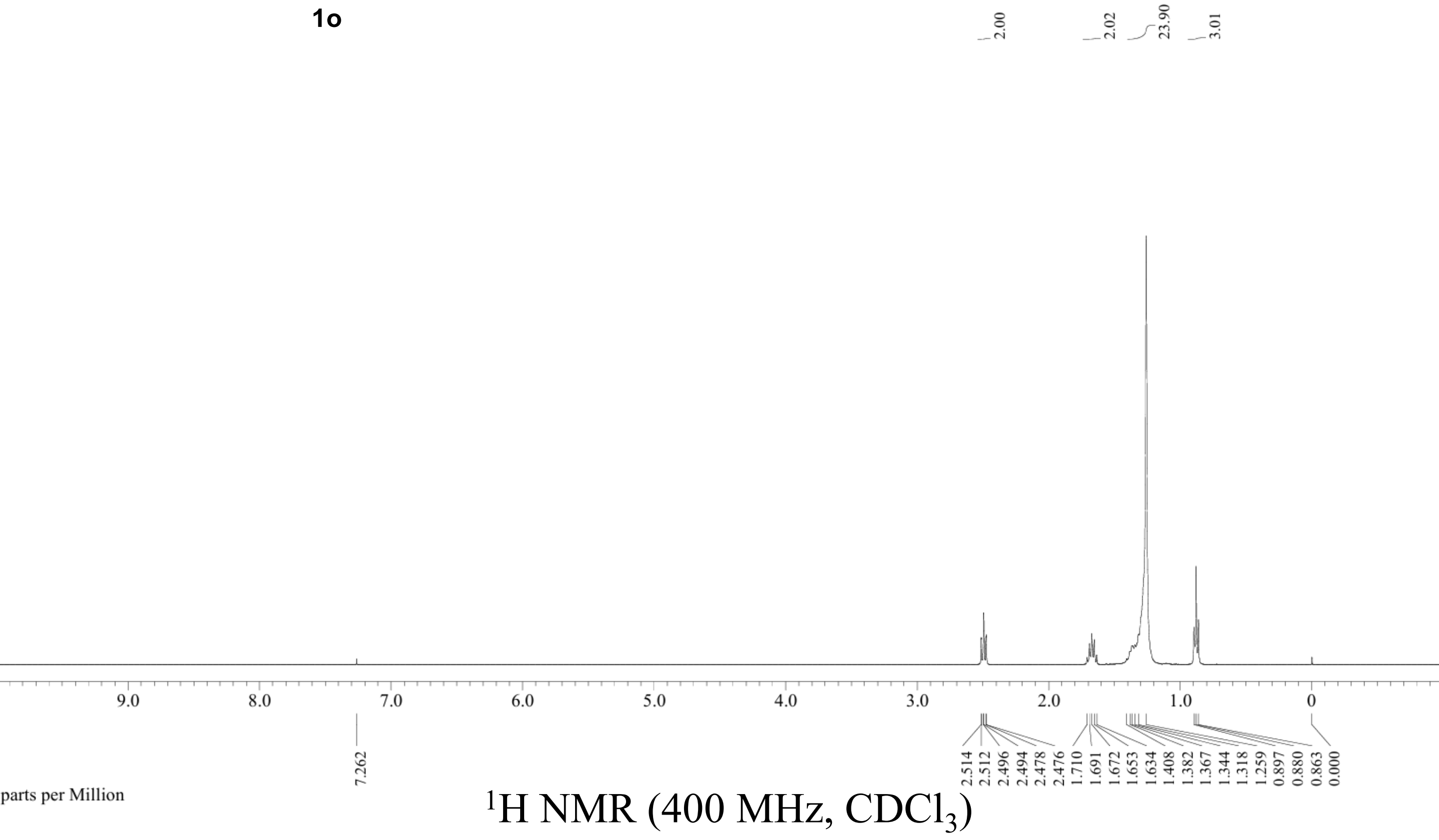

^{1}H NMR (400 MHz, $CDCl_3$)

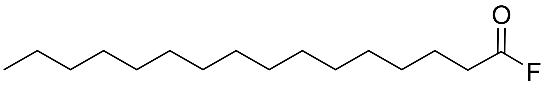

1o

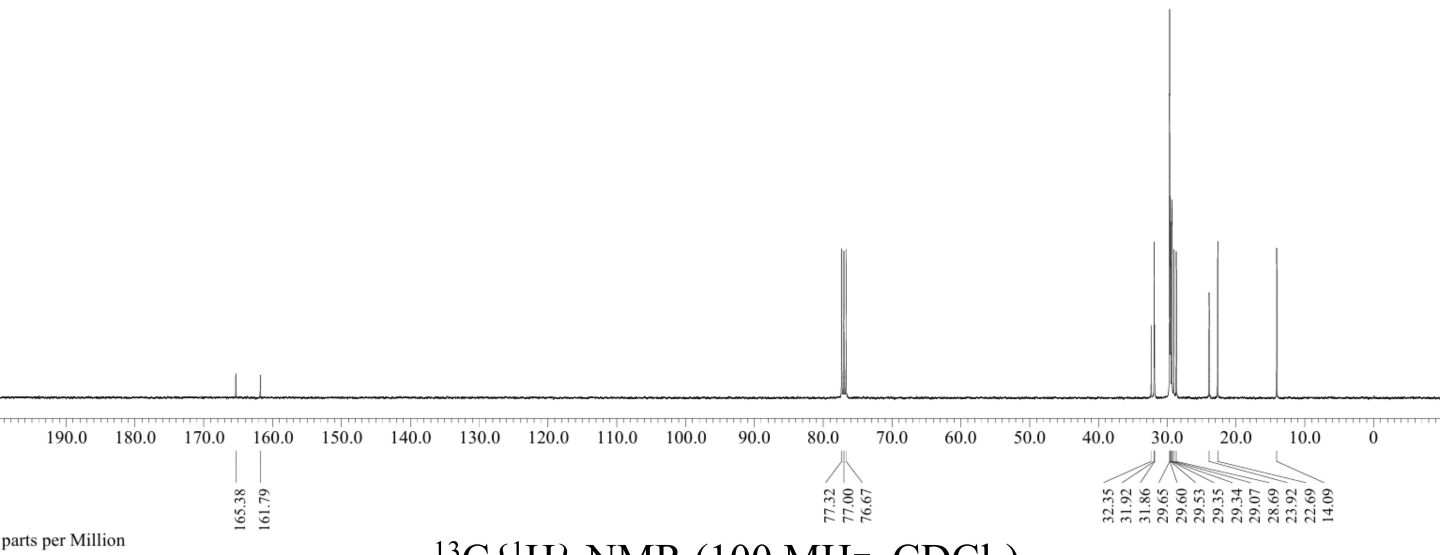

$^{13}C\{^{1}H\}$ NMR (100 MHz, $CDCl_3$)

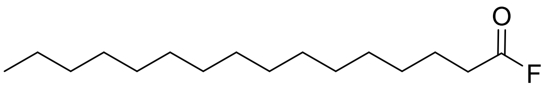

1o

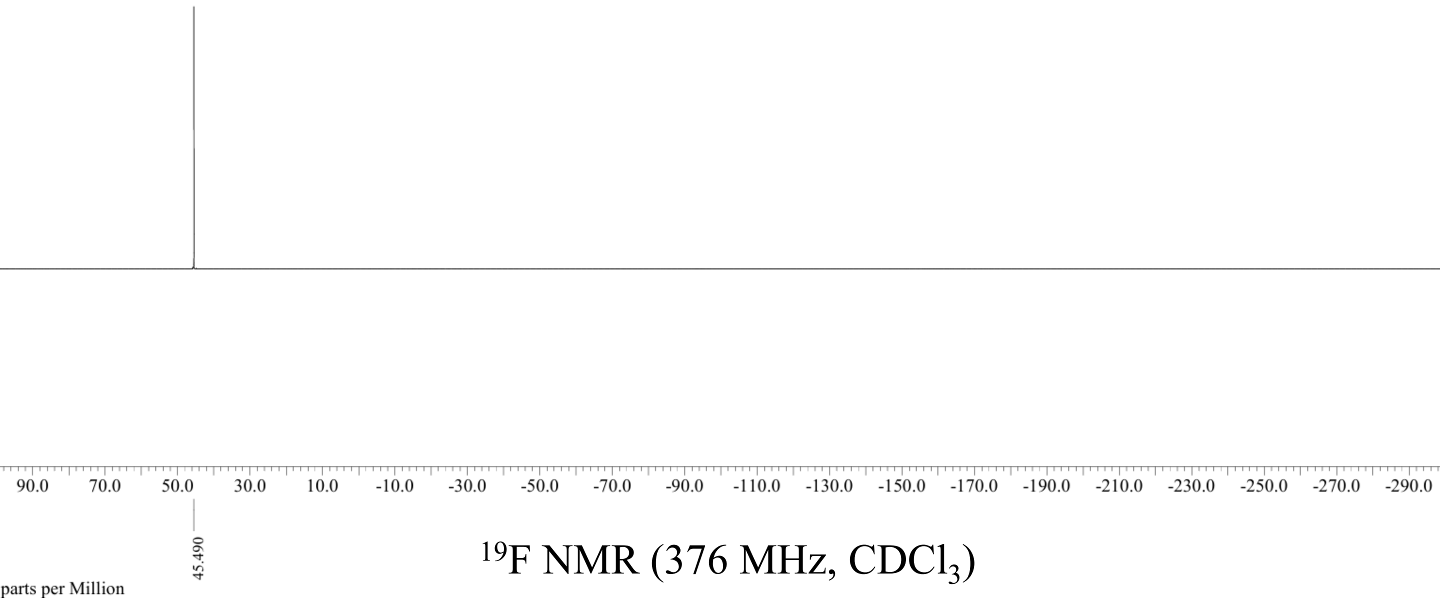

^{19}F NMR (376 MHz, $CDCl_3$)

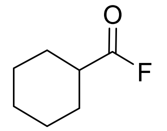

1p

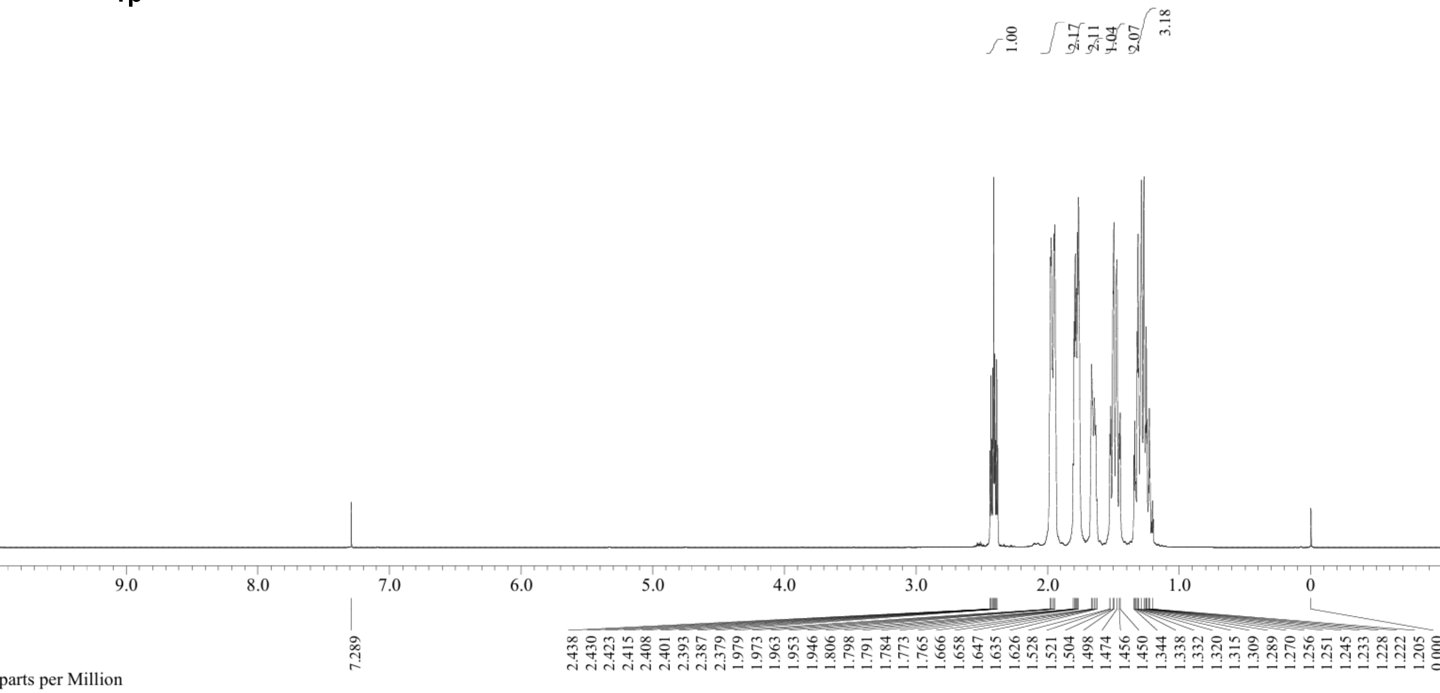

^{1}H NMR (500 MHz, $CDCl_3$)

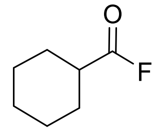

1p

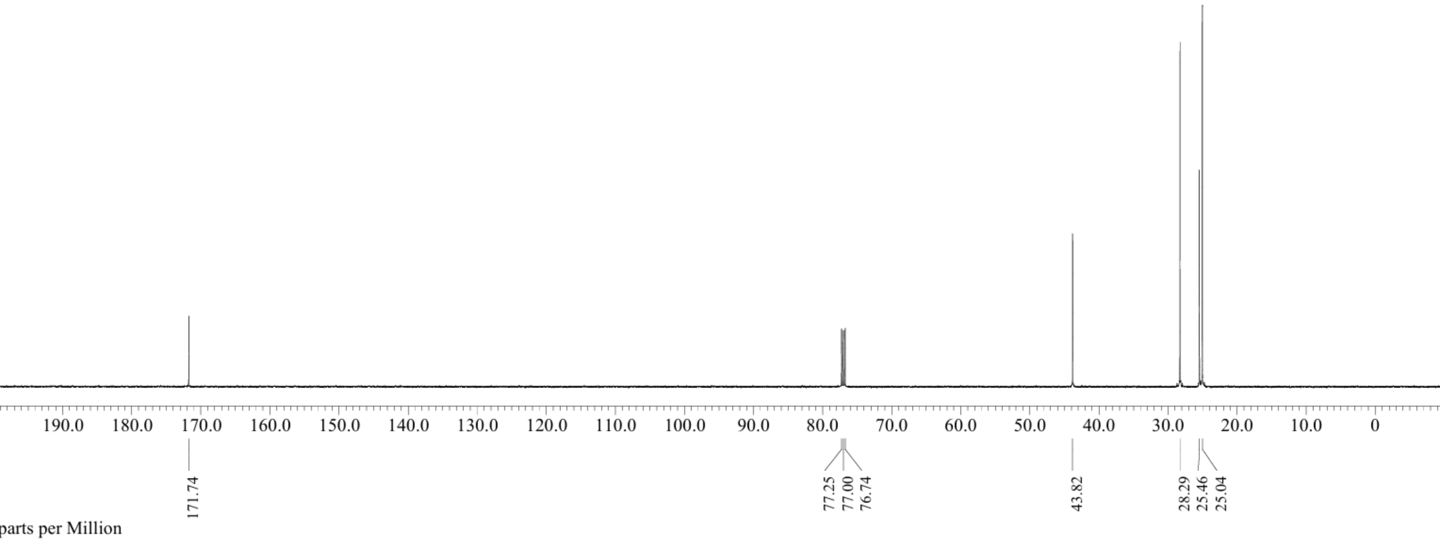

$^{13}C\{^{1}H\}$ NMR (126 MHz, $CDCl_3$)

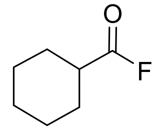

1p

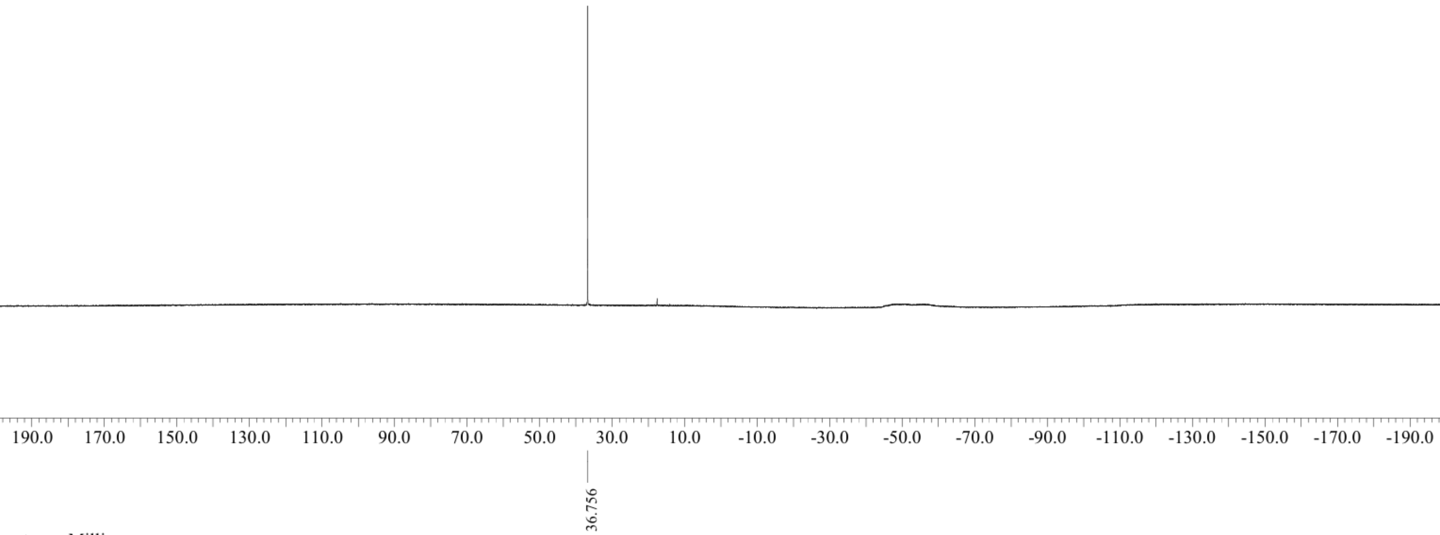

^{19}F NMR (471 MHz, $CDCl_3$)

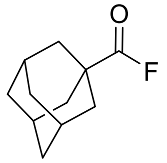

1q

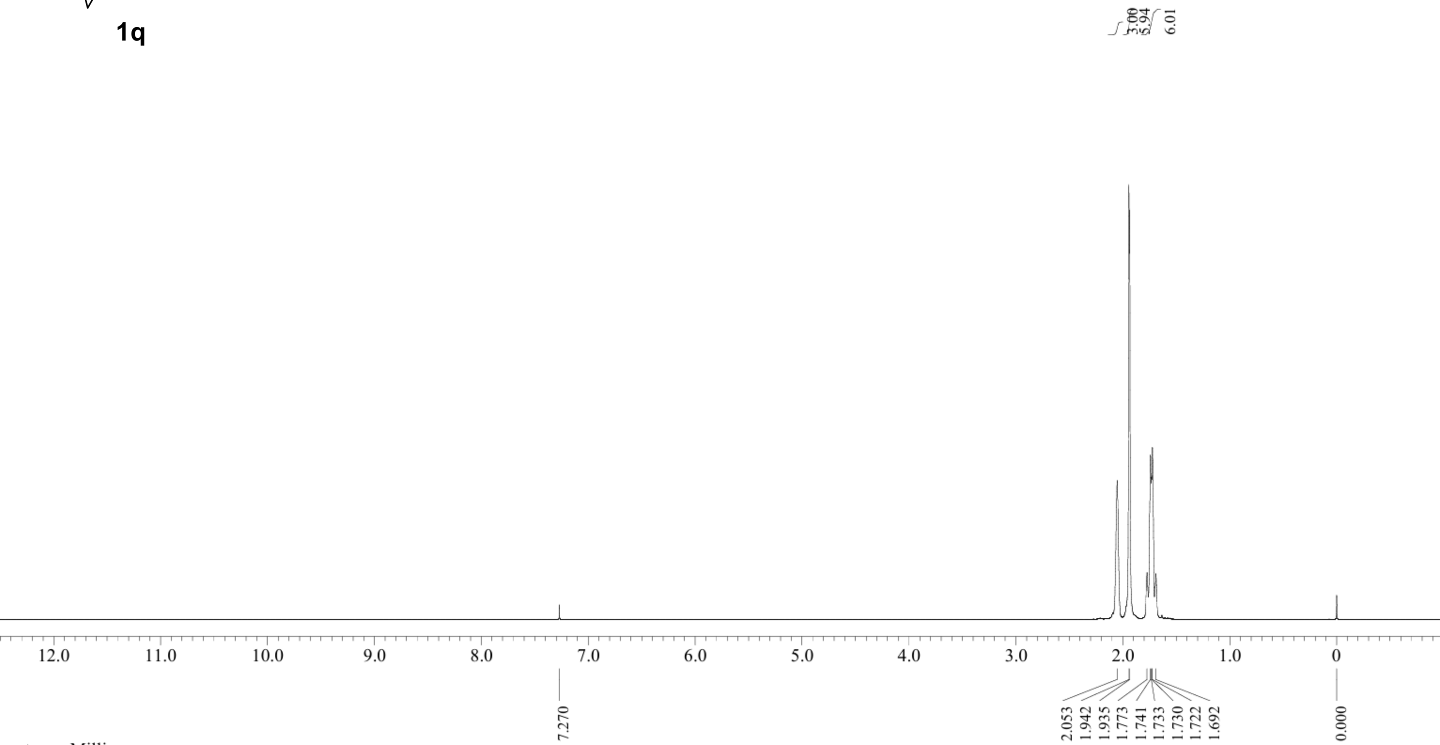

^{1}H NMR (400 MHz, $CDCl_3$)

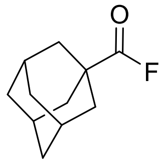

1q

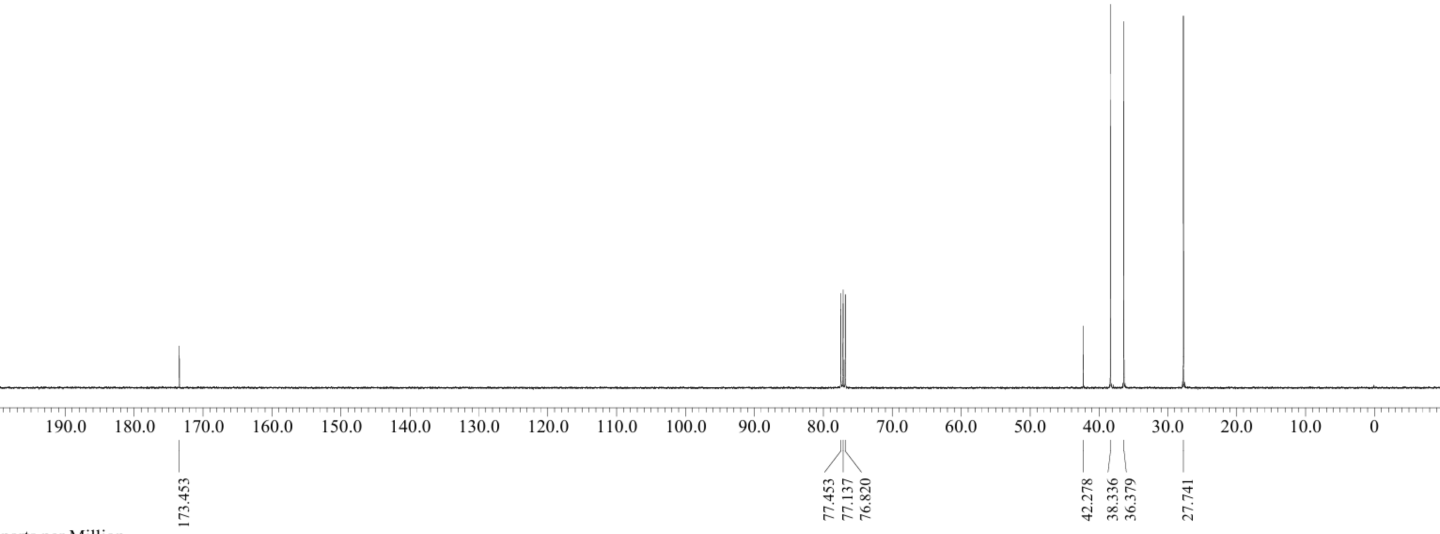

$^{13}C\{^{1}H\}$ NMR (100 MHz, $CDCl_3$)

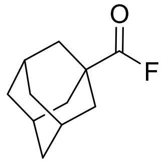

1q

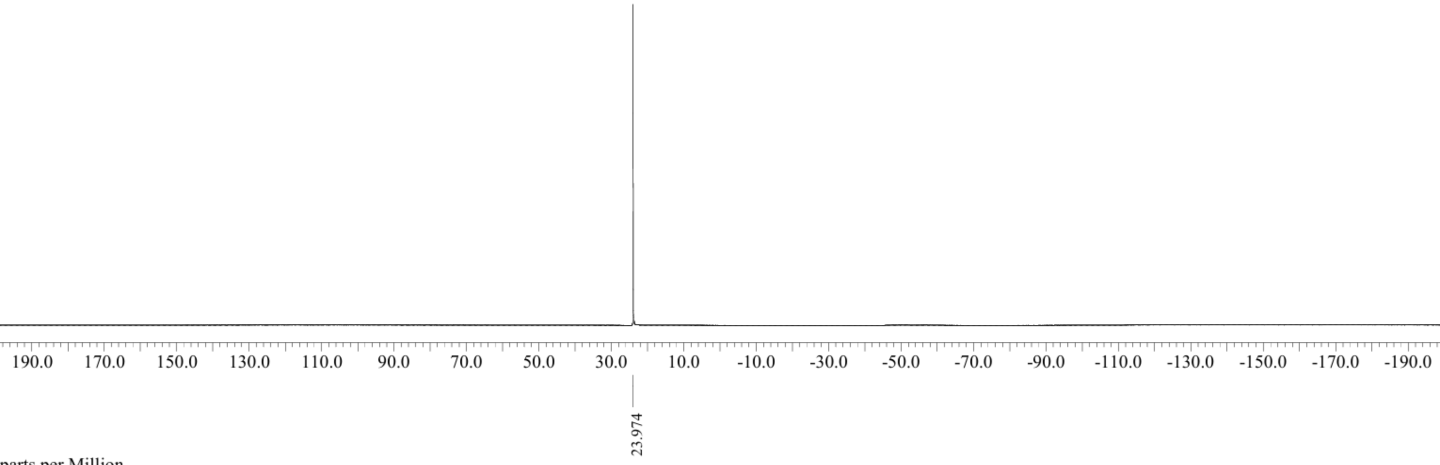

^{19}F NMR (376 MHz, $CDCl_3$)

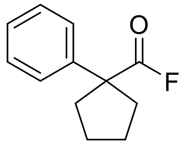

1r

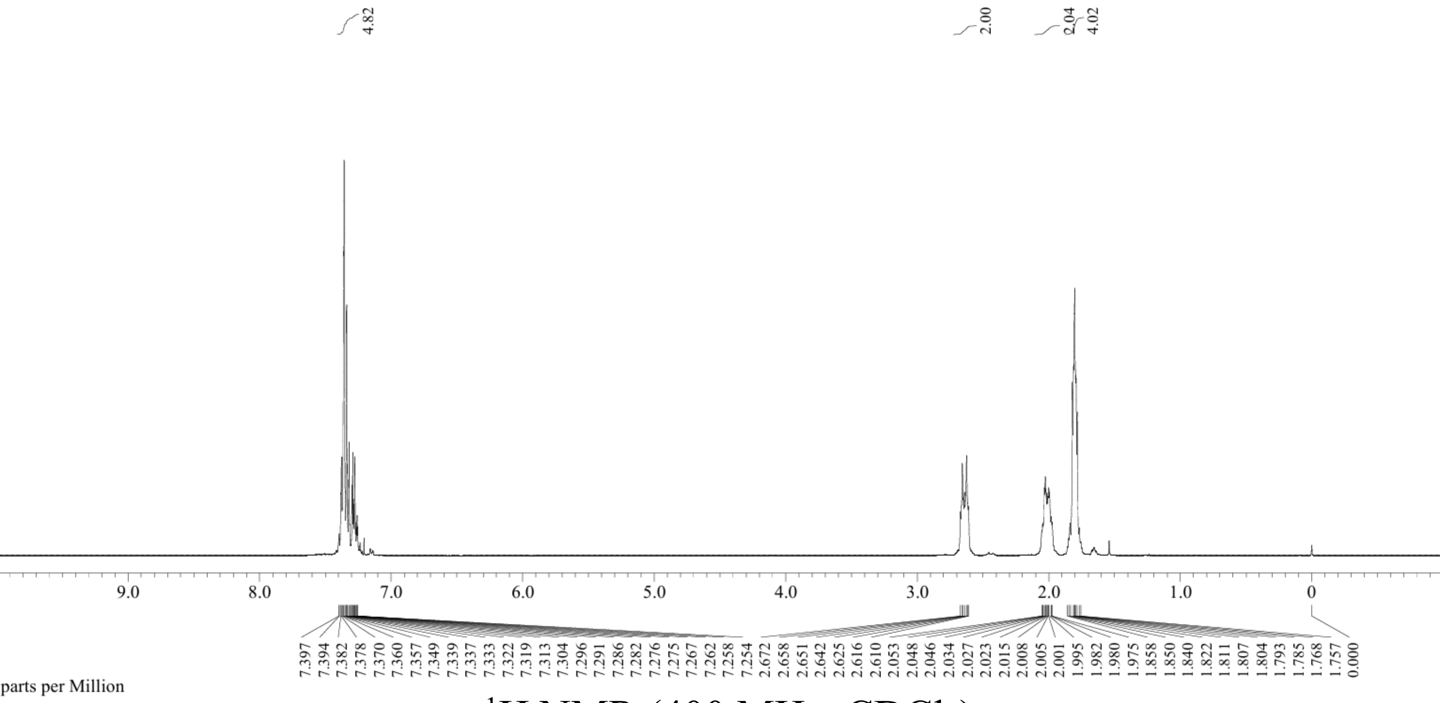

^{1}H NMR (400 MHz, $CDCl_3$)

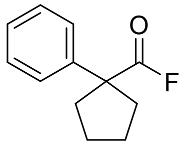

1r

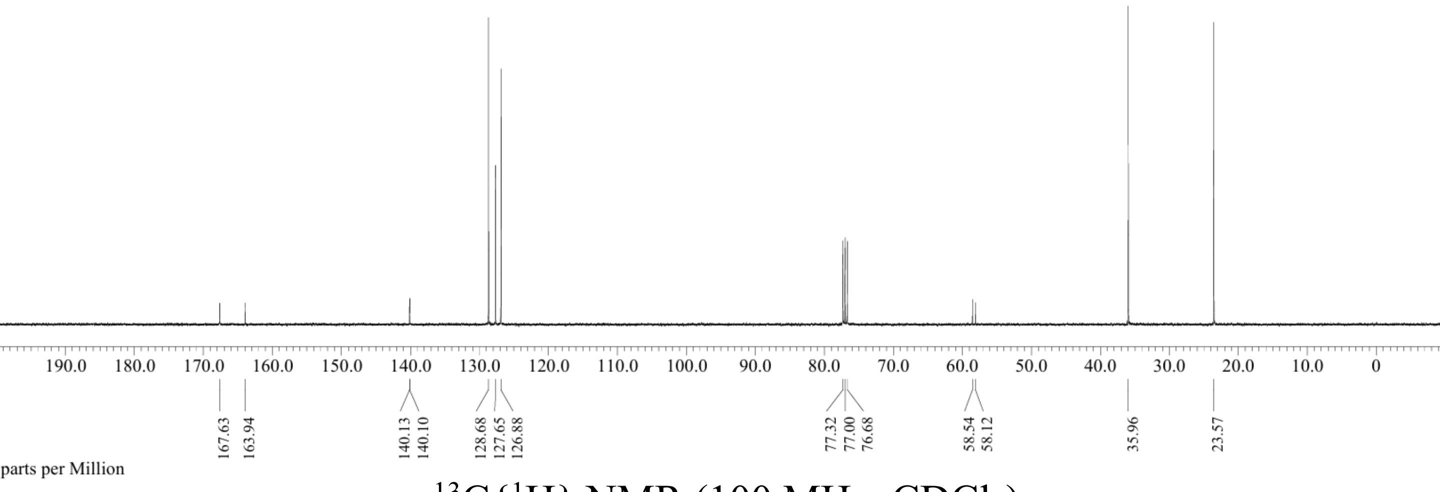

$^{13}C\{^{1}H\}$ NMR (100 MHz, $CDCl_3$)

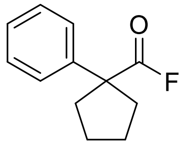

1r

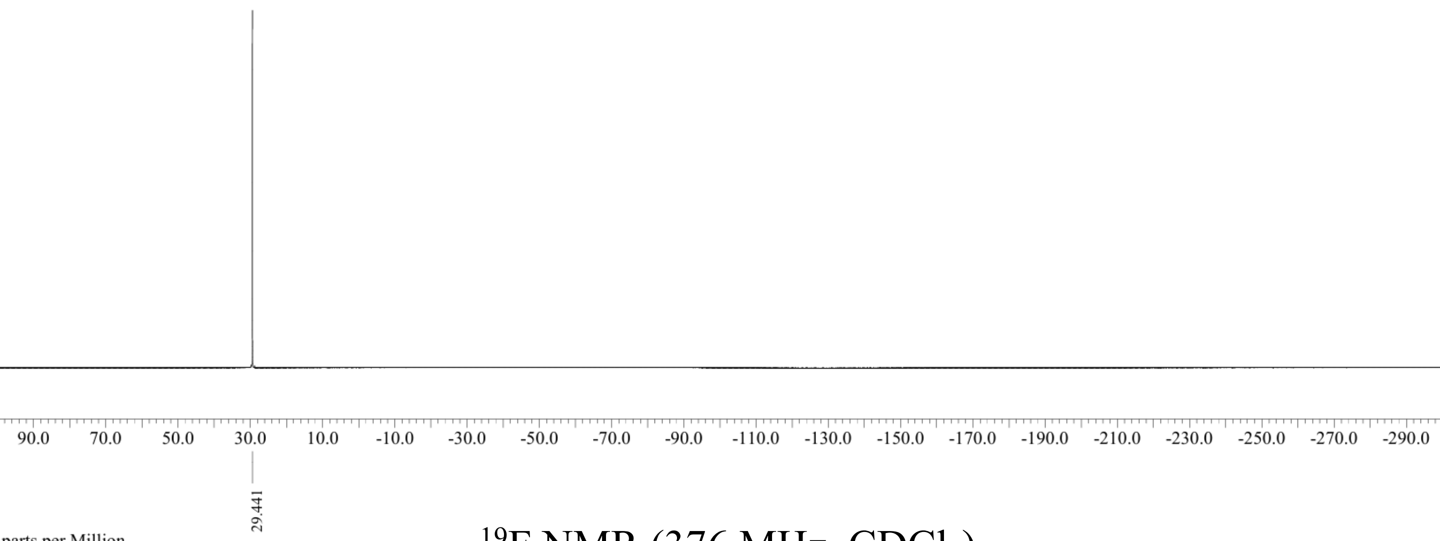

^{19}F NMR (376 MHz, $CDCl_3$)

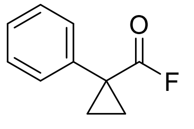

1s

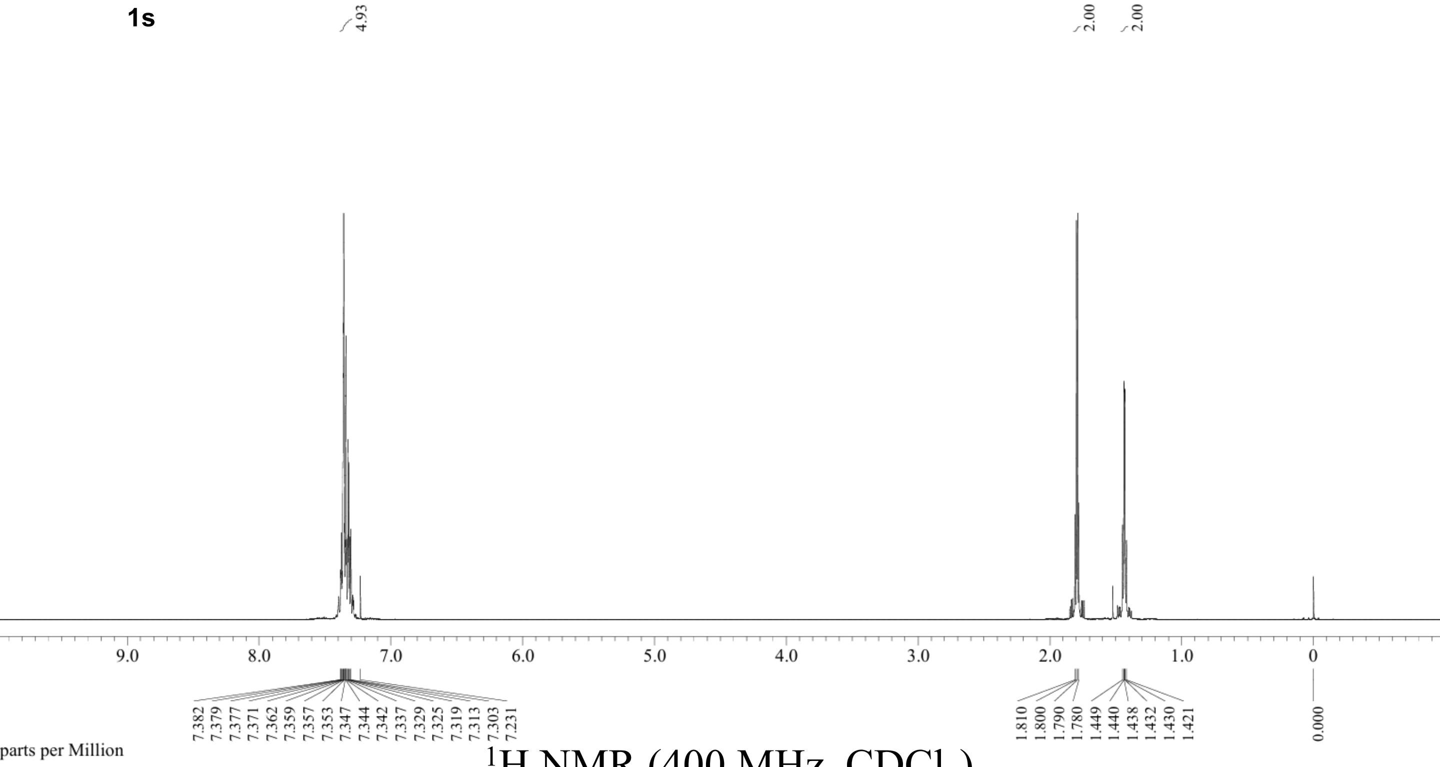

^{1}H NMR (400 MHz, $CDCl_3$)

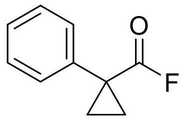

1s

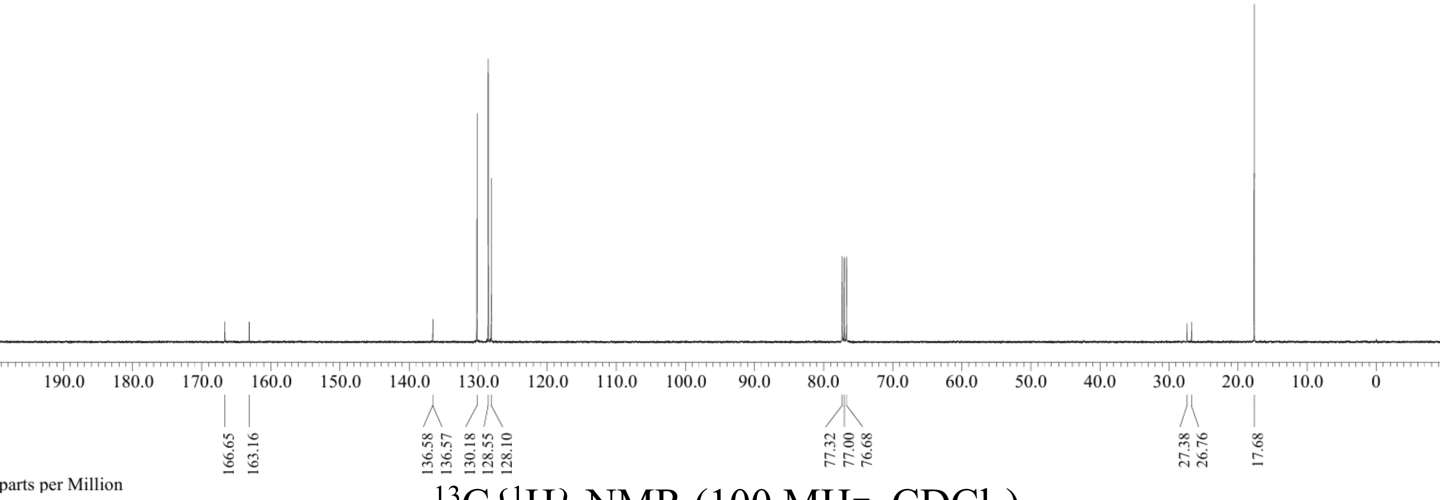

$^{13}C\{^{1}H\}$ NMR (100 MHz, $CDCl_3$)

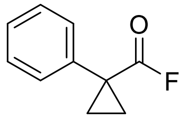

1s

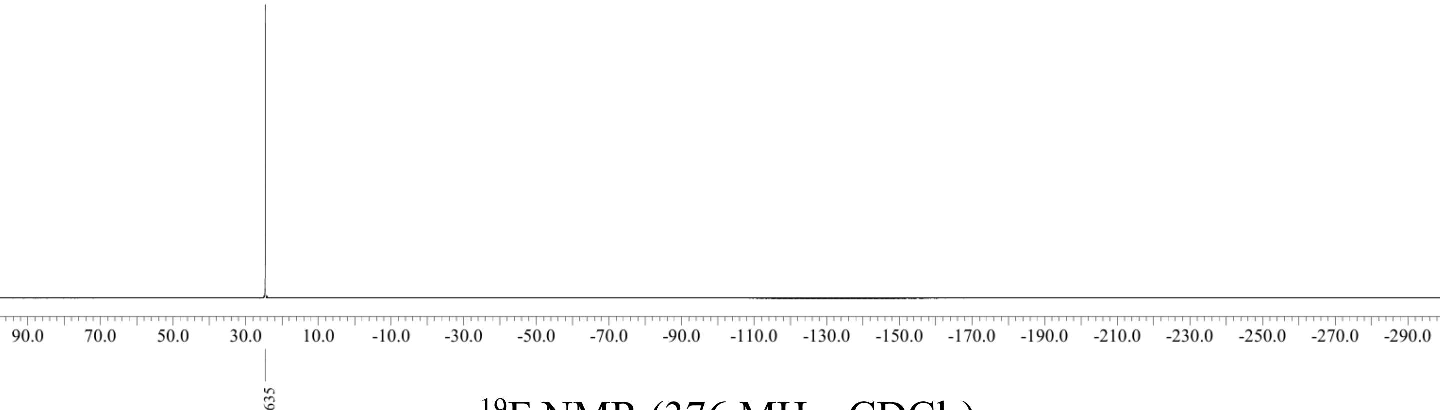

^{19}F NMR (376 MHz, $CDCl_3$)

parts per Million

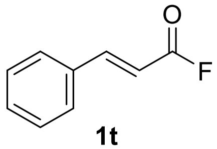

1t

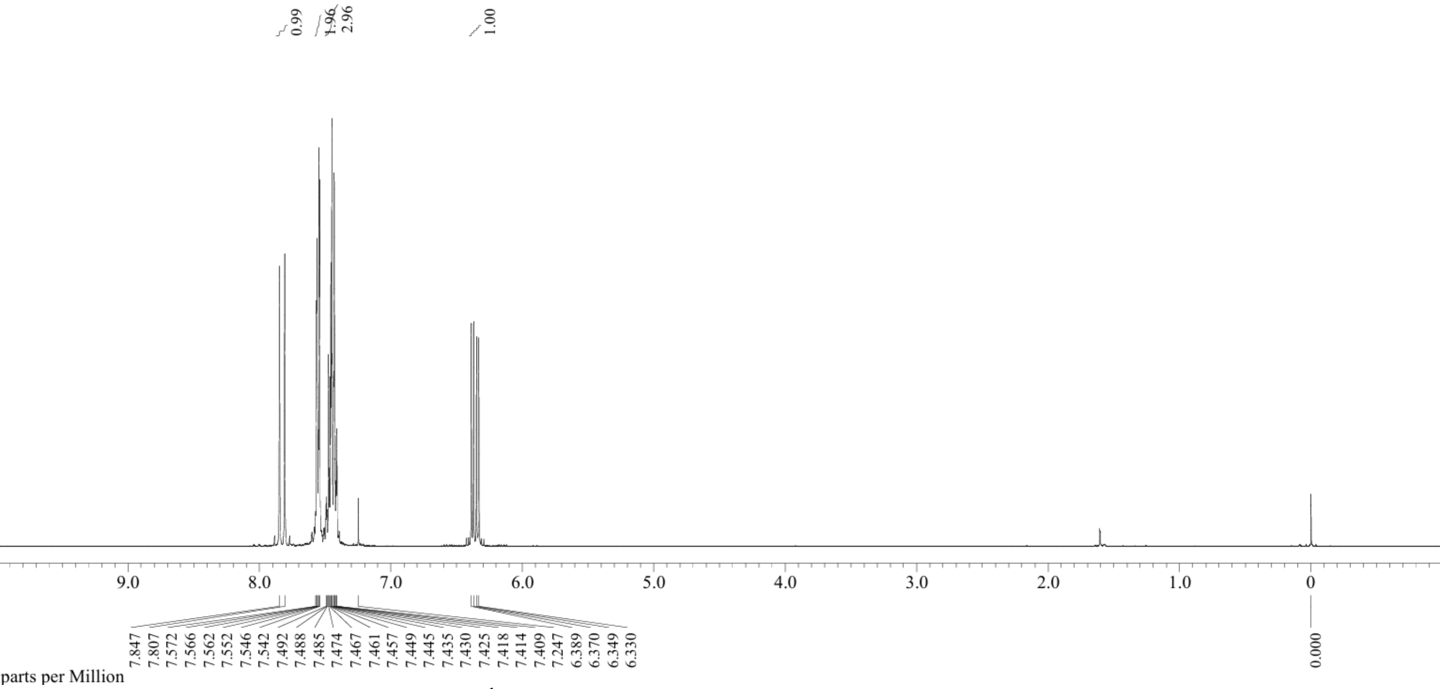

^{1}H NMR (400 MHz, $CDCl_3$)

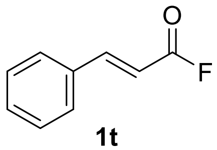

1t

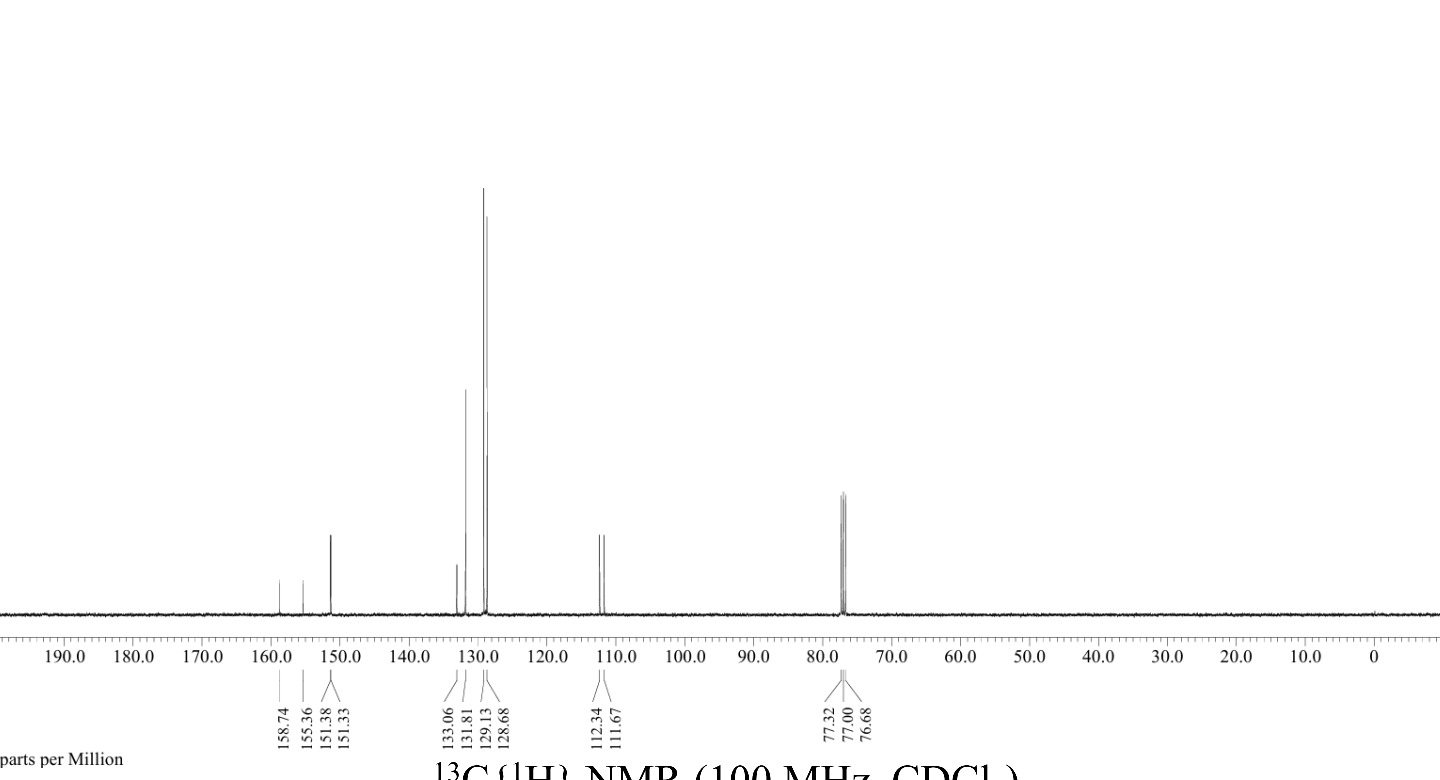

$^{13}C\{^{1}H\}$ NMR (100 MHz, $CDCl_3$)

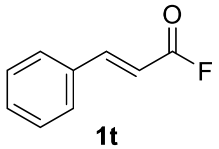

1t

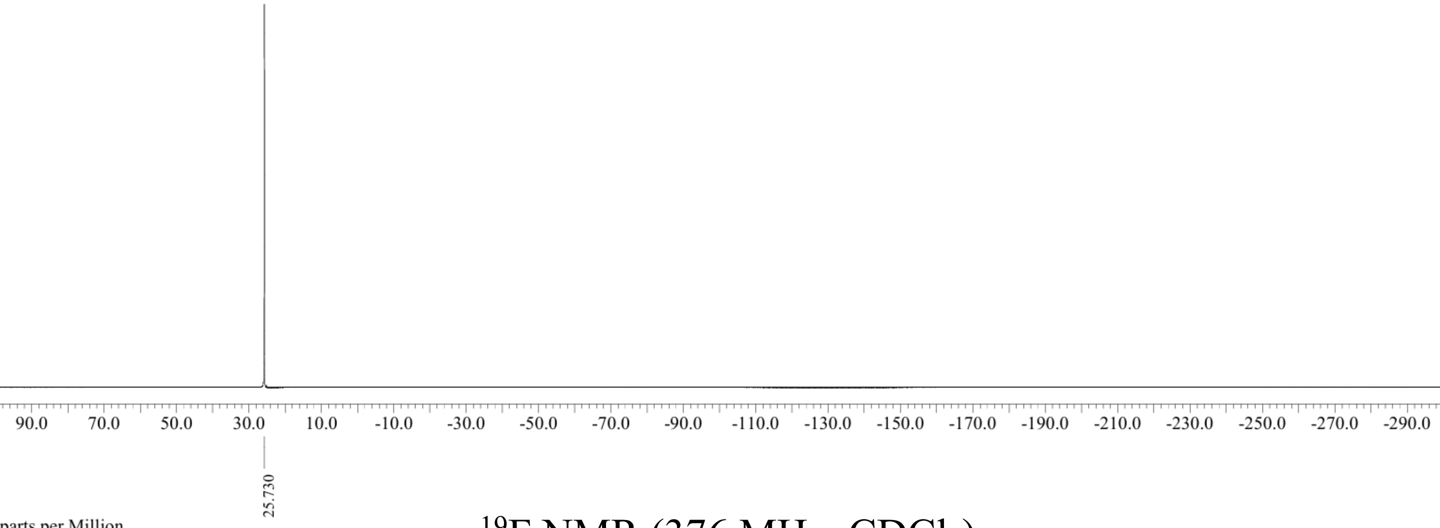

^{19}F NMR (376 MHz, $CDCl_3$)

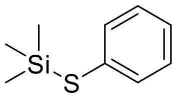

2a

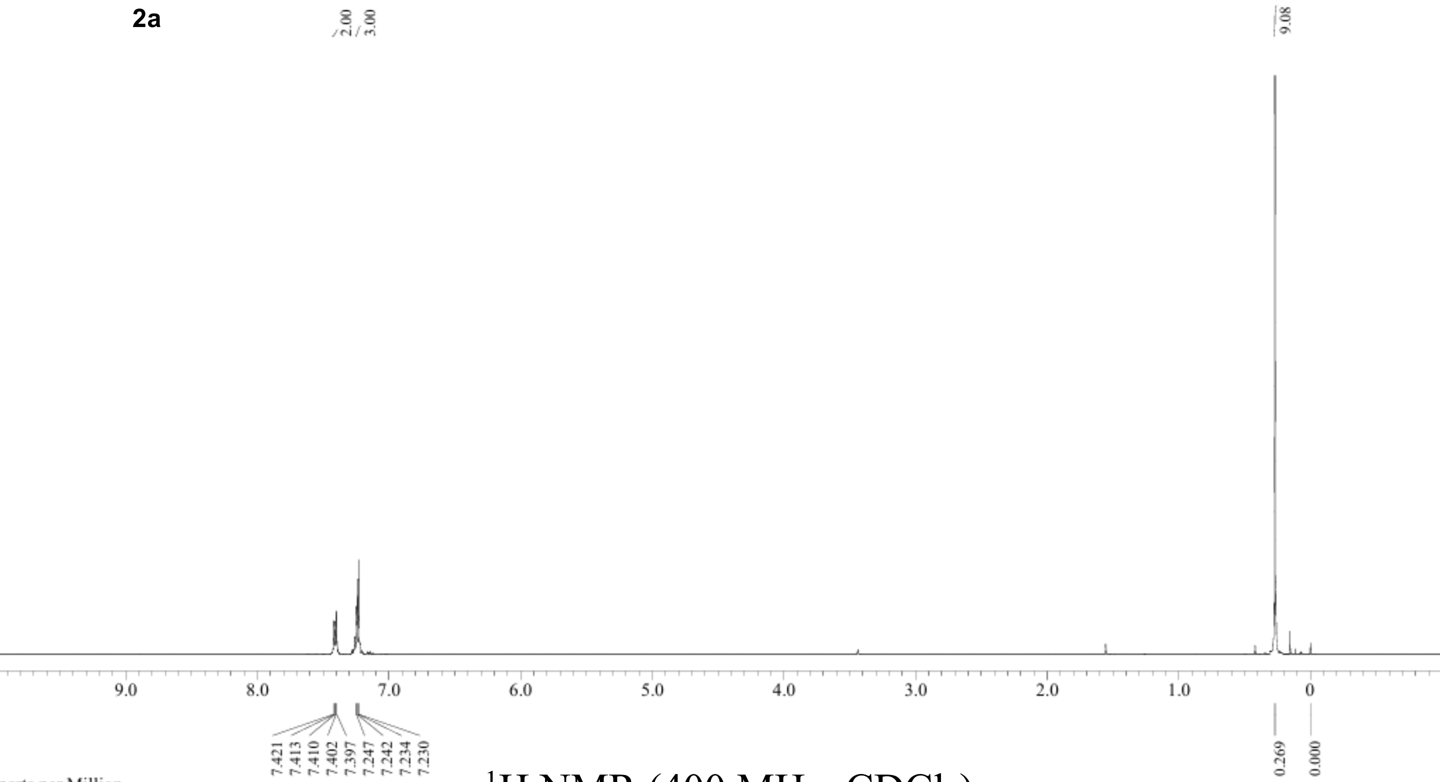

^{1}H NMR (400 MHz, $CDCl_3$)

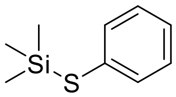

2a

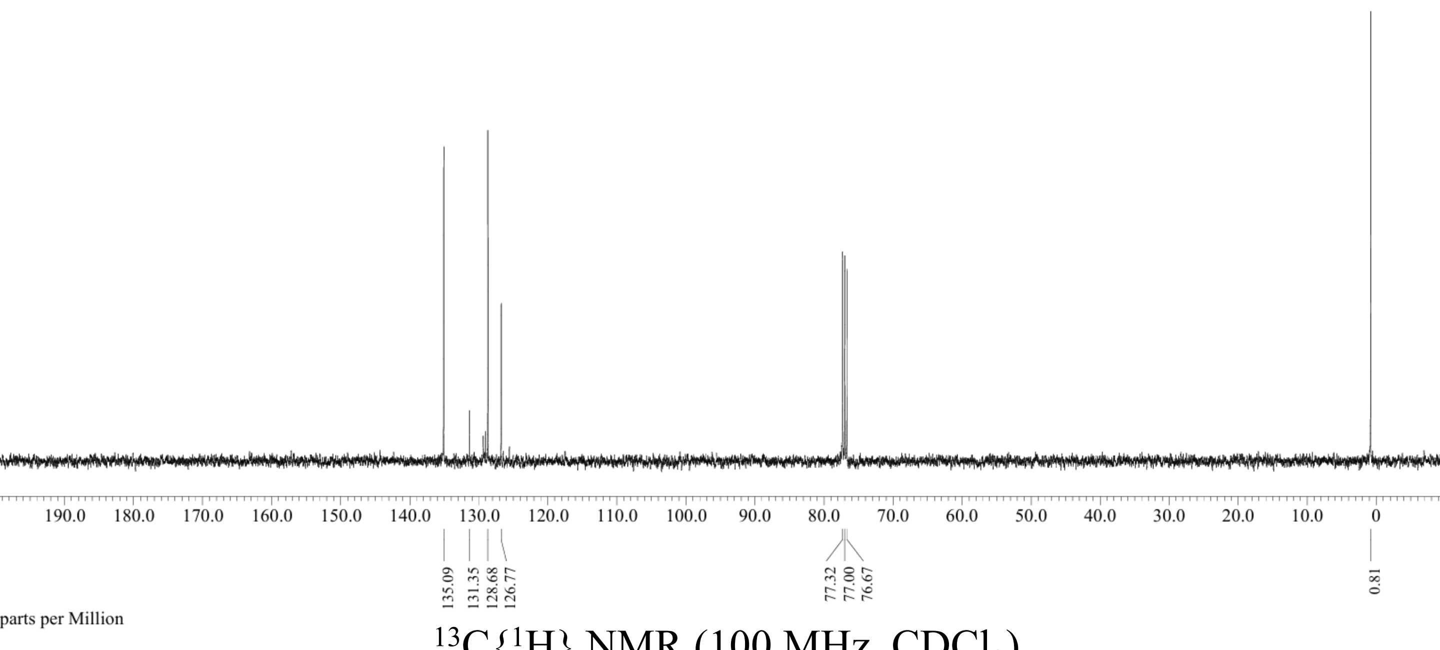

$^{13}C\{^{1}H\}$ NMR (100 MHz, $CDCl_3$)

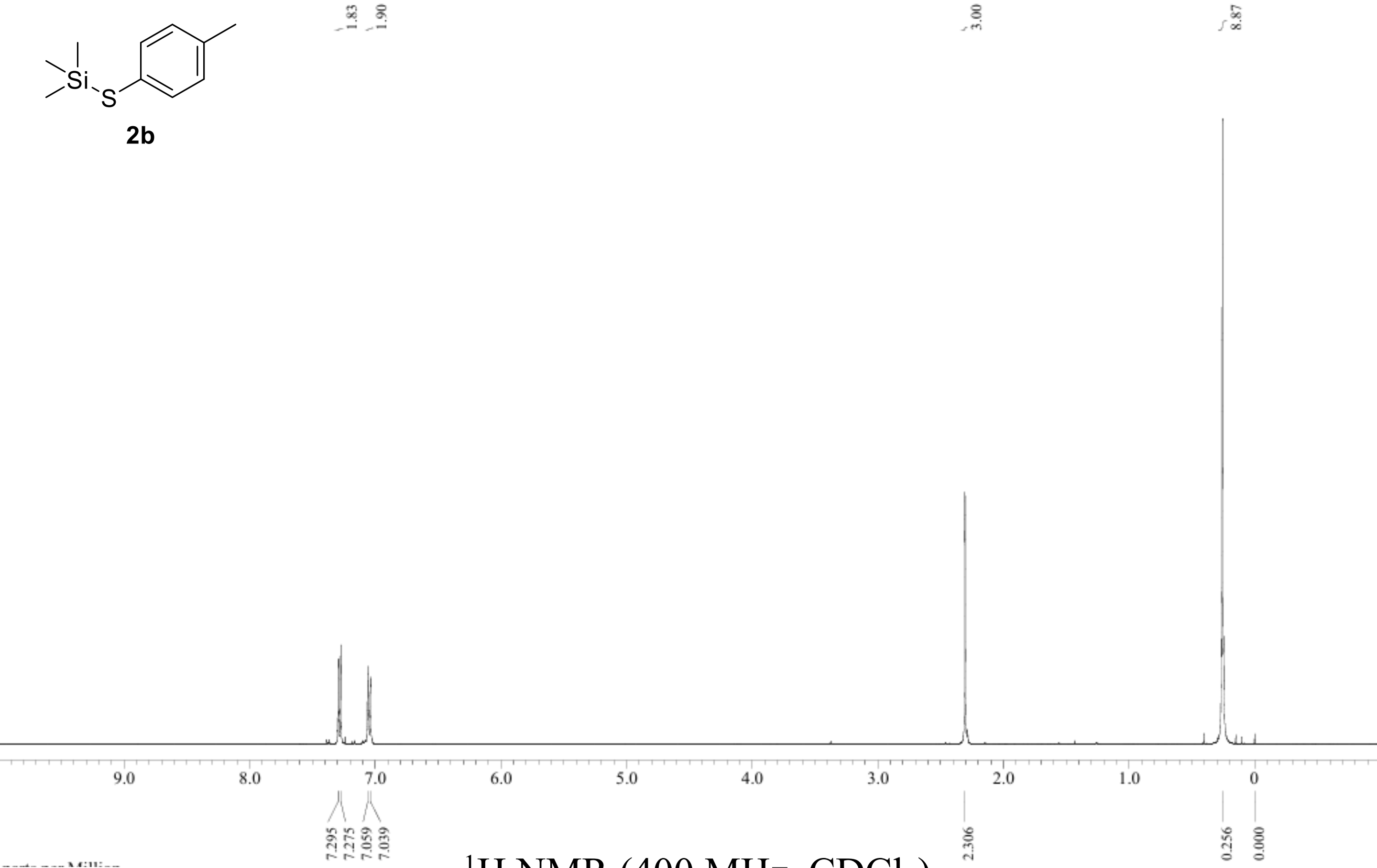

^{1}H NMR (400 MHz, $CDCl_3$)

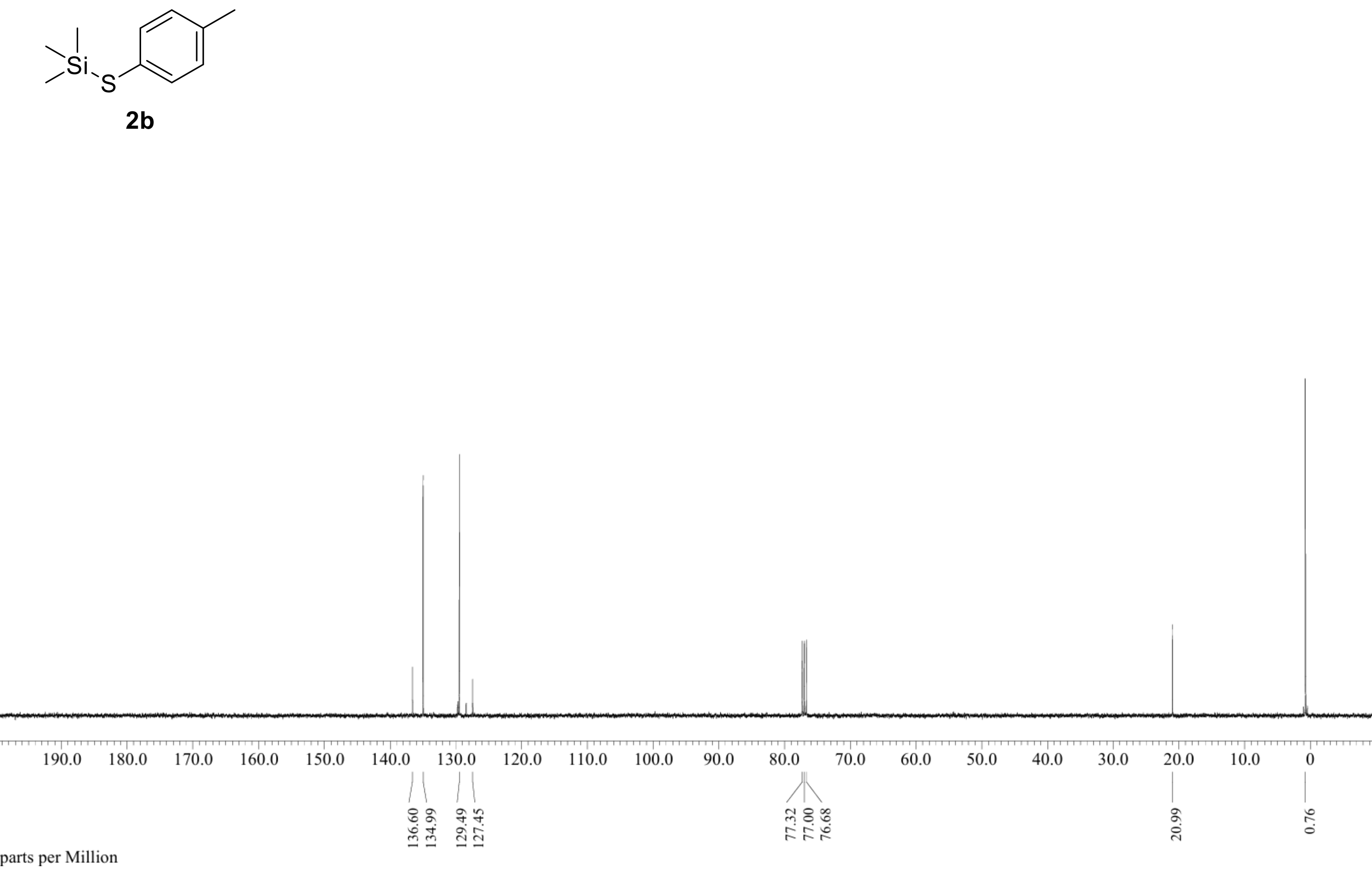

$^{13}C\{^{1}H\}$ NMR (100 MHz, $CDCl_3$)

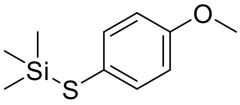

2c

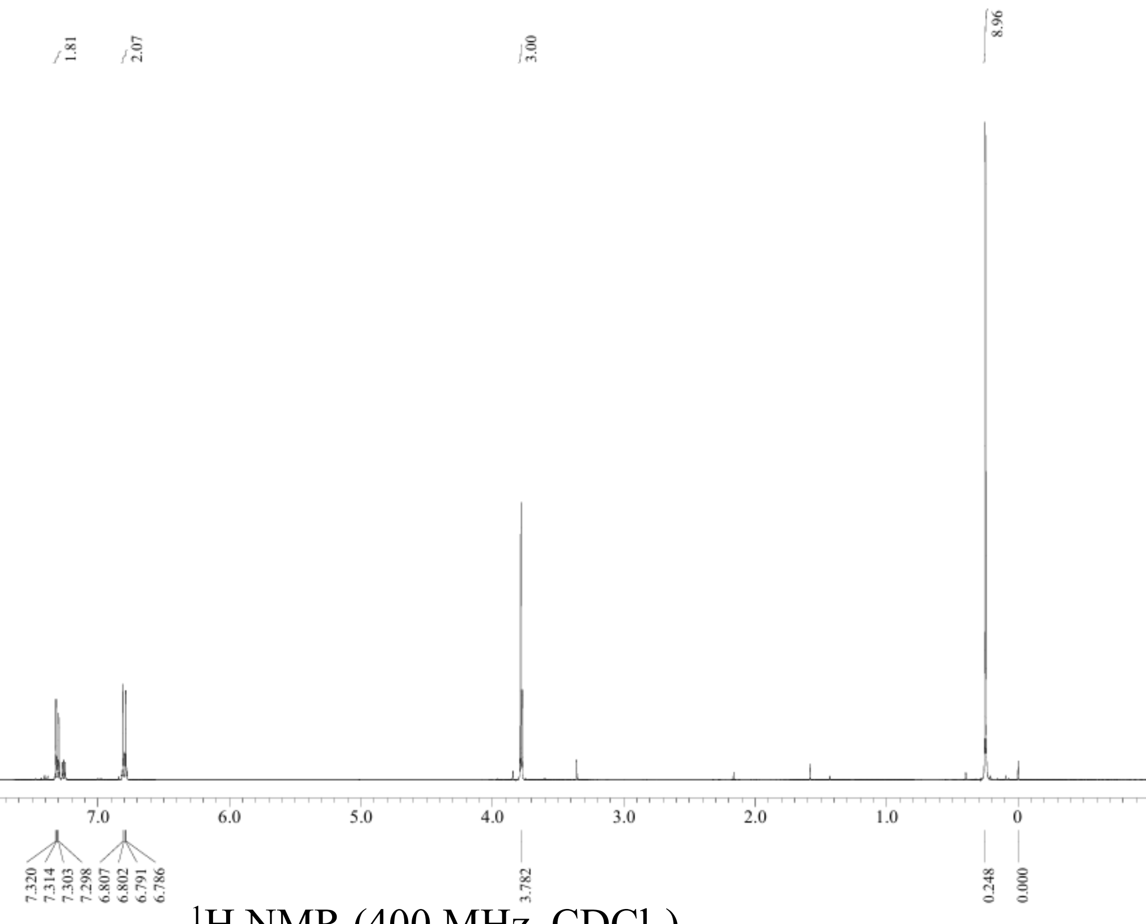

^{1}H NMR (400 MHz, $CDCl_3$)

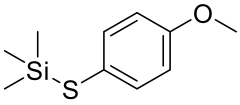

2c

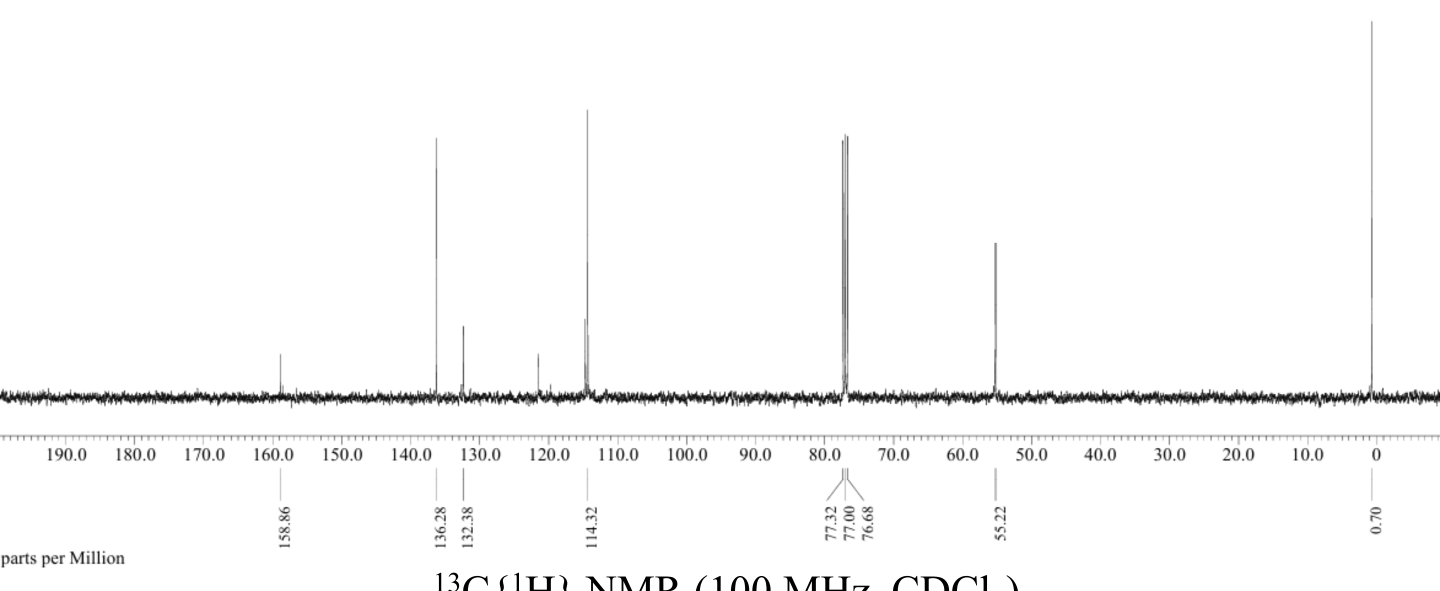

$^{13}C\{^{1}H\}$ NMR (100 MHz, $CDCl_3$)

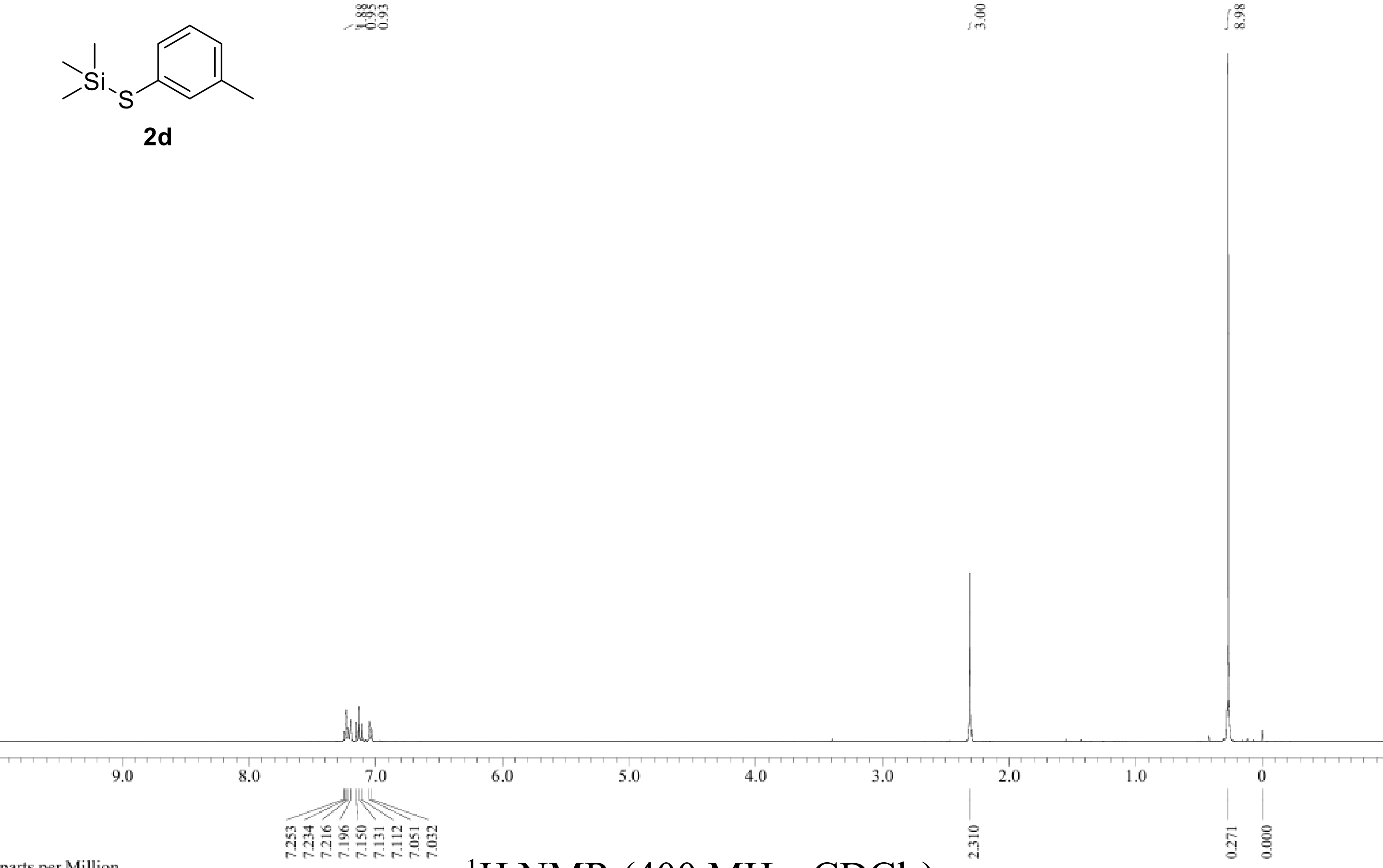

^{1}H NMR (400 MHz, $CDCl_3$)

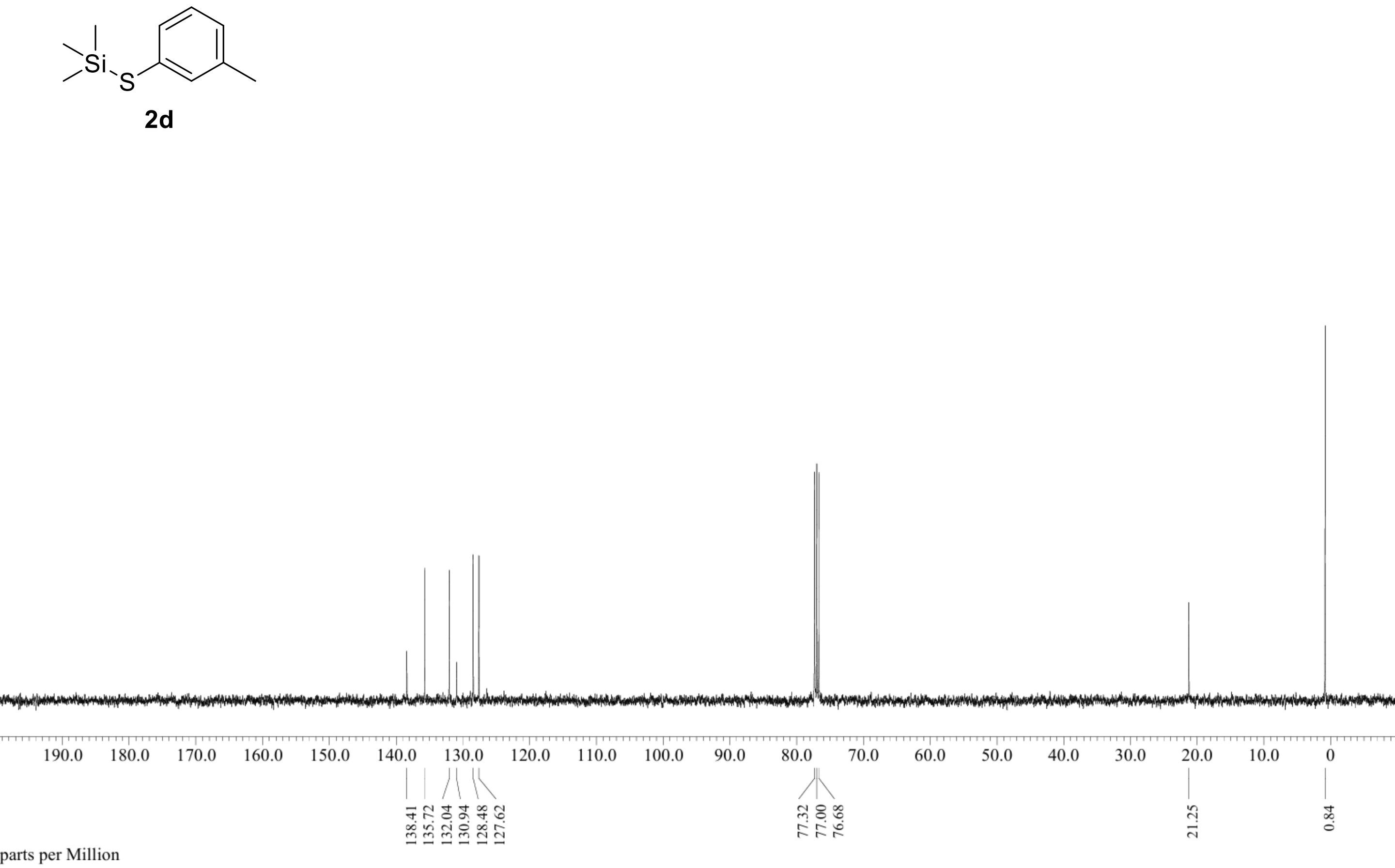

$^{13}C\{^{1}H\}$ NMR (100 MHz, $CDCl_3$)

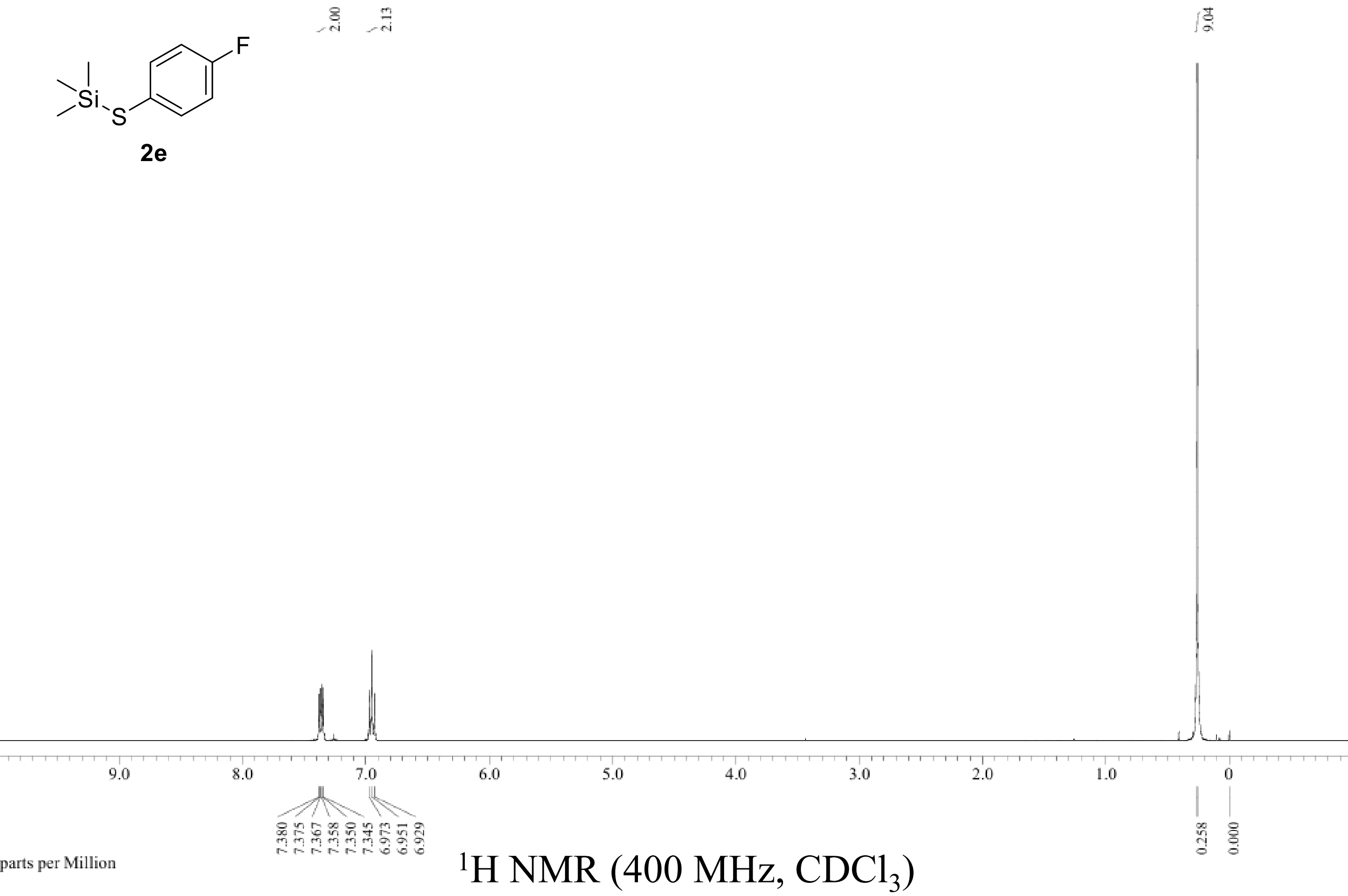

^{1}H NMR (400 MHz, $CDCl_3$)

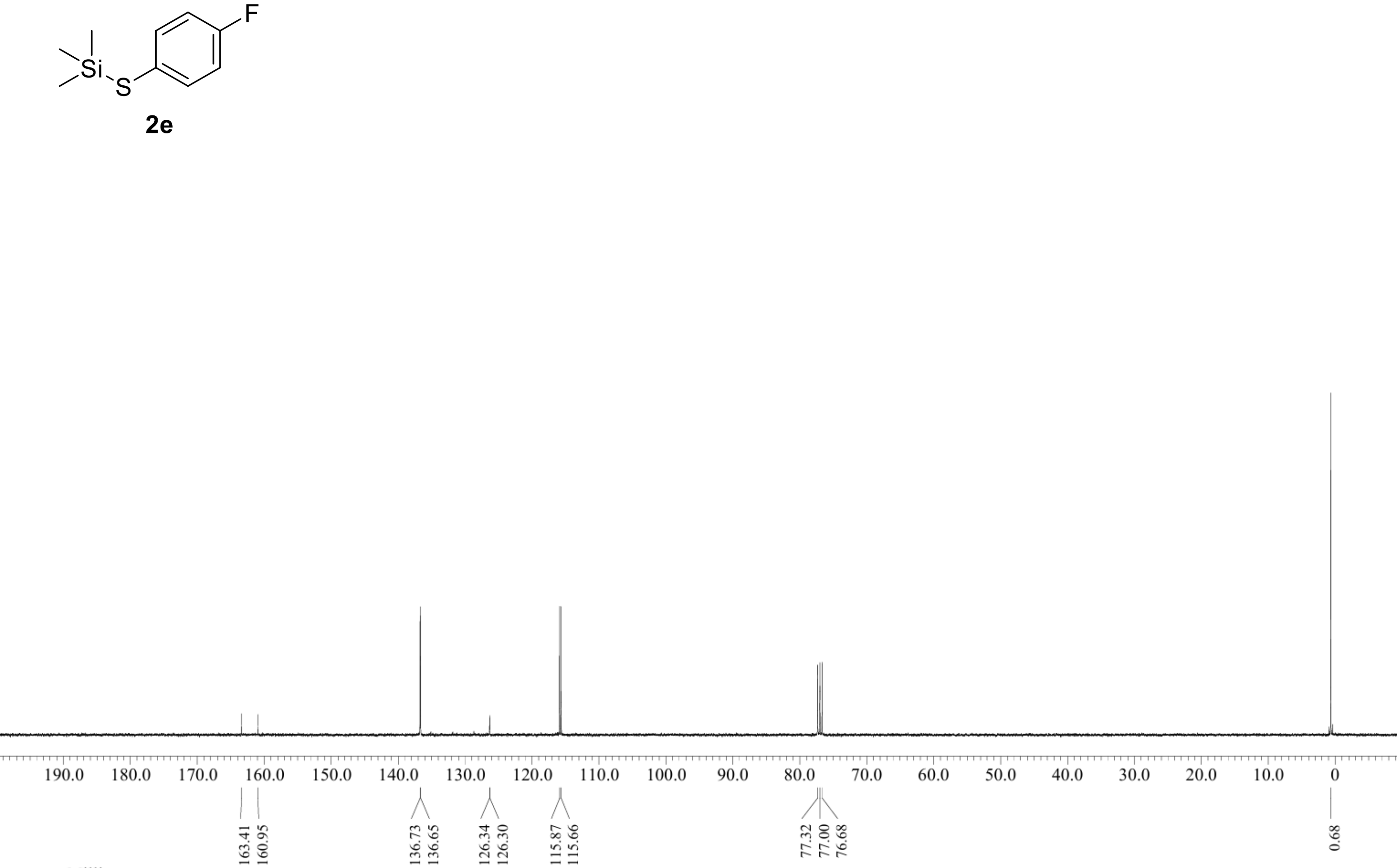

$^{13}C\{^{1}H\}$ NMR (100 MHz, $CDCl_3$)

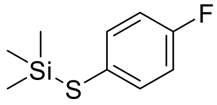

2e

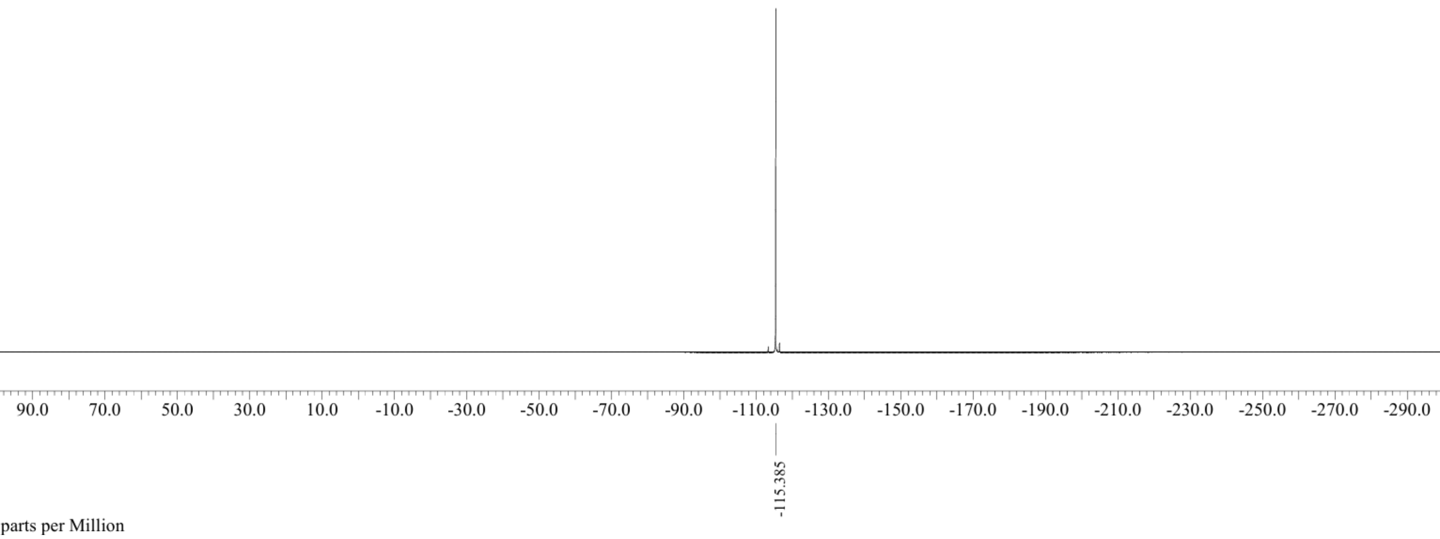

^{19}F NMR (376 MHz, $CDCl_3$)

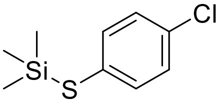

2f

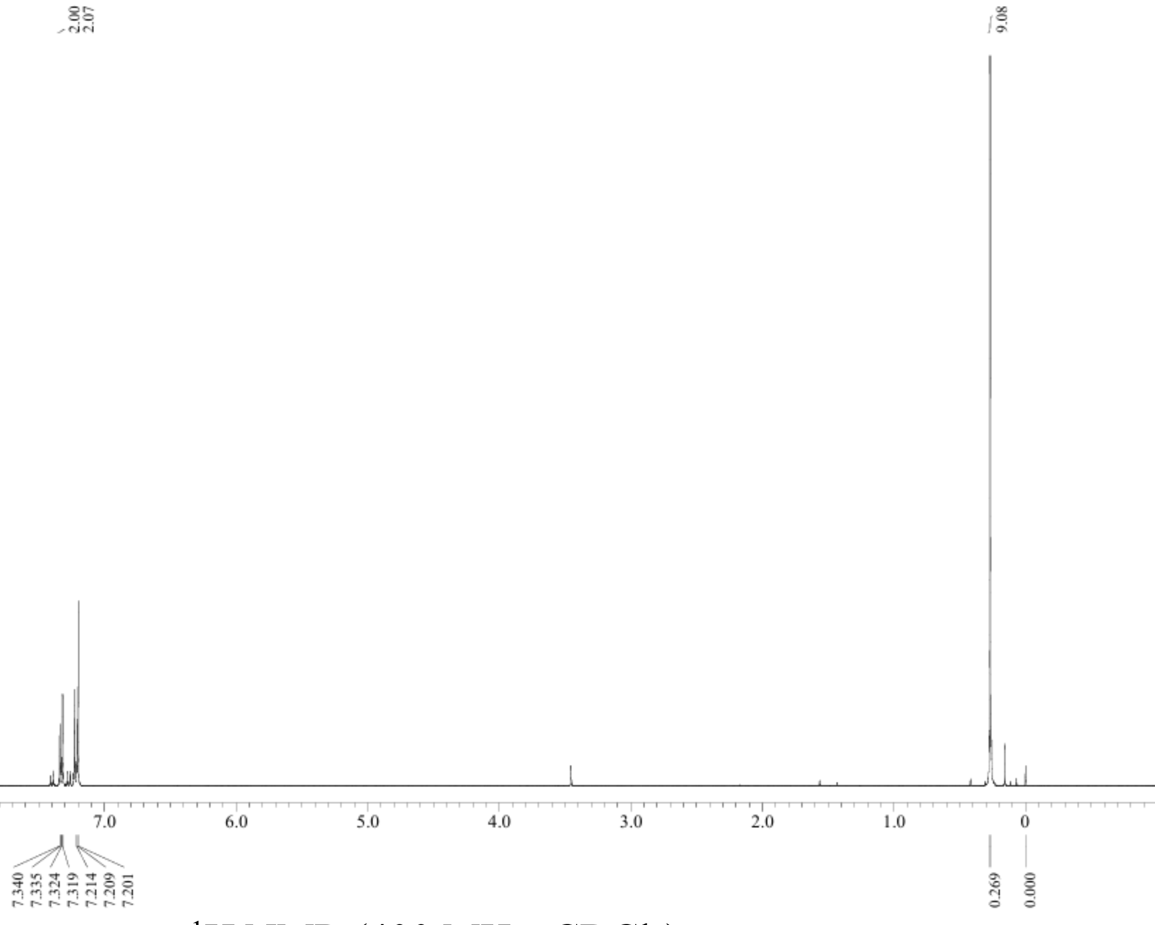

^{1}H NMR (400 MHz, $CDCl_3$)

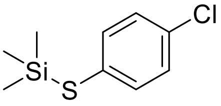

2f

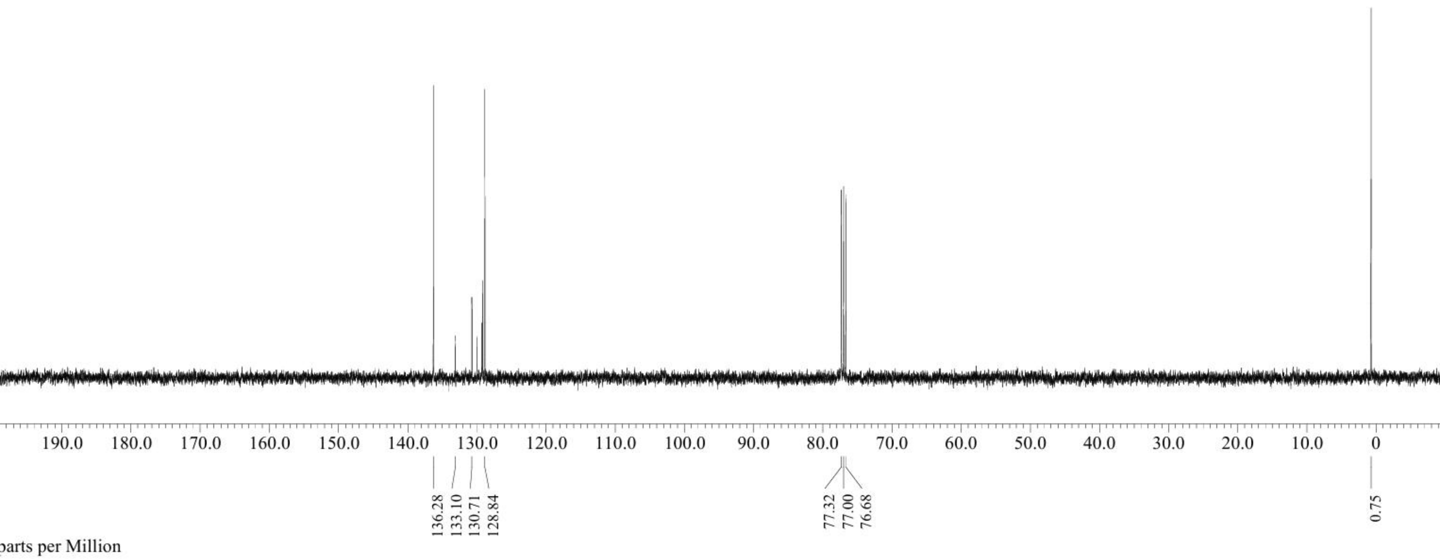

$^{13}C\{^{1}H\}$ NMR (100 MHz, $CDCl_3$)

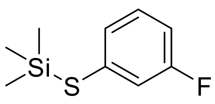

2g

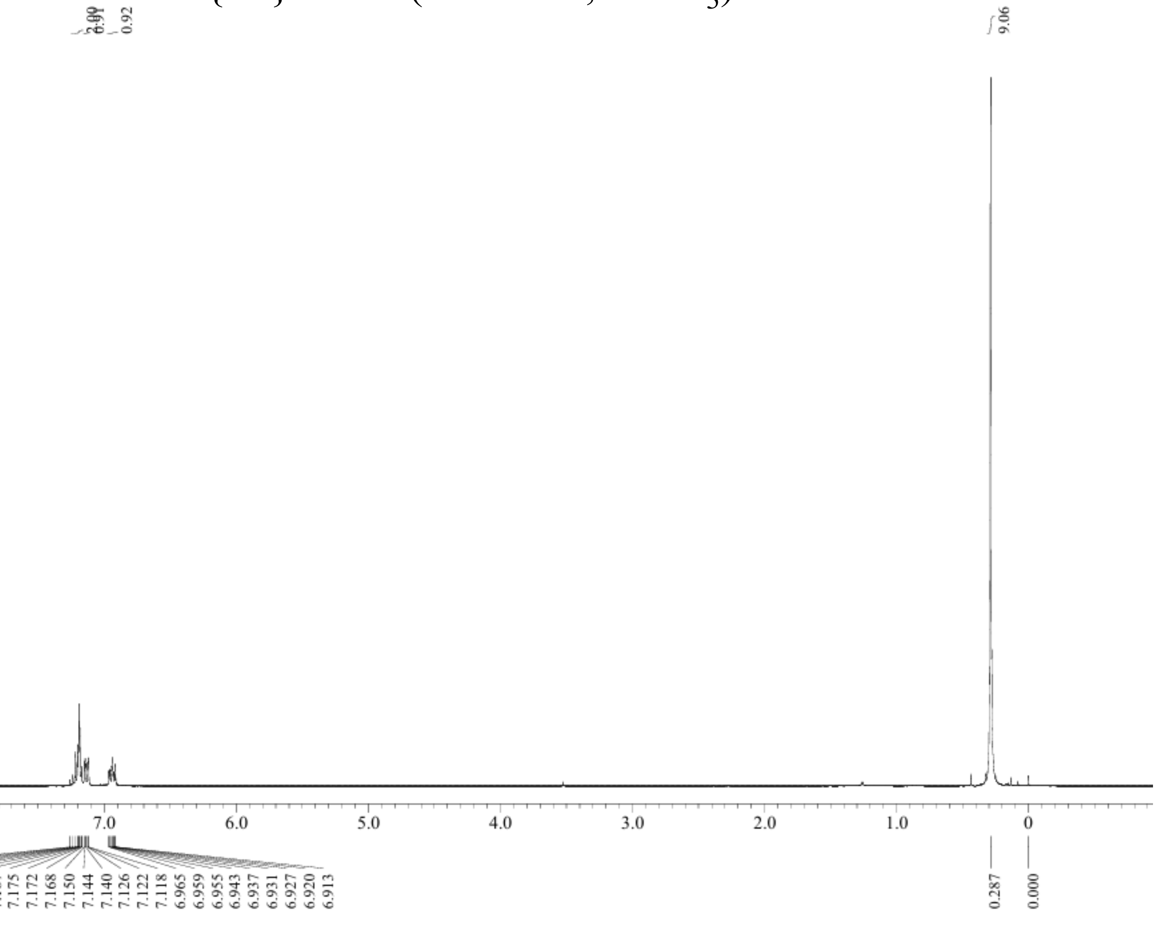

^{1}H NMR (400 MHz, $CDCl_3$)

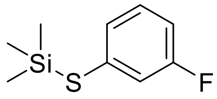

2g

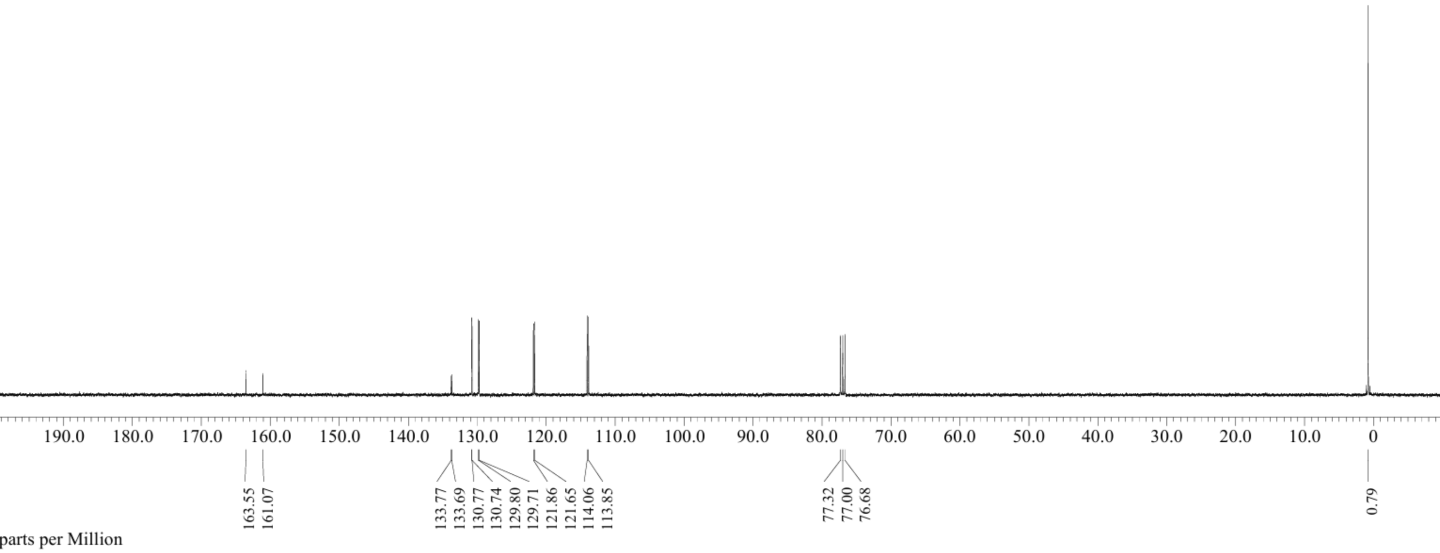

$^{13}C\{^{1}H\}$ NMR (100 MHz, $CDCl_3$)

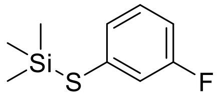

2g

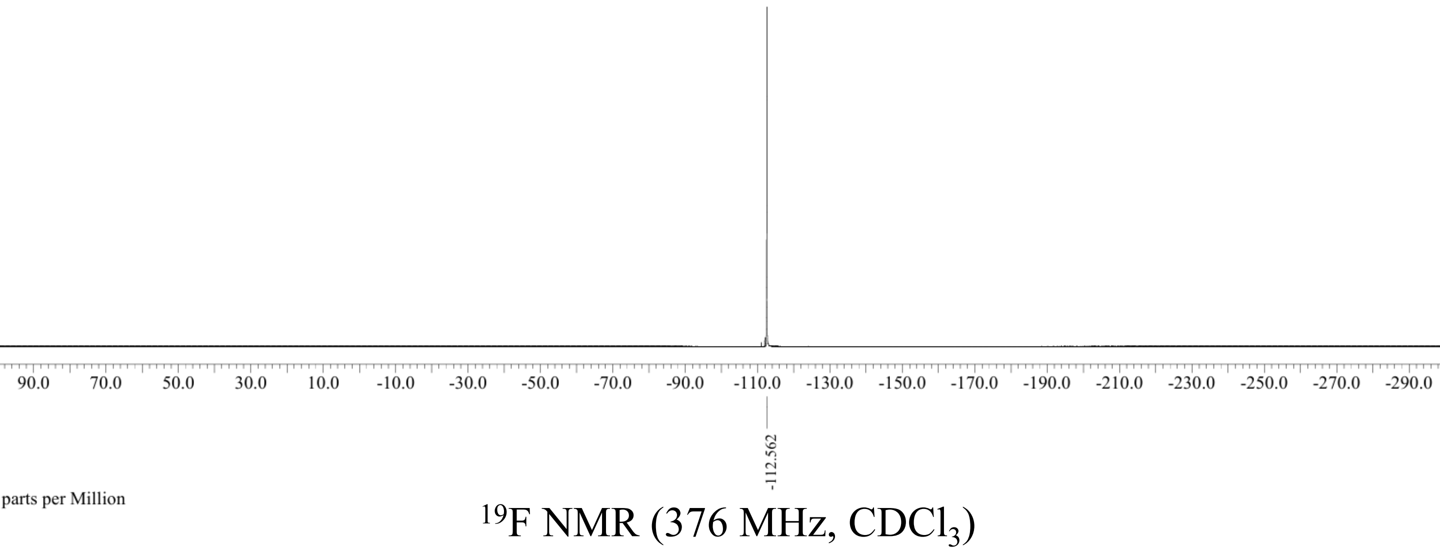

^{19}F NMR (376 MHz, $CDCl_3$)

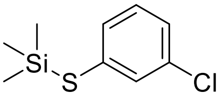

2h

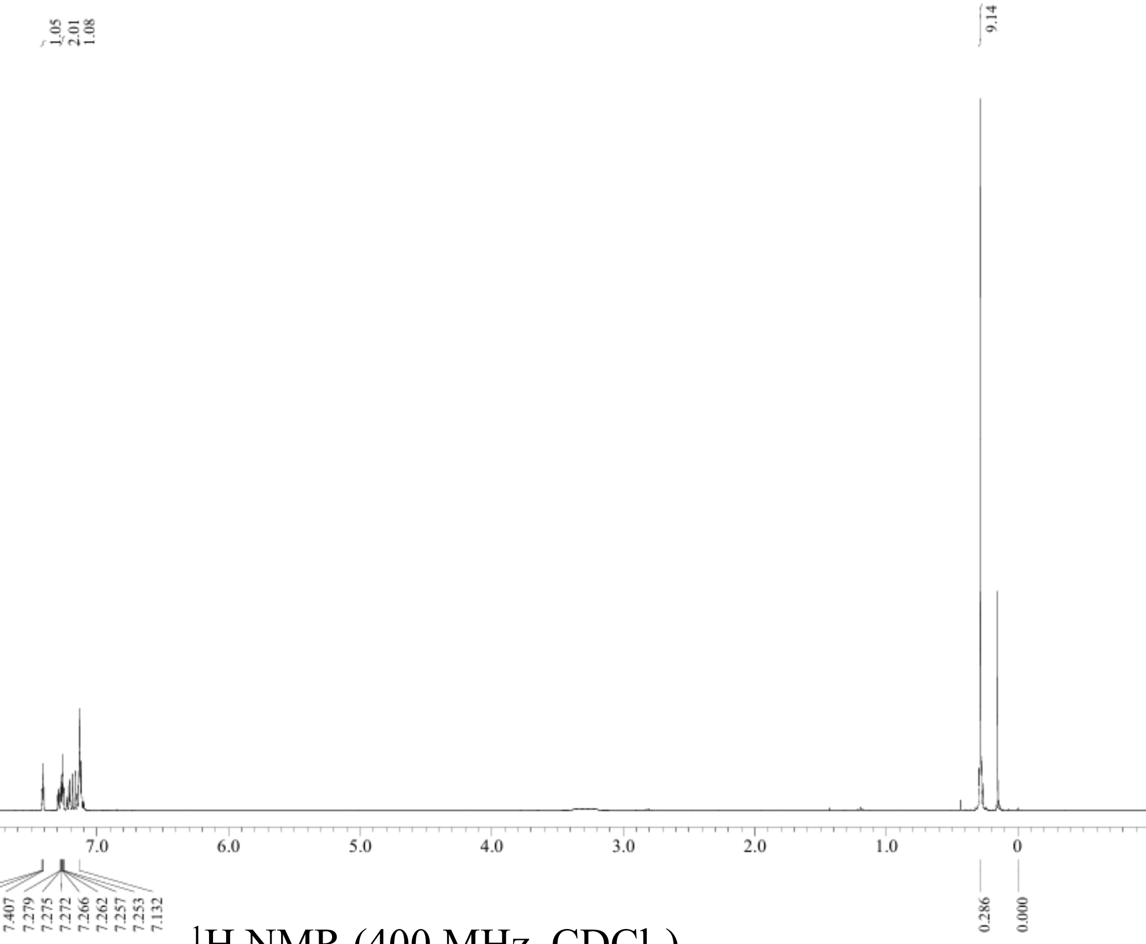

^{1}H NMR (400 MHz, $CDCl_3$)

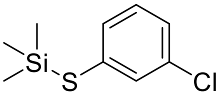

2h

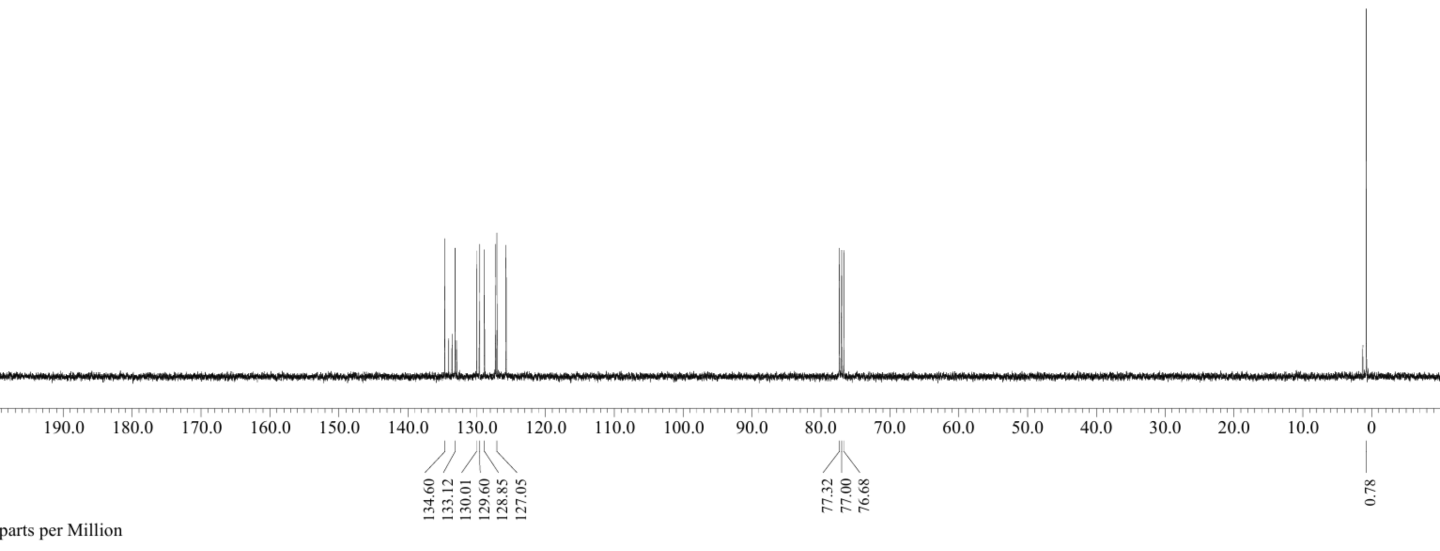

$^{13}C\{^{1}H\}$ NMR (100 MHz, $CDCl_3$)

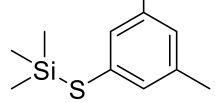

2i

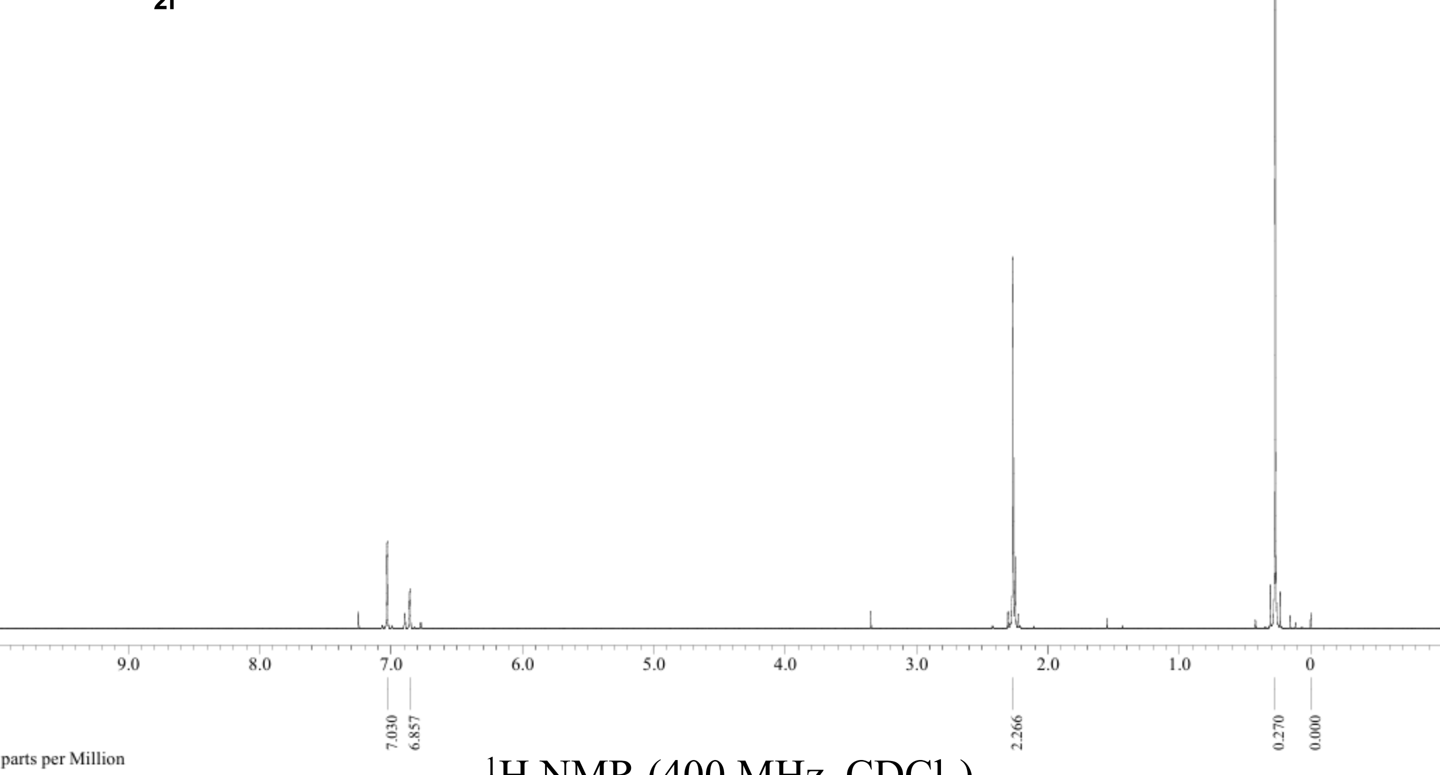

^{1}H NMR (400 MHz, $CDCl_3$)

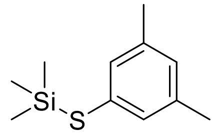

2i

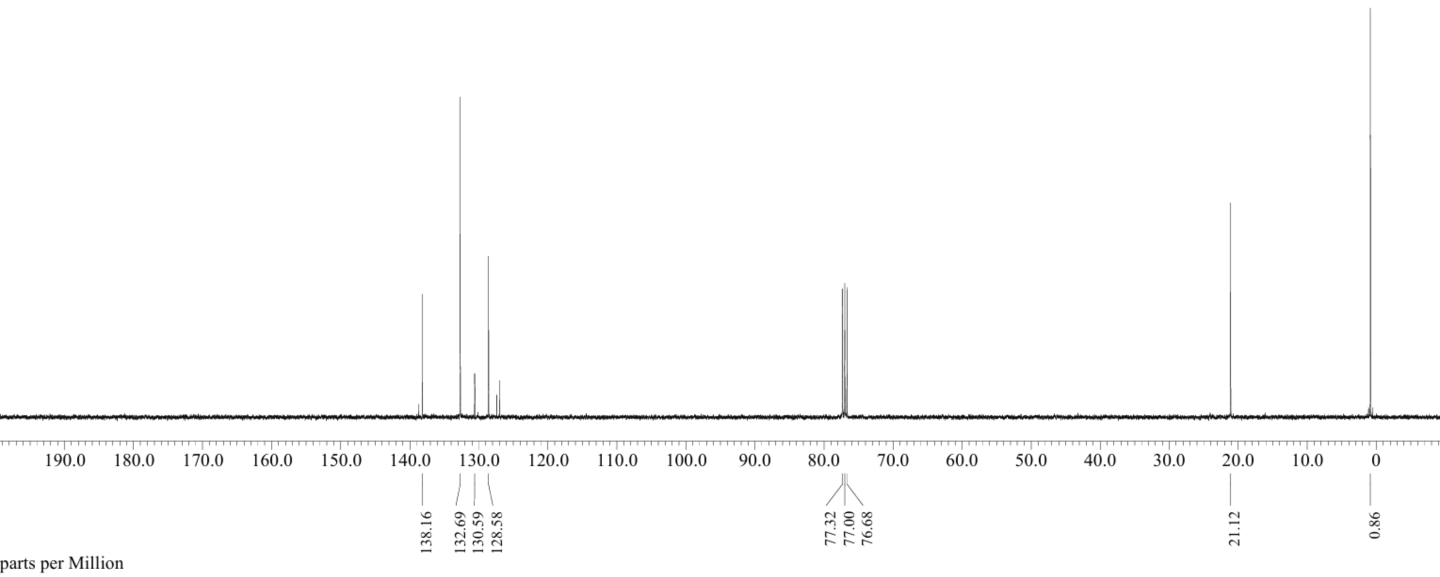

^{13}C{^{1}H} NMR (100 MHz, $CDCl_3$)

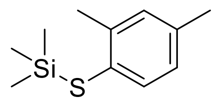

2j

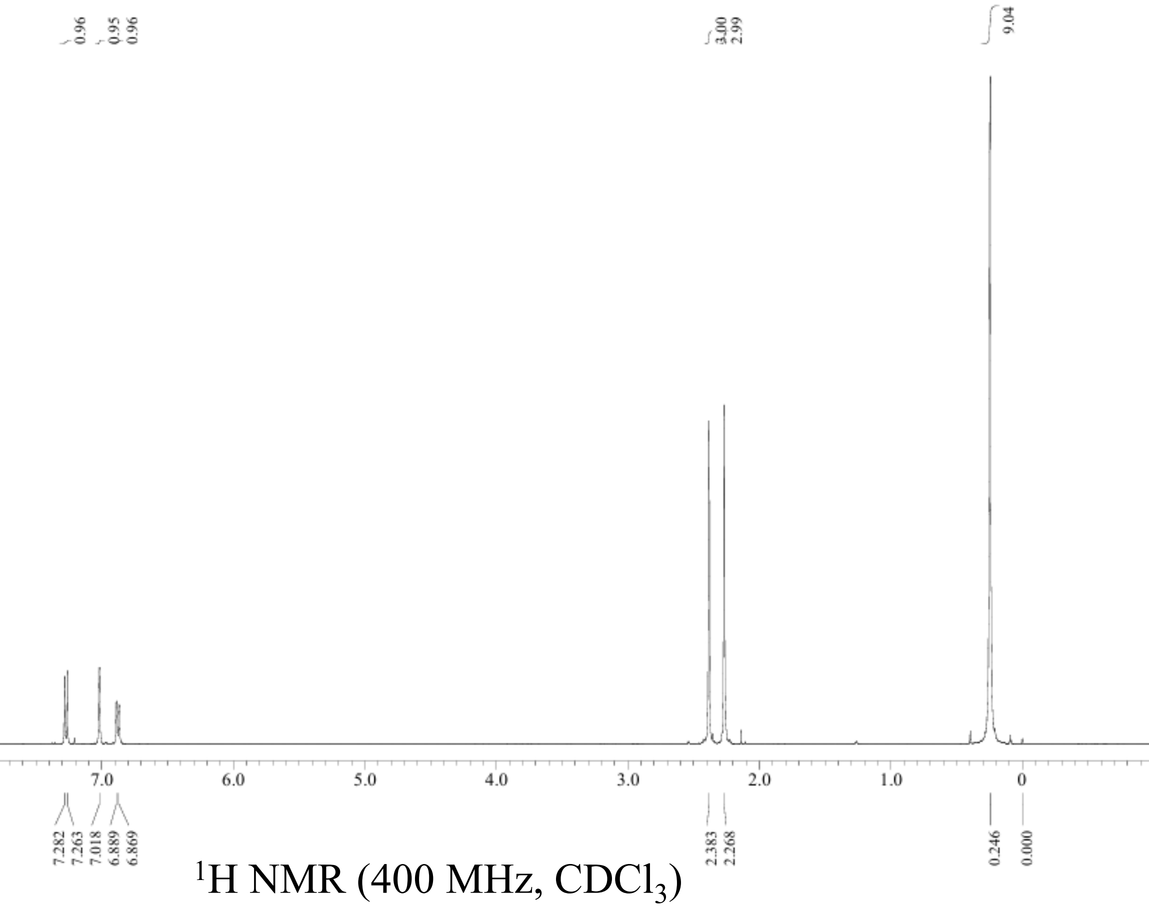

^{1}H NMR (400 MHz, $CDCl_3$)

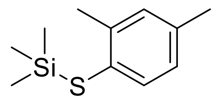

2j

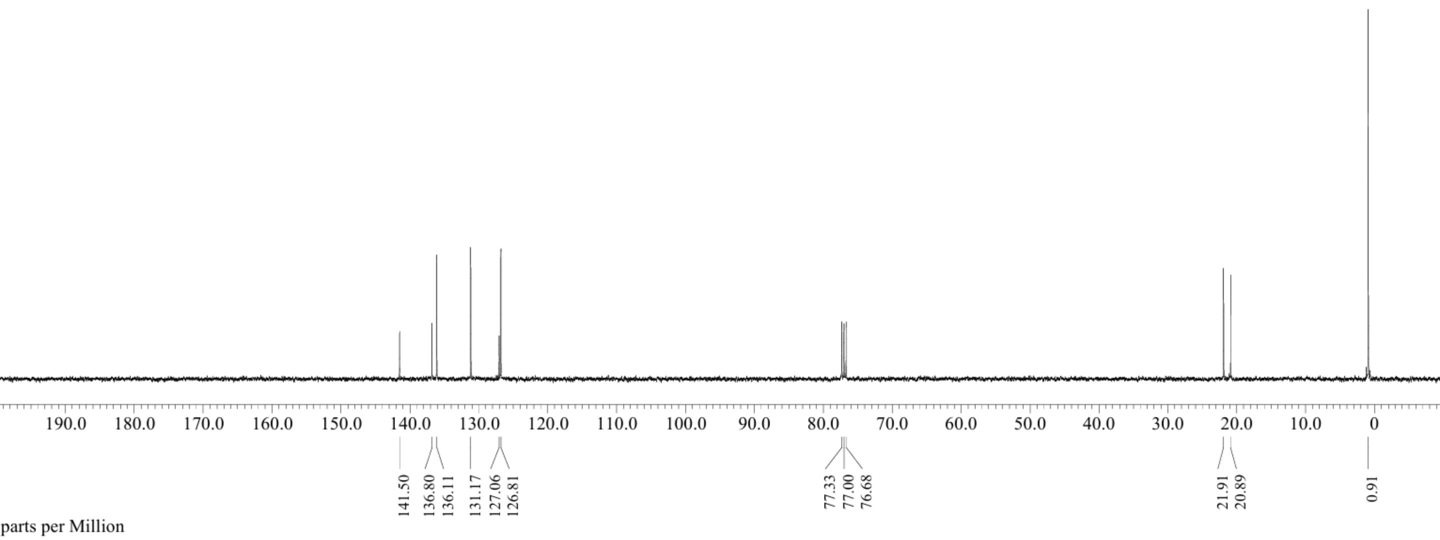

$^{13}C\{^{1}H\}$ NMR (100 MHz, $CDCl_3$)

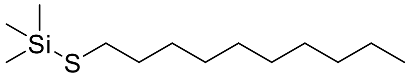

2k

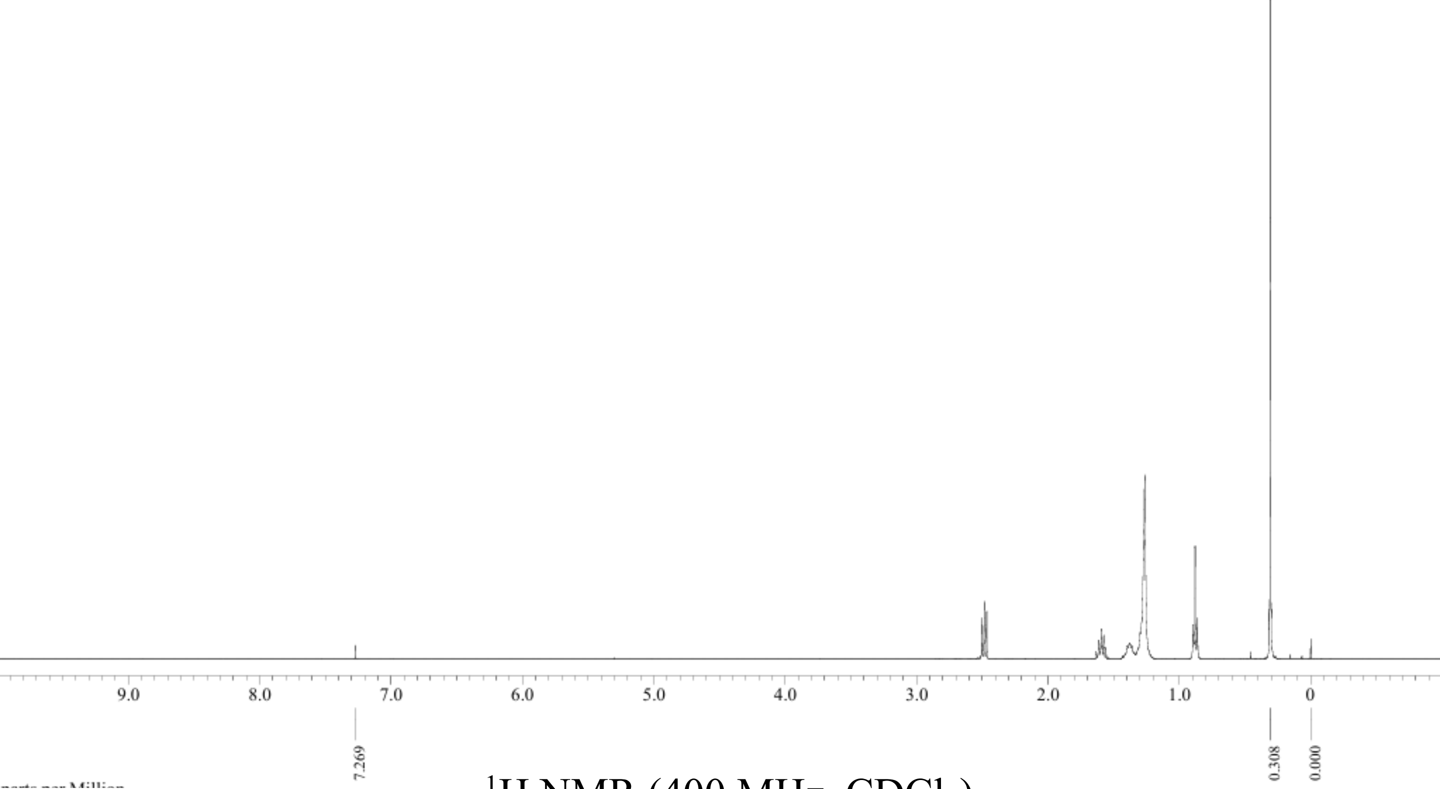

^{1}H NMR (400 MHz, $CDCl_3$)

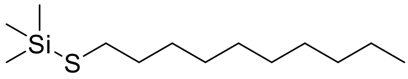

2k

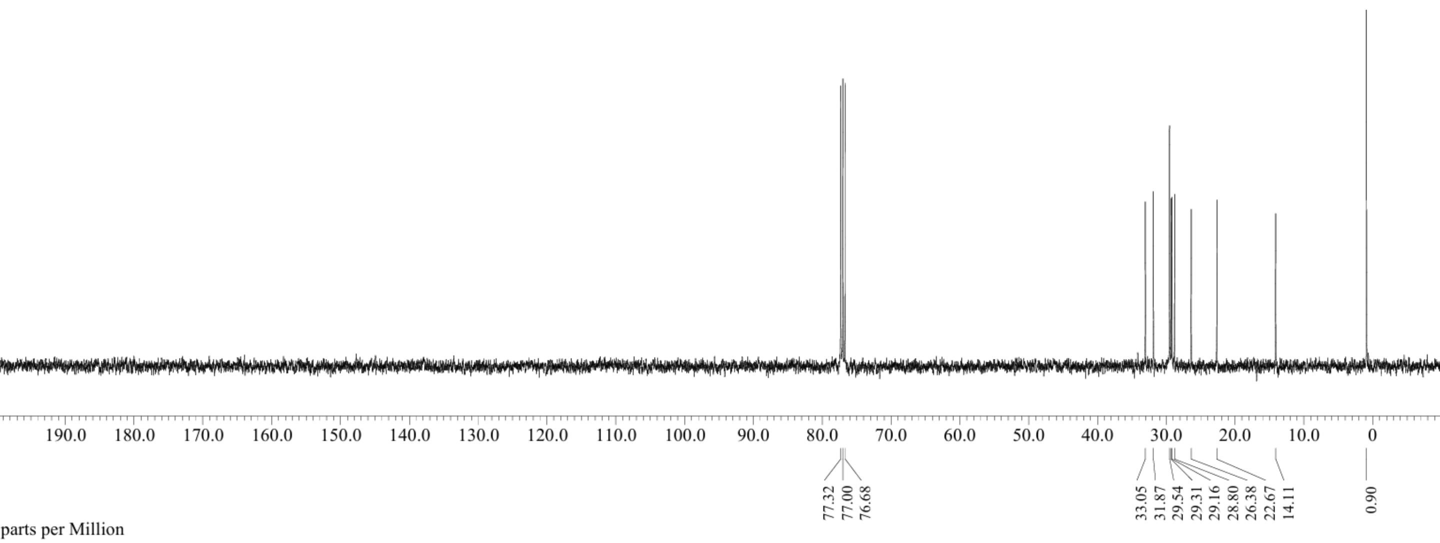

$^{13}C\{^{1}H\}$ NMR (100 MHz, $CDCl_3$)

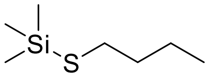

2l

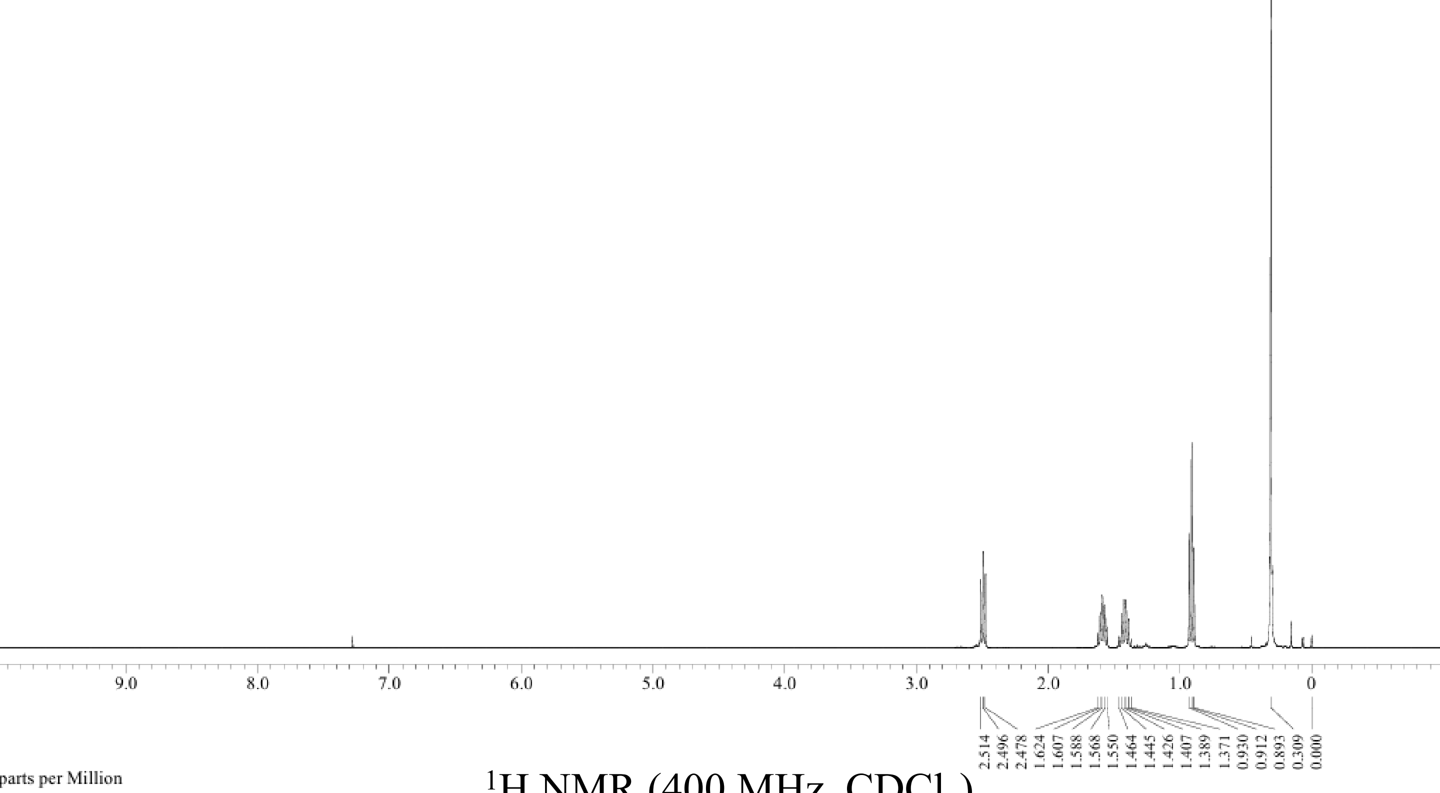

^{1}H NMR (400 MHz, $CDCl_3$)

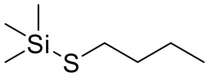

2l

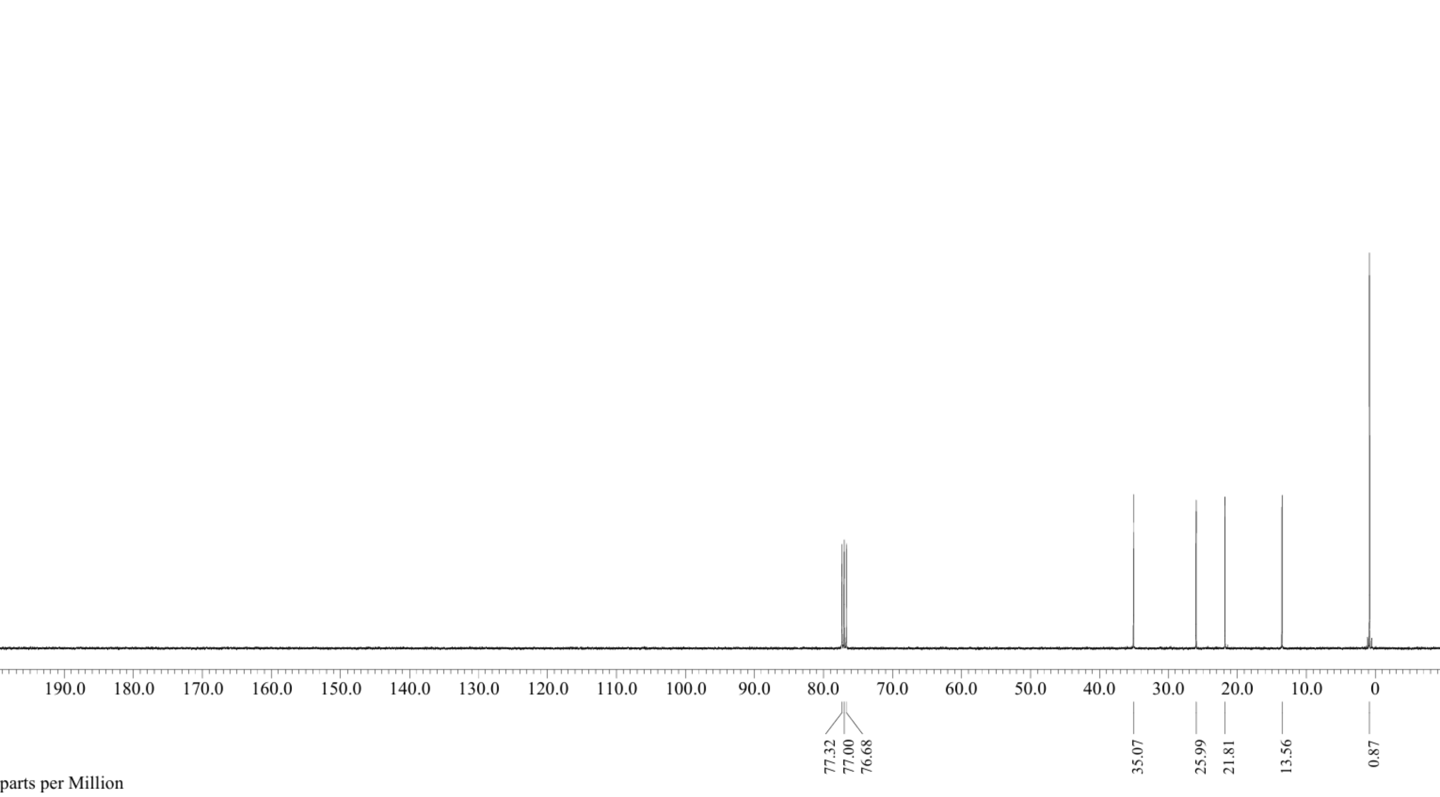

^{13}C{^{1}H} NMR (100 MHz, $CDCl_3$)

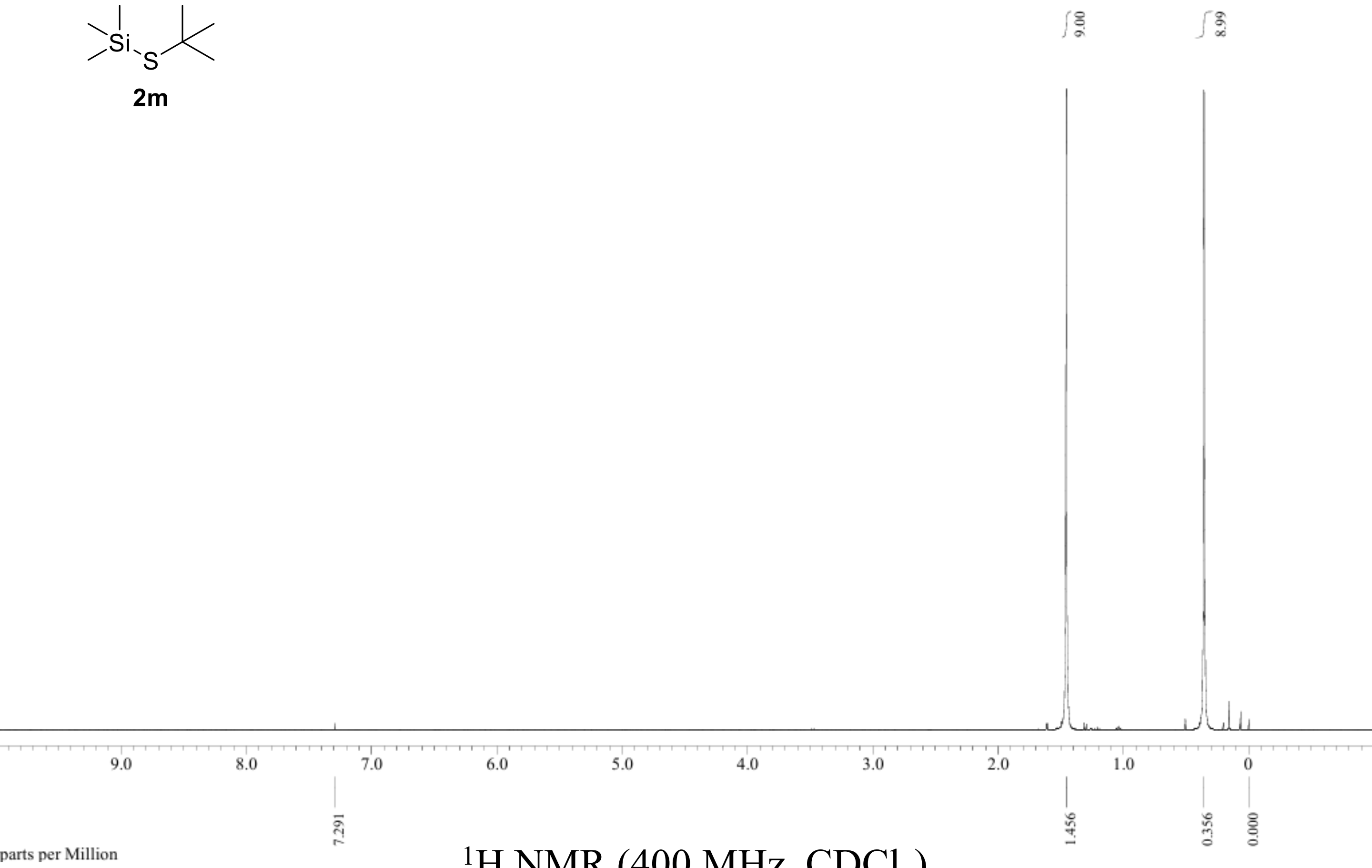

^{1}H NMR (400 MHz, $CDCl_3$)

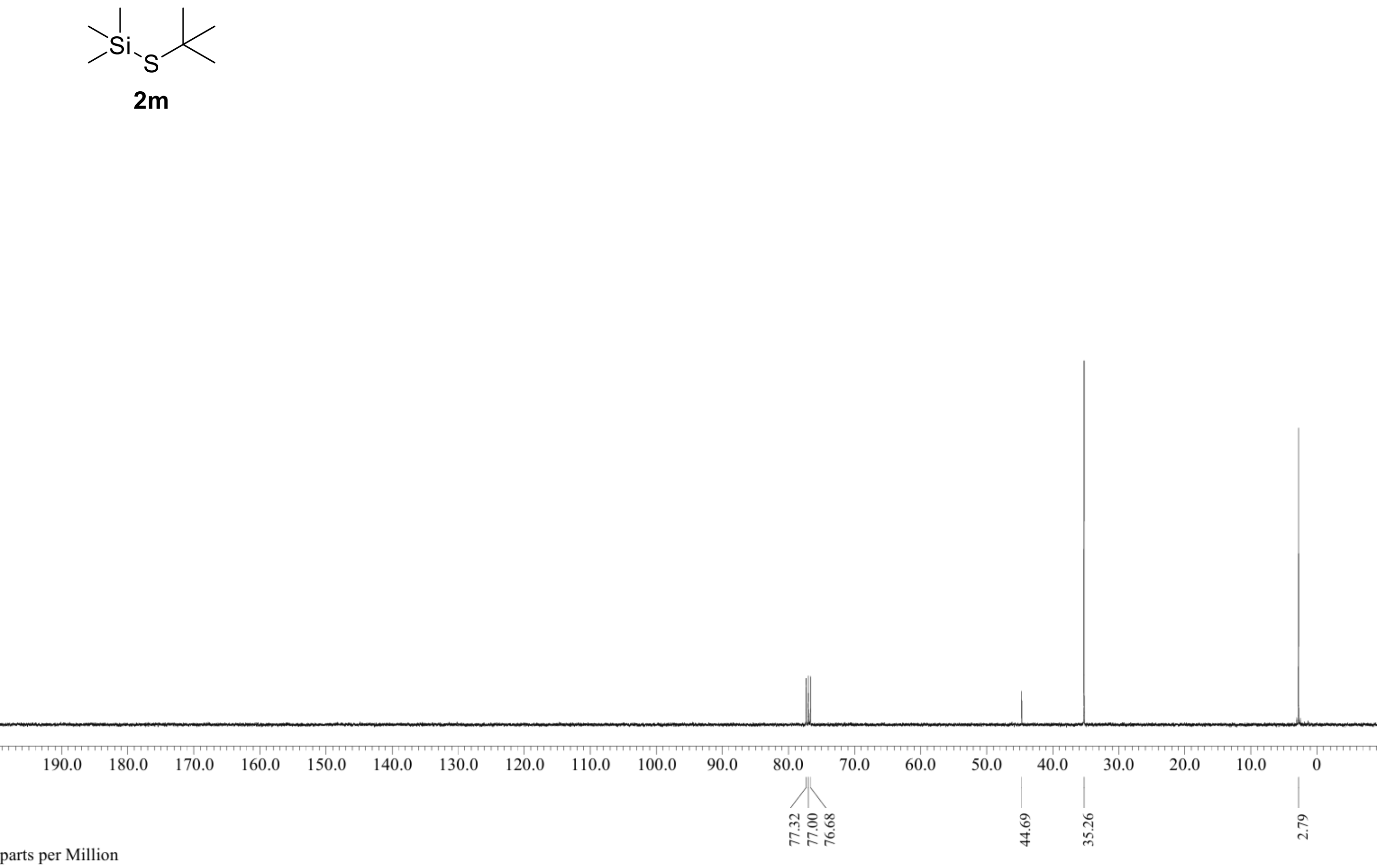

$^{13}C\{^{1}H\}$ NMR (100 MHz, $CDCl_3$)

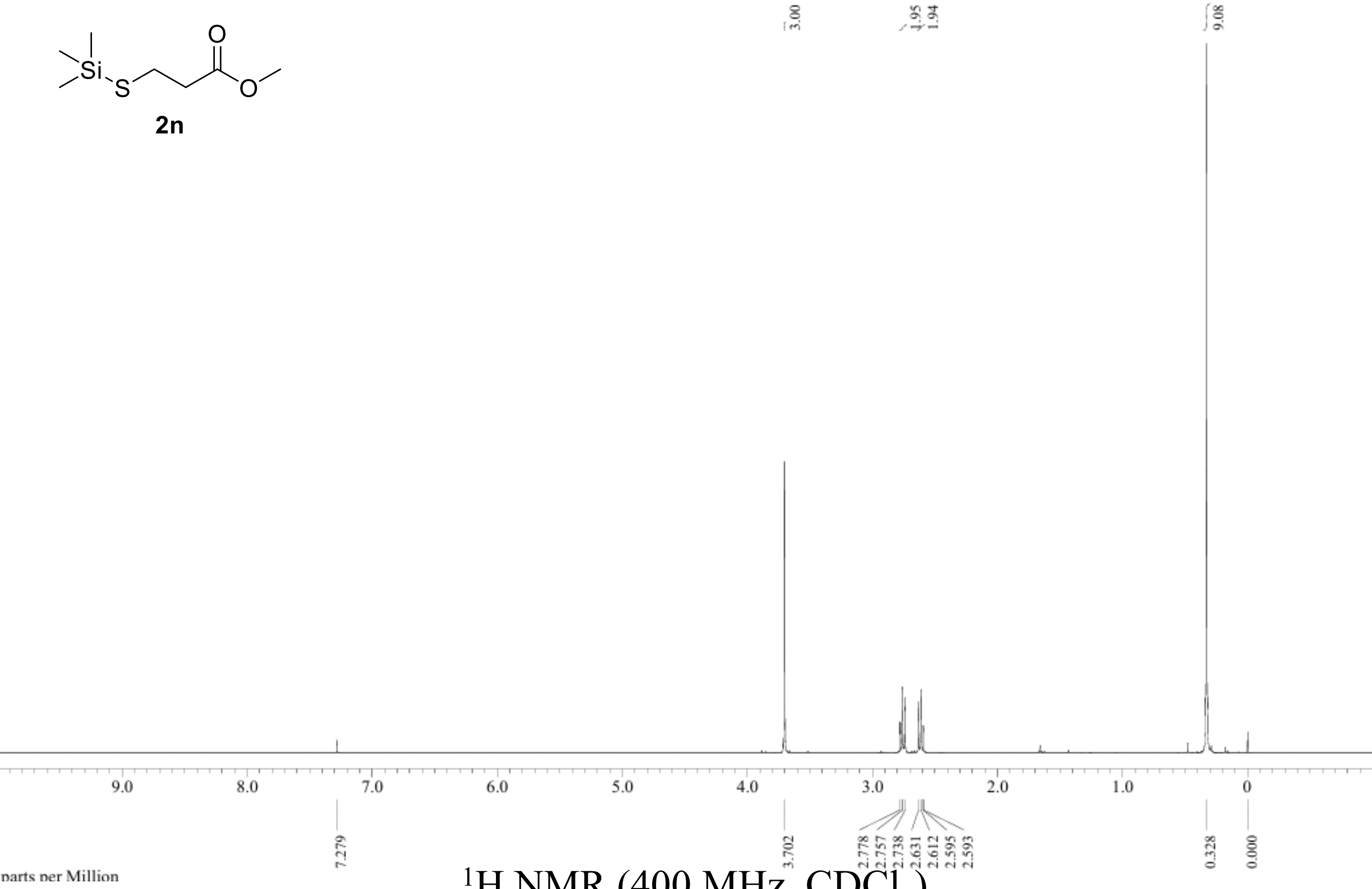

^{1}H NMR (400 MHz, $CDCl_3$)

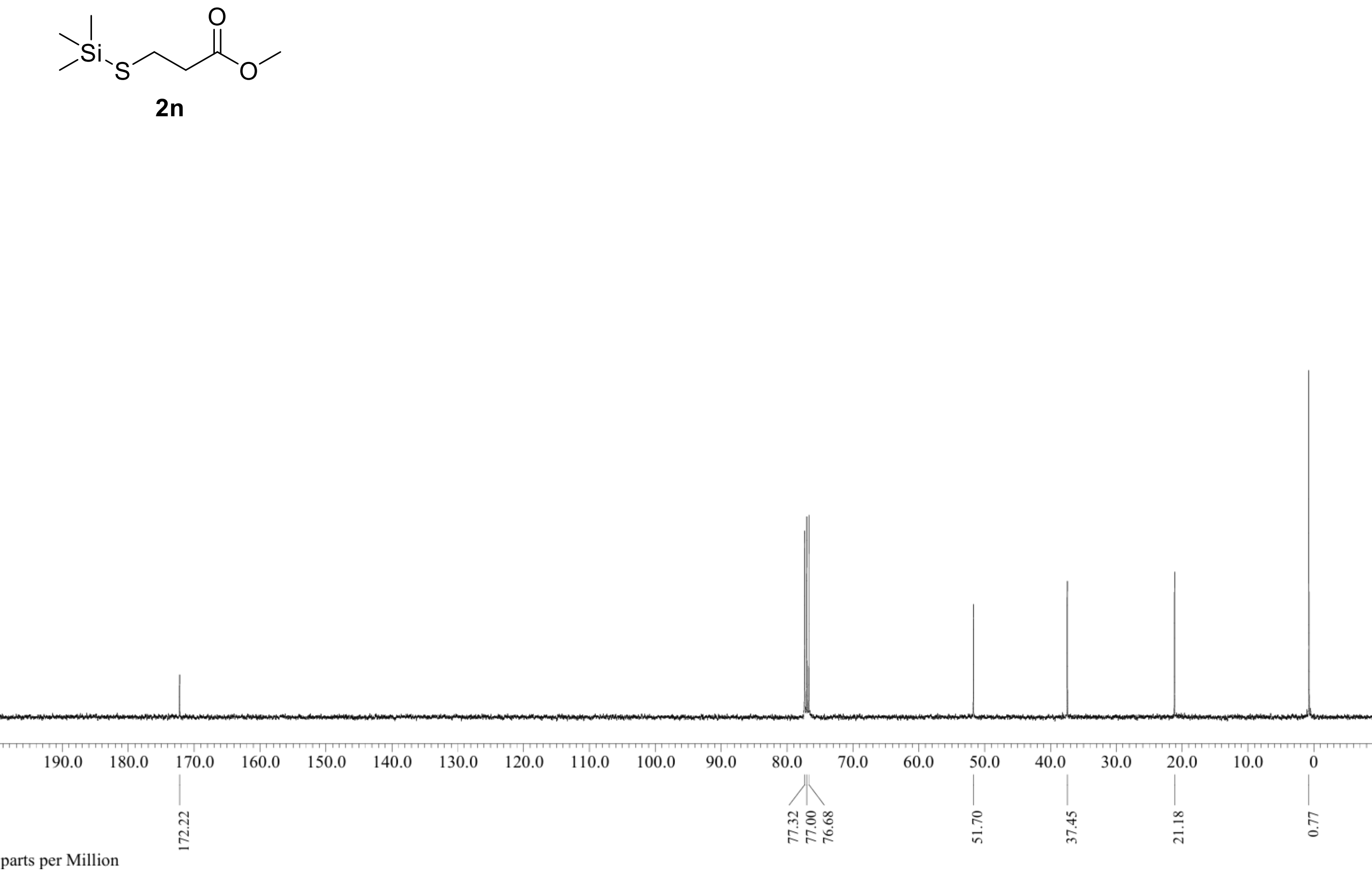

^{13}C{^{1}H} NMR (100 MHz, $CDCl_3$)

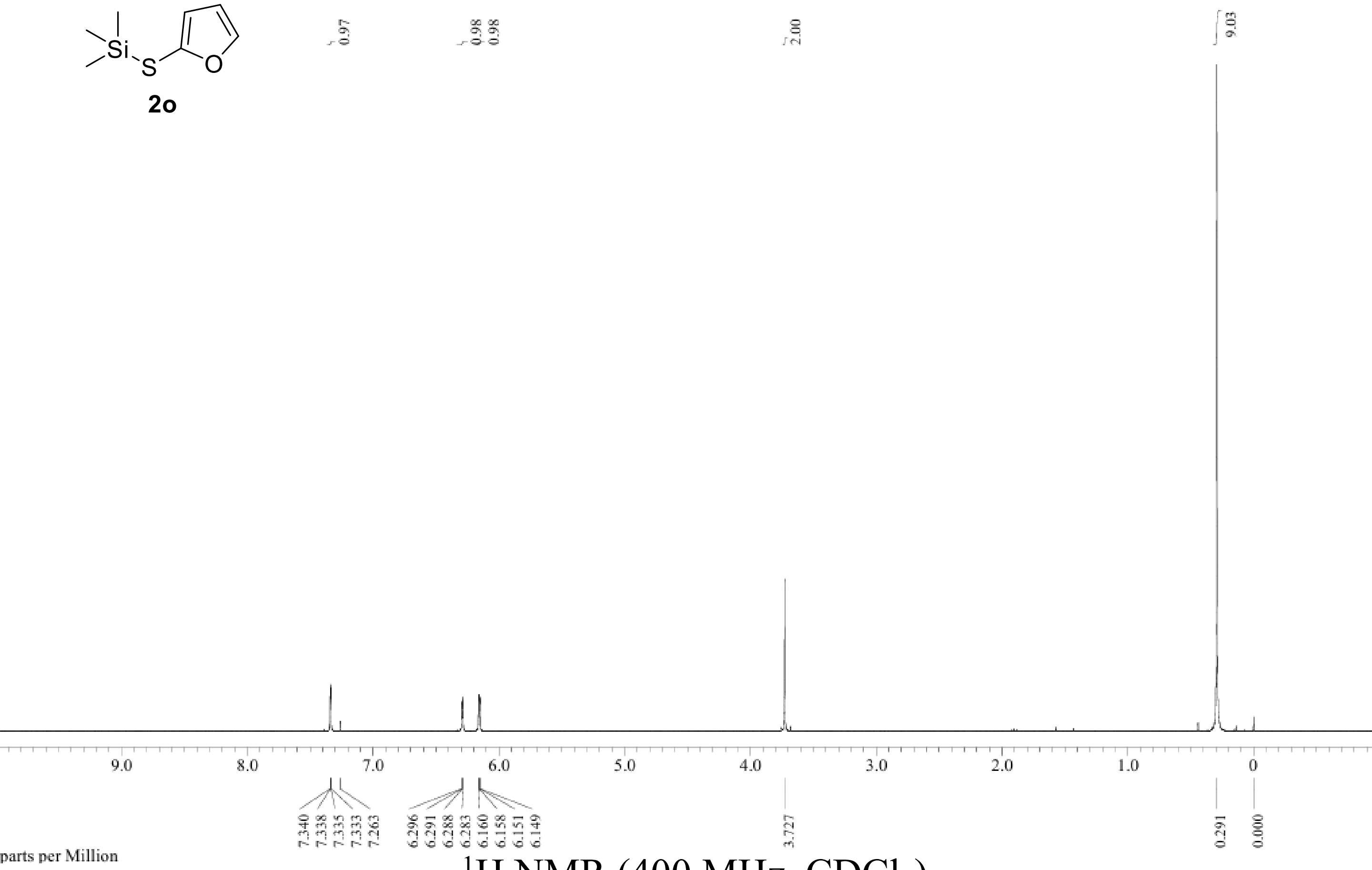

^{1}H NMR (400 MHz, $CDCl_3$)

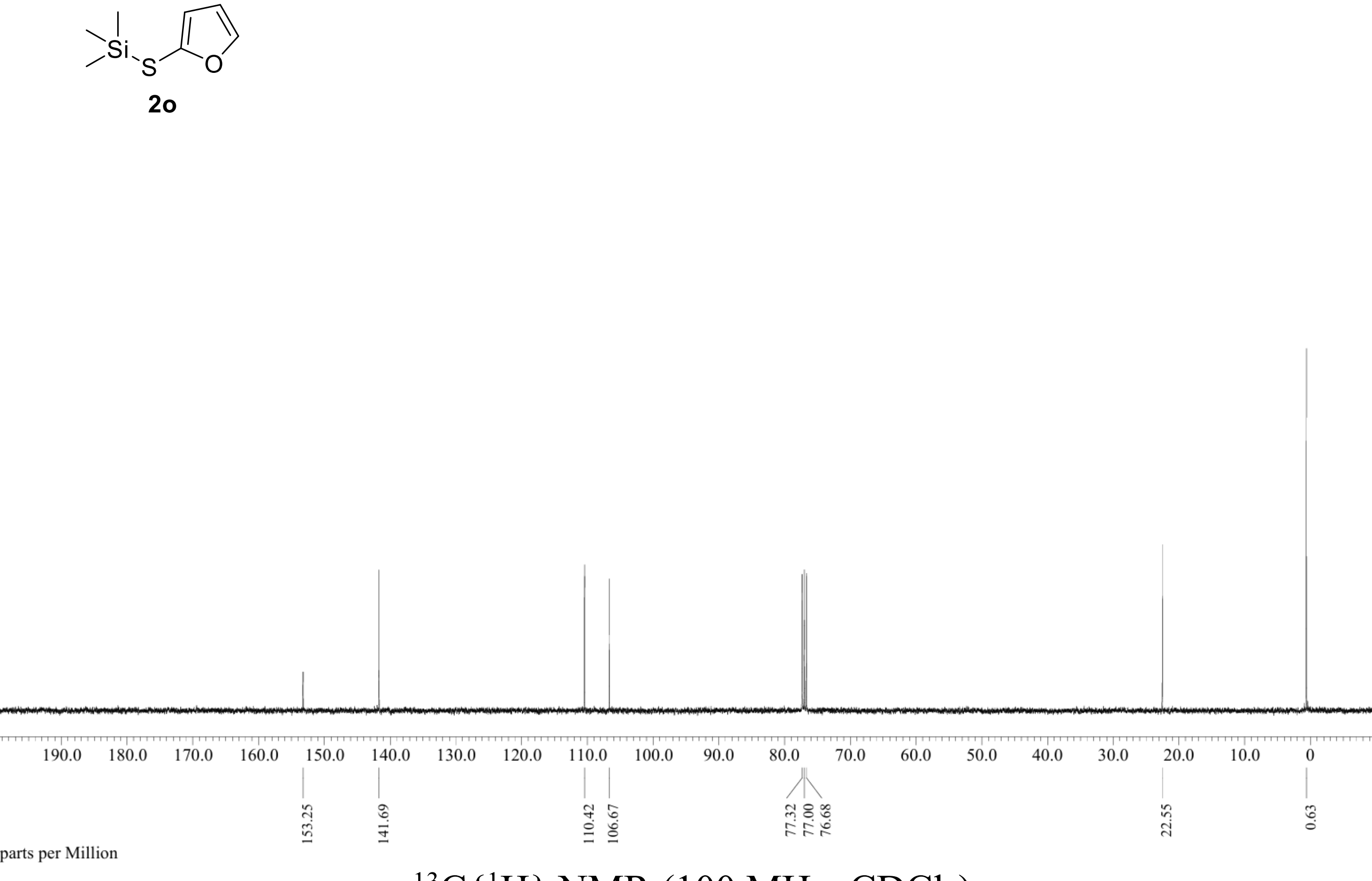

$^{13}C\{^{1}H\}$ NMR (100 MHz, $CDCl_3$)

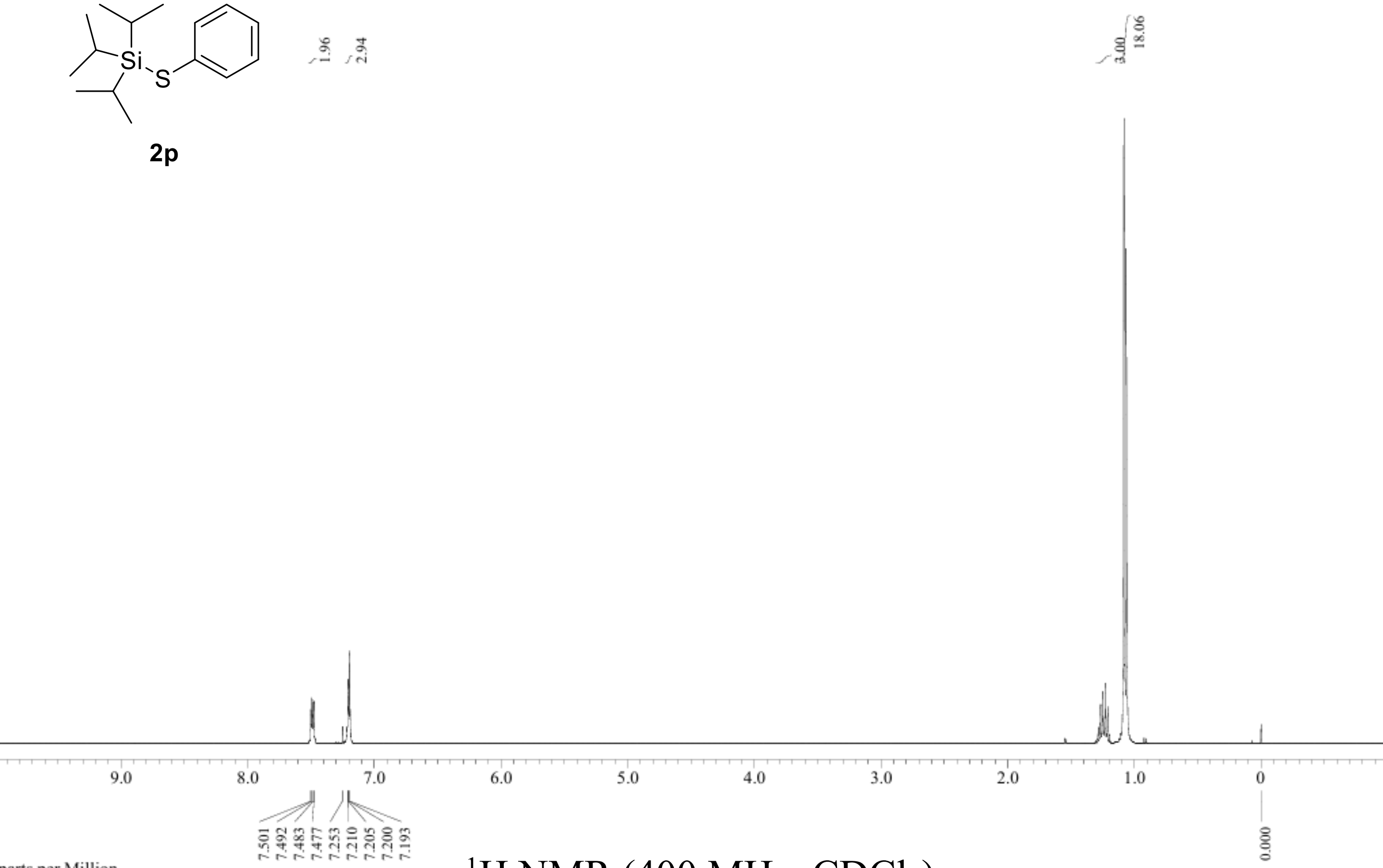

^{1}H NMR (400 MHz, $CDCl_3$)

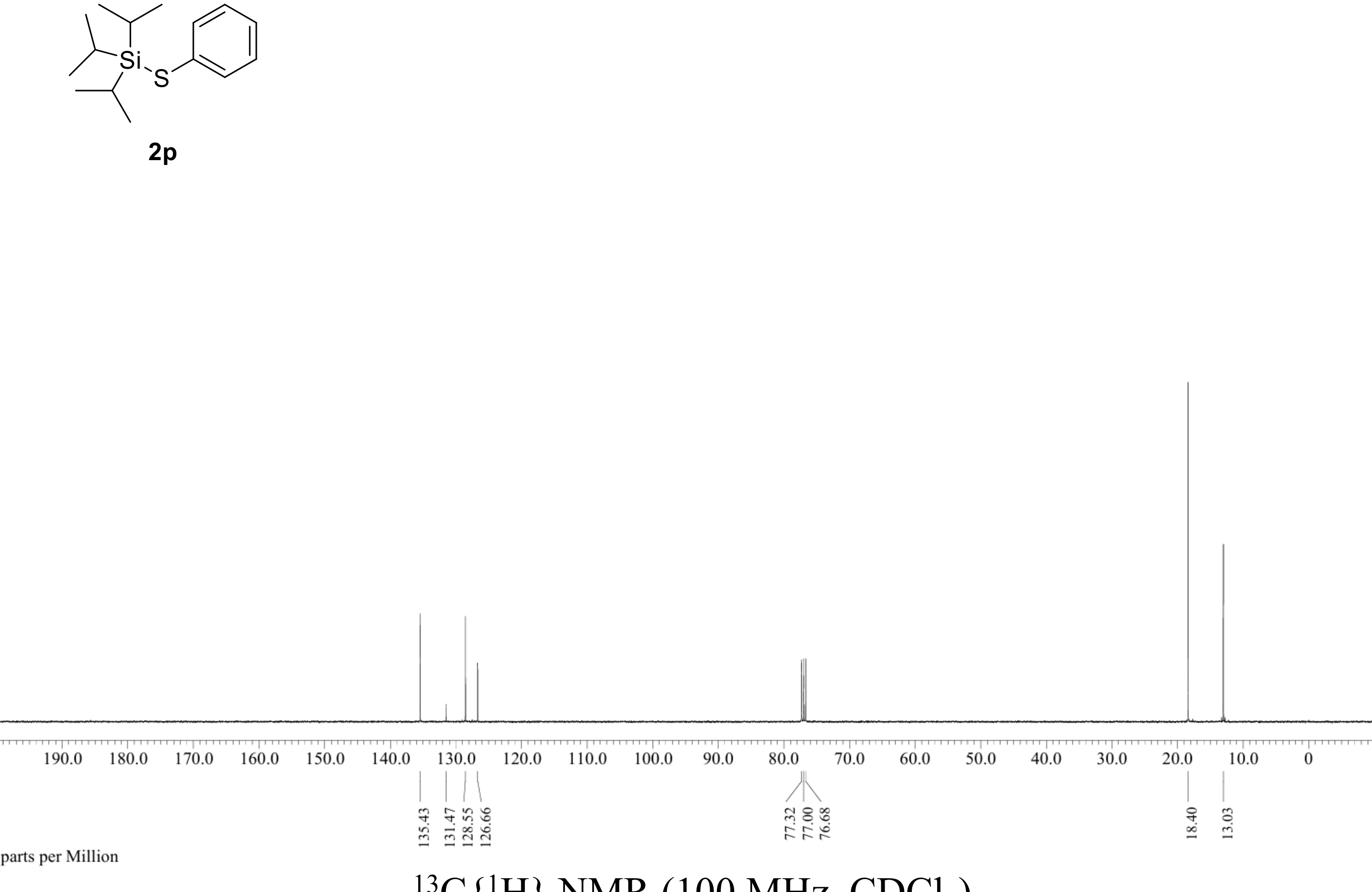

$^{13}C\{^{1}H\}$ NMR (100 MHz, $CDCl_3$)

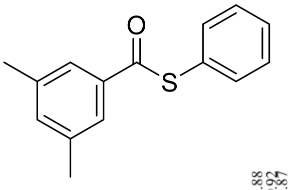

3aa

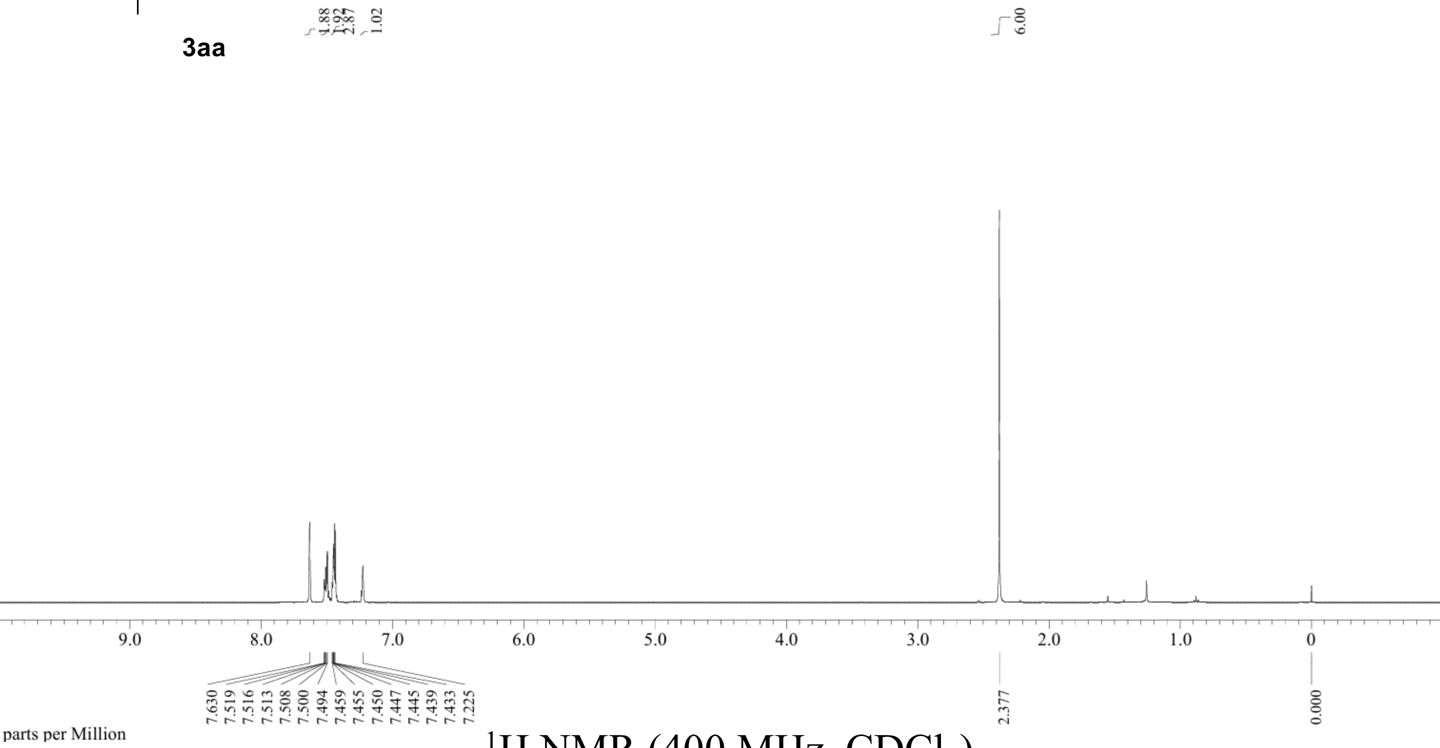

^{1}H NMR (400 MHz, $CDCl_3$)

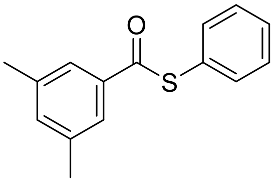

3aa

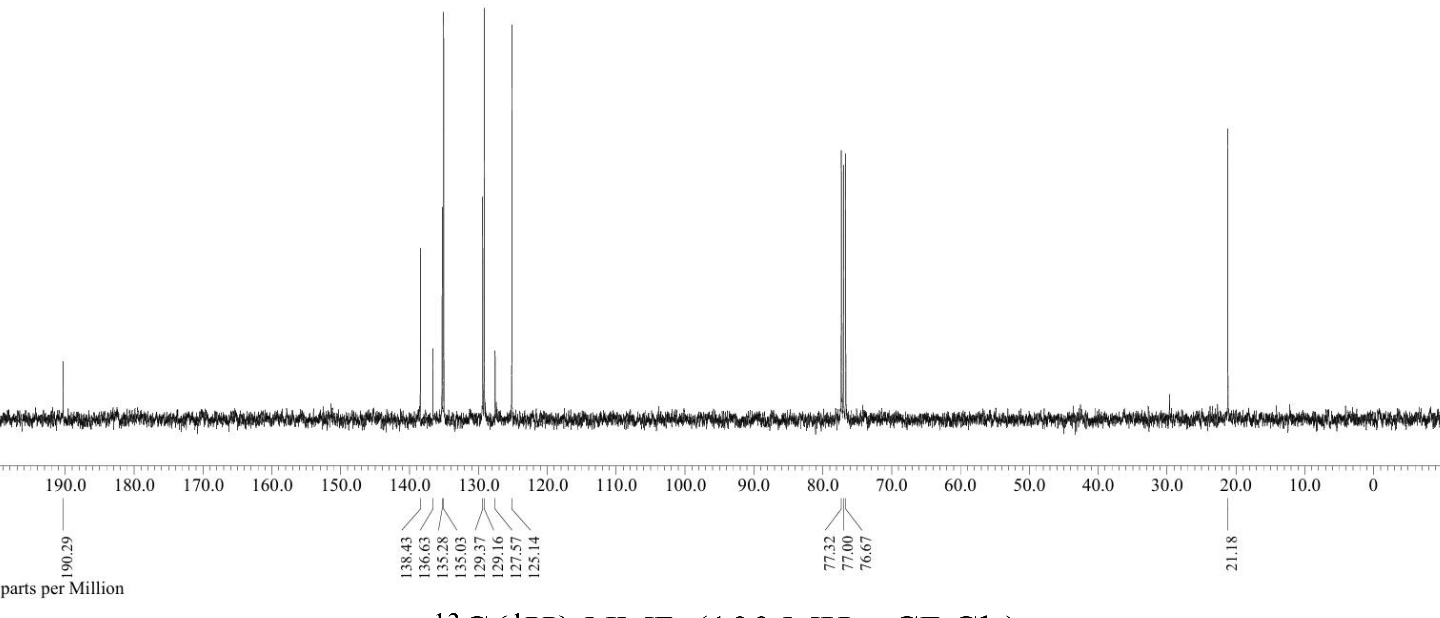

$^{13}C\{^{1}H\}$ NMR (100 MHz, $CDCl_3$)

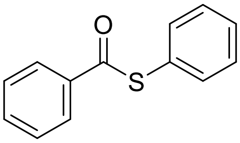

3ba

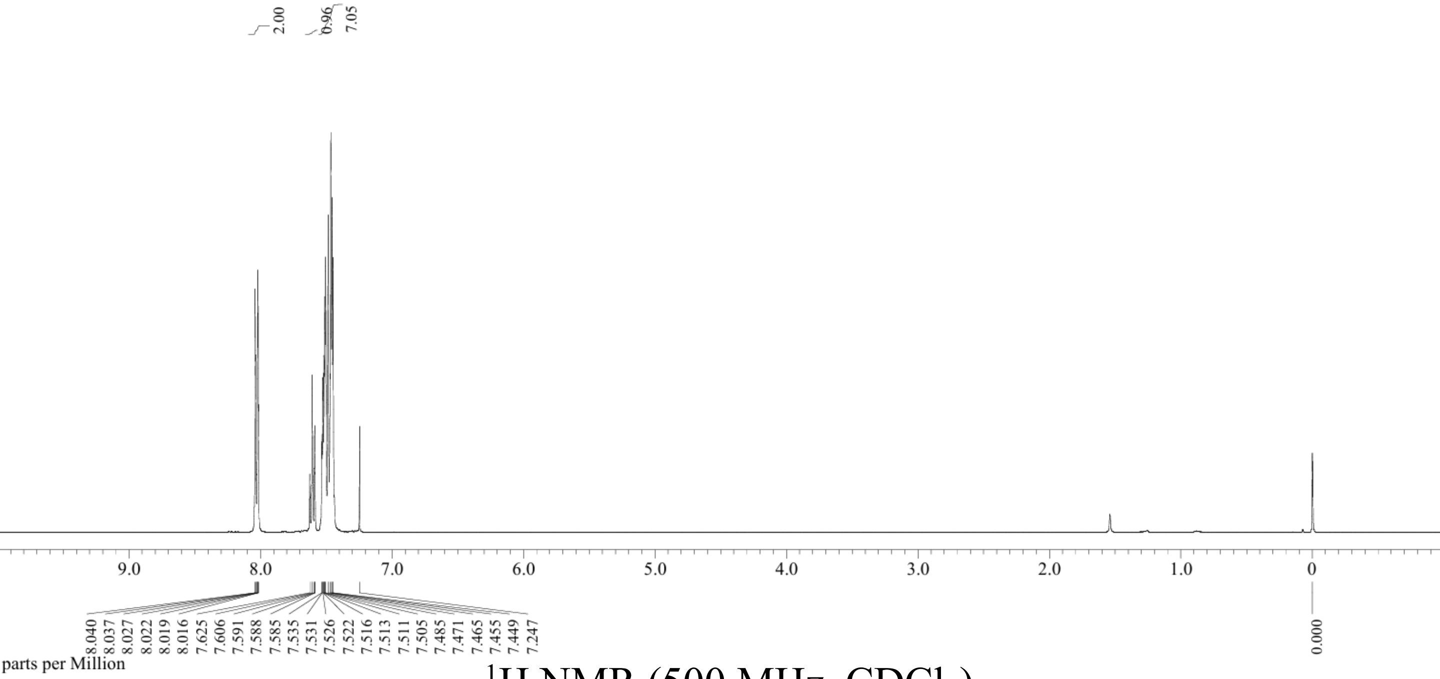

^{1}H NMR (500 MHz, $CDCl_3$)

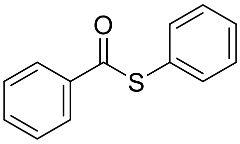

3ba

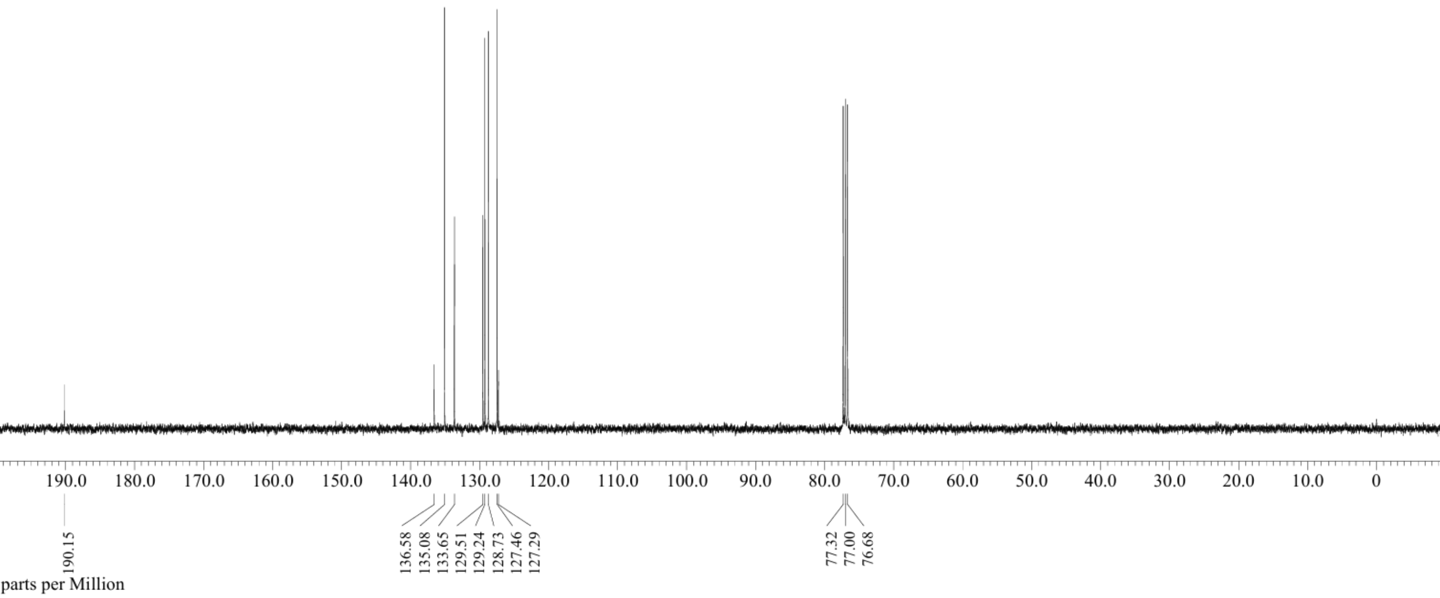

$^{13}C\{^{1}H\}$ NMR (126MHz, $CDCl_3$)

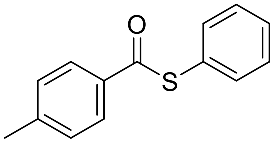

3ca

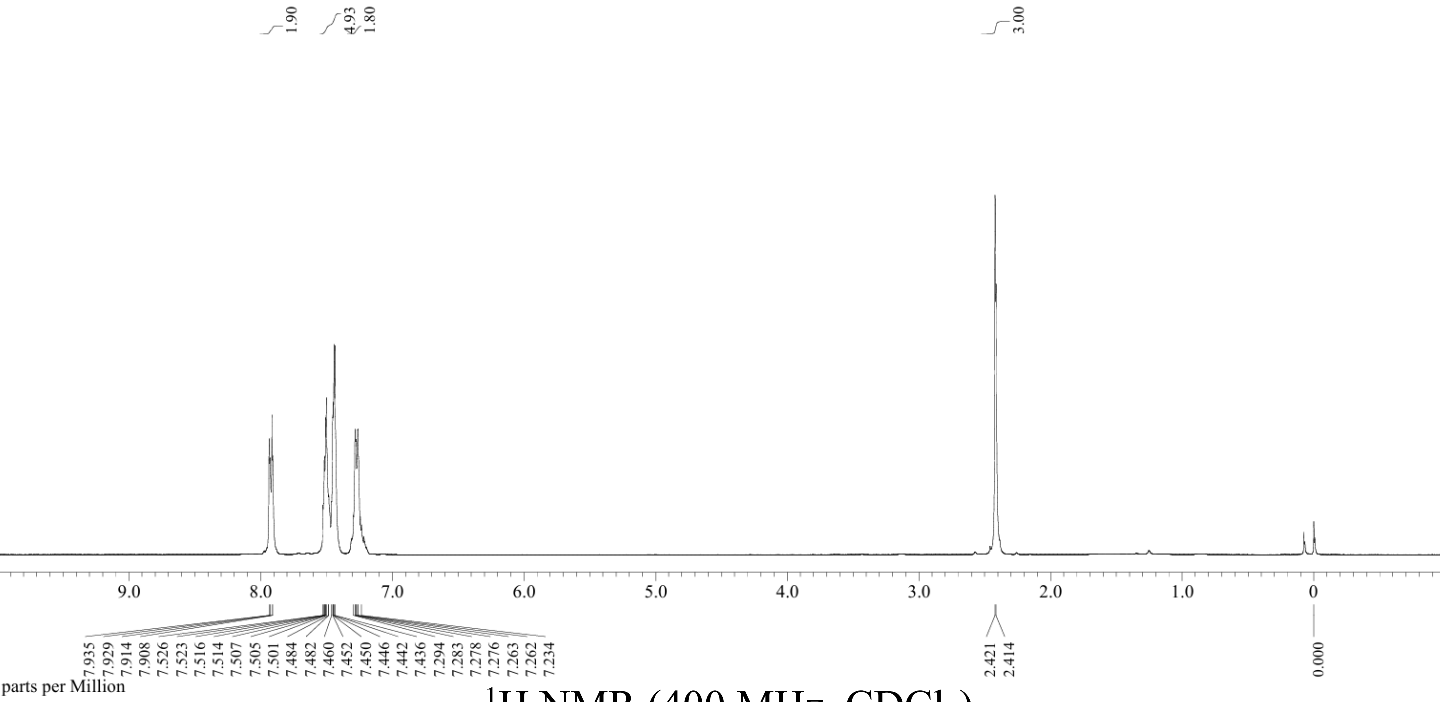

^{1}H NMR (400 MHz, $CDCl_3$)

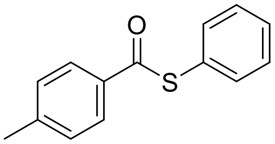

3ca

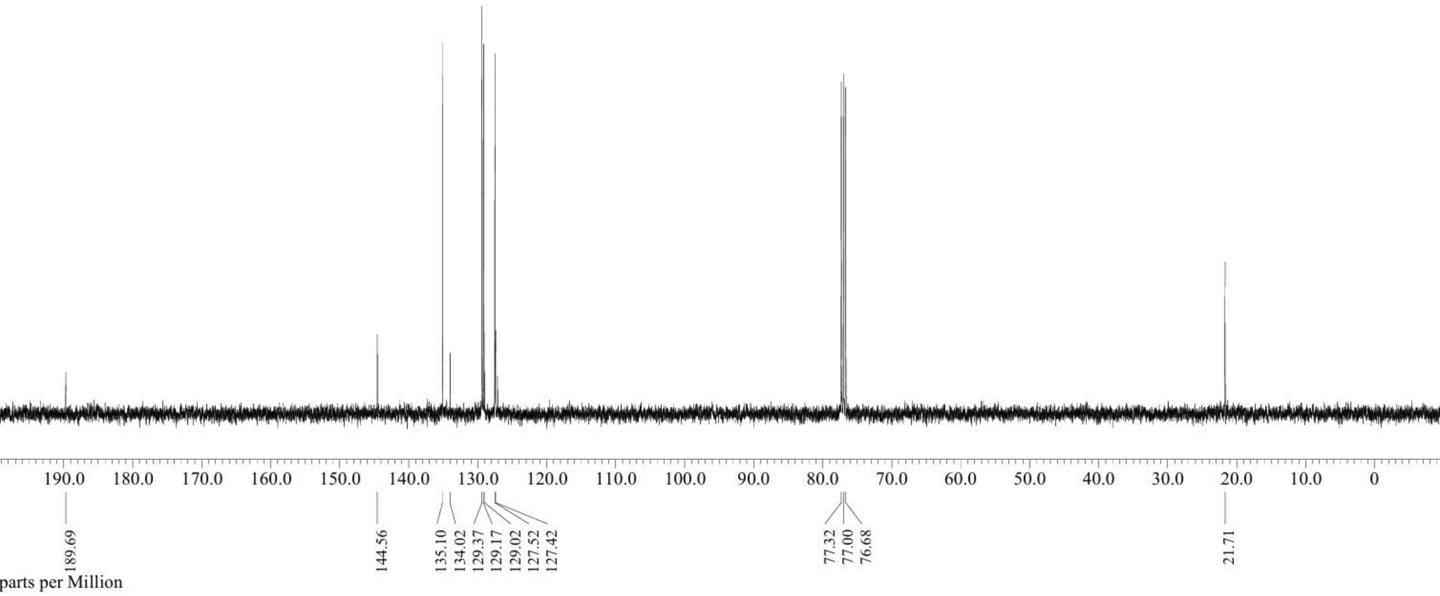

$^{13}C\{^{1}H\}$ NMR (100 MHz, $CDCl_3$)

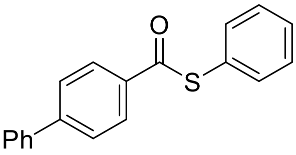

3da

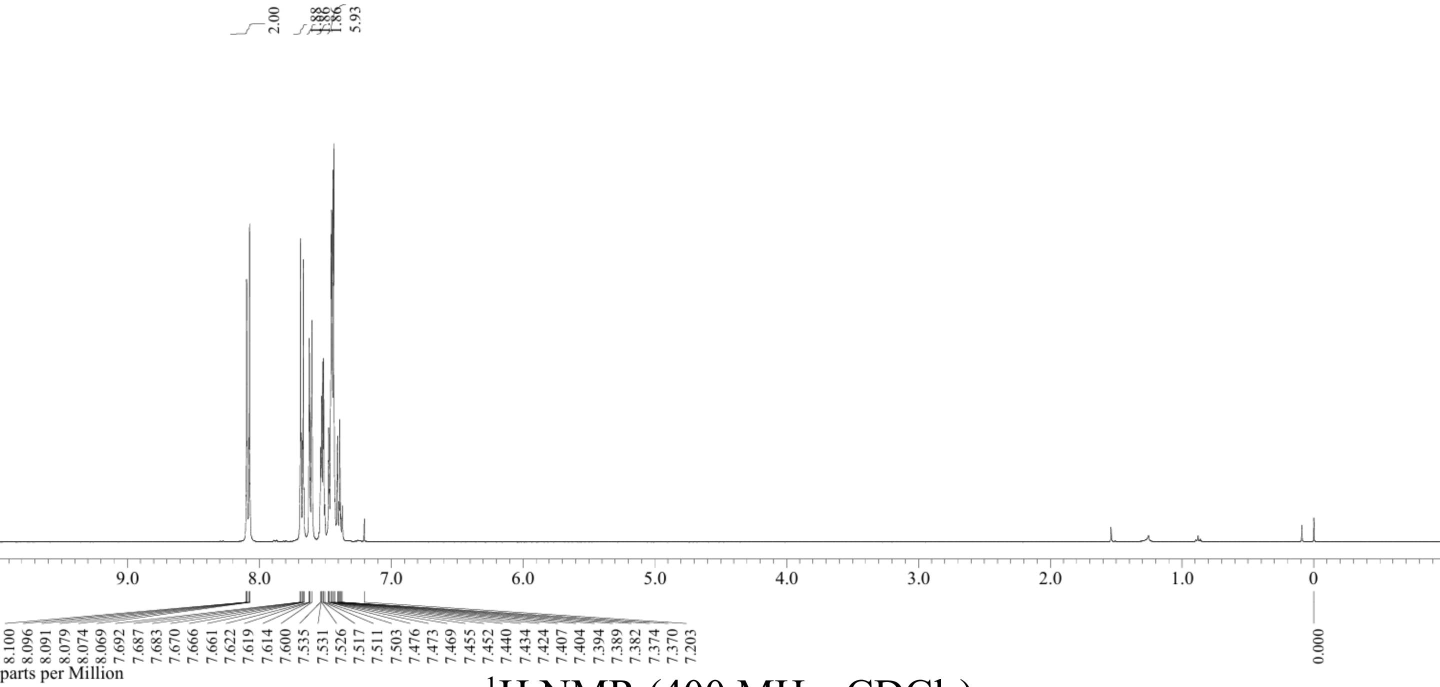

^{1}H NMR (400 MHz, $CDCl_3$)

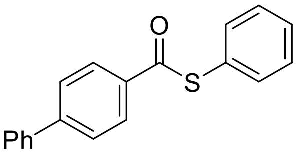

3da

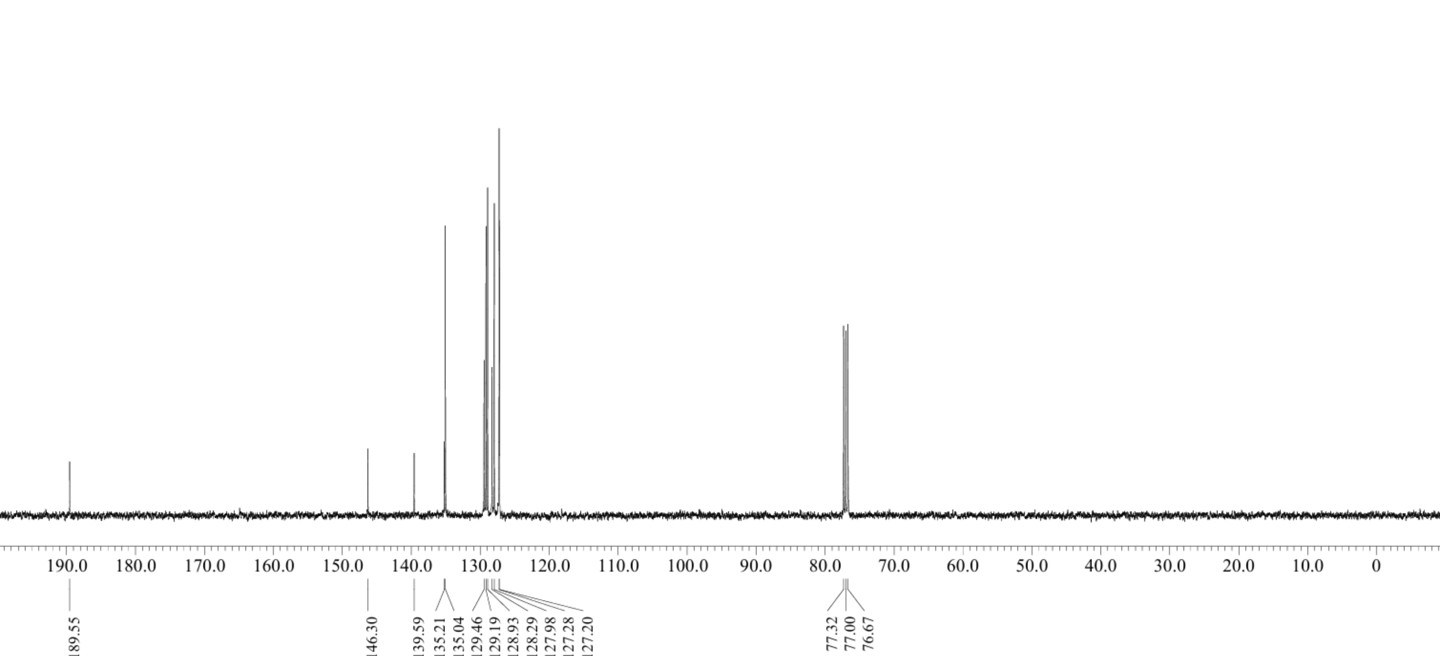

$^{13}C\{^1H\}$ NMR (100 MHz, $CDCl_3$)

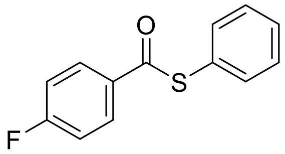

3ea

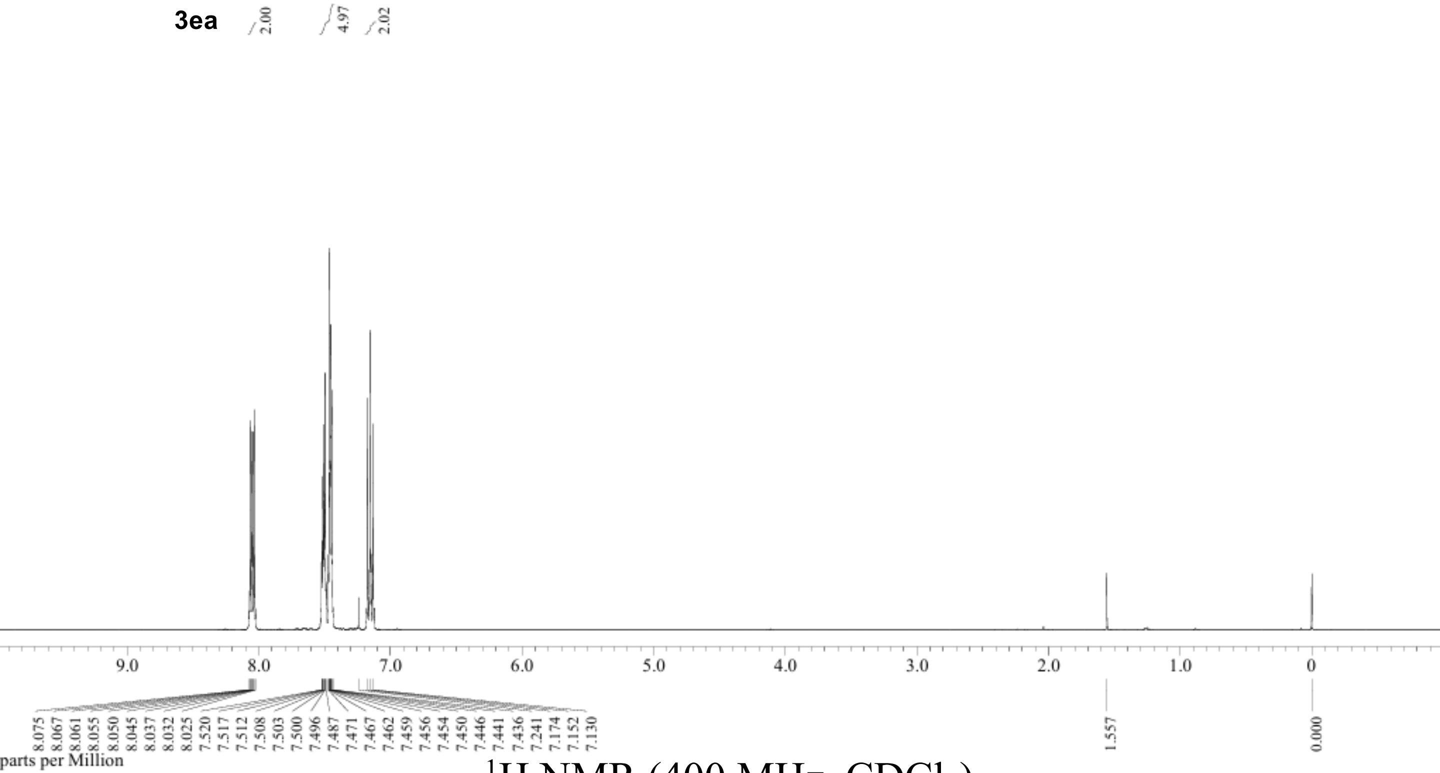

^{1}H NMR (400 MHz, $CDCl_3$)

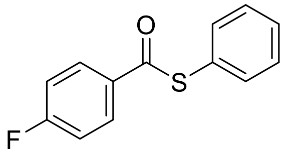

3ea

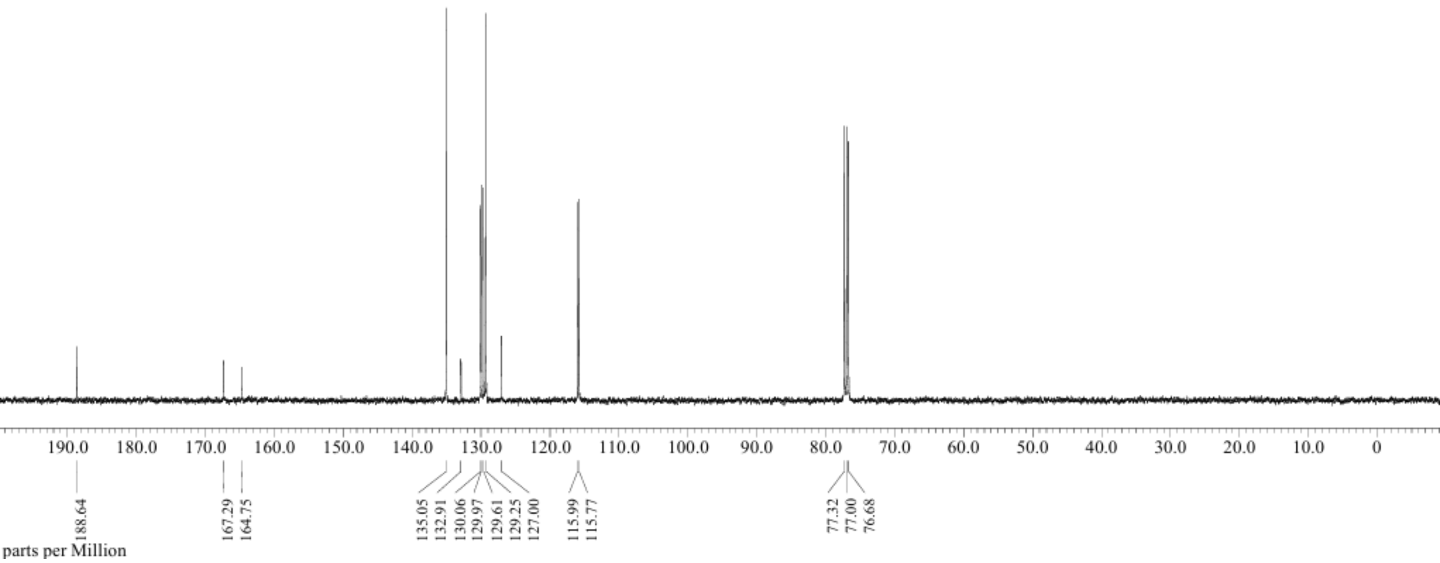

$^{13}C\{^{1}H\}$ NMR (100 MHz, $CDCl_3$)

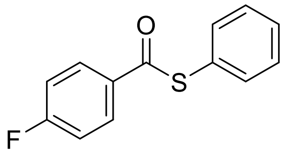

3ea

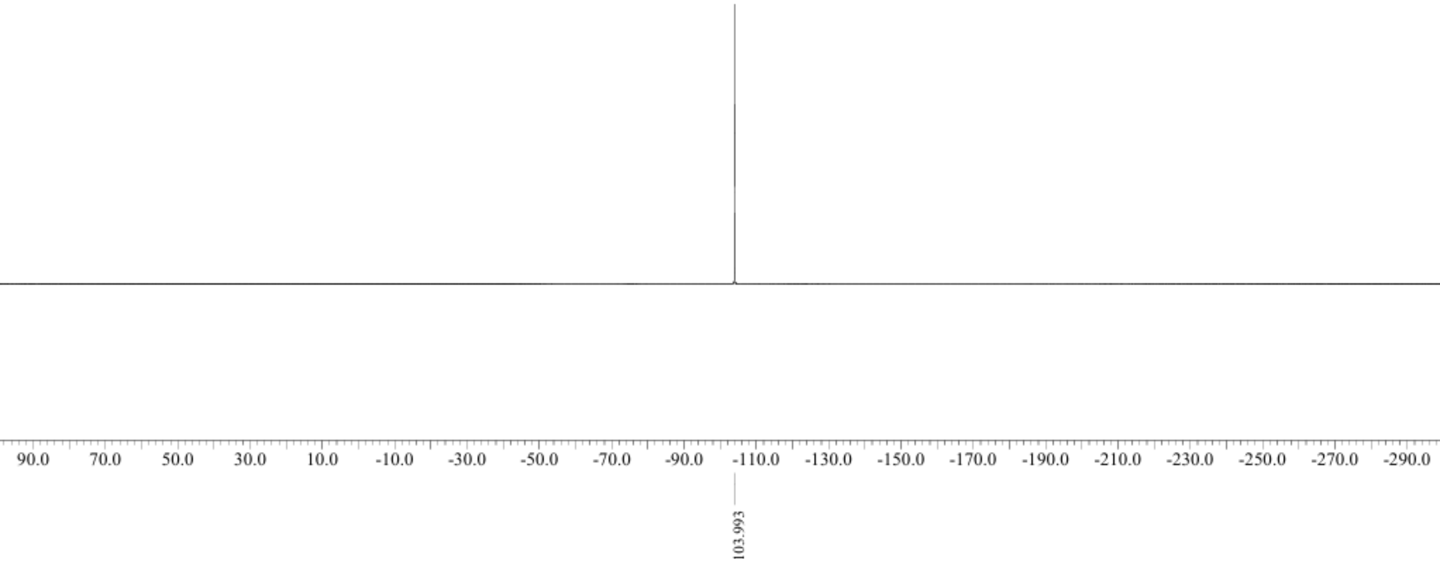

^{19}F NMR (376 MHz, $CDCl_3$)

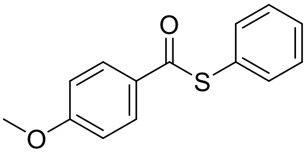

3fa

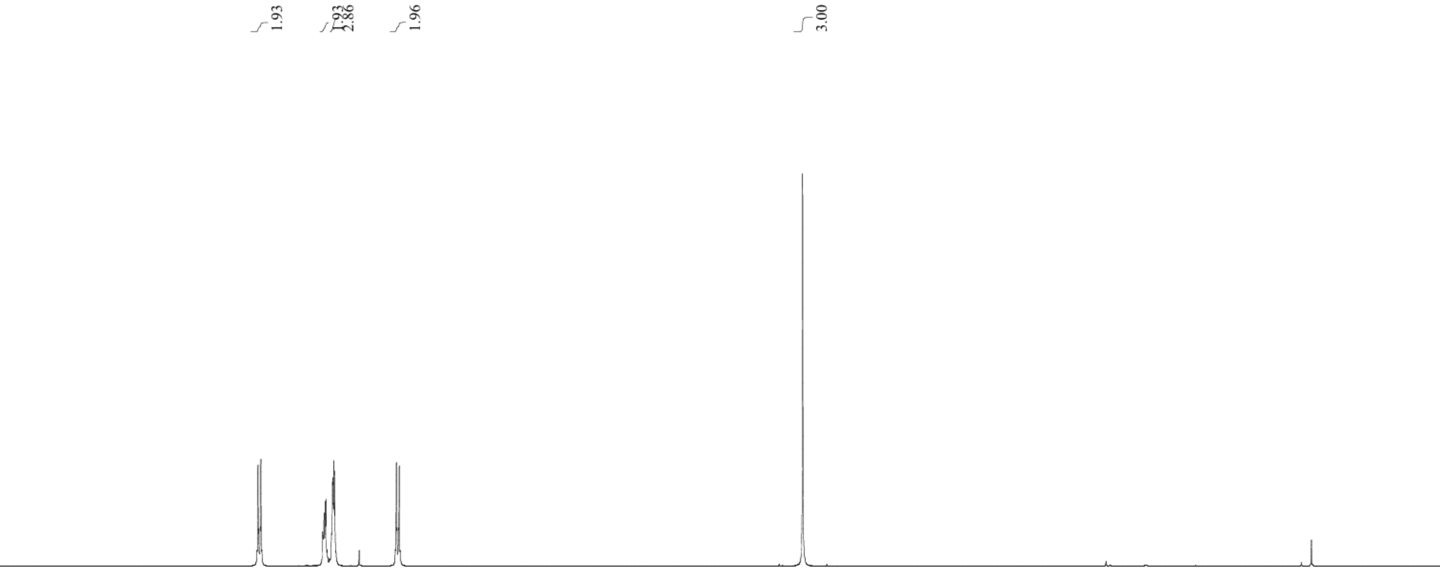

^{1}H NMR (400 MHz, $CDCl_3$)

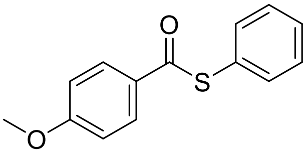

3fa

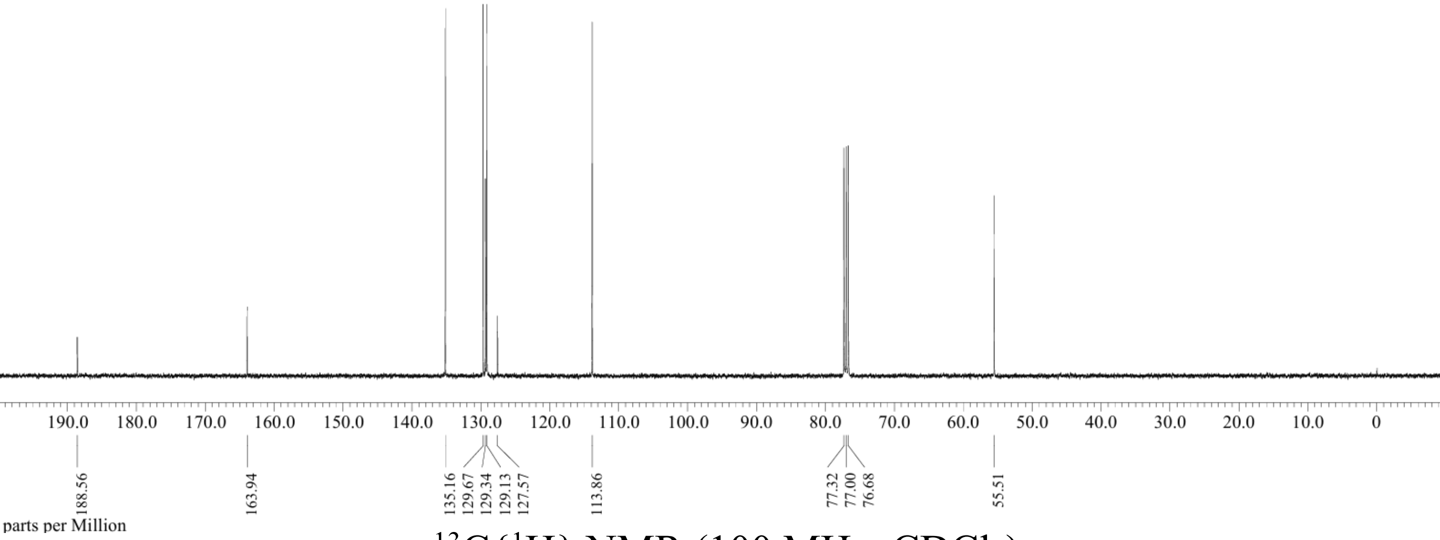

$^{13}C\{^{1}H\}$ NMR (100 MHz, $CDCl_3$)

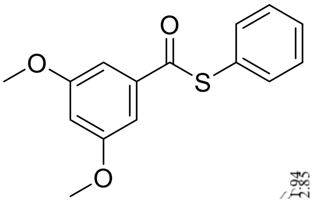

3ga

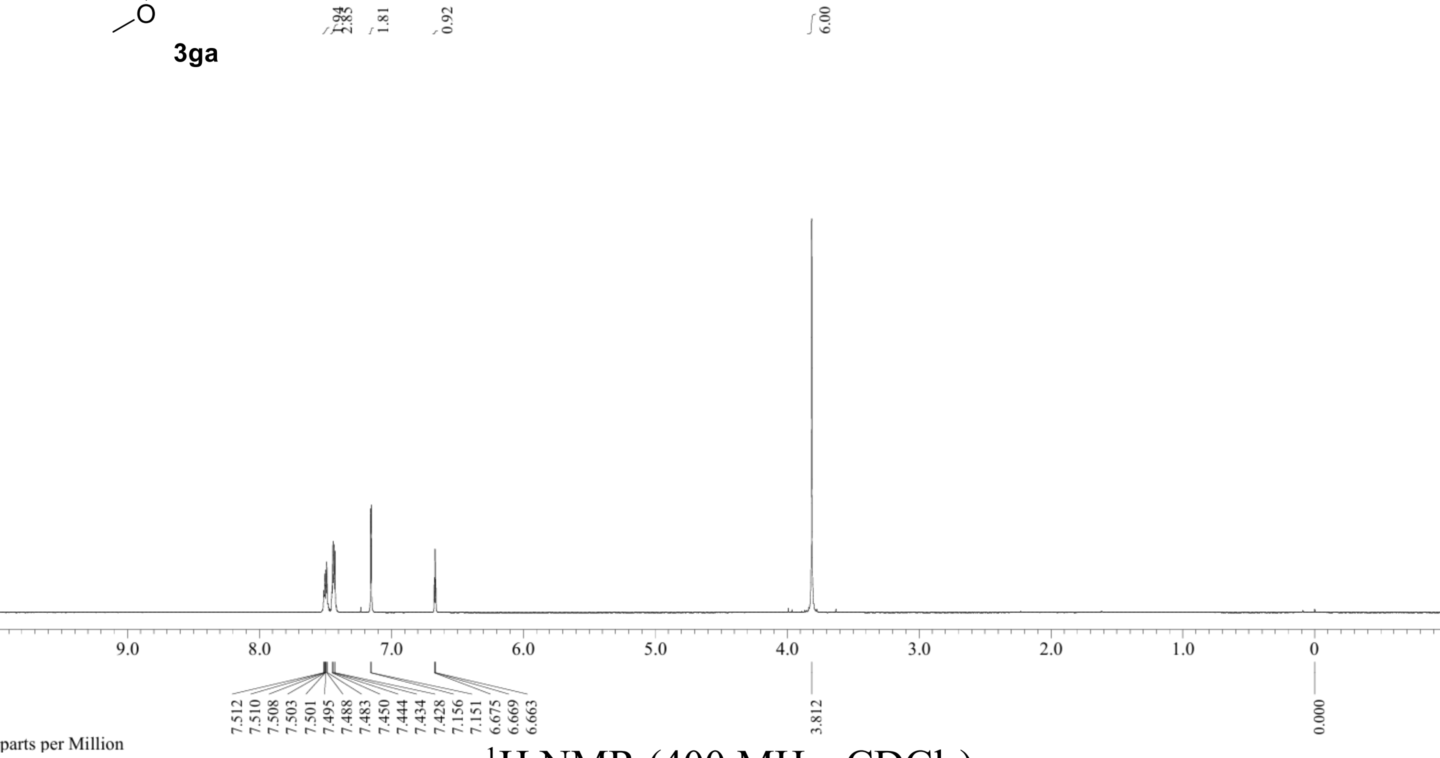

^{1}H NMR (400 MHz, $CDCl_3$)

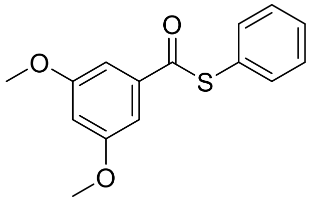

3ga

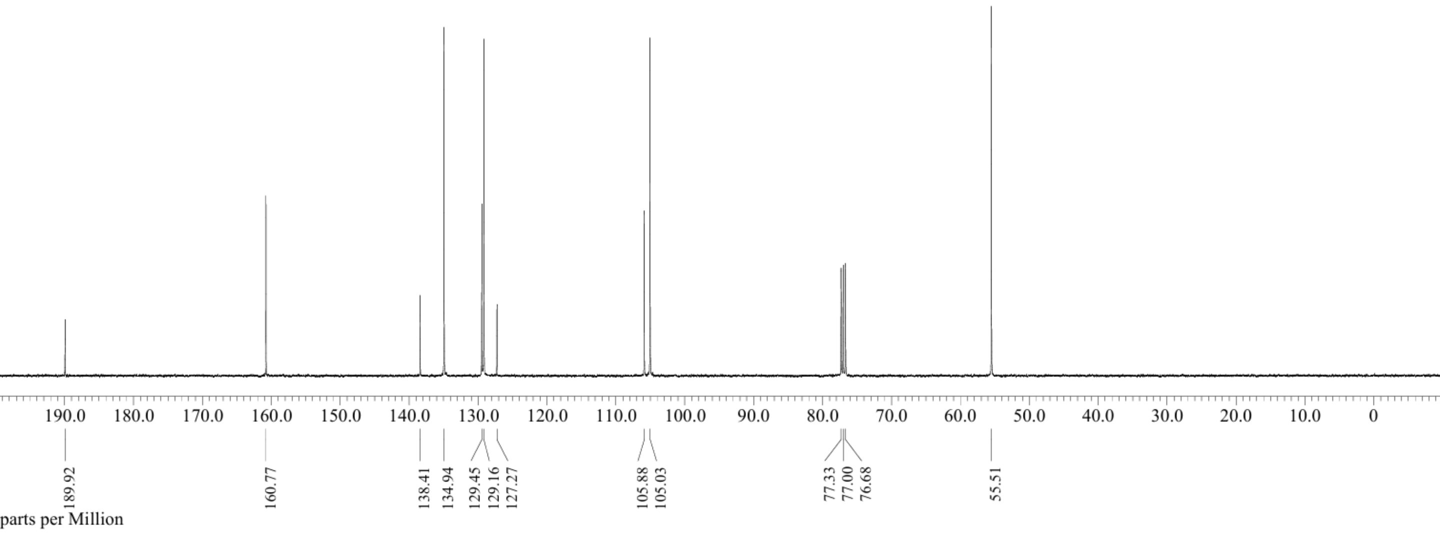

$^{13}C\{^{1}H\}$ NMR (100 MHz, $CDCl_3$)

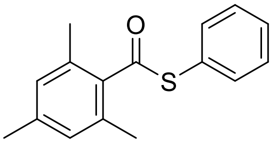

3ia

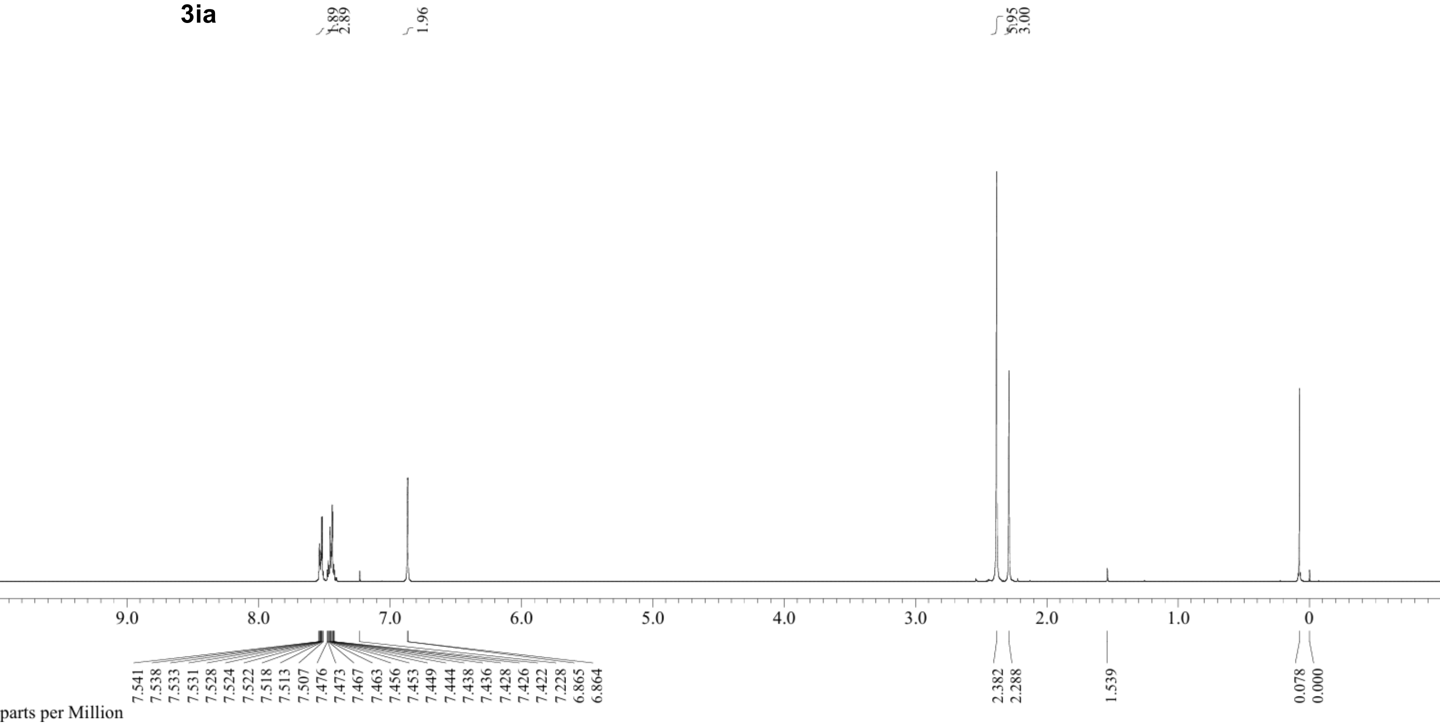

^{1}H NMR (400 MHz, $CDCl_3$)

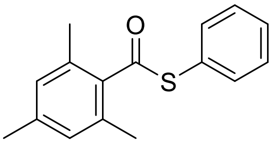

3ia

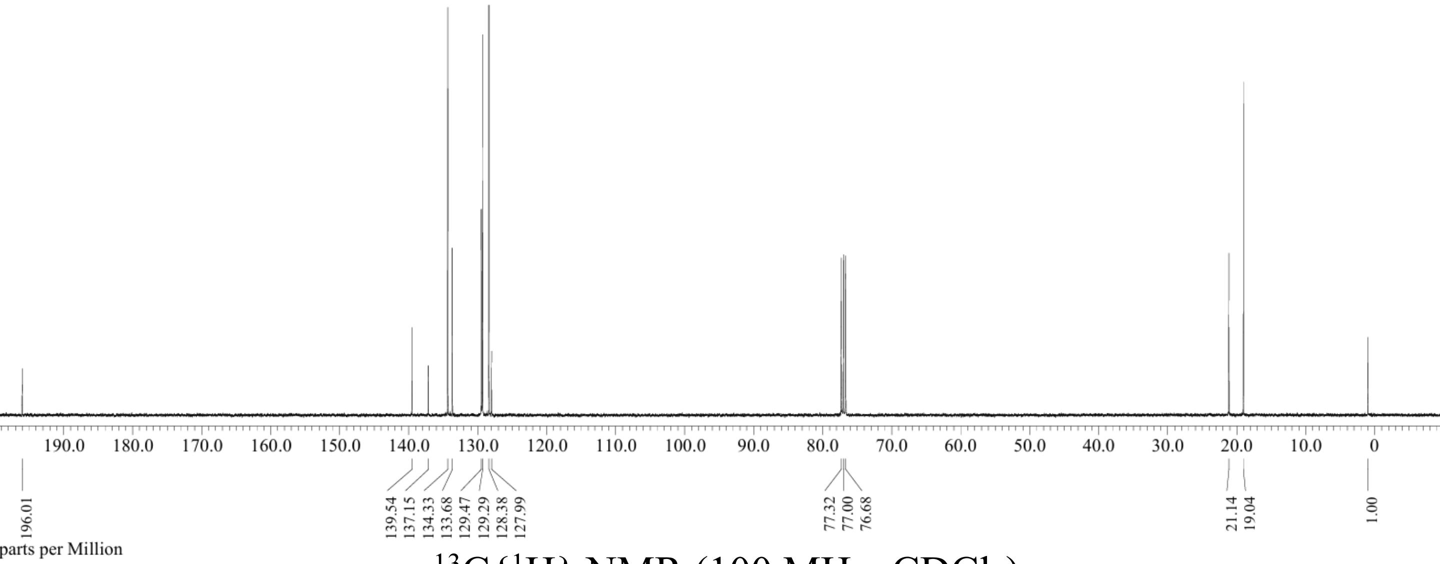

$^{13}C\{^{1}H\}$ NMR (100 MHz, $CDCl_3$)

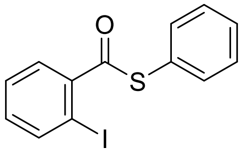

3ja

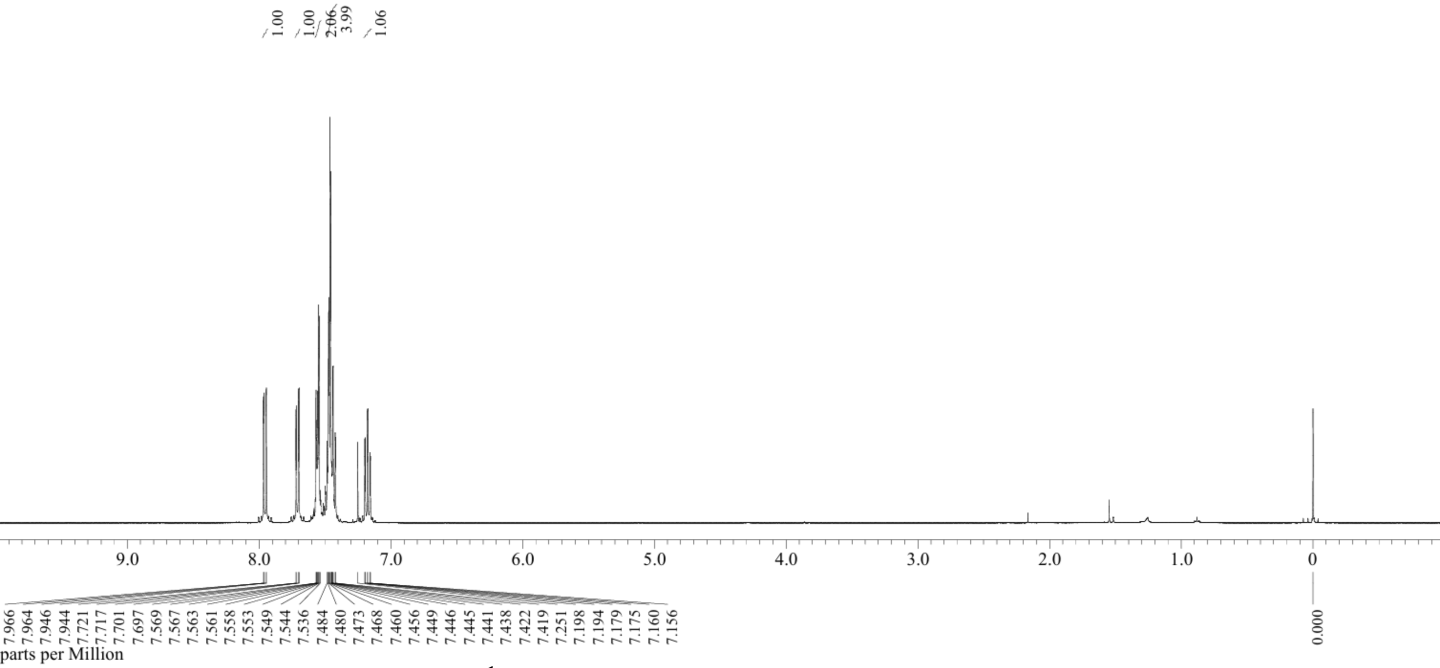

^{1}H NMR (400 MHz, $CDCl_3$)

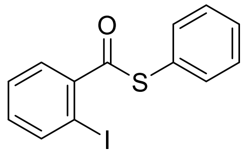

3ja

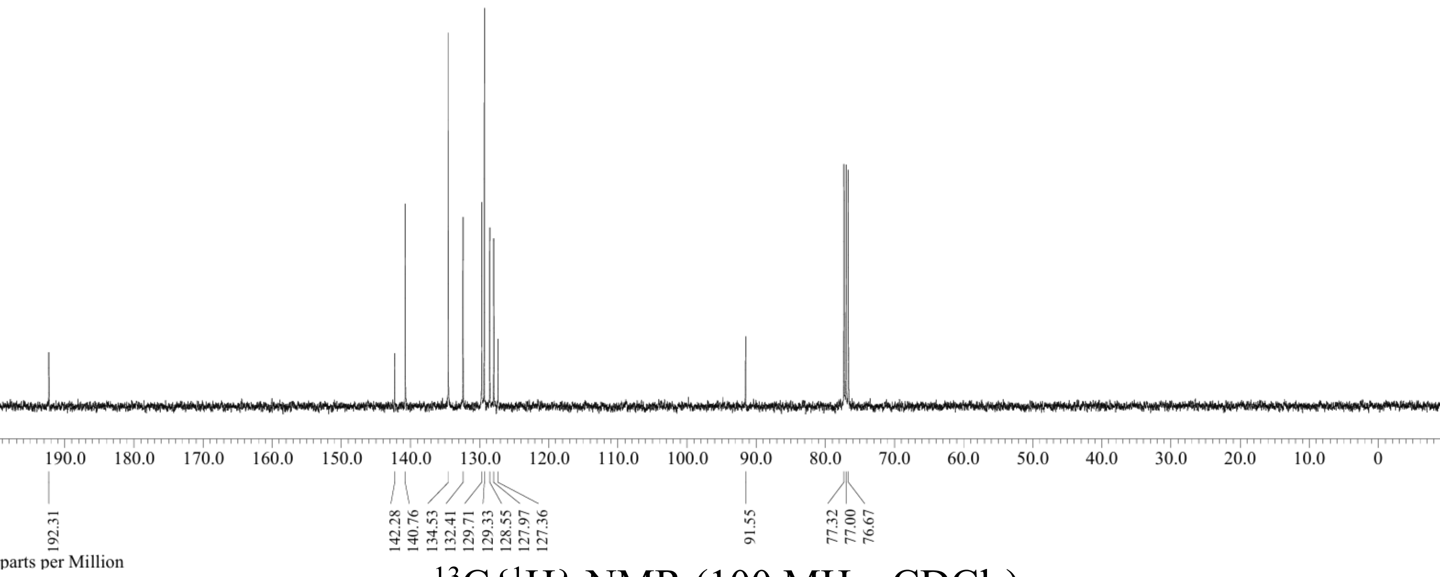

$^{13}C\{^{1}H\}$ NMR (100 MHz, $CDCl_3$)

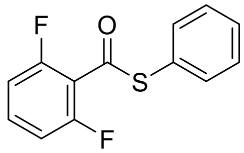

3ka

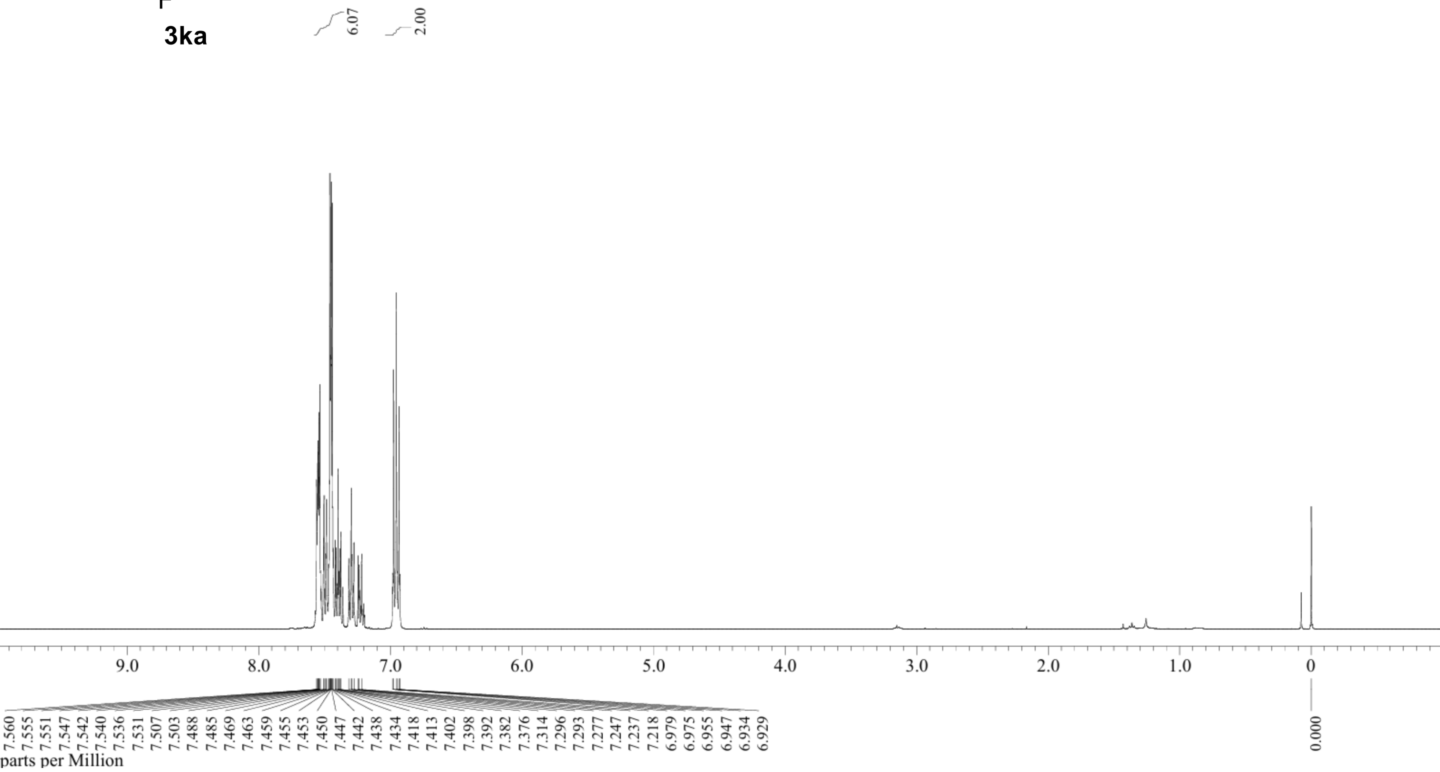

^{1}H NMR (400 MHz, $CDCl_3$)

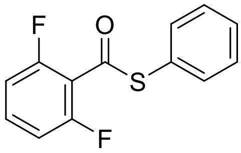

3ka

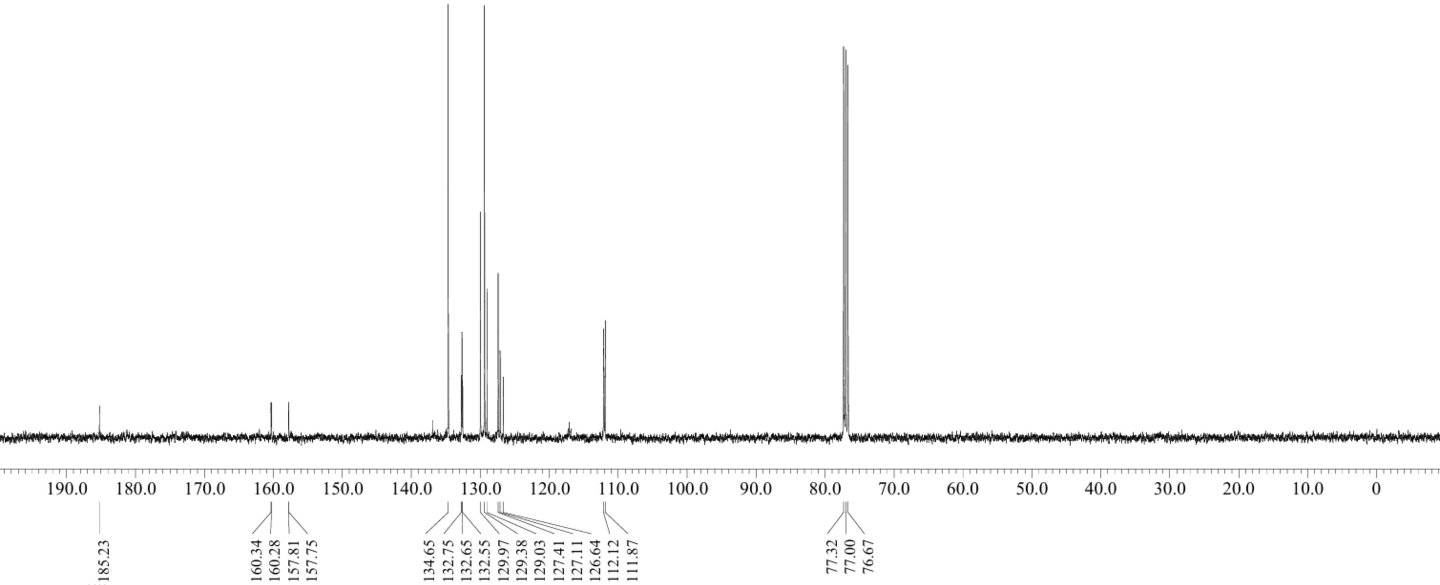

$^{13}C\{^{1}H\}$ NMR (100 MHz, $CDCl_3$)

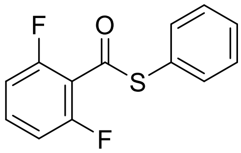

3ka

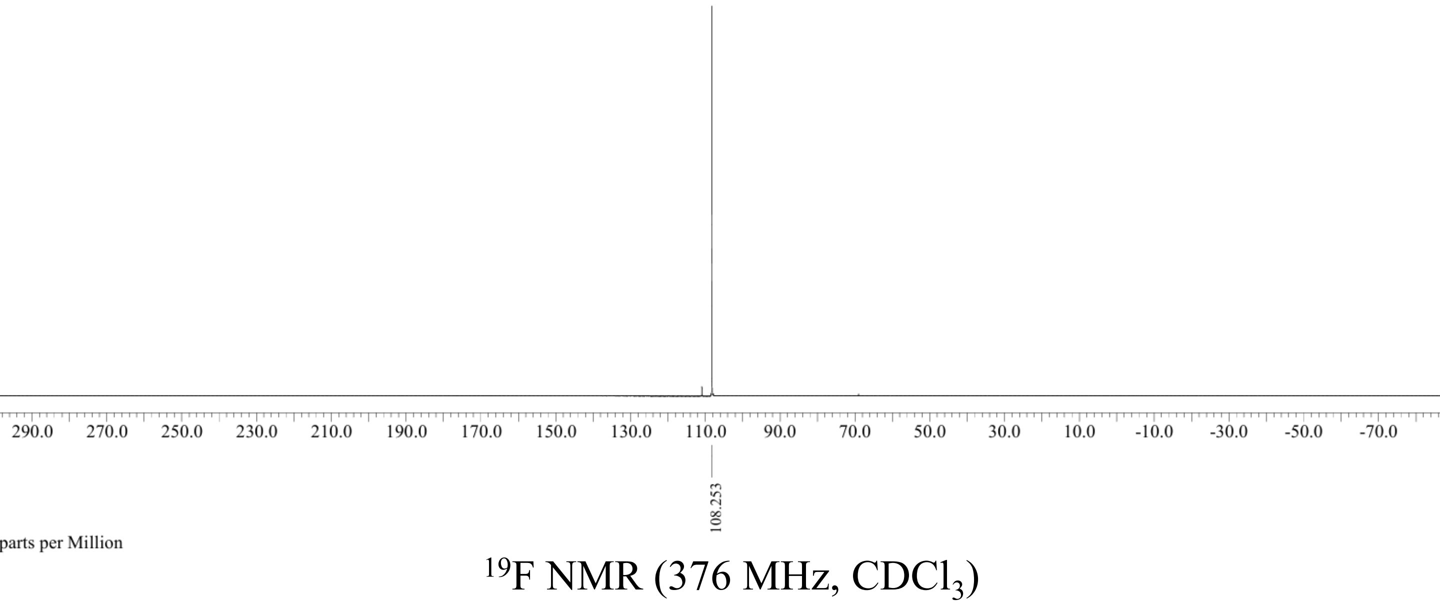

^{19}F NMR (376 MHz, $CDCl_3$)

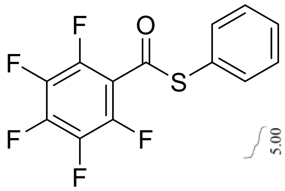

3la

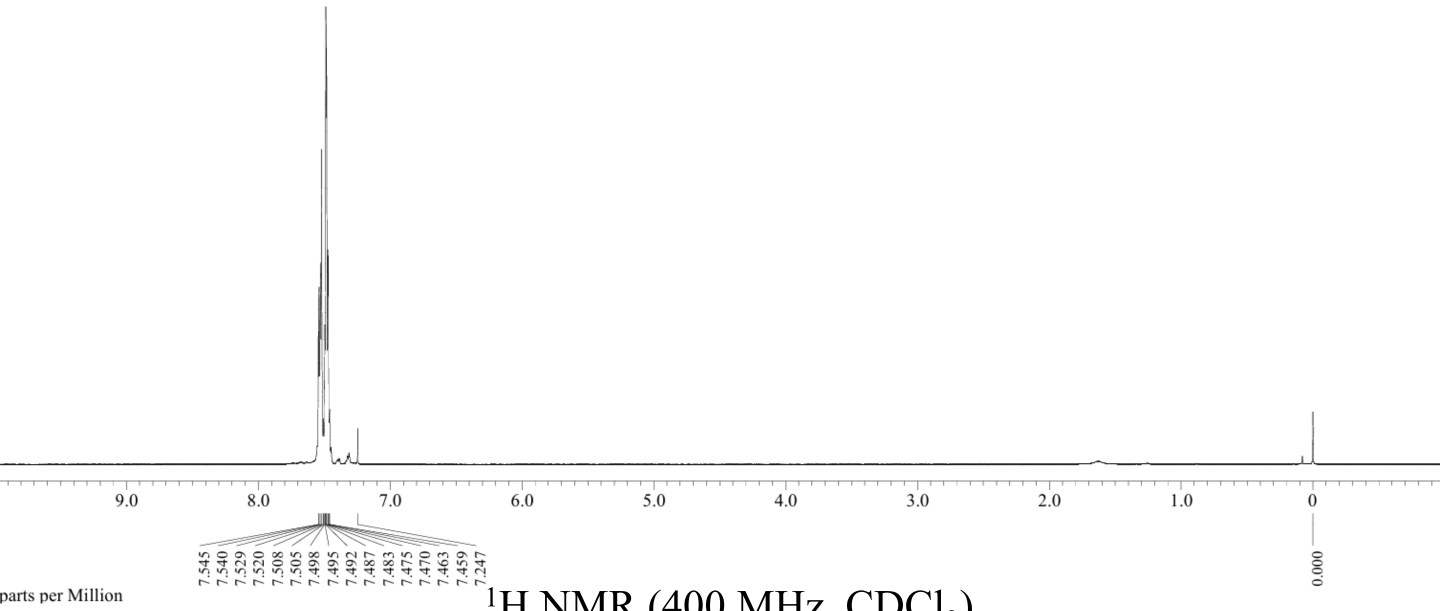

^{1}H NMR (400 MHz, $CDCl_3$)

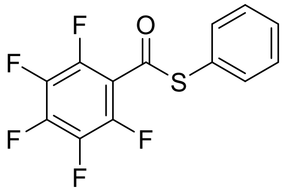

3la

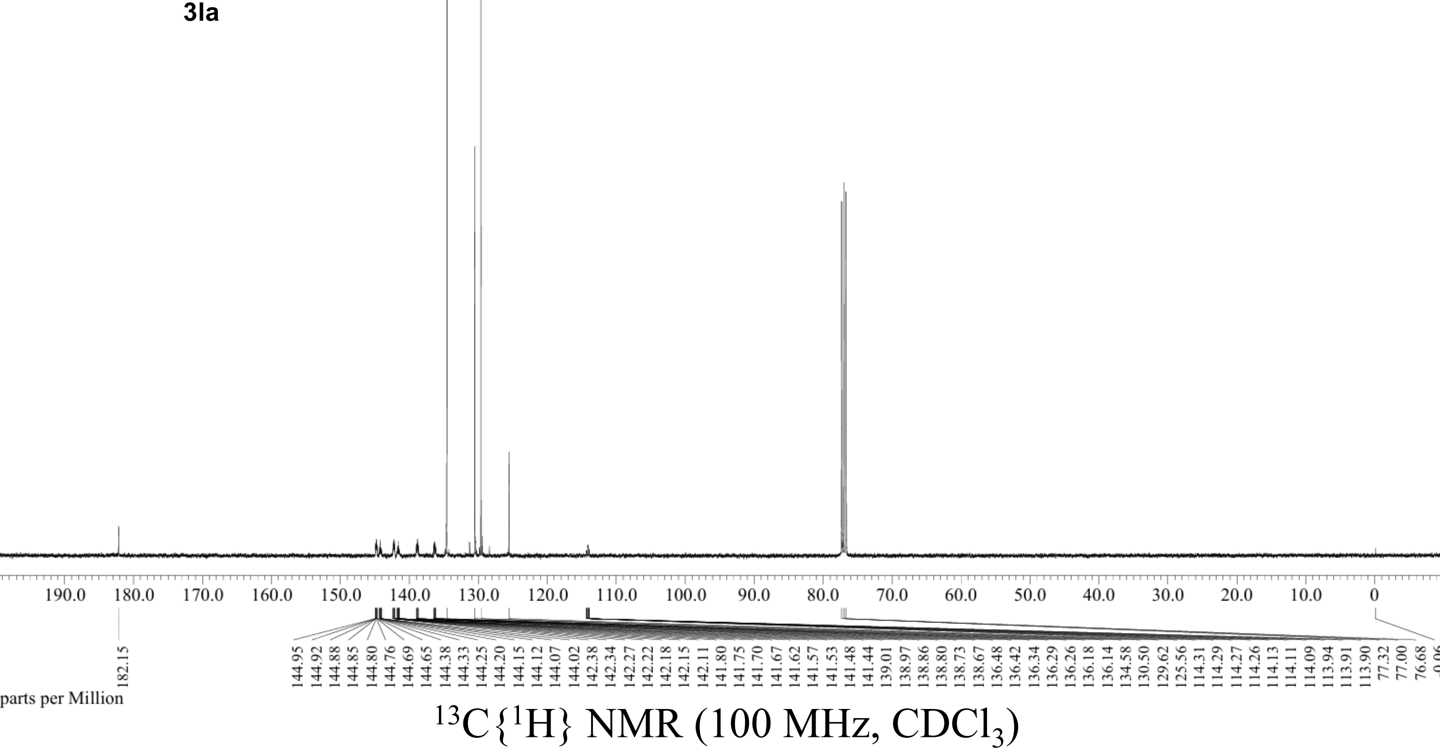

^{13}C{^{1}H} NMR (100 MHz, $CDCl_3$)

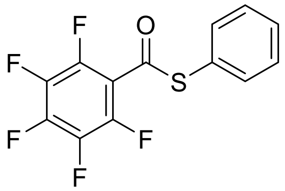

3la

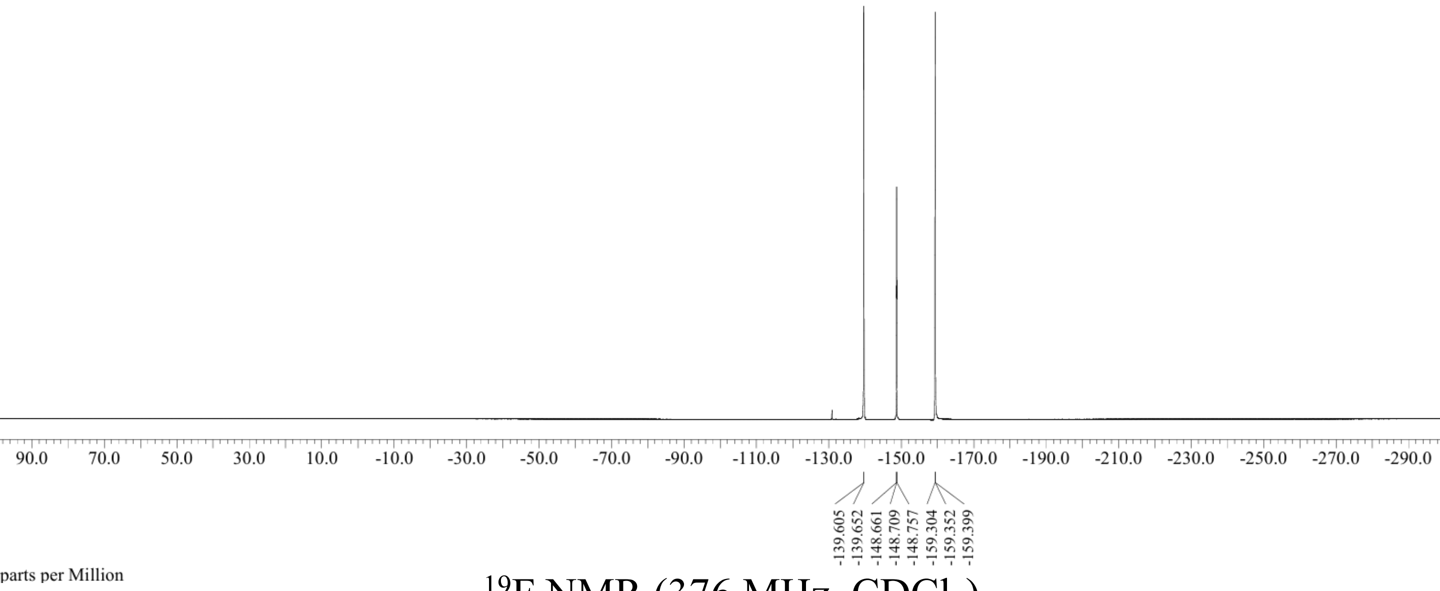

^{19}F NMR (376 MHz, $CDCl_3$)

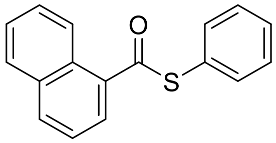

3ma

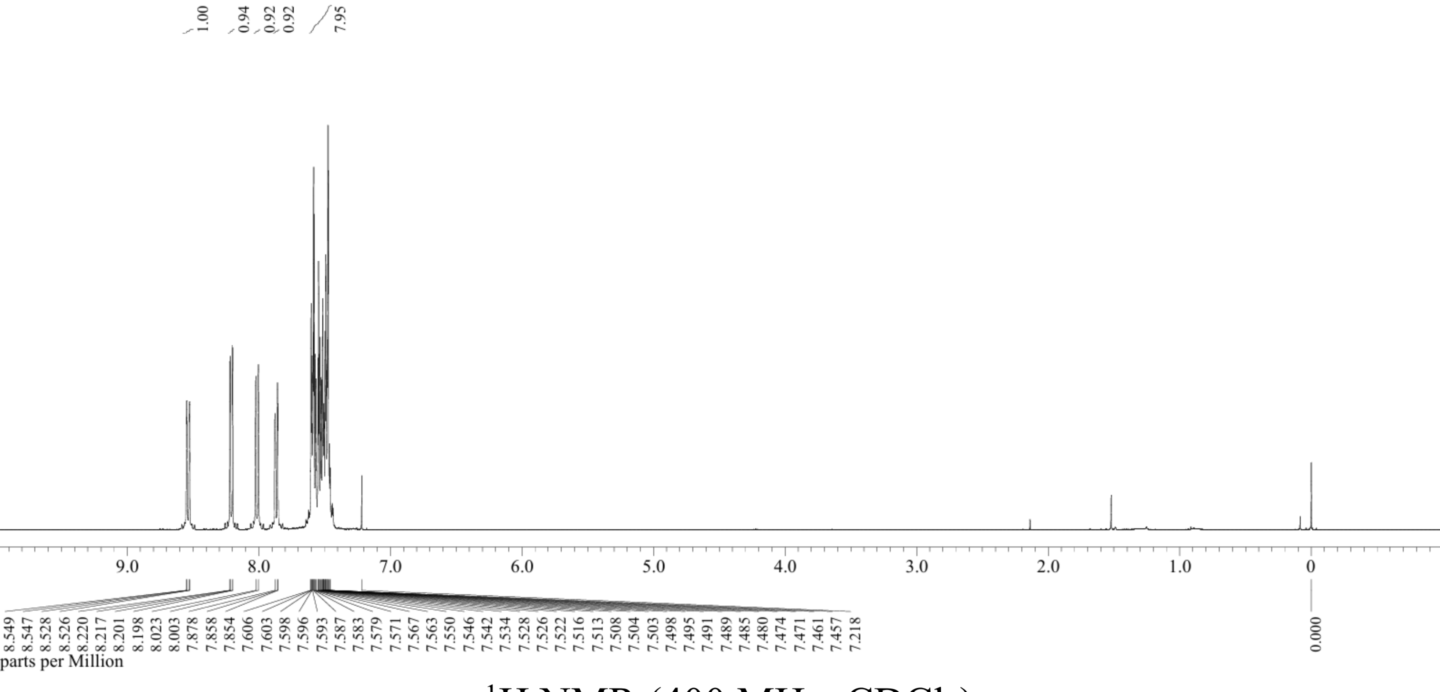

^{1}H NMR (400 MHz, $CDCl_3$)

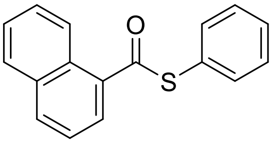

3ma

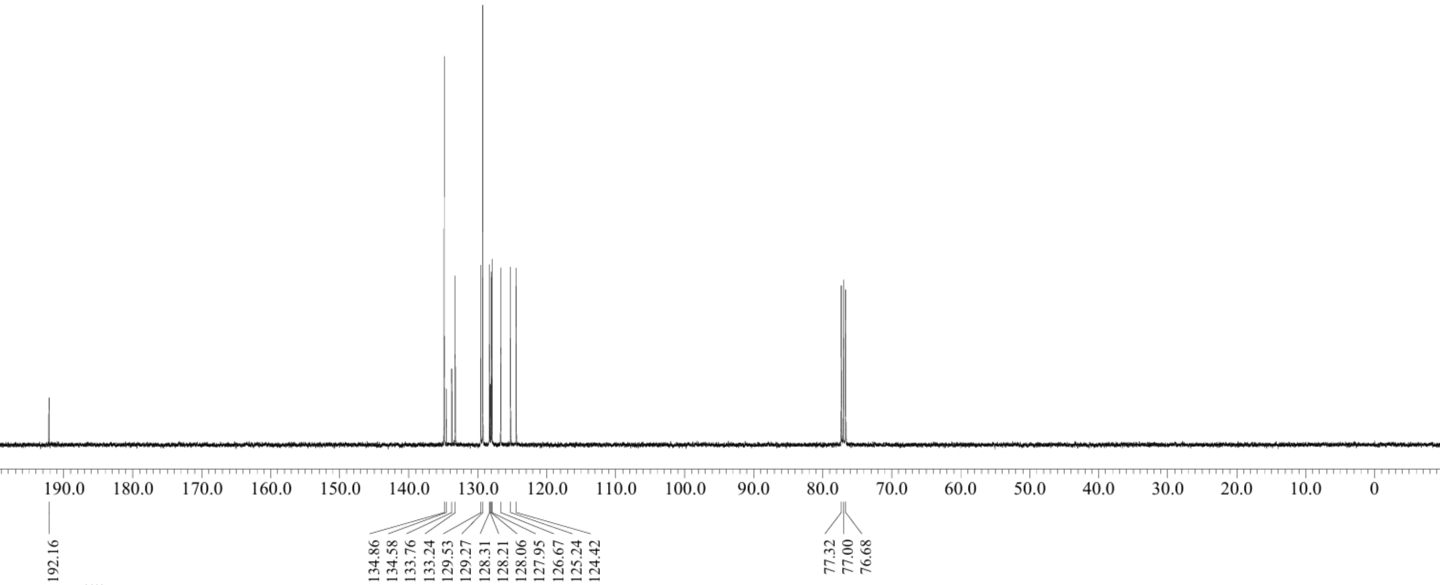

$^{13}C\{^{1}H\}$ NMR (100 MHz, $CDCl_3$)

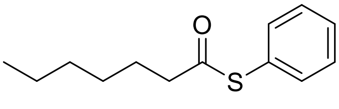

3na

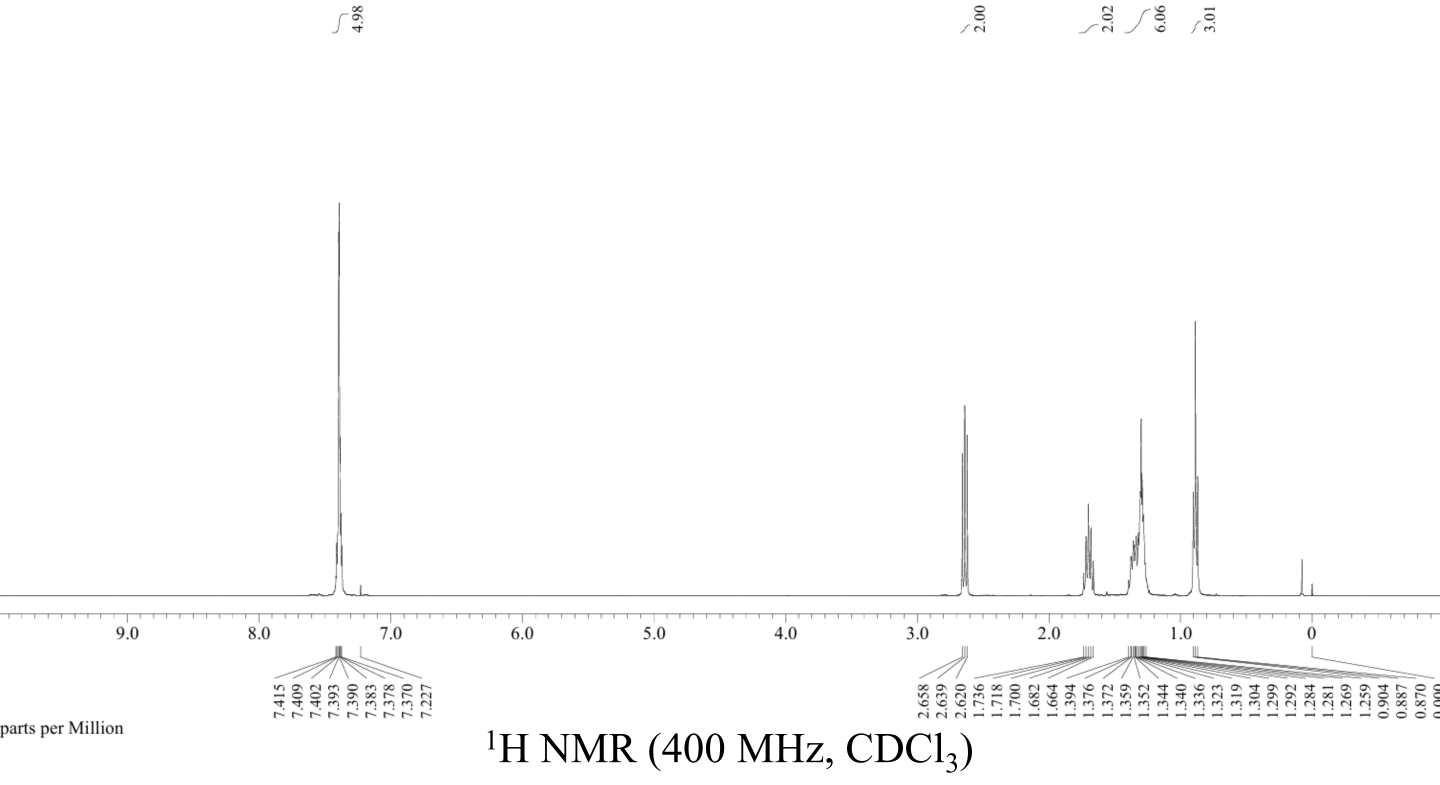

^{1}H NMR (400 MHz, $CDCl_3$)

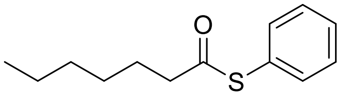

3na

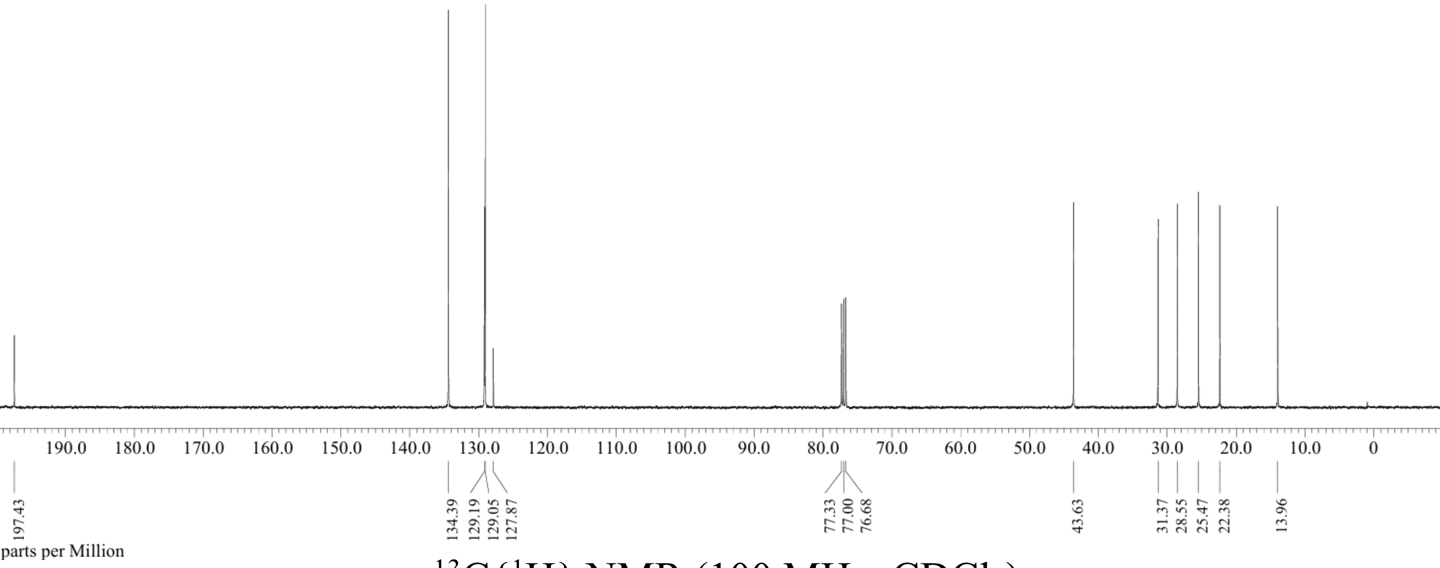

$^{13}C\{^{1}H\}$ NMR (100 MHz, $CDCl_3$)

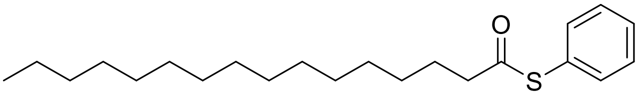

3oa

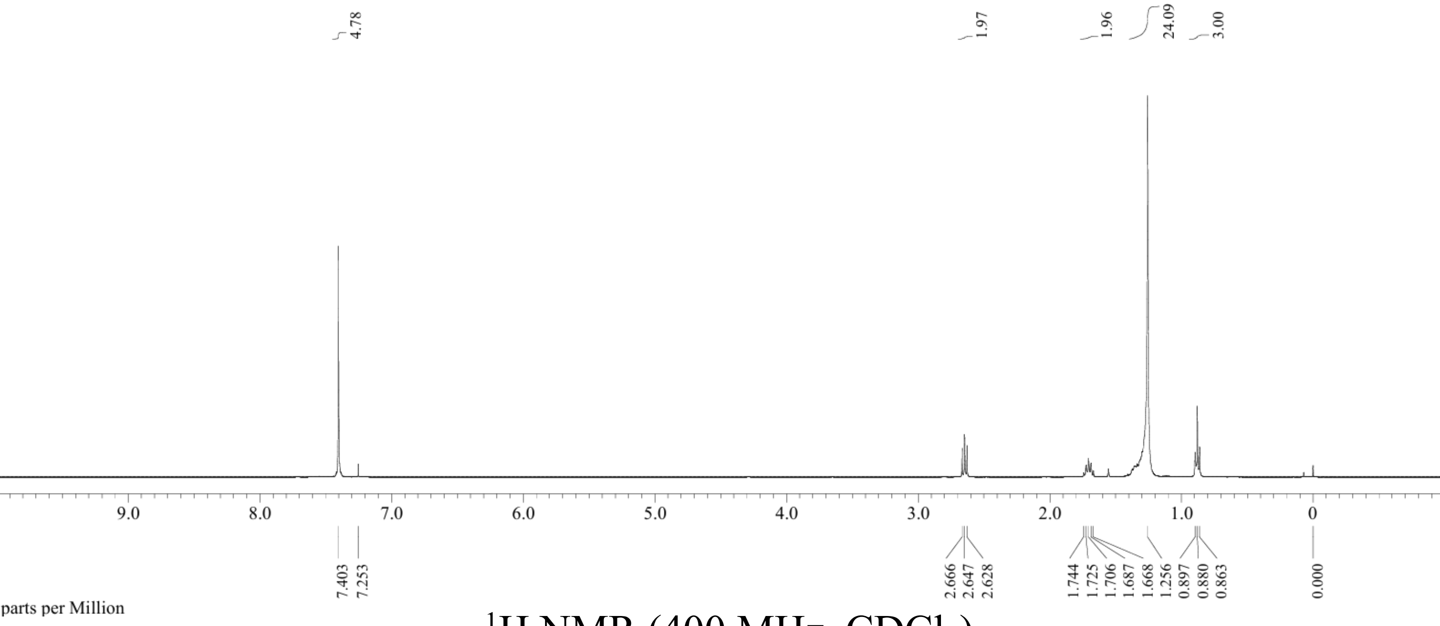

^{1}H NMR (400 MHz, $CDCl_3$)

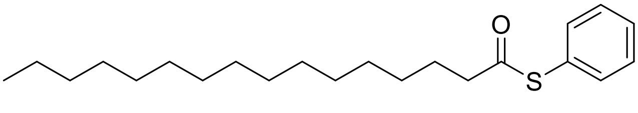

3oa

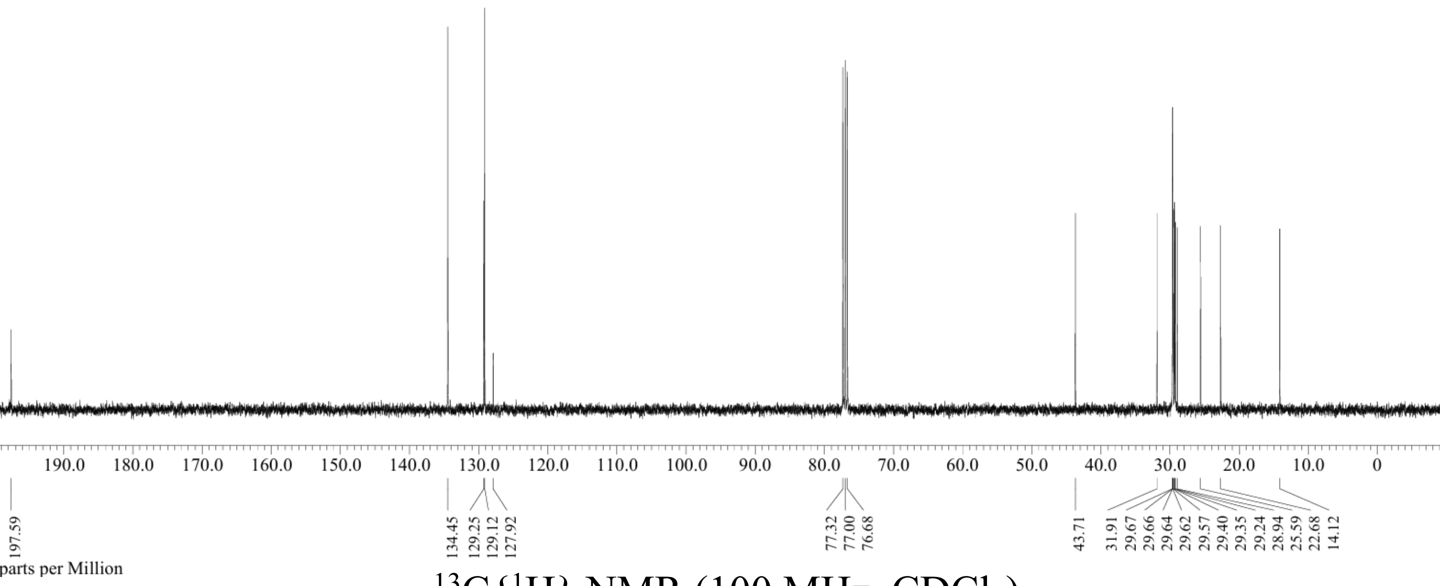

$^{13}C\{^{1}H\}$ NMR (100 MHz, $CDCl_3$)

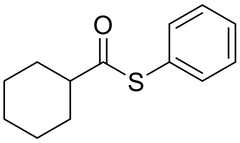

3pa

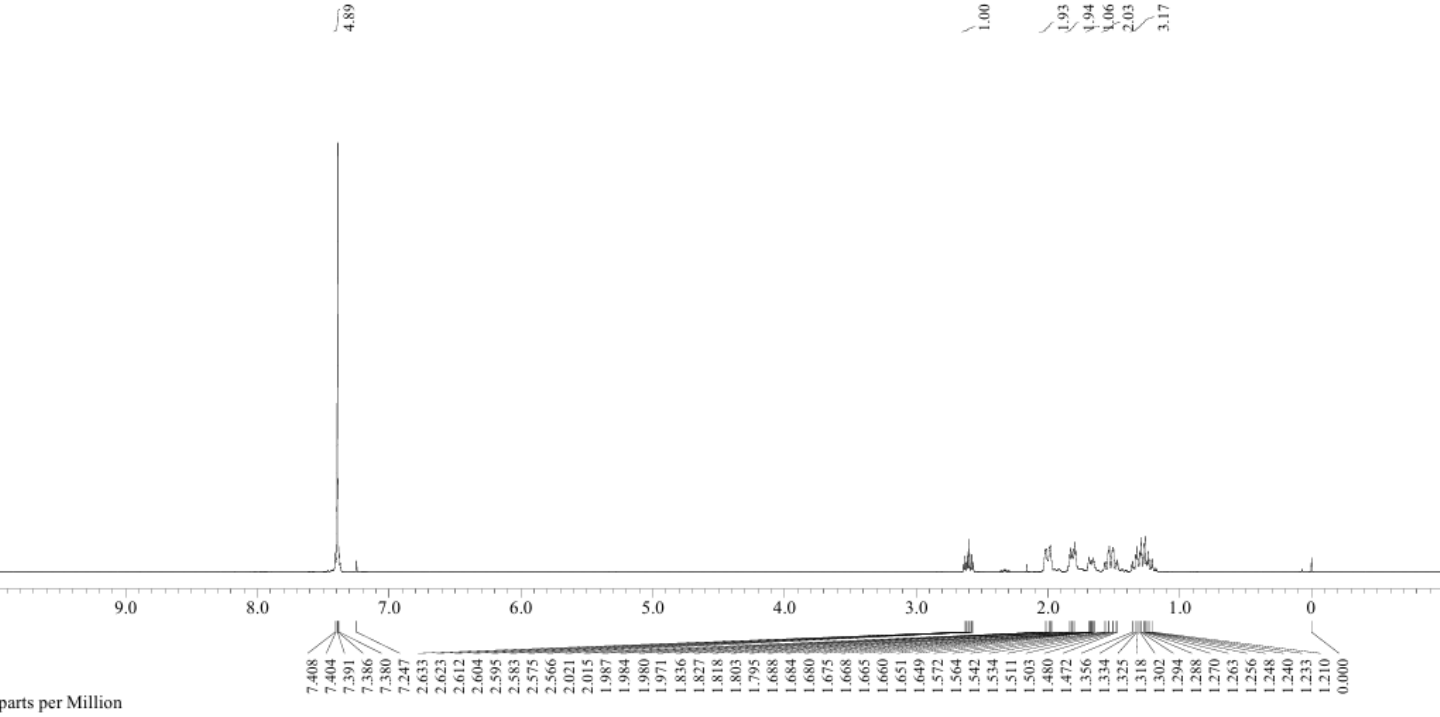

^{1}H NMR (400 MHz, $CDCl_3$)

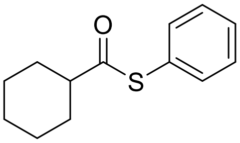

3pa

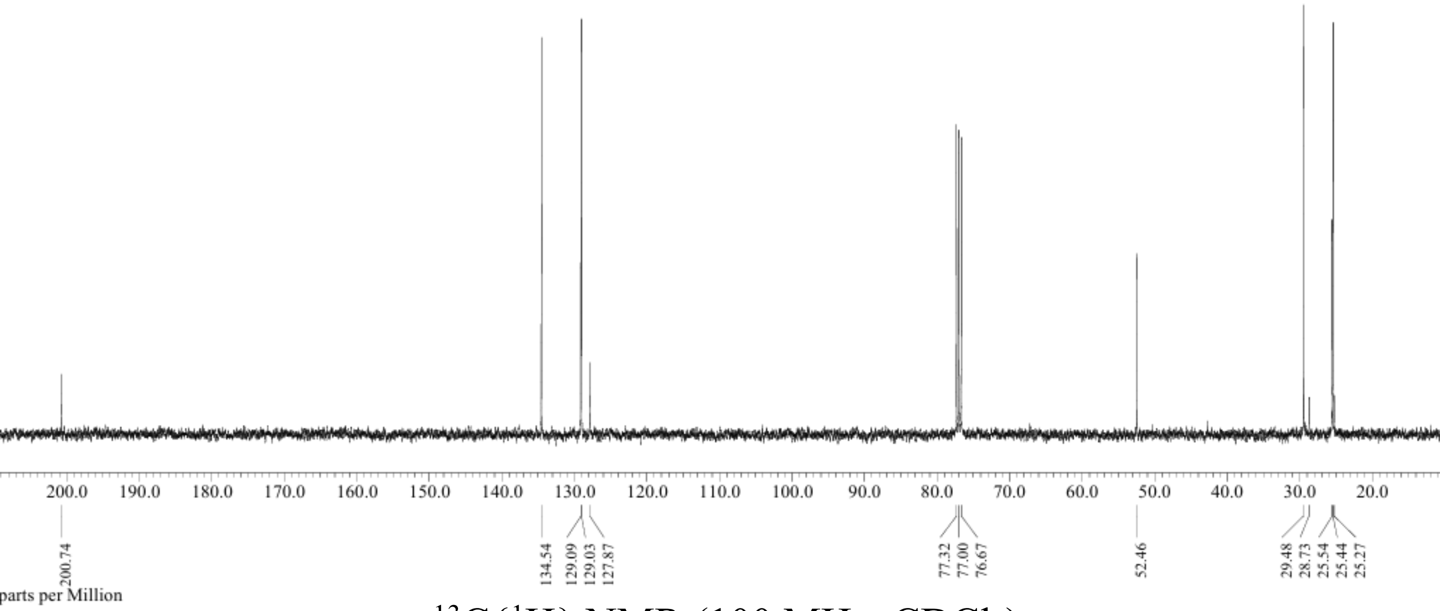

$^{13}C\{^{1}H\}$ NMR (100 MHz, $CDCl_3$)

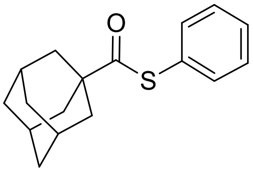

3qa

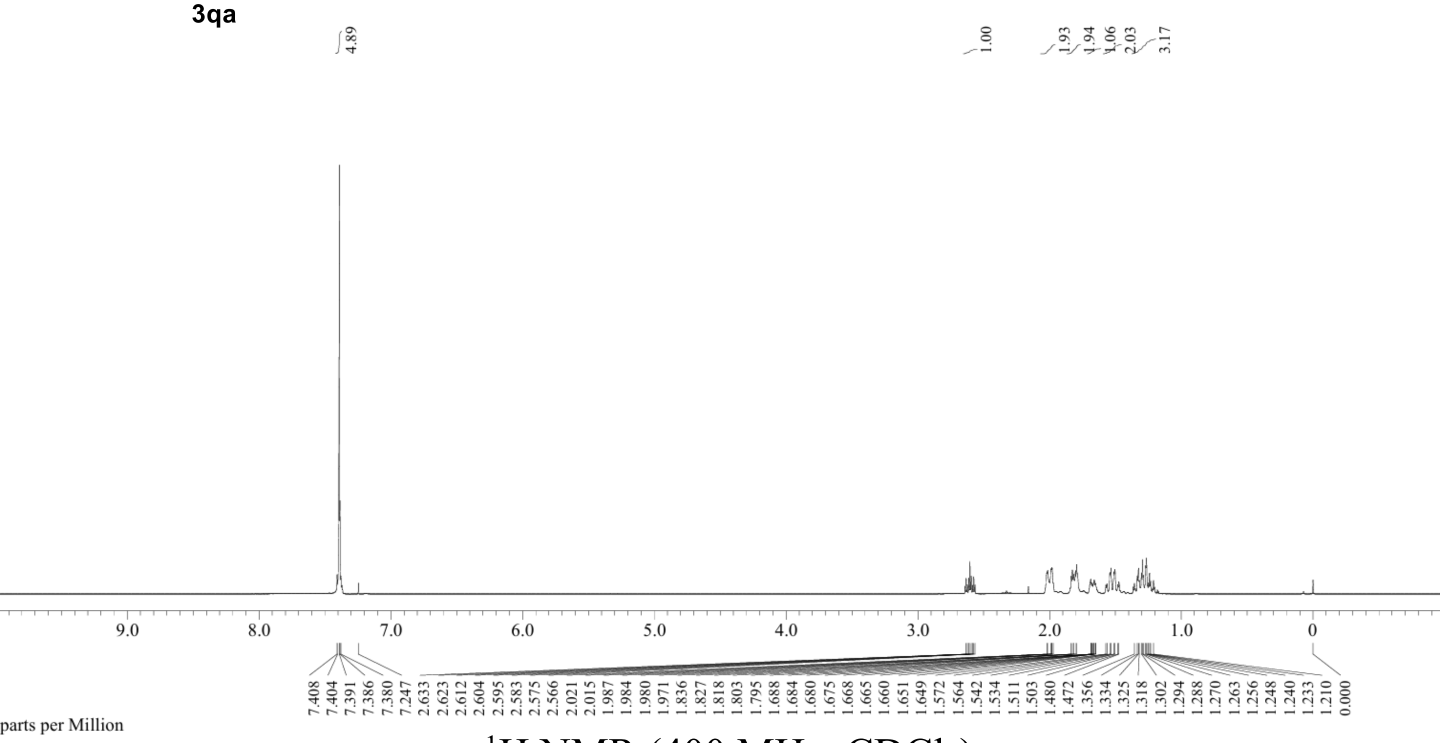

^{1}H NMR (400 MHz, $CDCl_3$)

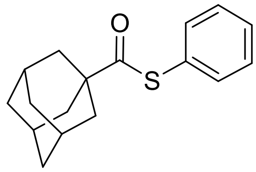

3qa

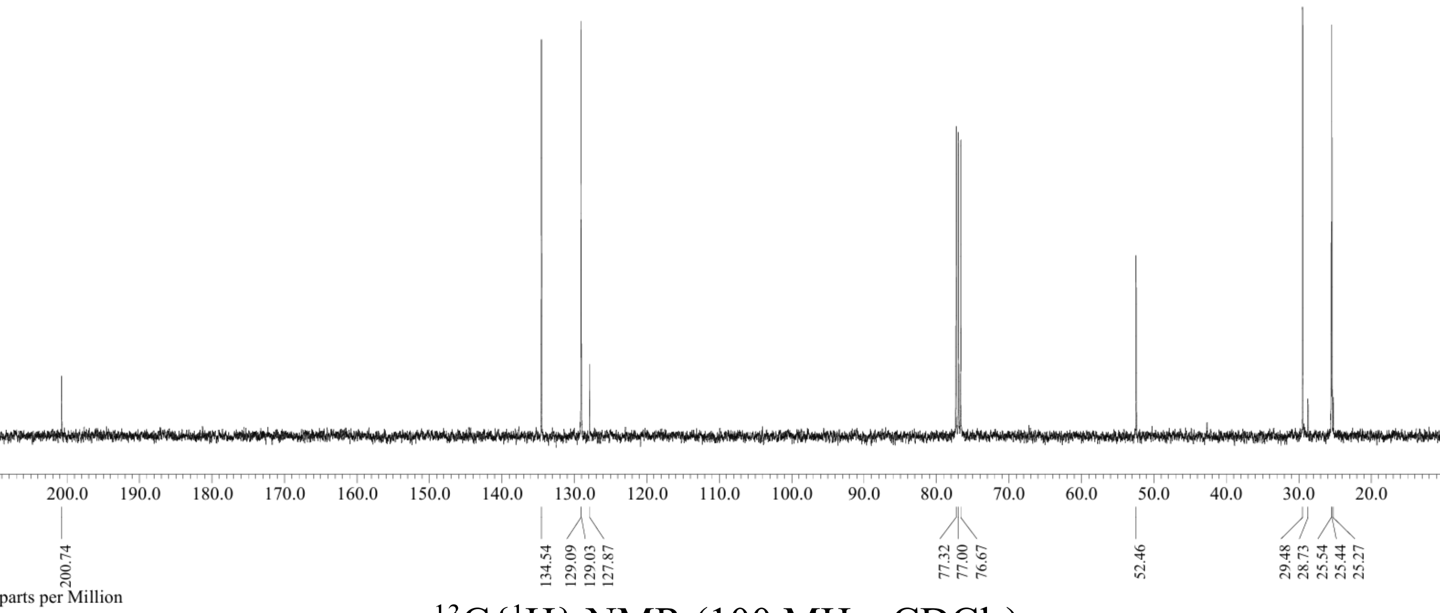

$^{13}C\{^1H\}$ NMR (100 MHz, $CDCl_3$)

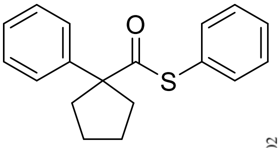

3ra

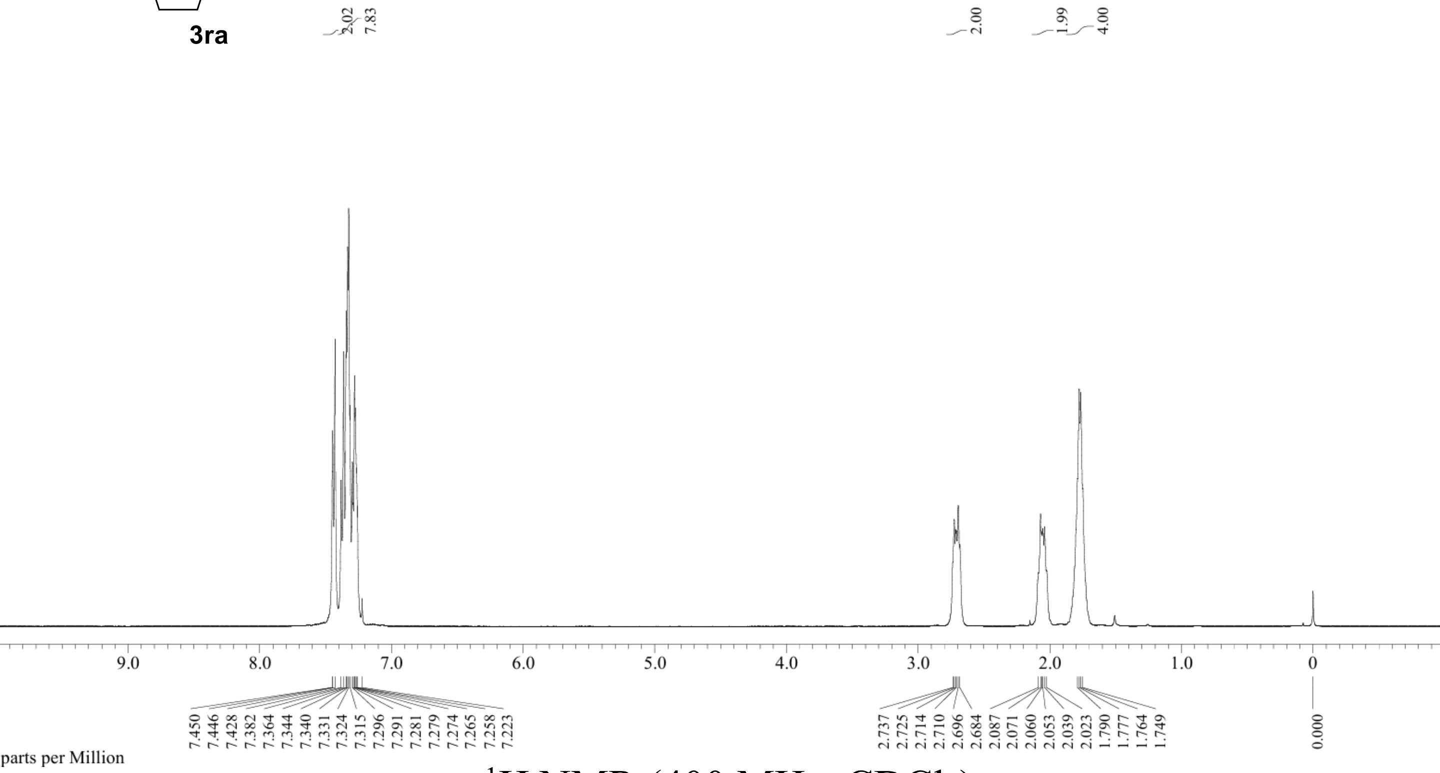

^1H NMR (400 MHz, $CDCl_3$)

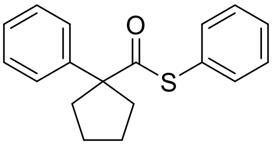

3ra

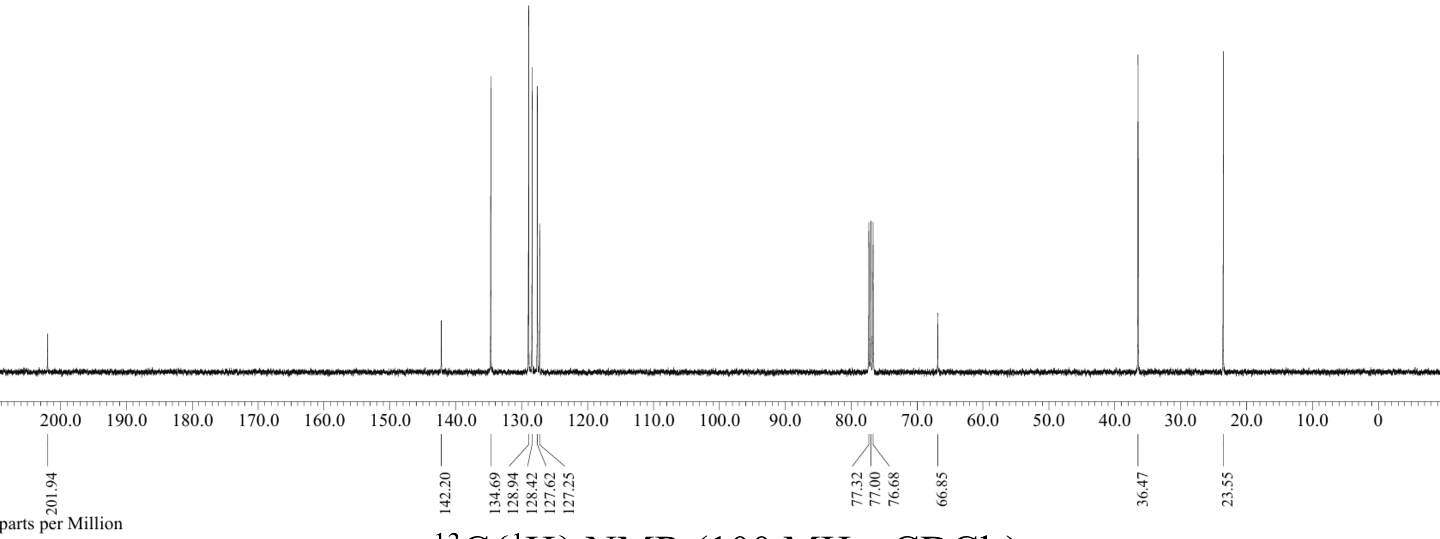

$^{13}C\{^{1}H\}$ NMR (100 MHz, $CDCl_3$)

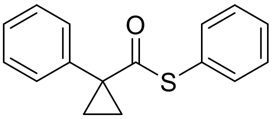

3sa

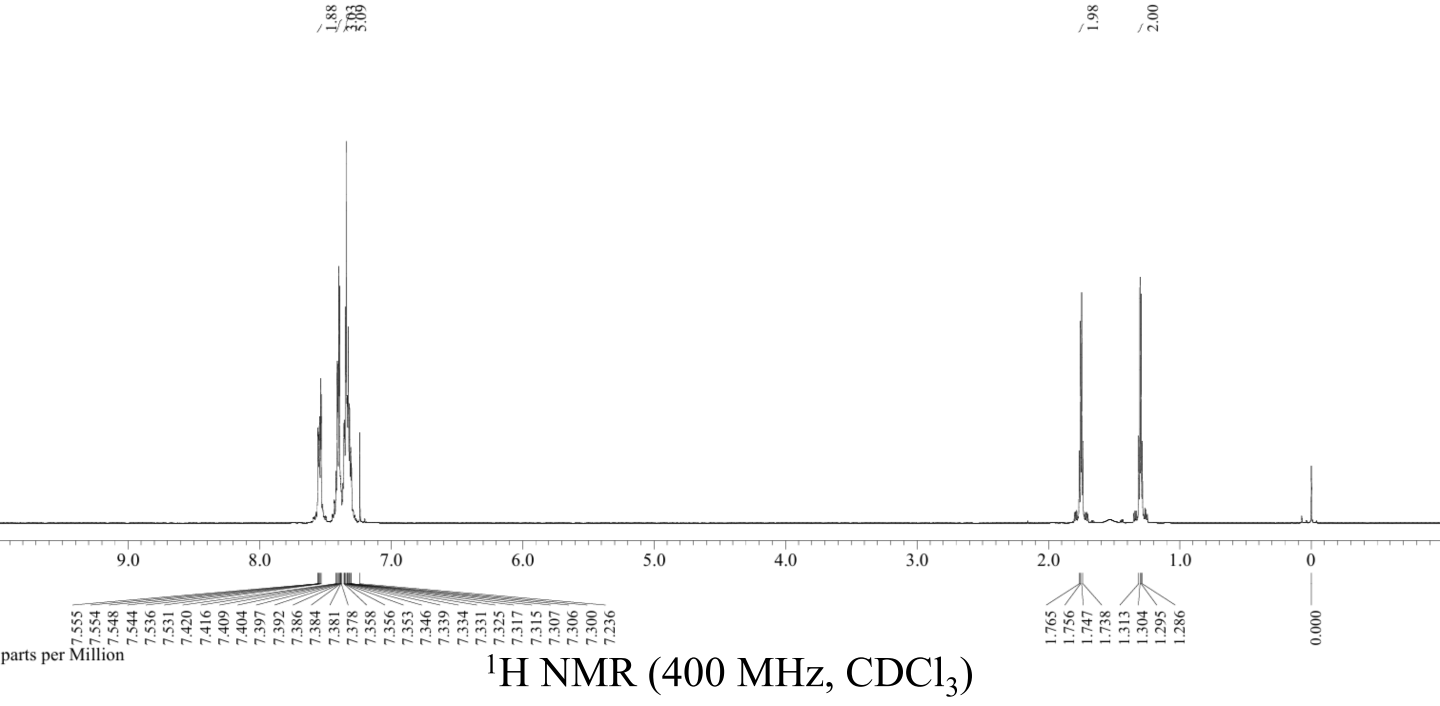

^{1}H NMR (400 MHz, $CDCl_3$)

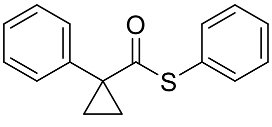

3sa

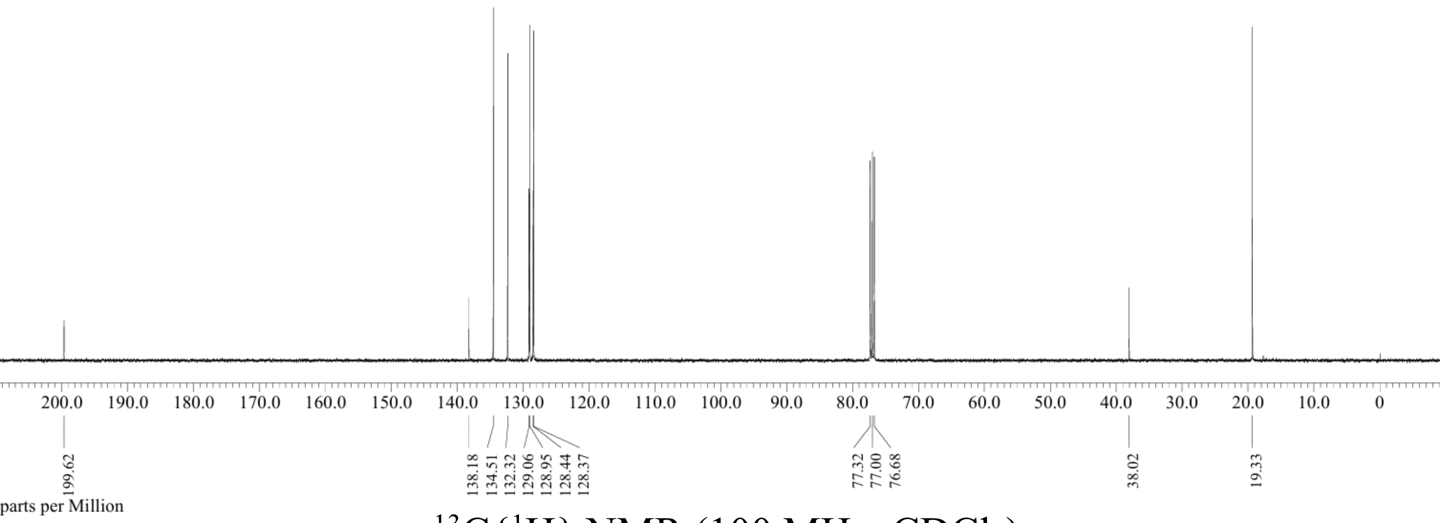

$^{13}C\{^{1}H\}$ NMR (100 MHz, $CDCl_3$)

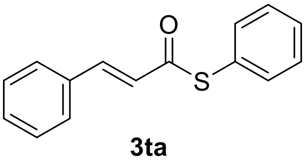

3ta

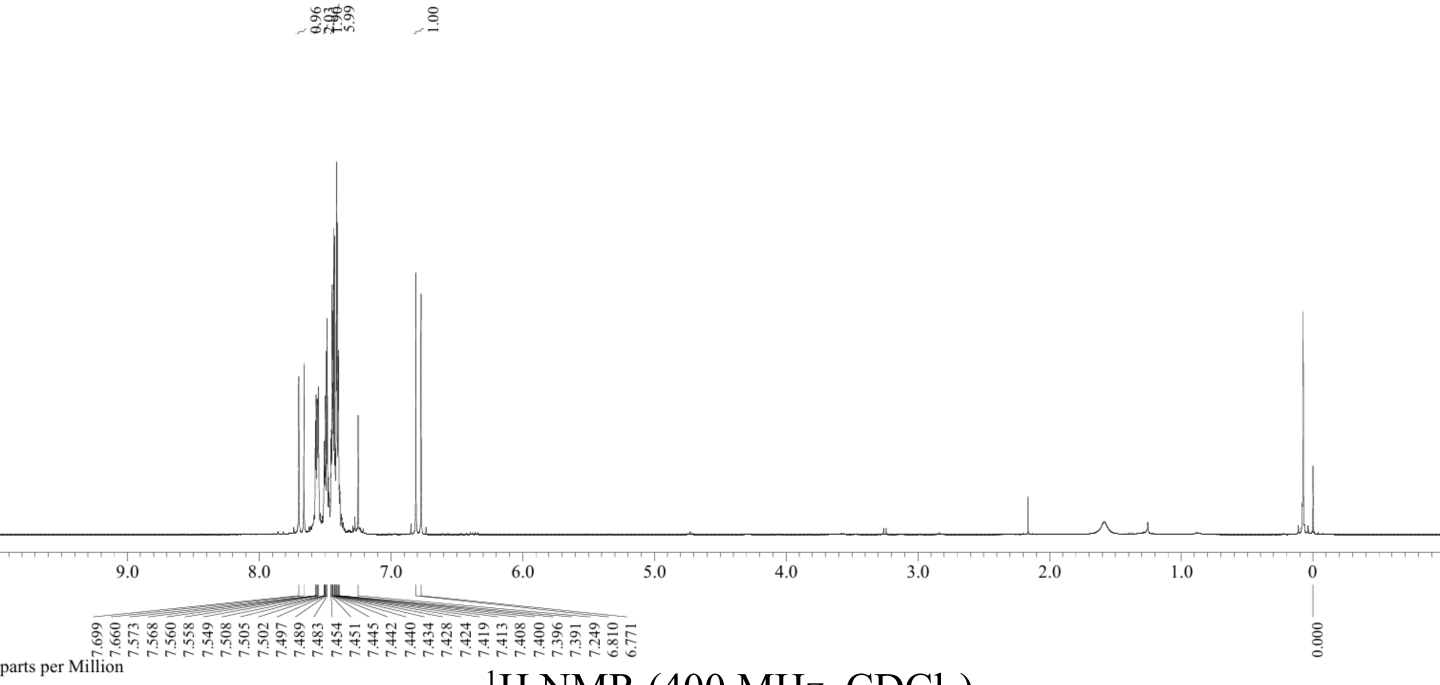

^{1}H NMR (400 MHz, $CDCl_3$)

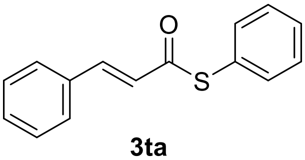

3ta

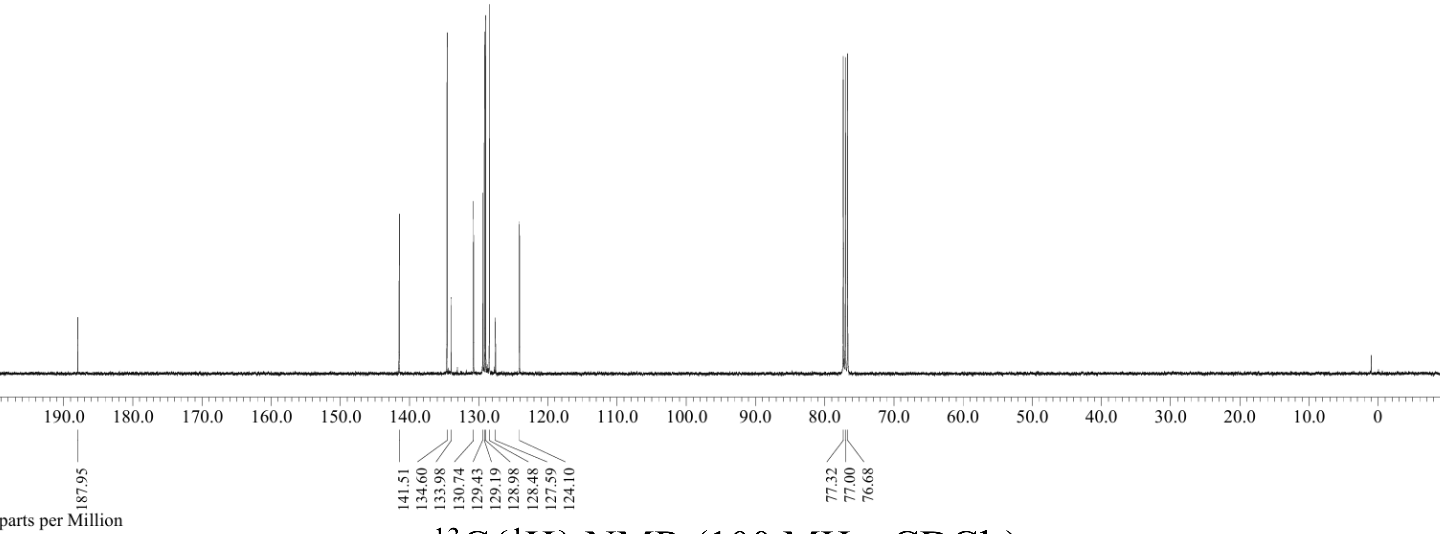

$^{13}C\{^{1}H\}$ NMR (100 MHz, $CDCl_3$)

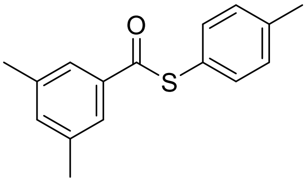

3ab

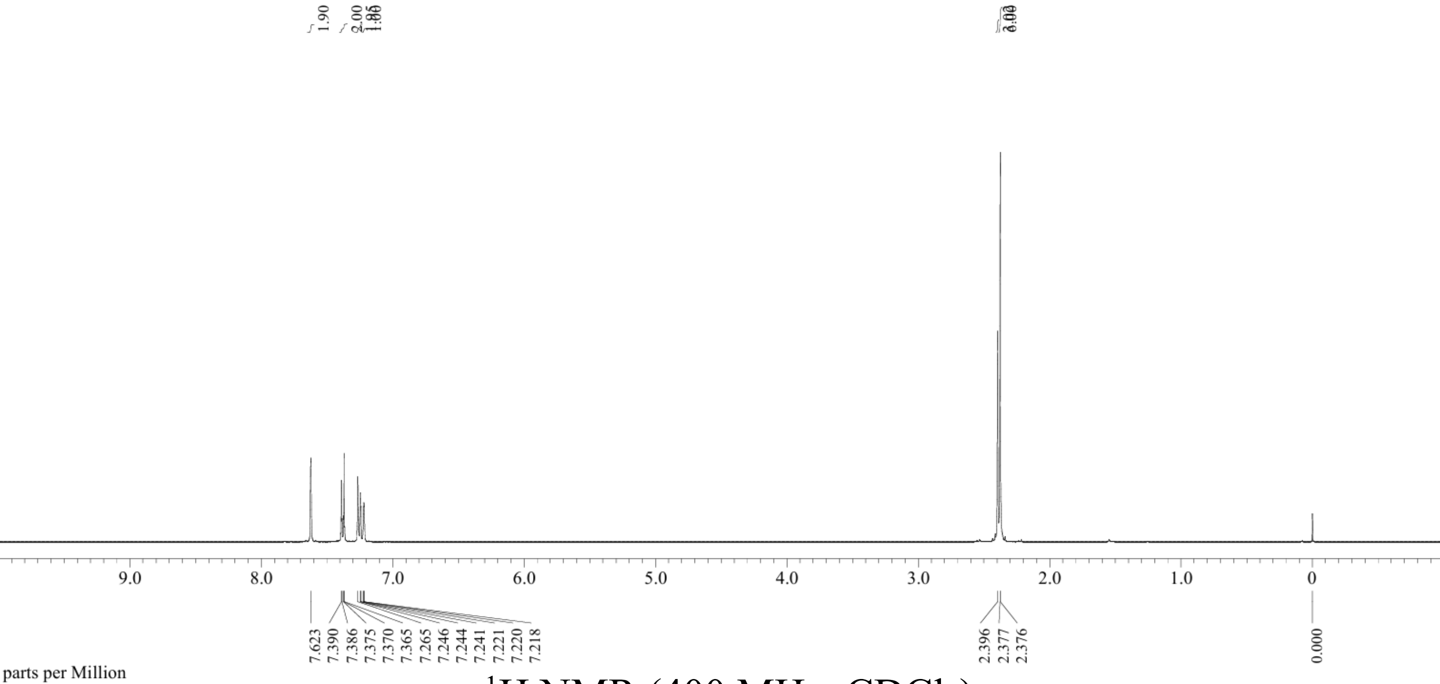

^{1}H NMR (400 MHz, $CDCl_3$)

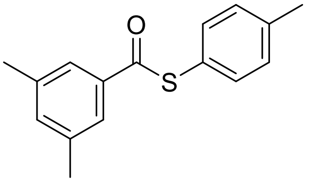

3ab

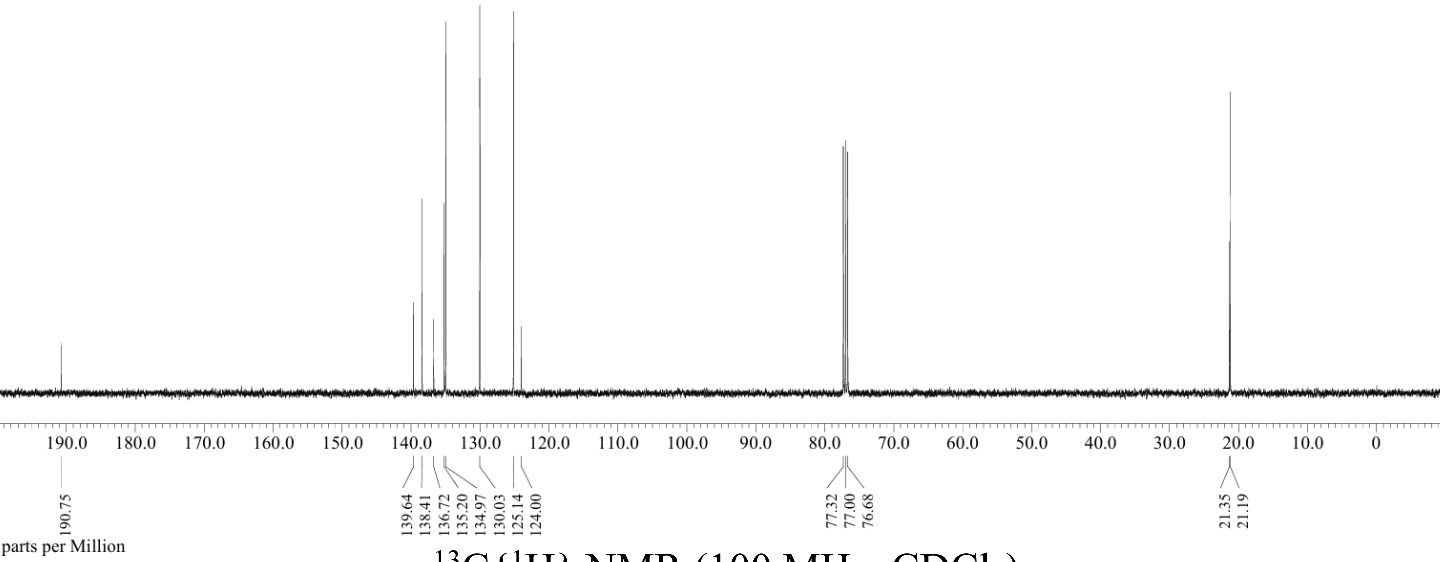

$^{13}C\{^{1}H\}$ NMR (100 MHz, $CDCl_3$)

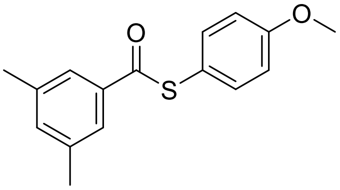

3ac

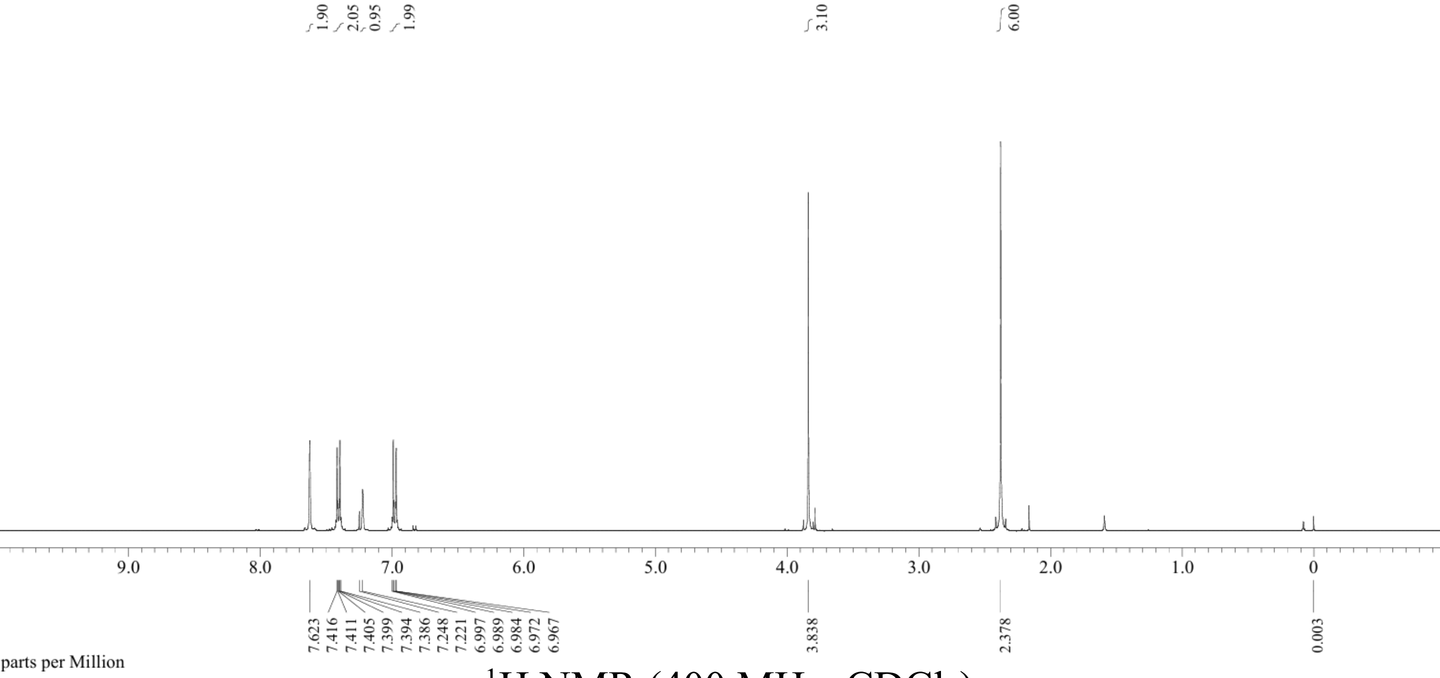

^{1}H NMR (400 MHz, $CDCl_3$)

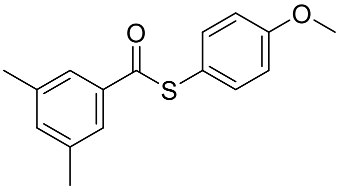

3ac

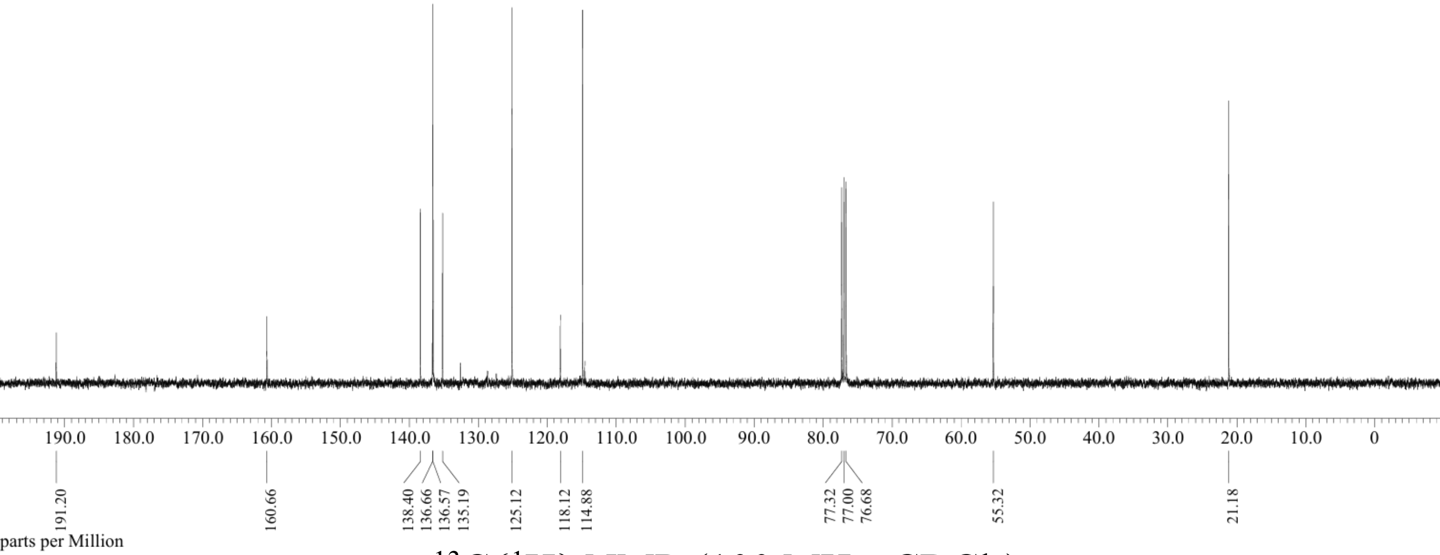

$^{13}C\{^{1}H\}$ NMR (100 MHz, $CDCl_3$)

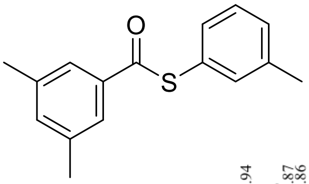

3ad

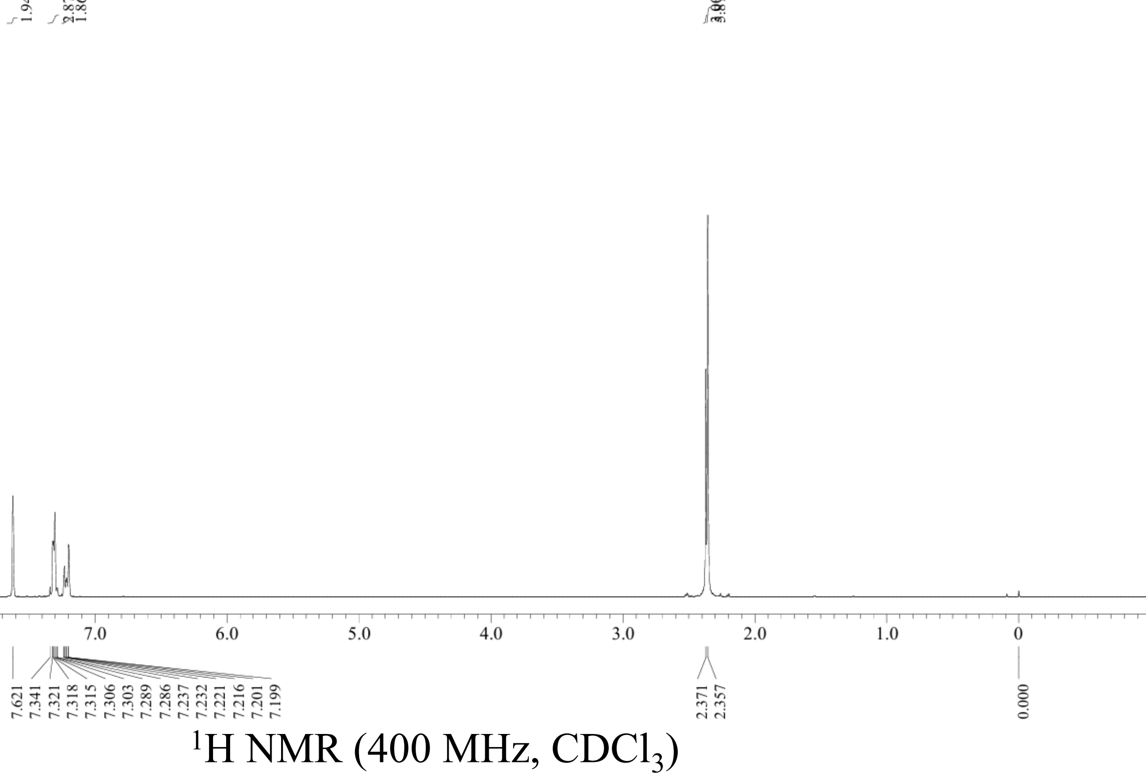

^{1}H NMR (400 MHz, $CDCl_3$)

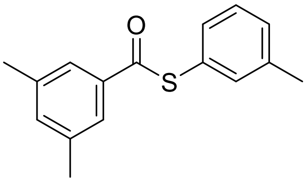

3ad

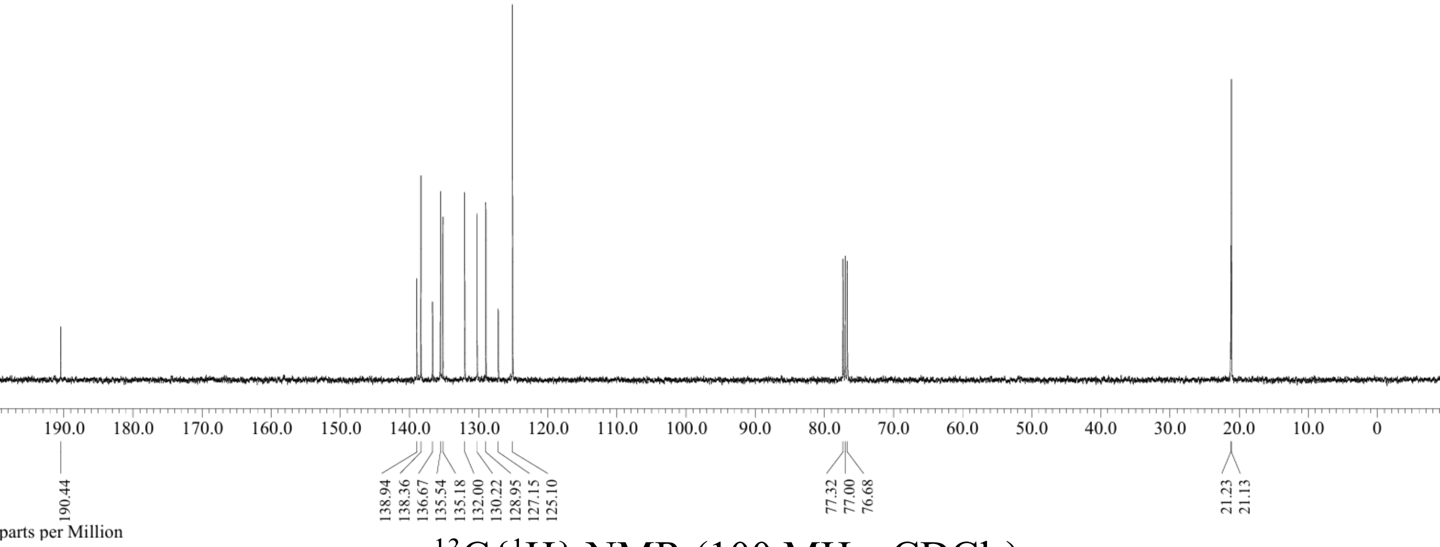

$^{13}C\{^{1}H\}$ NMR (100 MHz, $CDCl_3$)

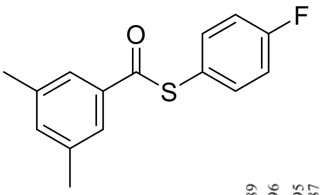

3ae

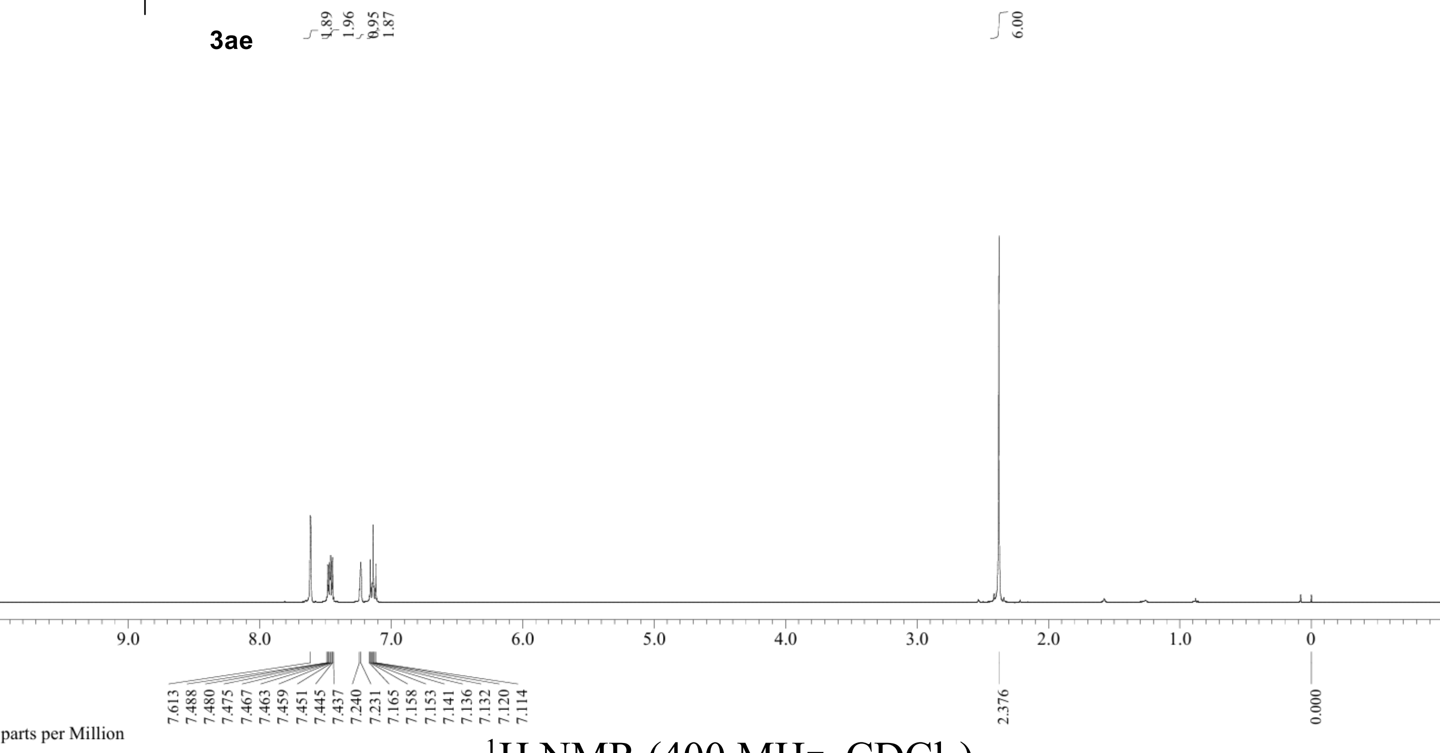

^{1}H NMR (400 MHz, $CDCl_3$)

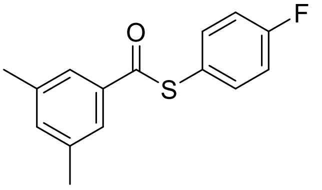

3ae

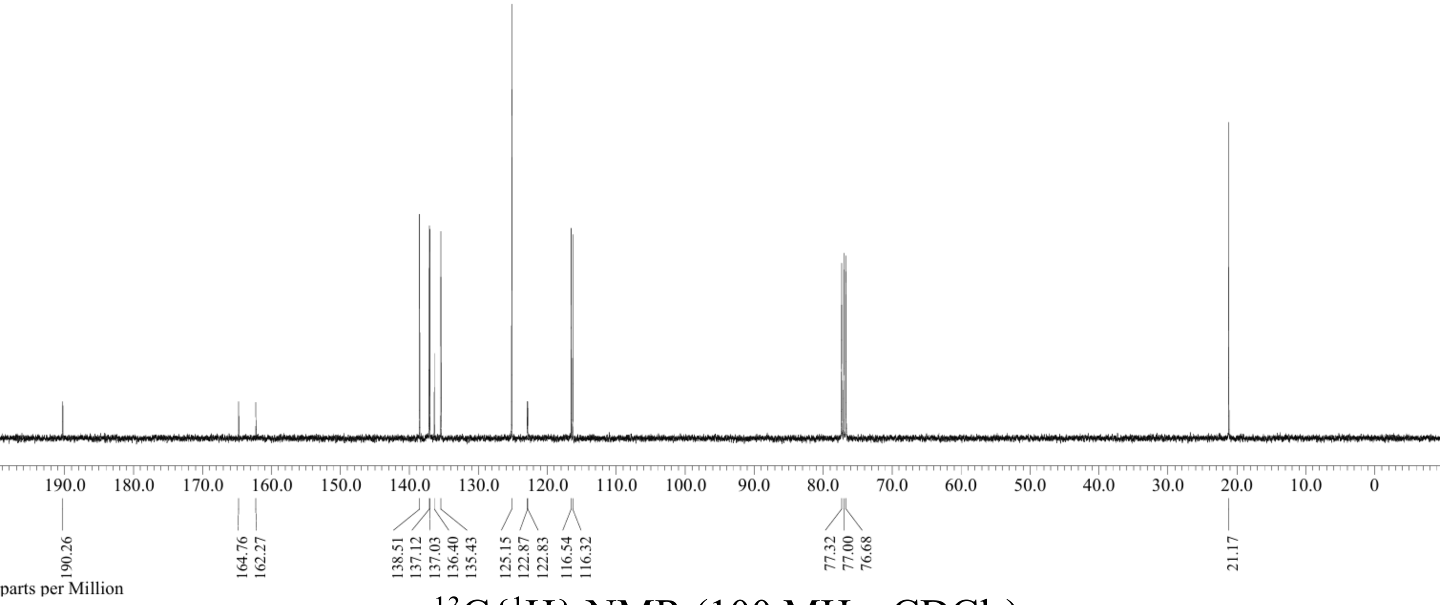

$^{13}C\{^{1}H\}$ NMR (100 MHz, $CDCl_3$)

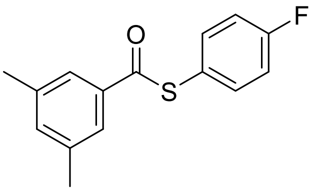

3ae

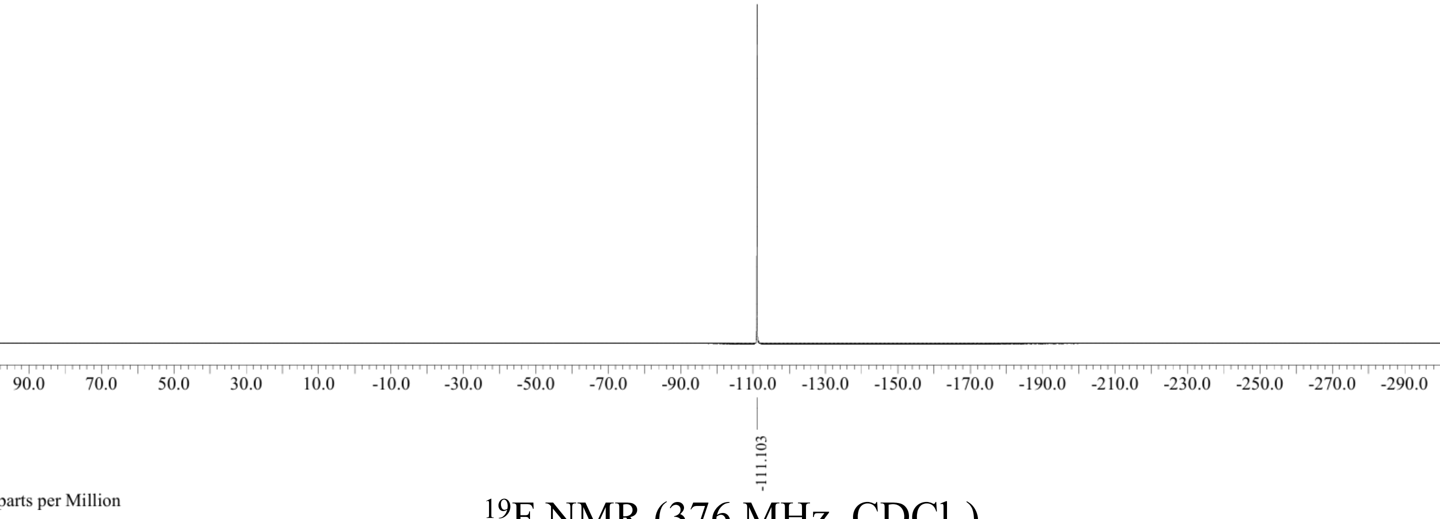

^{19}F NMR (376 MHz, $CDCl_3$)

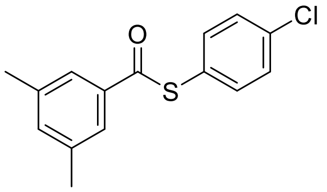

3af

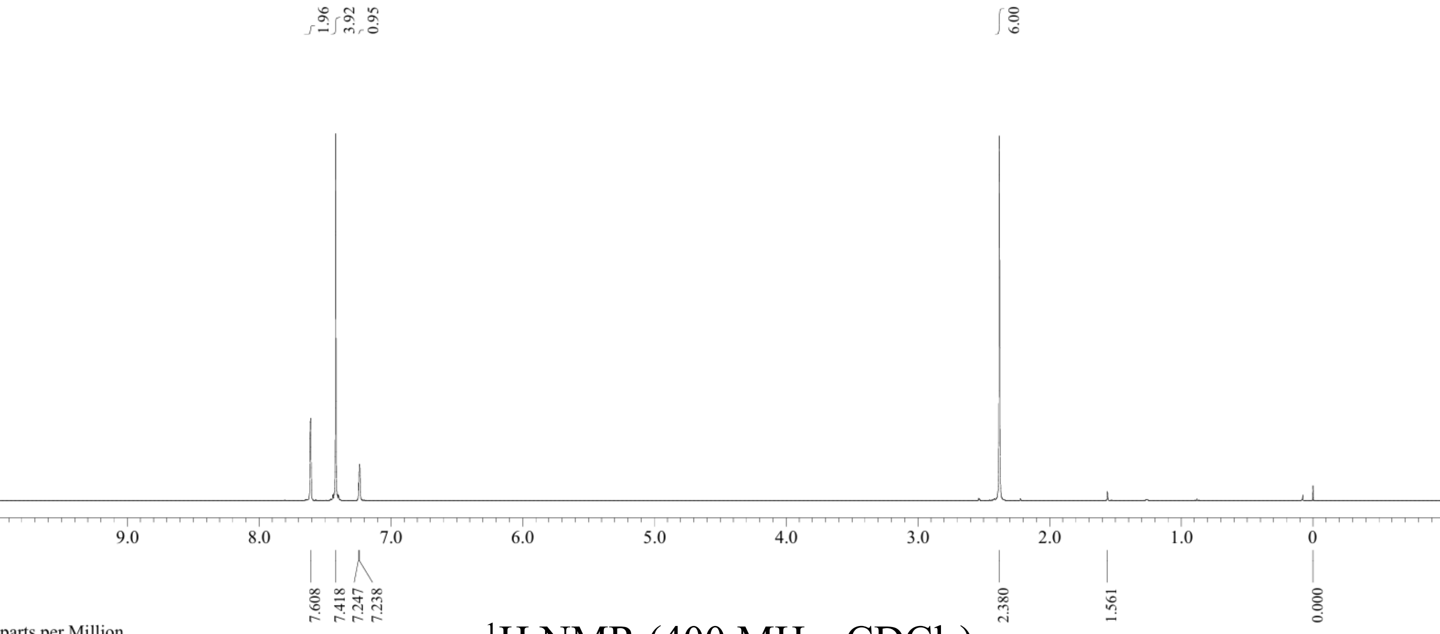

^{1}H NMR (400 MHz, $CDCl_3$)

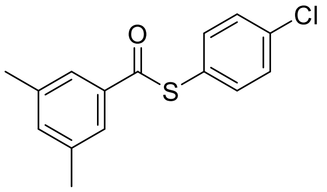

3af

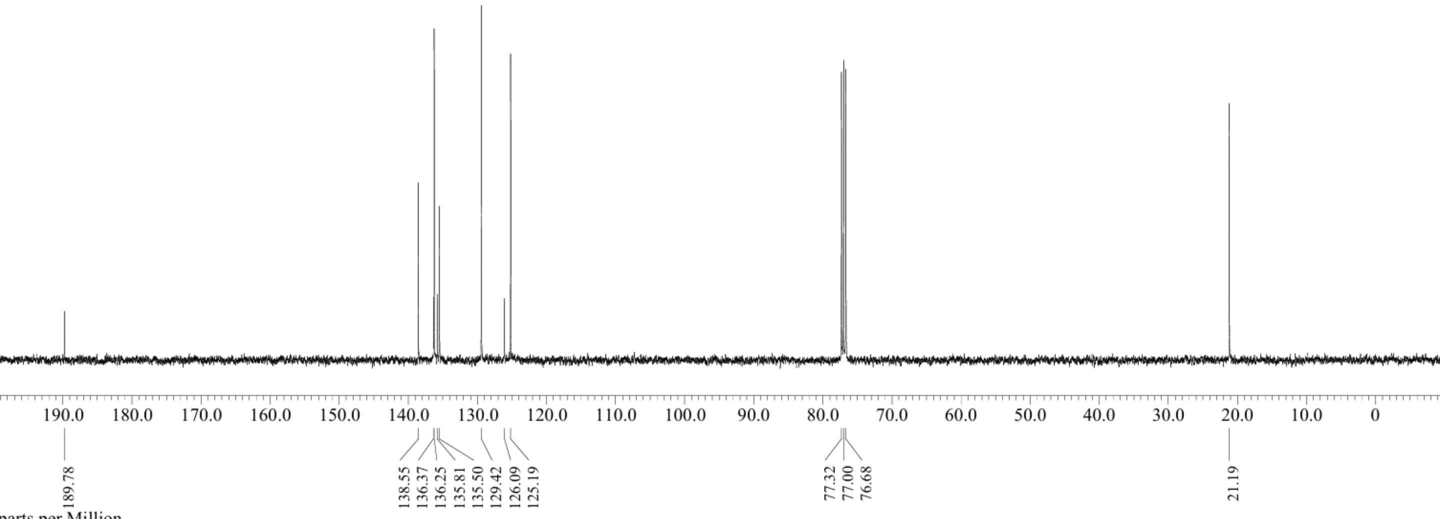

$^{13}C\{^{1}H\}$ NMR (100 MHz, $CDCl_3$)

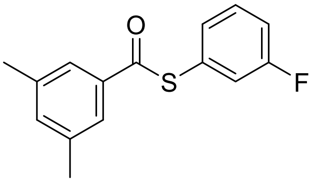

3ag

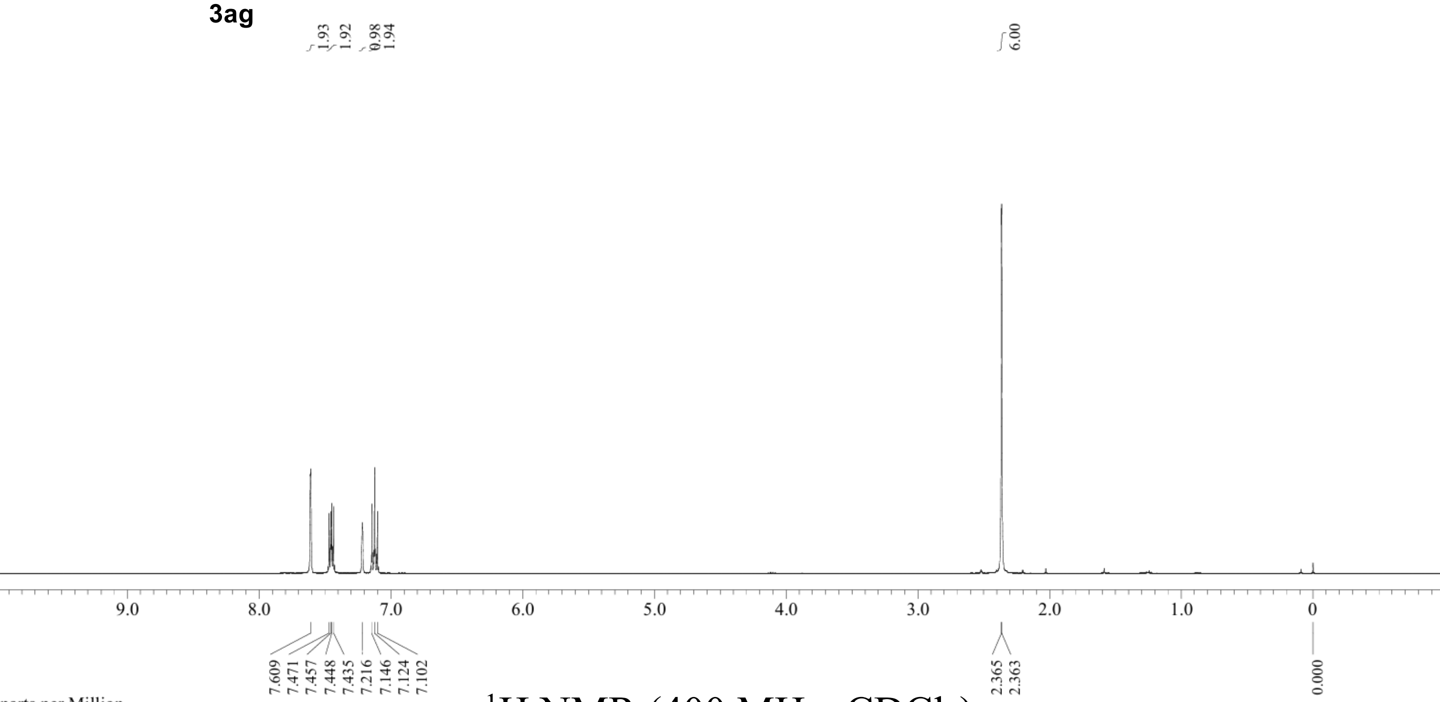

^{1}H NMR (400 MHz, $CDCl_3$)

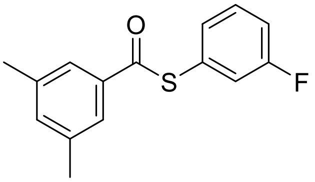

3ag

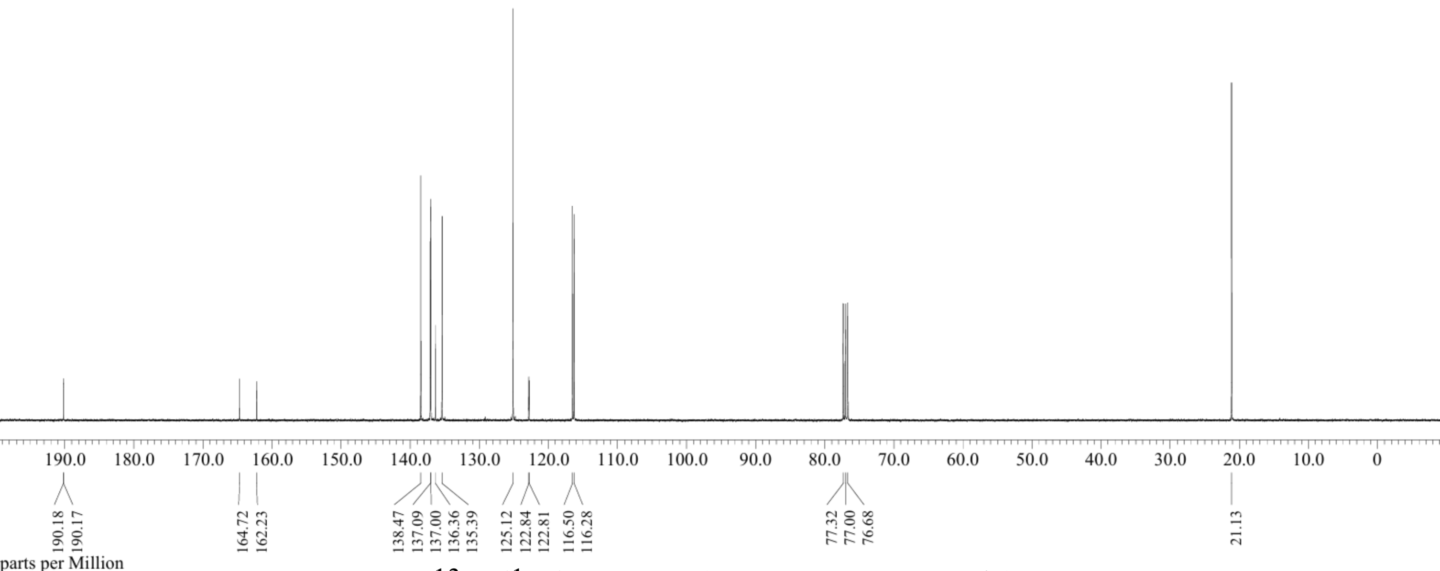

$^{13}C\{^{1}H\}$ NMR (100 MHz, $CDCl_3$)

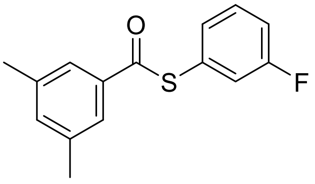

3ag

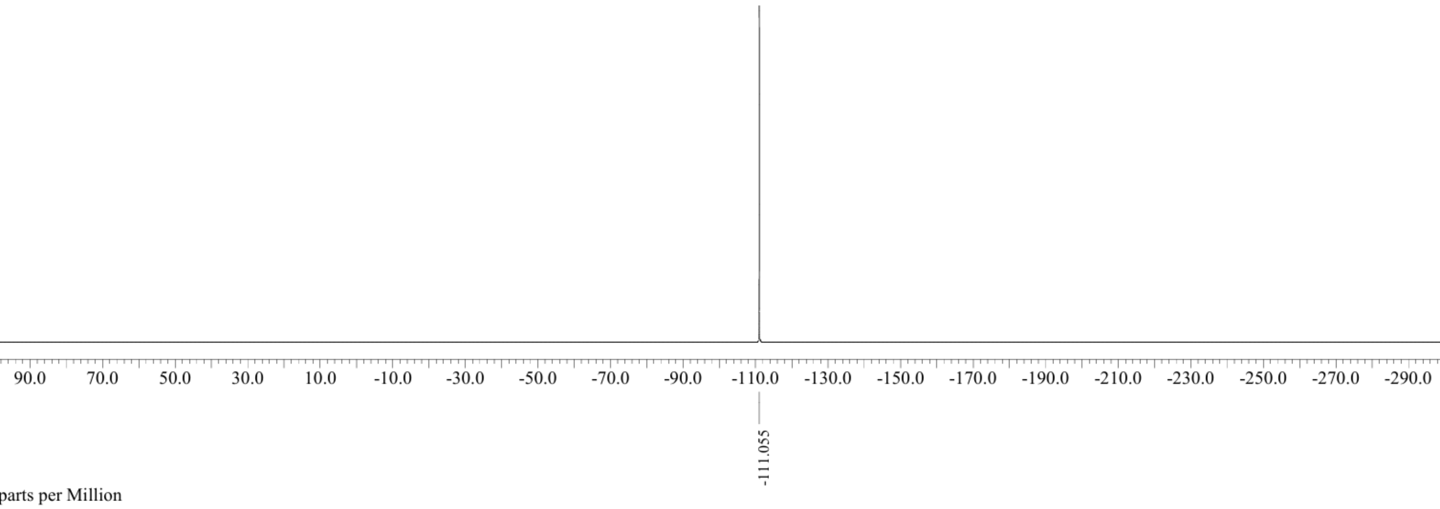

^{19}F NMR (376 MHz, $CDCl_3$)

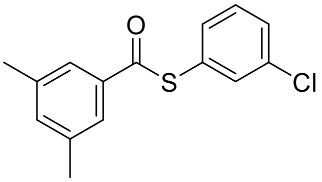

3ah

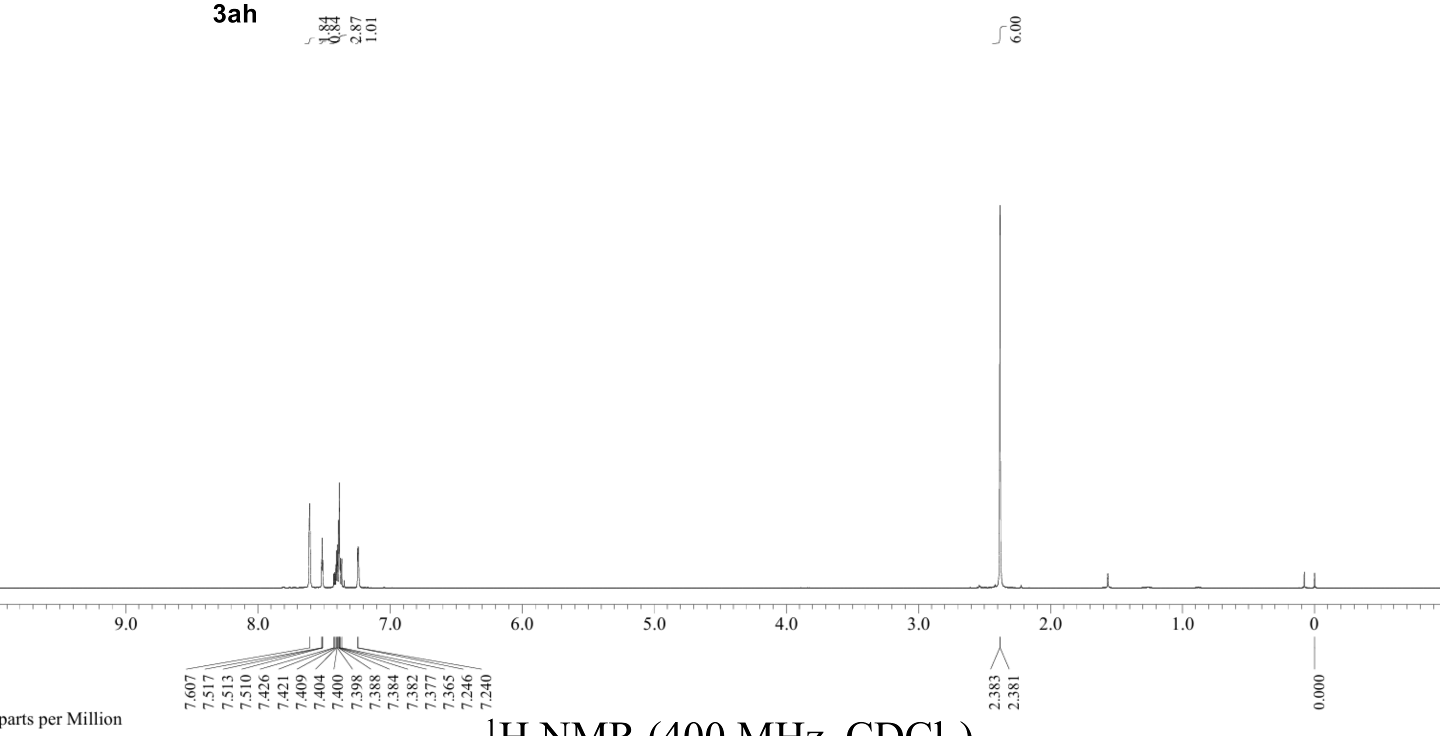

^{1}H NMR (400 MHz, $CDCl_3$)

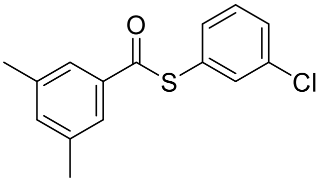

3ah

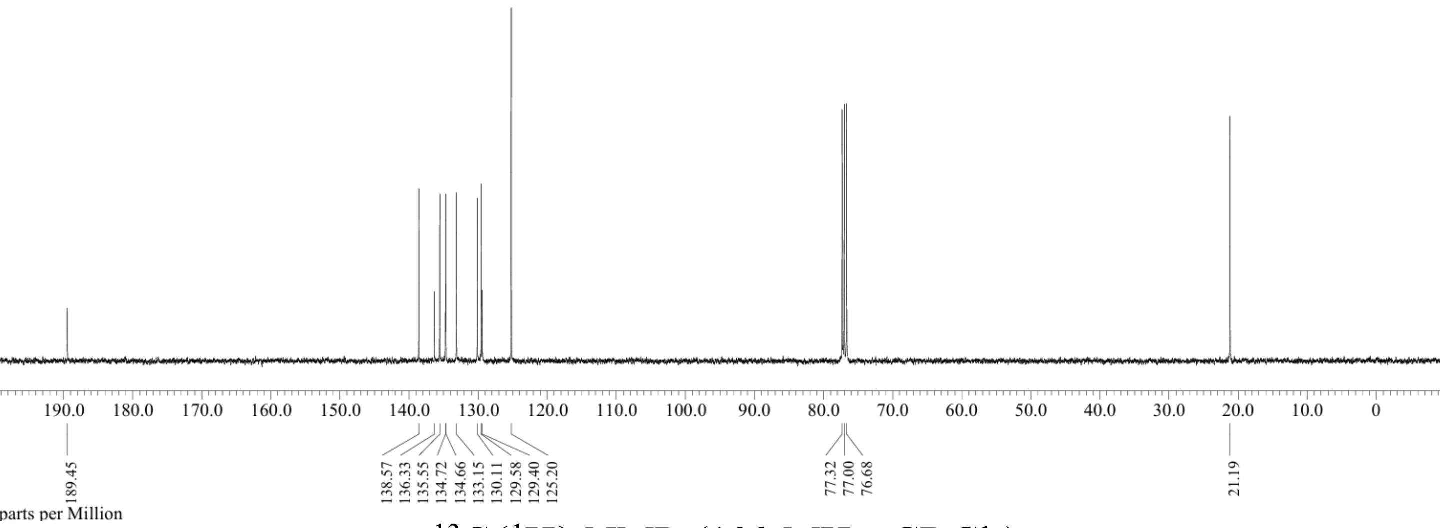

$^{13}C\{^{1}H\}$ NMR (100 MHz, $CDCl_3$)

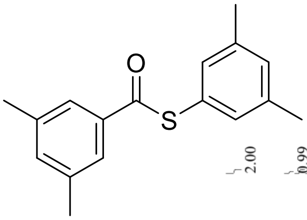

3ai

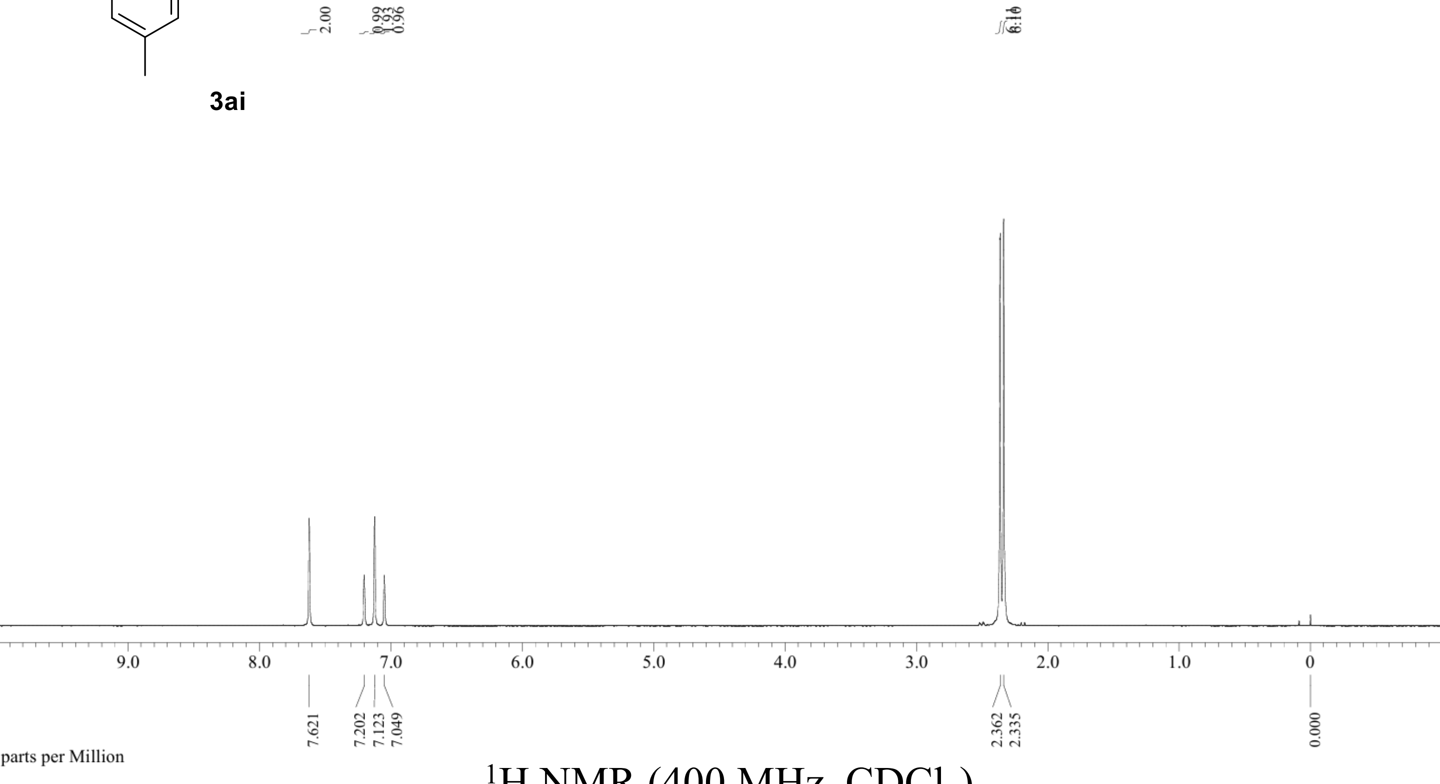

^{1}H NMR (400 MHz, $CDCl_3$)

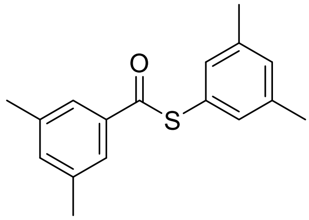

3ai

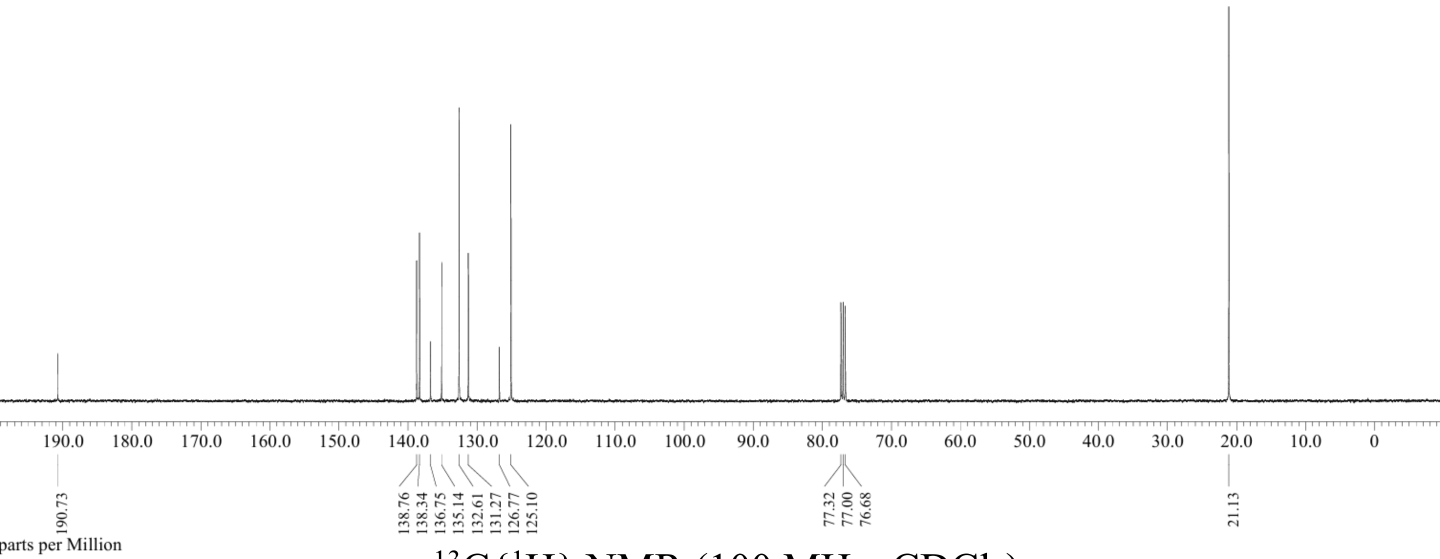

$^{13}C\{^1H\}$ NMR (100 MHz, $CDCl_3$)

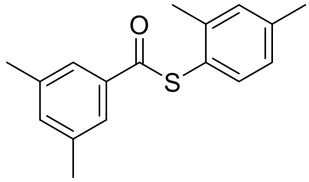

3aj

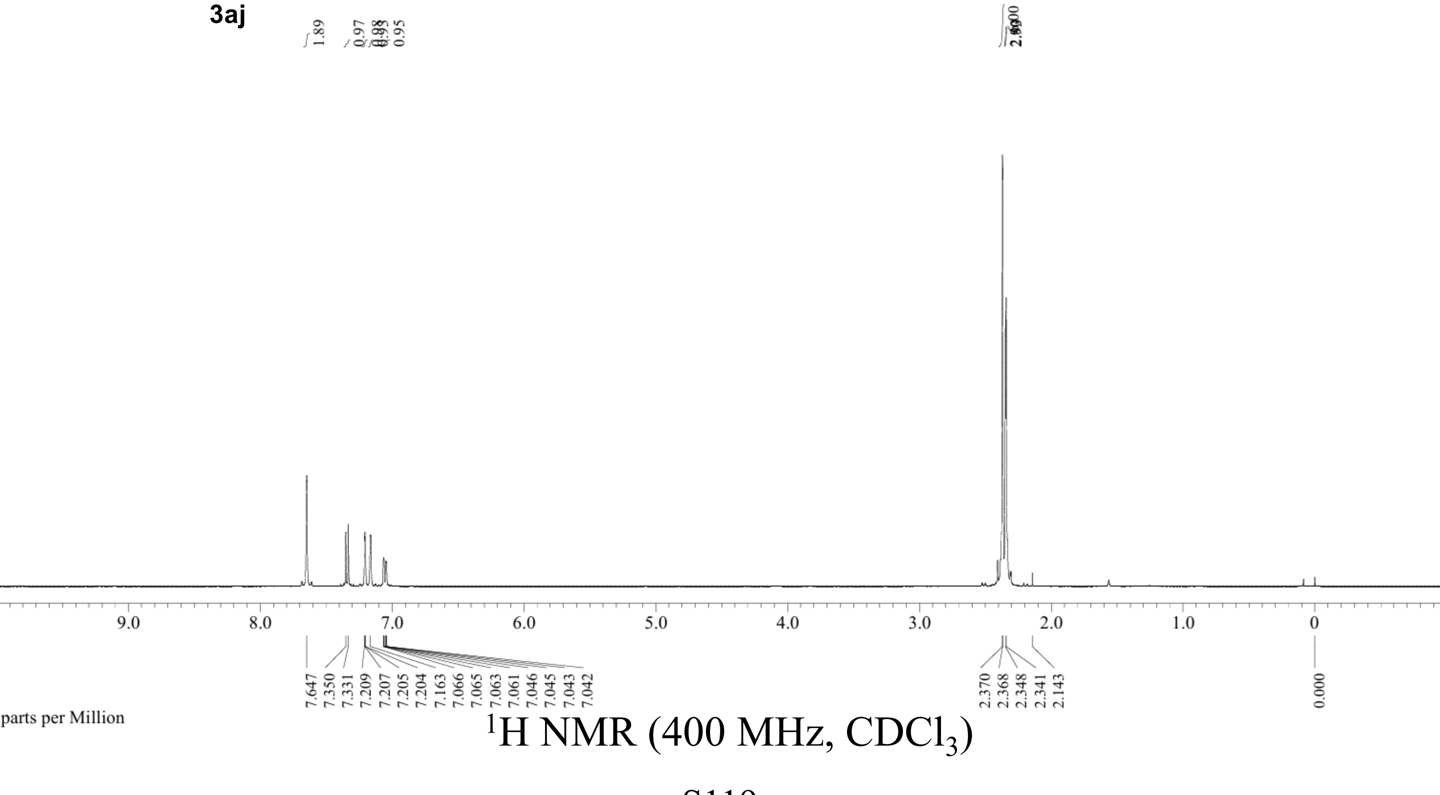

^1H NMR (400 MHz, $CDCl_3$)

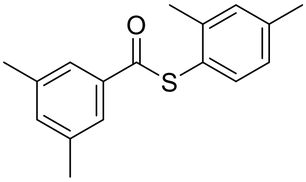

3aj

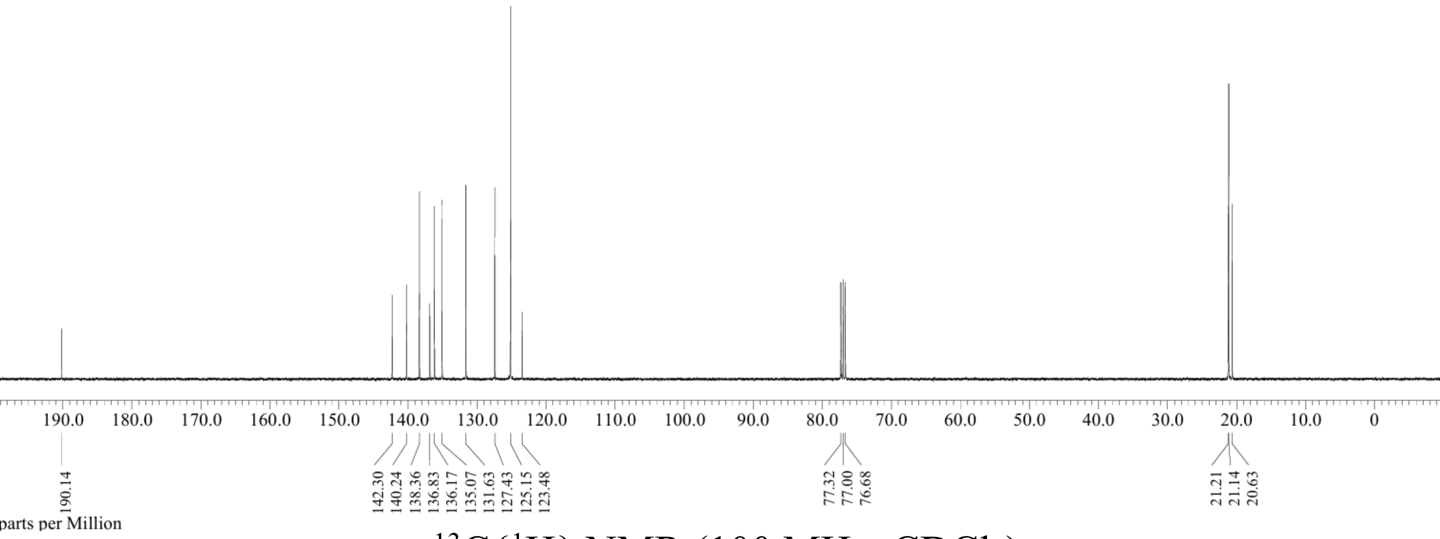

$^{13}C\{^{1}H\}$ NMR (100 MHz, $CDCl_3$)

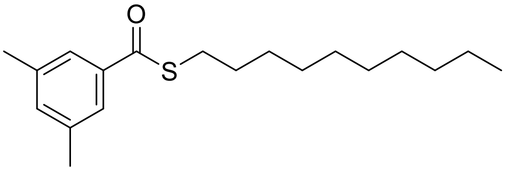

3ak

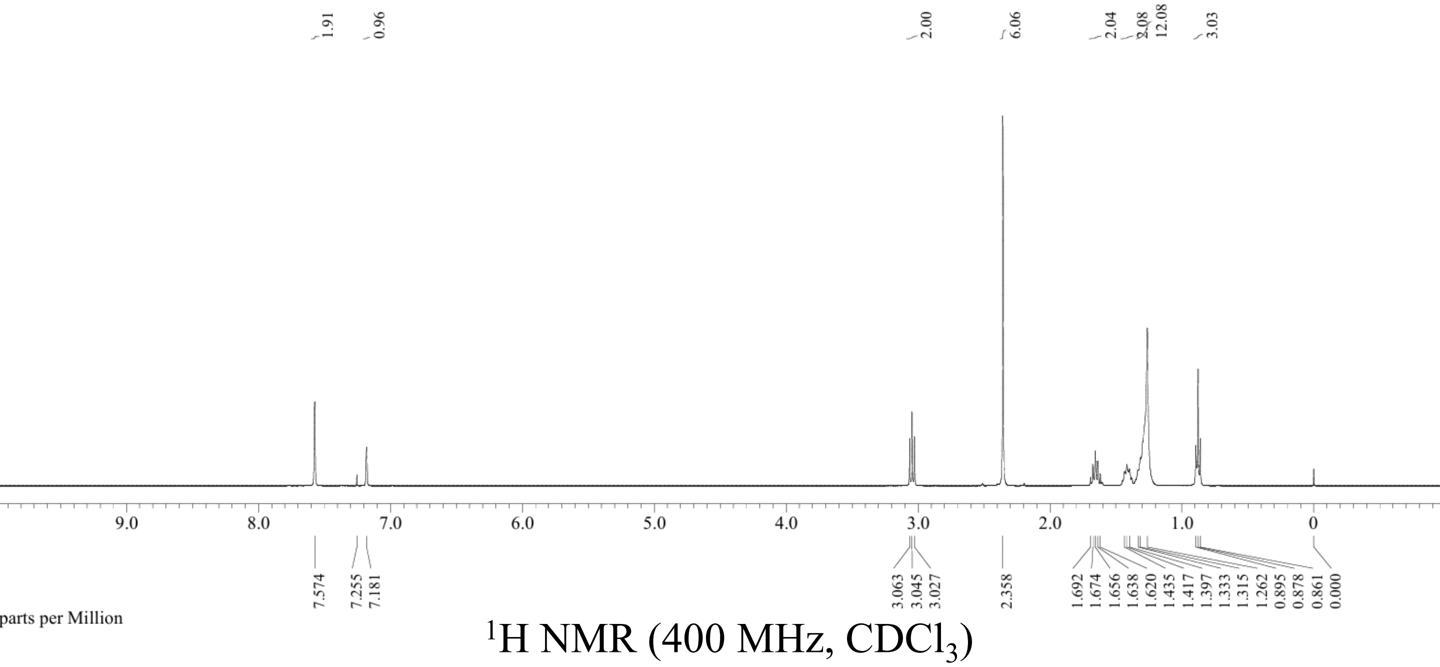

^{1}H NMR (400 MHz, $CDCl_3$)

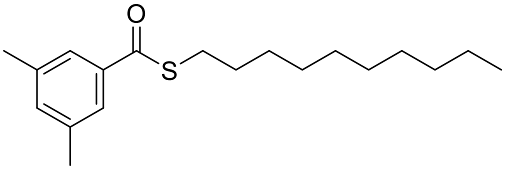

3ak

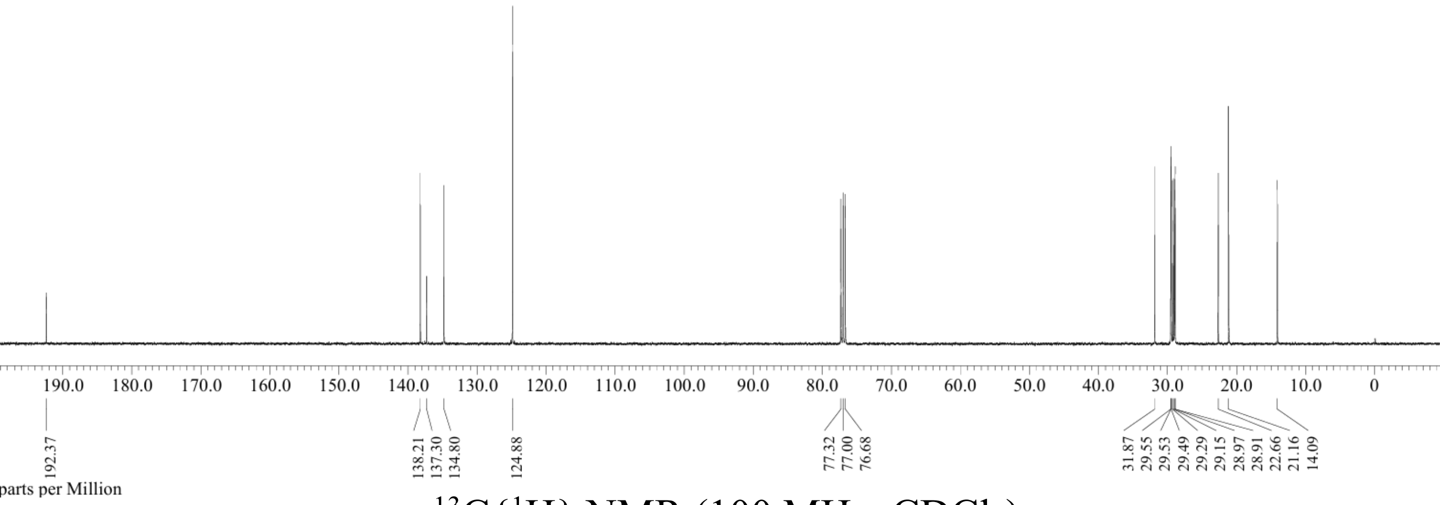

$^{13}C\{^1H\}$ NMR (100 MHz, $CDCl_3$)

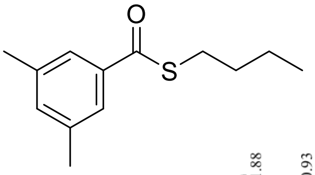

3al

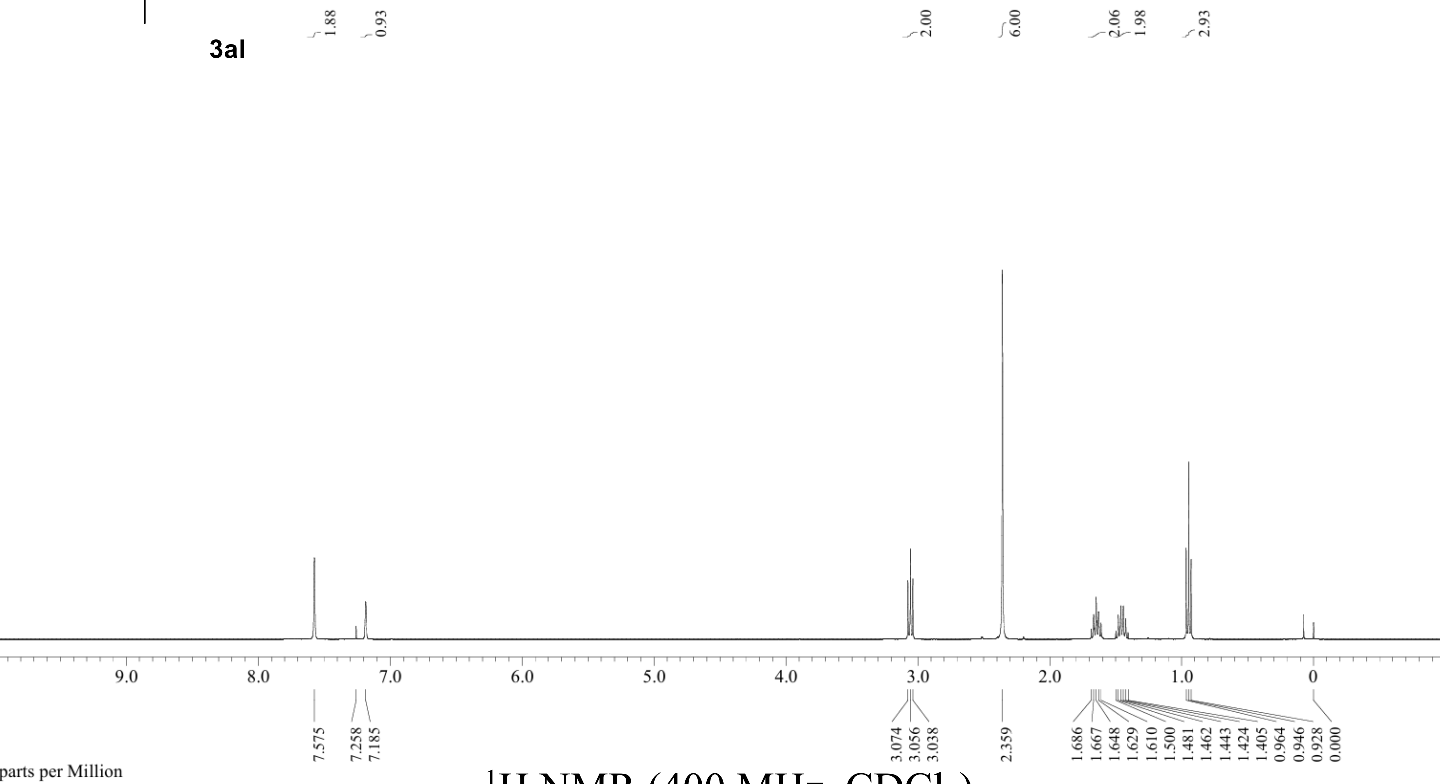

^1H NMR (400 MHz, $CDCl_3$)

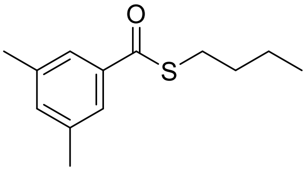

3al

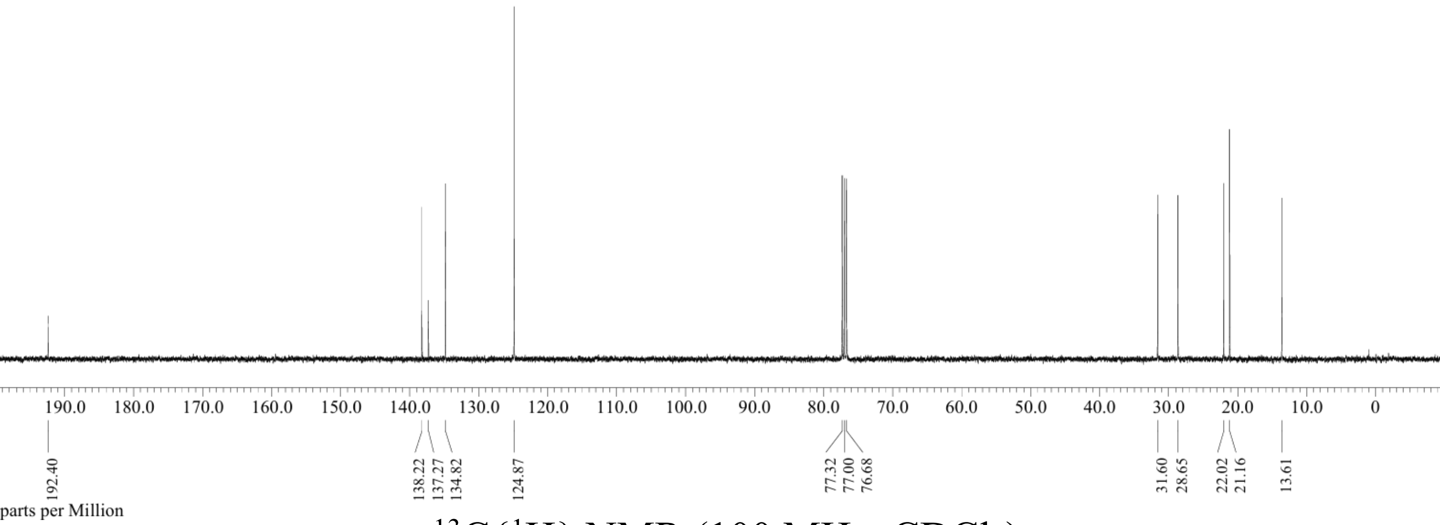

$^{13}C\{^1H\}$ NMR (100 MHz, $CDCl_3$)

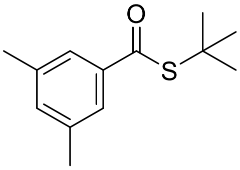

3am

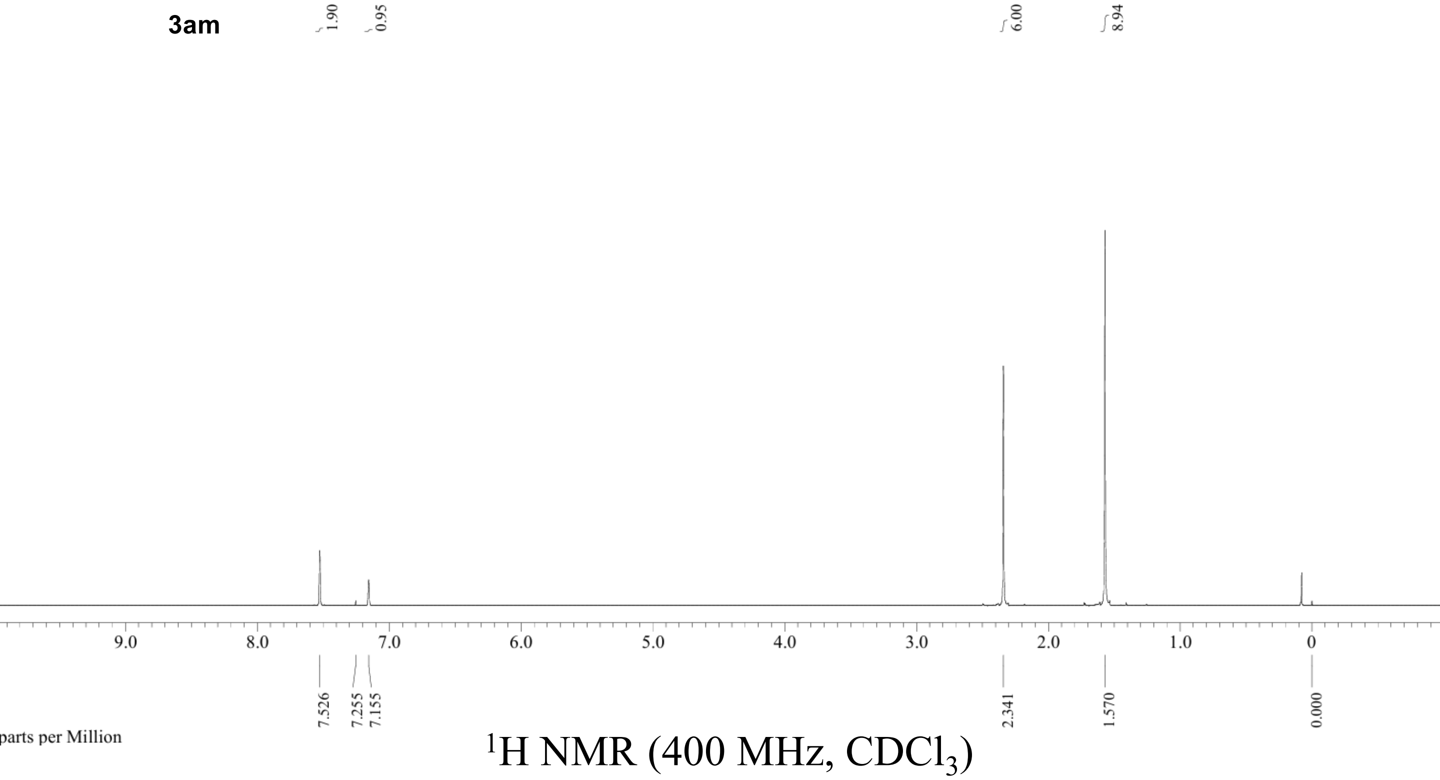

^1H NMR (400 MHz, $CDCl_3$)

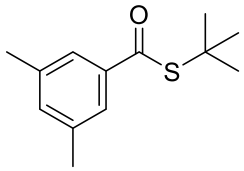

3am

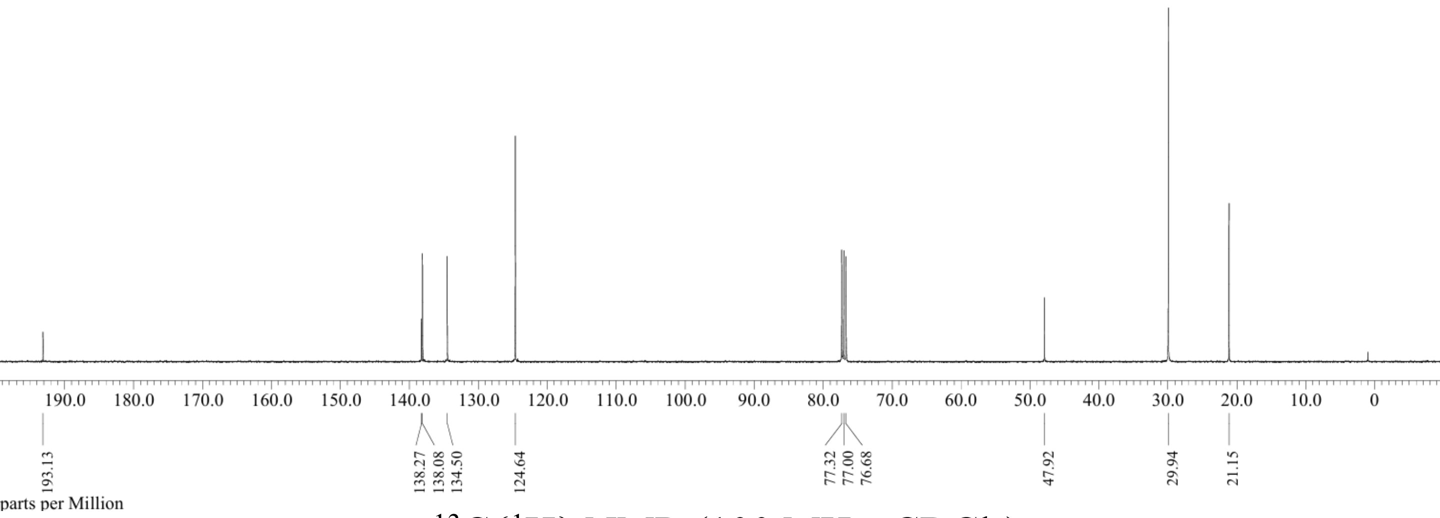

$^{13}C\{^{1}H\}$ NMR (100 MHz, $CDCl_3$)

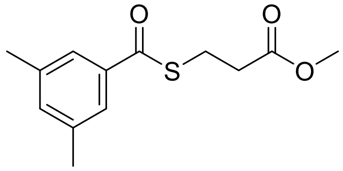

3an

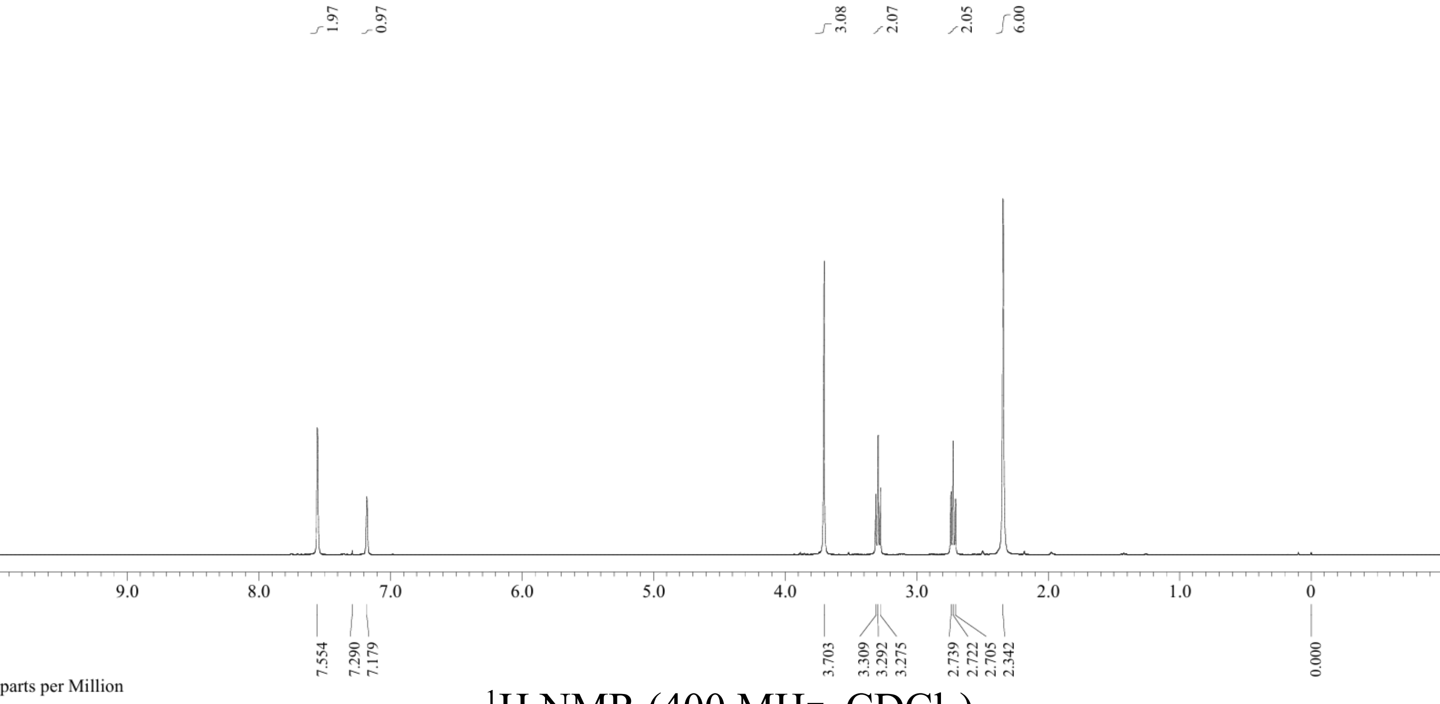

^{1}H NMR (400 MHz, $CDCl_3$)

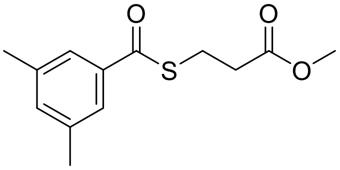

3an

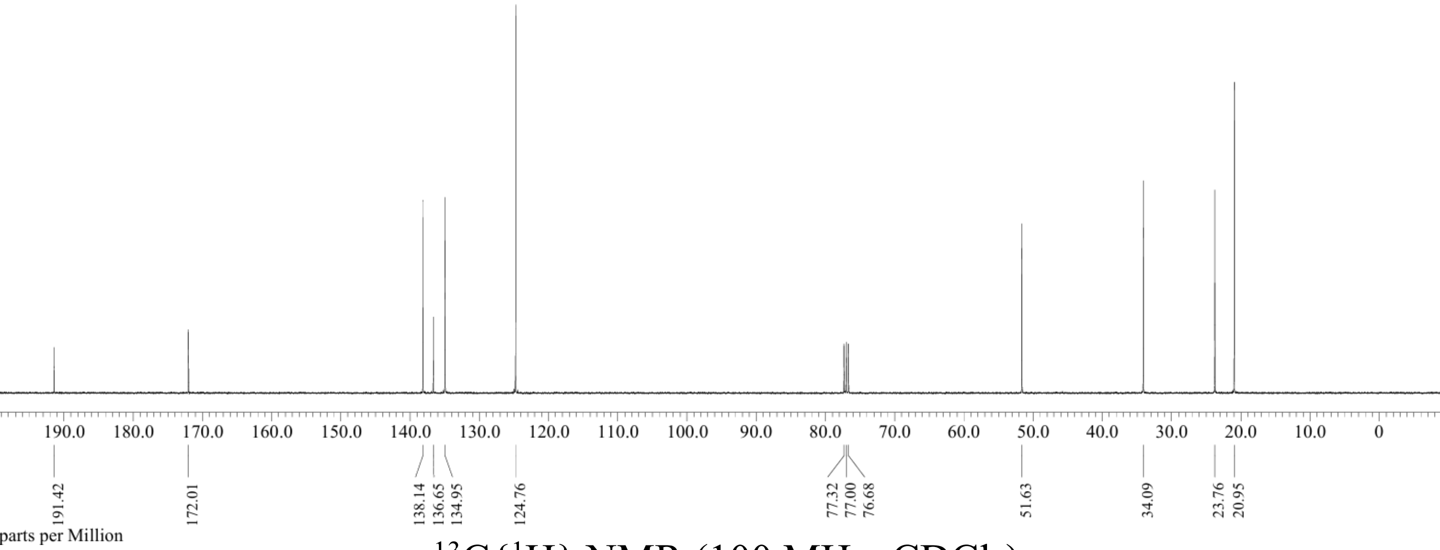

$^{13}C\{^1H\}$ NMR (100 MHz, $CDCl_3$)

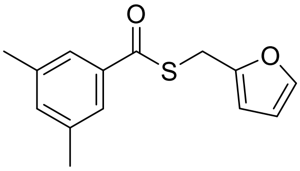

3ao

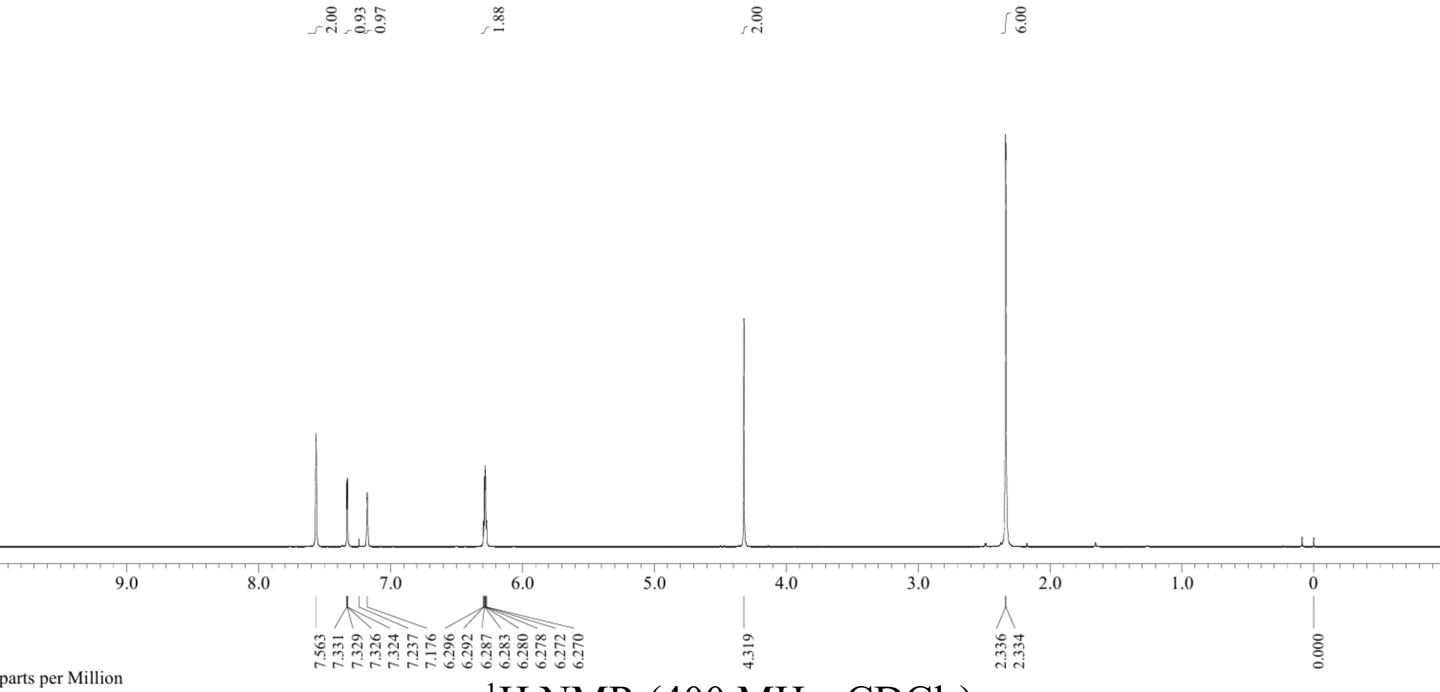

^1H NMR (400 MHz, $CDCl_3$)

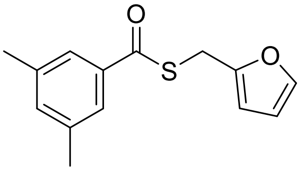

3ao

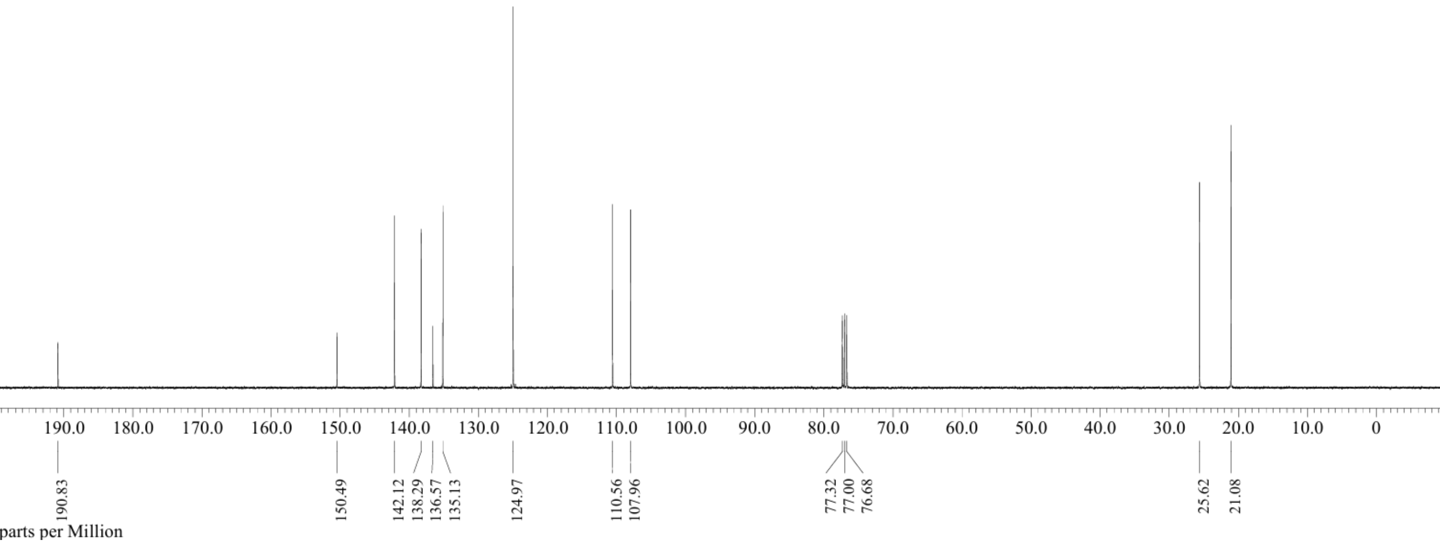

$^{13}C\{^1H\}$ NMR (100 MHz, $CDCl_3$)
